# Adjacent segment degeneration or disease after cervical total disc replacement: a meta-analysis of randomized controlled trials

**DOI:** 10.1186/s13018-018-0940-9

**Published:** 2018-10-03

**Authors:** Shuai Xu, Yan Liang, Zhenqi Zhu, Yalong Qian, Haiying Liu

**Affiliations:** Department of Spinal Surgery, Peking University People’s Hospital, Peking University, No. 11 Xizhimen South Street, Xicheng District, Beijing, People’s Republic of China

**Keywords:** Adjacent segment degeneration, Adjacent segment disease, TDR, ACDF, Meta-analysis

## Abstract

**Background:**

Anterior cervical discectomy and fusion (ACDF) has been widely used in cervical spondylosis, but adjacent segment degeneration/disease (ASD) was inevitable. Cervical total disc replacement (TDR) could reduce the stress of adjacent segments and retard ASD in theory, but the superiority has not been determined yet. This analysis aimed that whether TDR was superior to ACDF for decreasing adjacent segment degeneration (ASDeg) and adjacent segment disease (ASDis).

**Methods:**

A meta-analysis was performed according to the guidelines of the Cochrane Collaboration with PubMed, EMBASE, Cochrane Library and CBM (China Biological Medicine) databases. It included randomized controlled trials (RCTs) that reported ASDeg, ASDis, and reoperation on adjacent segments after TDR and ACDF. Two investigators independently selected trials, assessed methodological quality, and evaluated the quality of this meta-analysis using the grades of recommendation, assessment, development, and evaluation (GRADE) approach.

**Results:**

Eleven studies with 2632 patients were included in the meta-analysis. The overall rate of ASD in TDR group was lower than ACDF group (OR = 0.6; 95% CI [0.38, 0.73]; *P* < 0.00001). Both the incidence of ASDeg and the reoperation rate were statistically lower in the TDR group than in the ACDF group (OR = 0.58, *P* < 0.00001; OR = 0.52, *P* = 0.01, respectively). Subgroup analysis was performed according to the follow-up time and trial site; the rate of ASDeg was lower in patients underwent TDR no matter the follow-up time, and TDR tended to increase the superiority across time. The rate of ASDeg was also lower with TDR both in the USA and China (*P* < 0.0001, *P* = 0.03, respectively). But the cost-effectiveness result might be prone to neither of the two surgery approaches. According to GRADE, the overall quality of this meta-analysis was moderate.

**Conclusions:**

TDR decreased the rates of ASDeg and reoperation compared with that of ACDF, and the superiority may become more apparent over time. We cautiously and slightly suggest adopting TDR according to the GRADE but may not believe it excessively.

**Electronic supplementary material:**

The online version of this article (10.1186/s13018-018-0940-9) contains supplementary material, which is available to authorized users.

## Introduction

Anterior cervical discectomy and fusion (ACDF) is widely performed for the treatment of cervical diseases, estimated to provide relief for more than 90% of radicular and myelopathic complaints [1]. However, ACDF has been associated with the development of new degeneration at levels adjacent to the fused segments [2, 3]. This operation affected normal cervical spine alignment, and loss of mobility at one functional spinal unit increased the load sustained by the remaining units [4]. Hilibrand et al. [5] classified degeneration of adjacent segments as “adjacent segment degeneration” (ASDeg) and “adjacent segment disease” (ASDis) to formulate unified standards and to avoid confusion when researching these problems. ASDeg was defined as radiographic changes at the adjacent segments, whereas ASDis was used to refer to the development of new clinical symptoms, such as mechanical neck pain or coronal-sagittal imbalance. However, the etiology and symptomatology of these adjacent segment changes have remained intensely controversial. Some experts believe it is the natural progress of cervical disc disease [6–8], while others insist fusion can change the biomechanics of adjacent segments, accelerating adjacent segment degeneration/disease (ASD) [2, 9].

Total disc replacement (TDR) on cervical vertebra has been greatly improved over the last decade. Based on a large amount of biomechanical testing, TDR can theoretically decrease the incidence of adjacent segment degeneration by maintaining normal disc kinematics [10–12]. But few clinical studies, especially controlled trials, have specifically investigated ASDeg, ASDis, or reoperation after TDR or ACDF together with less relevant evidence-based medicine, including several systematic reviews that could not determine whether TDR is superior in ASD due to the poor qualified studies [13–15].

To focus on this issue, many studies on ASD have been published in recent years, and performing meta-analysis is necessary to describe the results. The present study aimed to determine whether TDR is superior to ACDF in reducing the incidence of ASD.

## Materials and methods

We performed this meta-analysis using the guidelines of the Cochrane Collaboration [16].

### Criteria for selecting studies for the meta-analysis

#### Types of studies

In view of the currency of randomized controlled trials (RCTs), the highest-grade evidence, comparing TDR with fusion, only RCTs were evaluated.

#### Types of participants

The study population included patients (>18 years) with radiculopathy or myelopathy cervical spondylosis, or other degenerative diseases. The two treatment groups were similar demographically, with no statistically significant differences on the variables of age, sex, or work status. Patients had failed active conservative management for at least 6 months.

#### Types of interventions

Jawahar et al. [17] indicated the non-statistical significance of single or double segment degenerative disc diseases for the prevalence of ASDeg and ASDis. Therefore, we compared the results of surgical treatment of single or double disc diseases treated by TDR or ACDF.

#### Types of outcomes studied

According to Hilibrand’s [5] definitions of ASDeg and ASDis, the incidence of ASDeg and ASDis can be described as direct results and primary outcomes. Reoperation on adjacent segments indirectly reflected the rate of ASD, and we adopted it as a secondary evaluation standard.

#### Search strategy and selection criteria

The databases used to search included PUBMED, EMBASE, Cochrane Library, CBM (China Biological Medicine Database), CNKI, and Wanfang Data.

Since the first study describing a commercially available TDR device was published in 2002 [18], the range was from January 2002 to December 2017. The following keywords were used: cervical vertebrae, total cervical disc replacement OR TDR OR arthroplasty OR prostheses OR dynamic stabilization device AND anterior cervical discectomy and fusion OR ACDF OR cervical arthrodesis AND “randomized controlled trials.”The inclusion criteria were (1) RCTs of cervical degenerative diseases involving single or double segments underwent TDR and ACDF; (2) definite, diagnostic, and direct evidence for ASD; (3) a minimum of a two-year follow-up; (4) at least a minimum of 30 patients per population; (5) containing specific data information on ASD for meta-analysis. Incompatible studies that may have been excluded were (1) case reports; (2) reviews; (3) studies with follow-up time less than 2 years; and (4) just with undesirable result although referring to ASD such as only a mention on ASD with no specific data, secondary surgeries not totally resulting from ASD, and confounding subjects of ASDis and ASDeg for a hardly data extraction. (Additional file 1: Table S1).

### Data extraction and management

Both reviewers (SX and ZQZ) assessed potentially eligible trials and extracted information independently from each potential study. Any discrepancies were resolved through a third reviewer (YLQ) to reach consensus. Extracted data included the general characteristics and outcome measures. General characteristics included study design, first author, sample size, intervention, and types of artificial total disc. Measures of outcomes included the number of ASDeg or ASDis and reoperation (Additional file 2: File S1).

### Risk of bias assessment

Two investigators independently graded each eligible study. We used the Cochrane Handbook for Systematic Reviews of Interventions, version 5.0 [19] for RCTs. The following domains were assessed: randomization, blinding (of patients, surgeons and assessors), allocation concealment, and follow-up coverage. Each domain of quality assessment was classified as adequate (A), unclear (B), or inadequate (C). If all domains were A, the study was A-level; if at least one domain was B, the study was B-level; if at least one domain was C, the study was C-level (Additional file 3: Table S2).

### GRADE approach

The GRADE (the grades of recommendation, assessment, development, and evaluation) approach was used to evaluate the strength of evidence [20]. Based on parameters, the quality assessment was classified as very low, low, moderate, or high according to the GRADE handbook(version 3.2), with the GRADE profiler software (version 3.6). A Summary of Findings Table (SoF Table) was used to explain the final results.

### Data analysis

Review Manager Software (RevMan Version 5.3) was used to conduct the statistical analysis.

#### Measures of treatment effect

Only dichotomous outcomes were presented in this study; the odds ratio (OR) and 95% confidence intervals (95% CI) were calculated for outcomes.

#### Assessment of heterogeneity

Results were regarded as statistically significant if *P* < 0.05. *I*^2^ was used to estimate the size of the heterogeneity [21]. *I*^2^ < 50% indicated low heterogeneity, and the results of comparable groups could be pooled using a fixed-effects model.

#### Subgroup analysis

Subgroup analysis that could reduce statistical heterogeneity to facilitate factor definition was worthwhile. If the overall heterogeneity was *I*^2^ < 50%, we could still divide studies into subgroups depending on professional principles and clinical meaning.

#### Bias of publication

We constructed a funnel plot for overall outcomes to assess publication bias and to examine the relationship between sample size and the effect.

There was no protocol.

## Results

### Description of the studies

**Fig. 1:**
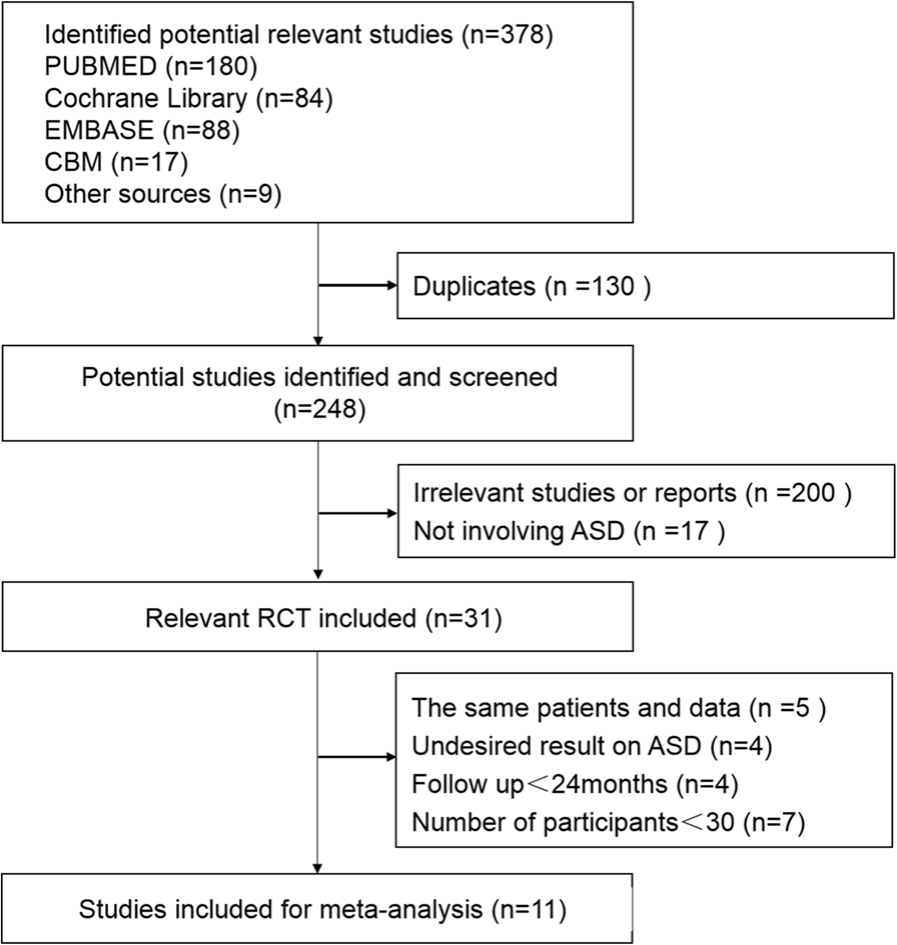
Selection process for meta-analysis of the studies

The process of identifying relevant studies is summarized in Fig 1. Three hundred seventy-eight references were obtained from the databases mentioned and a total of 11 studies [17, 22–31] met inclusion criteria with a total of 2632 patients: 1185 underwent ACDF and 1447 underwent TDR. As some studies were continuations of previous articles, we used the latest publication to avoid duplication. Thus, the search range was from 2002 to 2016, but the 11 included studies were published between 2010 and 2016.We recorded the characteristics of the 11 included RCTs in Table 1.

**Table 1:** Characteristics of the included studies

References	Design	Intervention		Patients		Level		Age(years)		FU (months)	ASDeg		ASDis		Reoperation		Quality^a^
		F	NF	F	NF	F	NF	F	NF		F	NF	F	NF	F	NF	
Phillips, F M(US) [31]	RCT (R:1:1 C:unclear B:unclear L:114/403)	ACDF	TDR (PCM)	185	218	S	S	comparable		60	92	72			11	4	B
Davis, R J(US) [30]	RCT (R:ratio1:2 C:Yes B:patients L:18/330)	ACDF	TDR (Mobi-C)	105	225	D	D	46.2	45.3	48	48	92					B
Zhang, H X(CHA) [29]	RCT (R:ratio1:1 C:unclear B:unclear L:0/111)	ACDF	TDR (Mobi-C)	56	55	S	S	46.7	44.8	48					4	0	B
Burkus, J K(US) [28]	RCT (R:ratio1:1 C:Yes B:patients L:146/541)	ACDF	TDR (Prestige)	265	276	S	S	43.9	43.3	84					10	8	B
Li, Z H(CHA) [27]	RCT (R:ratio1:1 C:unclear B:unclear L:0/81)	ACDF	TDR (Scient’x)	42	39	S	S	49.5	45.3	27	6	5					C
Guan, T (CHA) [26]	RCT (R:ratio1:1 C:unclear B:unclear L:6/66)	ACDF	TDR (Active-c)	34	32	S	S	52.6	49.6	34	21	13					B
Tian, W(CHA) [25]	RCT (R:ratio1:1 C:unclear B:unclear L:30/93)	ACDF	TDR (Bryan)	48	45	S/D (23/12)	S/D (20/8)	48.7	45	80	21	12					B
Nunley, P D(US) [24]	RCT (R:ratio1:2 C:unclear B:Patients L:12/182)	ACDF	TDR (unclear)	62	120	S/D (43/19)	S/D (71/29)	43	45	42	18	31	9	19			C
Coric, D(US) [23]	RCT (R:ratio1:1 C:unclear B:unclear L:35/269)	ACDF	TDR (Kineflex-C)	133	136	S	S	43.9	43.7	24	68	42			5	1	B
Sasso, R C(US) [22]	RCT (R:ratio1:1 C:Yes B:Both L:154/463)	ACDF	TDR (Bryan)	221	242	S	S	46.1	42.5	48			9	9	9	9	A
Jawahar, A(US) [17]	RCT (R:randomization number C:unclear B:Patients L:29/93)	ACDF	TDR (Kineflex-C/Mobi-C/Advent)	34	59	S/D (28/6)	S/D (43/16)	comparable		37			6	9			B

*Abbreviations: RCT* randomized controlled trial, *R* randomization, *C* concealment of allocation, *B* blinding, *L* losses to follow-up, *ACDF* anterior cervical discectomy and fusion, *TDR* total disc replacement, *S* single level, *D* double levels, *FU* follow-up, *ASDeg* adjacent segment degeneration, *ASDis* adjacent segment disease

^a^*Quality* was classified as A level (A), B level (B), or C level (C) by Cochrane Handbook for Systematic Reviews of Interventions

### Risk of bias in the studies

**Fig. 2:**
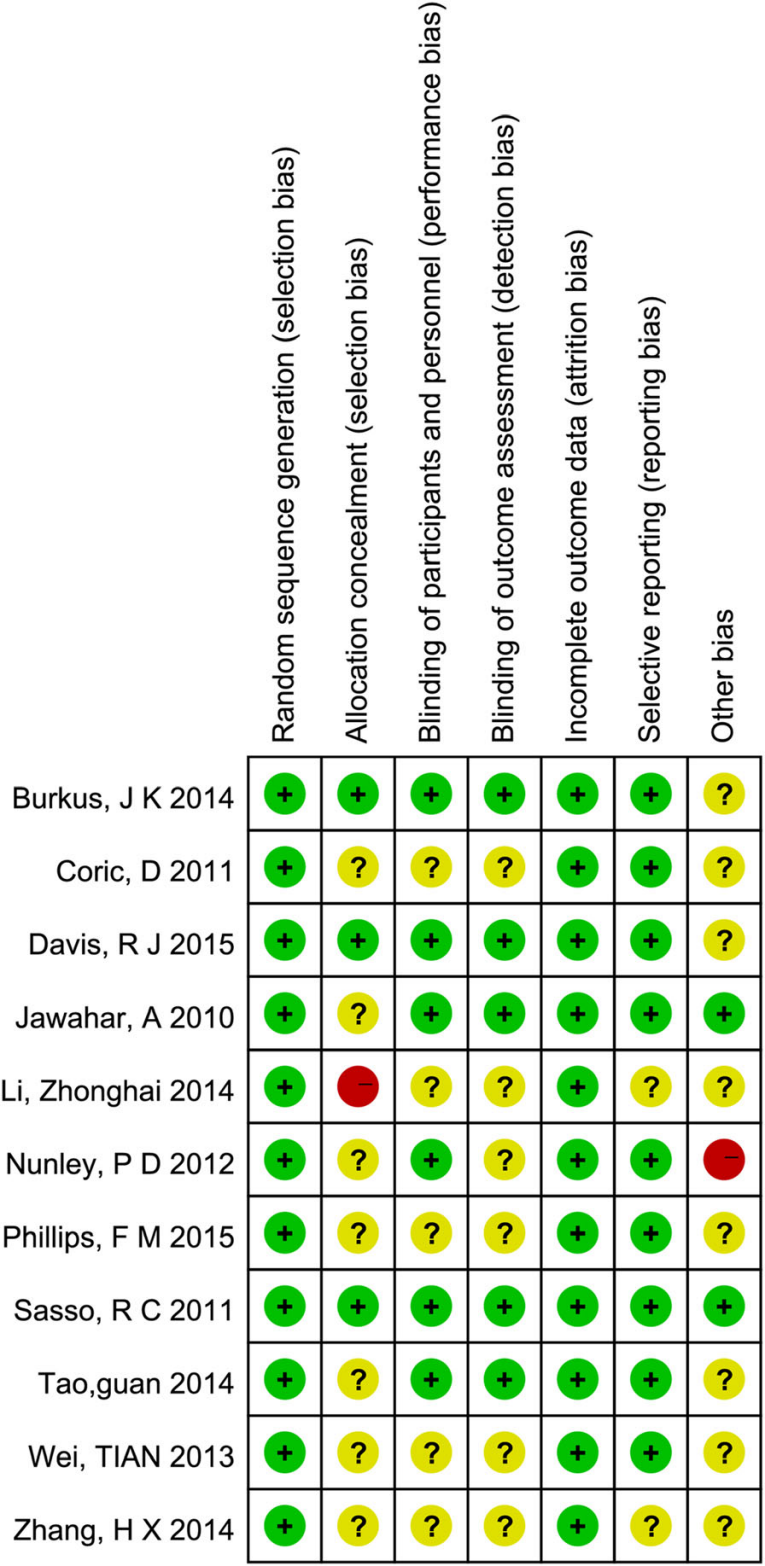
Risk of bias summary. The review authors’ judgments about each risk of bias item for each included study: + is “yes”, − is “no”, ? is “unclear”

According to the quality assessment criteria recommended by the Cochrane Handbook for Systematic Reviews of Interventions [19], 9 out of 11 were of high quality. One study was A-level quality [22], 8 articles were B-level [17, 23, 25, 26, 28–31], and 2 articles were C-level [24, 27] Fig. 2. The review authors’ judgments about each risk of bias item for each included study: + is “yes”, − is “no”, ? is “unclear”.

### Measures of overall outcomes

In this meta-analysis, the rates of ASDeg and ASDis were described as the direct outcomes, reoperation on adjacent segments was adopted as the indirect standard. All 11 RCTs used unified standards on ASD in line with Hilibrand’s [5] definitions. None of the studies simultaneously involved the three results of the rates of ASDeg, ASDis, and reoperation; 7 RCTs [23–27, 30, 31] mentioned ASDeg, 3 RCTs [17, 22, 24] mentioned ASDis, and 5 RCTs [22, 23, 28, 29, 31] mentioned reoperation. If both direct outcomes and indirect outcomes were involved in a study, the former was preferentially adopted.

**Fig. 3:**
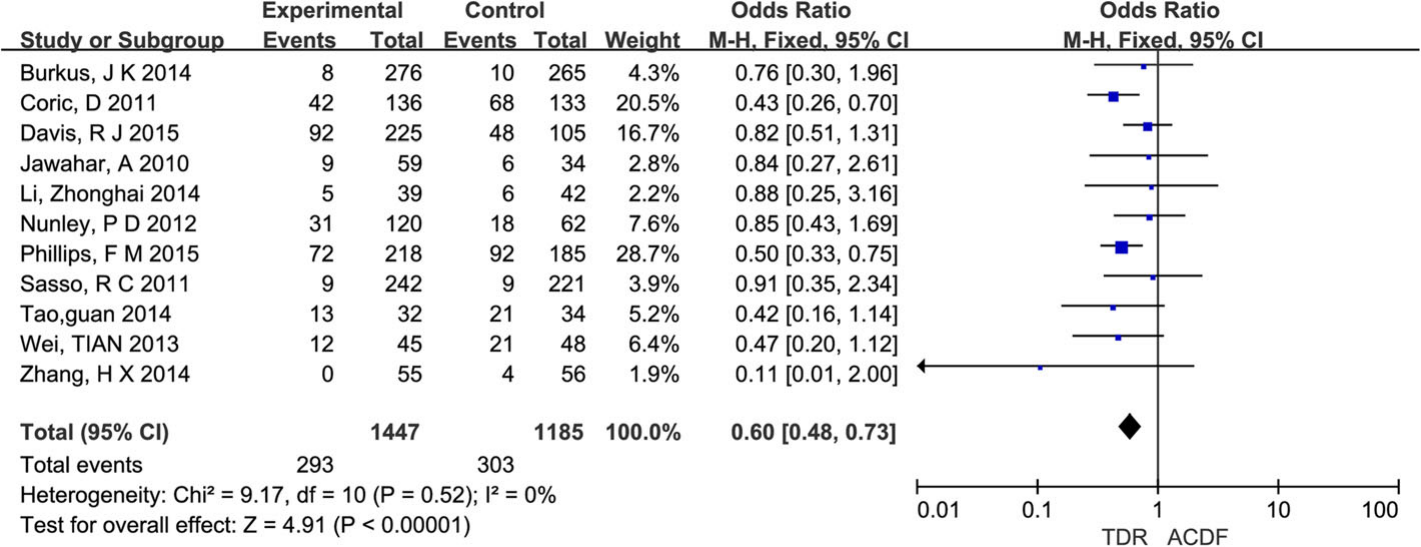
Results of the meta-analysis for the incidence of adjacent segment degeneration/disease and reoperation. *M-H* Mantel–Haenszel, *CI* confidence interval

After a meta-analysis, there was no statistical heterogeneity among all 11 studies (*I*^2^ = 0%). With the fixed-effects model, the overall rate of ASD was lower in the TDR group (20.2%) compared with ACDF group (25.6%), and the difference was statistically significant (OR = 0.6; 95% CI [0.38, 0.73]; *P* < 0.00001) with no heterogeneity (*I*^2^ = 0%), which was showed in Fig. 3.

#### Adjacent segment degeneration (ASDeg)

**Fig. 4:**
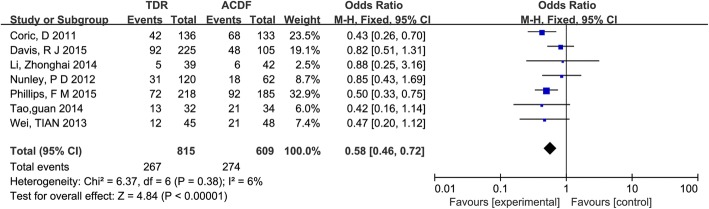
Results of the meta-analysis for adjacent segment degeneration (ASDeg).*M-H* Mantel–Haenszel, *CI* confidence interval

Seven studies [23–27, 30, 31] reported ASDeg. The rate of ASDeg was lower in patients who underwent TDR, and the difference was statistically significant (OR = 0.58, 95% CI [0.46, 0.72]; *P* < 0.00001), which was showed in Fig. 4.

#### Adjacent segment disease (ASDis)

**Fig. 5:**
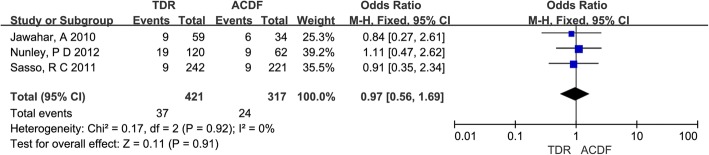
Results of the meta-analysis for adjacent segment disease (ASDis). *M-H* Mantel–Haenszel, *CI* confidence interval

Three studies [17, 22, 24] reported ASDis. The rate of ASDis was similar in two groups (8.8%, 7.6% respectively) with no statistical significance (OR = 0.97, 95% CI [0.56, 1.69]; *P* = 0.91) and it is shown in Fig. 5.

#### Reoperation

**Fig. 6:**
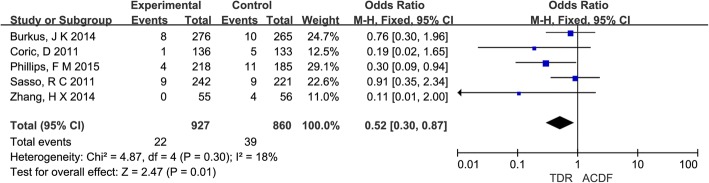
Results of the meta-analysis for reoperation for adjacent segments. *M-H* Mantel–Haenszel, *CI* confidence interval

Five studies [22, 23, 28, 29, 31] and reported reoperation on adjacent segments. The reoperation rate was lower in patients with TDR (2.4%) than in patients who underwent ACDF (4.5%) (OR = 0.52, 95% CI [0.30, 0.87]; *P* = 0.01), which is shown in Fig. 6.

### Subgroup analysis

Subgroup analysis was performed according to follow-up time. Table 2 listed the average follow-up time spanned 24–84 months. We divided the follow-up time into two periods: < 5 years and ≥ 5 years.

**Table 2:** Subgroup analysis according to follow-up time

FU	<5y						≥5y					
	OR [95%CI]	*P* value	I^2^(%)	NO. of P	NO. of ASD	NO. of S	OR [95%CI]	P value	I^2^(%)	NO. of P	NO. of ASD	NO. of S
ASDeg	0.63 [0.48, 0.84]	0.001	23	928	344	5	0.49 [0.34, 0.71]	0.0002	0	496	197	2
ASDis	0.99 [0.57, 1.73]	0.98	0	738	61	3	–	–	–	–	–	0
Reop	0.52 [0.24, 1.13]	0.1	40	843	28	3	0.51 [0.25, 1.04]	0.07	35	944	33	2

*Abbreviations: FU* follow-up, *ASDeg* adjacent segment degeneration, *ASDis* adjacent segment disease, *Reop* Reoperation, *NO. of P* the number of patients, *NO. of ASD* the number of ASD, *NO. of S* the number of studies

Eight studies involving < 5 years of follow-up [17, 22–24, 26, 27, 29, 30] showed that the rate of ASDeg was lower in TDR (*P* = 0.001), but the rates of ASDis and reoperation were not significantly different (*P* = 0.98 and *P* = 0.1, respectively). Three studies involving ≥ 5 years of follow-up [25, 28, 31] showed that the rate of ASDeg was much lower in TDR group (*P* = 0.0002).

Subgroup analysis was also performed according to the trial sites. The trial sites in the 11 RCTs were from two countries: the USA and China. We divided 11 studies into two subgroups in Table 3.

**Table 3:** Subgroup analysis according to study sites

SITE	U.S.						CHINA					
	OR [95%CI]	P value	I^2^(%)	NO. of P	NO. of ASD	NO. of S	OR [95%CI]	P value	I^2^(%)	NO. of P	NO. of ASD	NO. of S
ASDeg	0.59 [0.46, 0.75]	0.0001	44	1184	463	4	0.52 [0.29, 0.92]	0.03	0	240	78	3
ASDis	0.99 [0.57, 1.73]	0.98	0	738	61	3	–	–	–	–	–	0
Reop	0.56 [0.32, 0.96]	0.03	11	1676	57	4	0.11 [0.01, 2.00]	0.13	–	111	4	1

*Abbreviations: FU* follow-up, *ASDeg* adjacent segment degeneration, *ASDis* adjacent segment disease, *Reop* Reoperation, *NO. of P* the number of patients, *NO. of ASD* the number of ASD, *NO. of S* the number of studies

Seven studies performed in the USA [17, 22–24, 28, 30, 31] showed the rate of ASDeg and the reoperation rate for adjacent segments were lower on TDR (*P* < 0.0001 and *P* = 0.03, respectively) but not ASDis (*P* = 0.98). Four studies performed in China [25–27, 29] showed the rate of ASDeg was lower in patients who underwent TDR than ACDF (*P* = 0.03) but not the rate of reoperation (*P* = 0.13).

**Fig. 7:**
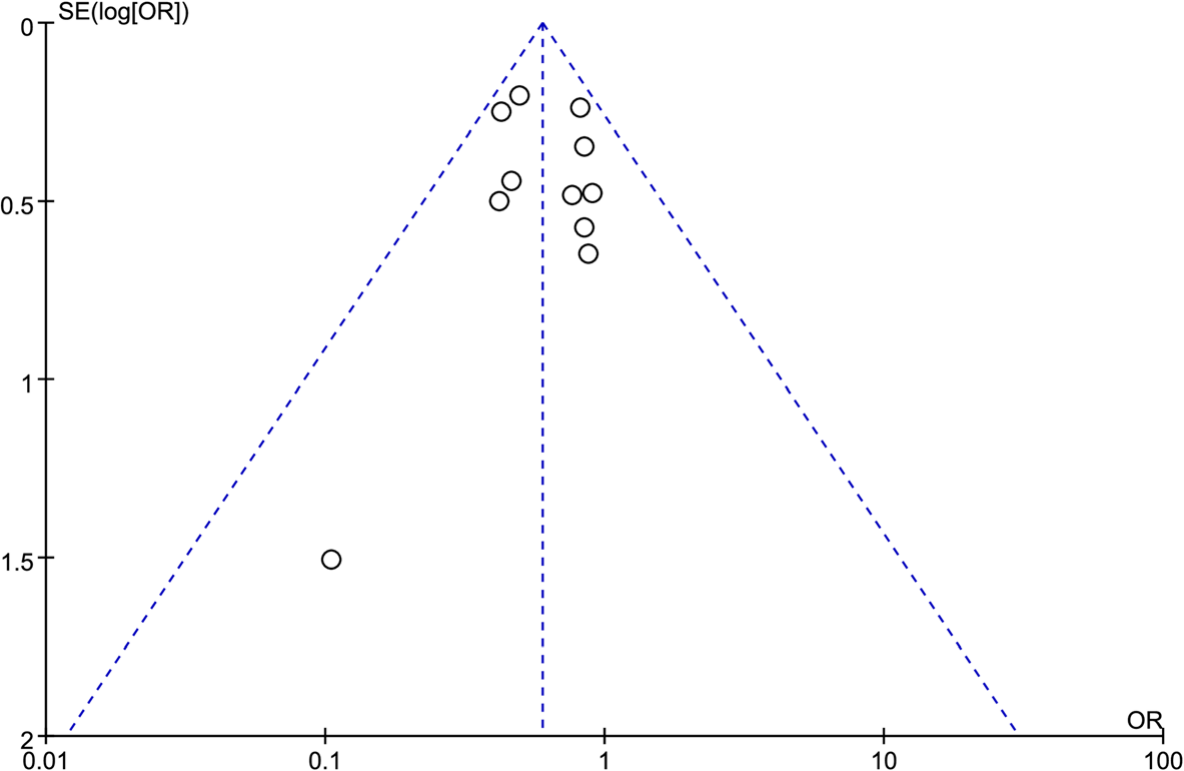
Funnel plot for the occurrence of ASD

The meta-analysis of ASDin Fig. 7 showed no evidence of publication bias.

### The GRADE of this meta-analysis

The SoF Table presents the grade of the ultimate outcome (ASD) under the intervention of TDR and ACDF according to academic and clinical experiences as well as the quality grade of this meta-analysis in Table 4. According to the GRADE [20], the grade of the ultimate outcome (ASD) was critical, and the overall grade quality of our meta-analysis was moderate.

**Table 4:** Preview SoF table of the GRADE for this meta-analysis

TDR compared to ACDF for ASD						
Patient or population: patients with ASD Settings: Intervention: TDR Comparison: ACDF						
Outcomes	Illustrative comparative risks^a^ (95% CI)		Relative effect (95% CI)	No of participants (studies)	Quality of the evidence (GRADE)	Comments
	Assumed risk	Corresponding risk				
	ACDF	TDR				
ASD Follow-up: 24–84 months	Study population		OR 0.6 (0.49 to 0.73)	2632 (11 studies)	⊕ ⊕ ⊕⊝ moderate	
	256 per 1000	171 per 1000 (144 to 201)				
	Moderate					
GRADE Working group grades of evidence						
High quality: Further research is very unlikely to change our confidence in the estimate of effect.						
Moderate quality: Further research is likely to have an important impact on our confidence in the estimate of effect and may change the estimate.						
Low quality: Further research is very likely to have an important impact on our confidence in the estimate of effect and is likely to change the estimate.						
Very low quality: We are very uncertain about the estimate.						

*Abbreviations: CI* Confidence interval, *OR* Odds ratio, *GRADE* grading of recommendations assessment, development and evaluation, *TDR* total disc replacement, *ACDF* anterior cervical discectomy and fusion, *ASD* adjacent segment degeneration/disease

^a^The basis for the assumed risk (e.g., the median control group risk across studies) is provided in footnotes. The corresponding risk (and its 95% confidence interval) is based on the assumed risk in the comparison group and the relative effect of the intervention (and its 95% CI)

## Discussion

ACDF has been recognized as a classic surgical treatment of cervical disease [32, 33], but limitations in range of motion, increased stress on adjacent segments made it defective at simulating physiology. Many publications have reported their observations of this and summarized pathological causal factors of ASD [33–35]. Some experts believe it is a natural process; Hilibrand’s results indicated that ASD was indeed a common problem but may reflect the natural history of the underlying cervical spondylosis [5]. Some have suggested that the alignment, curvature, and activity of the cervical spine are relevant factors that result in ASD [33, 35]. Takeshima [36] concluded cervical dynamic change may increase the adjacent intervertebral stress and accelerate degeneration of adjacent segments.

Since 2002, progressive superiority of TDR in biomechanics theory has been applied in clinical practice and the more technological mature is accepted. The mechanical difference between TDR and ACDF made us increasingly concerned about ASD and hereafter meaningful comparison. But poor qualified studies led to lots of bias and many patients unfit to experimental or control group caused unsatisfactory efficacy, in addition substantial costs made it difficult to perform a multi-center RCT.

There were several articles referred to ASD between TDR and ACDF, but few conclusions indicated ASD has radiological or clinical statistical difference [37–39]. Probable reasons were (1) ASD was a natural process unrelated to surgery, corresponding to the view of Hilibrand and Herkowitz [7, 8]; (2) inconsistencies in surgical indications between TDR and ACDF, non-controlled trials, and demographical differences could bring about clear bias. Therefore, RCTs of high grade were optimal to perform a meta-analysis, then prospective cohort studies. Many meta-analysis publications comparing TDR to ASD have had ambiguous outcomes. Yang, B.et al. [14] mentioned the defect of including only five studies without stratification on factors and exclusion of publication bias.

This meta-analysis contained two poor quality studies [24, 27] of inadequate (C) grade, and most were high quality with low risk of bias. Overall, there was a statistically confirmed lower occurrence of ASD in TDR group and TDR could be considered as a treatment of deferring ASD in comparison with ACDF, so was ASDeg and reoperation in subgroup. The positive outcome of rate may be based on cost-effectiveness analysis, patients believed the efficacy of TDR should be better than ACDF with a higher cost, leaving them a bias of no-attribution to ASDis on TDR with relevant symptom and prefer not to selection a second surgery. With extended follow-up, it may be difficult to explain the difference in reoperation rate; Coric, D [23] mentioned it was ambiguous to draw conclusions with fewer patients added lower incidence on ASDis. The rate of ASDis of no significance likely resulted from the inadequate number of positive population, in addition, ASDis occurred postpone as a symptomatology compared with ASDeg, with no difference between the two groups till current endpoint.

In subgroup analysis, the difference in incidence of ASDeg was still statistically different whether it was shorter than 5 years or not. Furthermore, the results were better with 5 years of follow-up than follow-up within 5 years, probably implying that the longer the follow-up, the more superiority in TDR. But Davis, R J [30] mentioned that the underlying mechanism defining the relationship between decreased radiographic degeneration in patients treated with TDR remains uncorrelated, and further long-term follow-up should continue to correlate these results. The incidence of ASDeg was statistically different between TDR and ACDF both in the USA and China, and TDR may have an advantage over ACDF. However, no matter in China or the USA, there are both tendency in clinical trials towards to the positive results with a larger selection bias, and overall, ensuing a certain lack of representation in incidence of ASD, which may be also related to the policy on cost reimbursement.

There were some limitations in this meta-analysis. Eleven studies may still limit our assessment of potential publication bias and more relevant studies should be included. Then, the various types of disc may affect the occurrence of ASD, so it should be stratified for further analysis. However, there were too many types of discs referred in the literatures, 8 kinds mentioned in 11 documents, and the kind of prosthesis mentioned by Nunley, P D is unclear [24] and even the document by Jawahar, A [17] includes three discs (Kineflex-C/Mobi-C/Advent) at the same time. Therefore, it was difficult to process a stratified analysis with inconsistency and disorganization.

In addition, we did not but it was of vital importance to perform a cost-effectiveness analysis, and most countries was dealing with a nearly unaffordable costs of health care. Qureshi SA [40] suggested in single-segment operation between TDR and ACDF indicated TDR must remain functional for at least 14 years to establish greater cost-effectiveness than ACDF. Ament, J D et al. [41, 42] in cost-effectiveness analysis with two-level segments reaffirmed TDR a stability of the model and the sustainability of this intervention. In this meta-analysis, the difference referred to the ASDis between TDR and ACDF is 2.1% and the NNT is 43.5 but 11 articles did not involve the detailed calculations on cost-effectiveness issues. It is of no meaning if we offered huge cost to make up a little disadvantage, and it could have resulted in an overestimation of the effectiveness of interventions.

The overall grade quality of our meta-analysis was moderate according to the GRADE, and we suggest adopting TDR for the reduction of incidence of ASD to a certain degree. TDR can reduce the rate of reoperation and ASDeg compared with ACDF with probable obvious advantages as the time prolonged basing the prerequisites of a larger sample, but the result should be accepted with caution.

## Conclusion

TDR decreased the rates of ASDeg and reoperations compared with ACDF, and the superiority may be more apparent overtime. TDR can be selected purely in terms of mitigation on ASD, but the overall efficacy through cost-effectiveness analysis that values. The overall grade quality of our meta-analysis was moderate according to the GRADE, and we cautiously and slightly suggest adopting TDR.

## Additional files

Additional file 1: **Table S1.** Excluded studies and main reason for exclusion. (PDF 101 kb)

Additional file 2: **File S1.** Original data of 11 included articles. (ZIP 12 mb)

Additional file 3: **Table S2.** The Cochrane Collaboration’s tool for assessing quality and risk of bias. (PDF 266 kb)

## Abbreviations

ACDF: Anterior cervical discectomy and fusion
ASD: Segment degeneration disease
GRADE: Grades of recommendation, assessment, development and evaluation
TDR: Total disc replacement

## References

[1] Robinson RA, Smith GW (2010). Anterolateral cervical disc removal and interbody fusion for cervical disc syndrome. SAS Journal.

[2] Baba H, Furusawa N, Imura S (1993). Late radiographic findings after anterior cervical fusion for spondylotic myeloradiculopathy. Spine.

[3] Wu W, Thuomas KA, Hedlund R (1996). Degenerative changes following anterior cervical discectomy and fusion evaluated by fast spin-echo MR imaging. ACTA RADIOL.

[4] Matsunaga S, Kabayama S, Yamamoto T (1999). Strain on intervertebral discs after anterior cervical decompression and fusion. Spine (Phila Pa 1976).

[5] Hilibrand AS, Robbins M. Adjacent segment degeneration and adjacent segment disease: the consequences of spinal fusion? SPINE J. 2004, 4;(6 Suppl):190S–4S. 10.1016/j.spinee.2004.07.007 15541666

[6] Lund T, Oxland TR (2011). Adjacent level disk disease--is it really a fusion disease?. Orthop Clin North Am.

[7] Herkowitz HN, Kurz LT, Overholt DP (1990). Surgical management of cervical soft disc herniation. A comparison between the anterior and posterior approach. Spine (Phila Pa 1976).

[8] Song KJ, Choi BW, Jeon TS (2011). Adjacent segment degenerative disease: is it due to disease progression or a fusion-associated phenomenon? Comparison between segments adjacent to the fused and non-fused segments. Eur Spine J.

[9] Gore DR, Sepic SB (1984). Anterior cervical fusion for degenerated or protruded discs. A review of one hundred forty-six patients. Spine (Phila Pa 1976).

[10] Puttlitz CM, Rousseau MA, Xu Z (2004). Intervertebral disc replacement maintains cervical spine kinetics. Spine.

[11] Diangelo DJ, Foley KT, Morrow BR (2004). In vitro biomechanics of cervical disc arthroplasty with the ProDisc-C total disc implant. Neurosurg Focus.

[12] Wigfield C, Gill S, Nelson R (2002). Influence of an artificial cervical joint compared with fusion on adjacent-level motion in the treatment of degenerative cervical disc disease. J Neurosurg.

[13] Botelho RV, Moraes OJ, Fernandes GA (2010). A systematic review of randomized trials on the effect of cervical disc arthroplasty on reducing adjacent-level degeneration. Neurosurg Focus.

[14] Yang B, Li H, Zhang T (2012). The incidence of adjacent segment degeneration after cervical disc arthroplasty (CDA): a meta analysis of randomized controlled trials. PLoS One.

[15] Luo J, Gong M, Huang S (2015). Incidence of adjacent segment degeneration in cervical disc arthroplasty versus anterior cervical decompression and fusion meta-analysis of prospective studies. Arch Orthop Trauma Surg.

[16] Moher D, Cook DJ (2000). Eastwood S, et al. Improving the quality of reports of meta-analyses of randomized controlled trials: the QUOROM statement. Rev Esp Salud Publica.

[17] Jawahar A, Cavanaugh DA, Kerr ER (2010). Total disc arthroplasty does not affect the incidence of adjacent segment degeneration in cervical spine: results of 93 patients in three prospective randomized clinical trials. Spine J.

[18] Goffin J, Casey A, Kehr P (2002). Preliminary clinical experience with the Bryan cervical disc prosthesis. Neurosurgery.

[19] Higgins JP, Altman DG, Gotzsche PC (2011). The Cochrane Collaboration’s tool for assessing risk of bias in randomised trials. BMJ.

[20] Atkins D, De Briss PA, Eccles M, et al. Systems for grading the quality of evidence and the strength of recommendations II: pilot study of a new system. BMC Health Serv Res. 2005:5. 10.1186/1472-6963-5-25 PMC1084246 15788089

[21] Deeks JJ, Higgins JPT, Altman DG, Higgins JPT, Green S (2011). Analysing data and undertaking meta-analyses. Cochrane handbook for systematic reviews of interventions version 5.1.0 (updated march 2011).

[22] Sasso RC, Anderson PA, Riew KD (2011). Results of cervical arthroplasty compared with anterior discectomy and fusion: four-year clinical outcomes in a prospective, randomized controlled trial. Orthopedics.

[23] Coric D, Nunley PD, Guyer RD (2011). Prospective, randomized, multicenter study of cervical arthroplasty: 269 patients from the Kineflex|C artificial disc investigational device exemption study with a minimum 2-year follow-up: clinical article. J Neurosurg Spine..

[24] Nunley PD, Jawahar A, Kerr ER (2012). Factors affecting the incidence of symptomatic adjacent-level disease in cervical spine after total disc arthroplasty: 2- to 4-year follow-up of 3 prospective randomized trials. Spine (Phila Pa 1976).

[25] Tian W, Yan K, Han X (2013). Comparison of the mid-term follow-up results between Bryan cervical artificial disc replacement and anterior cervical decompression and fusion for cervical degenerative disc disease. Chinese Journal of Orthopedics.

[26] Guan T, Hu Z, Xiu L (2014). Effect of cervical disc arthroplasty and anterior cervical decompression and fusion on adjacent segment degeneration. Zhongguo Xiu Fu Chong Jian Wai Ke Za Zhi.

[27] Li Z, Yu S, Zhao Y (2014). Clinical and radiologic comparison of dynamic cervical implant arthroplasty versus anterior cervical discectomy and fusion for the treatment of cervical degenerative disc disease. J Clin Neurosci.

[28] Burkus JK, Traynelis VC, Haid RJ (2014). Clinical and radiographic analysis of an artificial cervical disc: 7-year follow-up from the prestige prospective randomized controlled clinical trial: clinical article. J Neurosurg Spine..

[29] Zhang HX, Shao YD, Chen Y (2014). A prospective, randomised, controlled multicentre study comparing cervical disc replacement with anterior cervical decompression and fusion. Int Orthop.

[30] Davis RJ, Nunley PD, Kim KD (2015). Two-level total disc replacement with Mobi-C cervical artificial disc versus anterior discectomy and fusion: a prospective, randomized, controlled multicenter clinical trial with 4-year follow-up results. J Neurosurg Spine.

[31] Phillips FM, Geisler FH, Gilder KM (2015). Long-term outcomes of the US FDA IDE prospective, randomized controlled clinical trial comparing PCM cervical disc arthroplasty with anterior cervical discectomy and fusion. Spine (Phila Pa 1976).

[32] Zoega B, Karrholm J, Lind B (1998). Plate fixation adds stability to two-level anterior fusion in the cervical spine: a randomized study using radiostereometry. Eur Spine J.

[33] Katsuura A, Hukuda S, Saruhashi Y (2001). Kyphotic malalignment after anterior cervical fusion is one of the factors promoting the degenerative process in adjacent intervertebral levels. Eur Spine J.

[34] Auerbach JD, Anakwenze OA, Milby AH (2011). Segmental contribution toward total cervical range of motion: a comparison of cervical disc arthroplasty and fusion. Spine (Phila Pa 1976).

[35] Eck JC, Humphreys SC, Lim TH (2002). Biomechanical study on the effect of cervical spine fusion on adjacent-level intradiscal pressure and segmental motion. Spine (Phila Pa 1976).

[36] Takeshima T, Omokawa S, Takaoka T (2002). Sagittal alignment of cervical flexion and extension: lateral radiographic analysis. Spine Phila Pa 1976.

[37] Kelly MP, Mok JM, Frisch RF (2011). Adjacent segment motion after anterior cervical discectomy and fusion versus Prodisc-c cervical total disk arthroplasty: analysis from a randomized, controlled trial. Spine (Phila Pa 1976).

[38] Hauerberg J, Kosteljanetz M, Boge-Rasmussen T (2008). Anterior cervical discectomy with or without fusion with ray titanium cage: a prospective randomized clinical study. Spine (Phila Pa 1976).

[39] Mummaneni PV, Burkus JK, Haid RW (2007). Clinical and radiographic analysis of cervical disc arthroplasty compared with allograft fusion: a randomized controlled clinical trial. J Neurosurg Spine..

[40] Qureshi SA, Mcanany S, Goz V (2013). Cost-effectiveness analysis: comparing single-level cervical disc replacement and single-level anterior cervical discectomy and fusion: clinical article. J Neurosurg Spine..

[41] Ament JD, Yang Z, Nunley P (2014). Cost-effectiveness of cervical total disc replacement vs fusion for the treatment of 2-level symptomatic degenerative disc disease. JAMA Surg.

[42] Warren D, Andres T, Hoelscher C (2013). Cost-utility analysis modeling at 2-year follow-up for cervical disc arthroplasty versus anterior cervical discectomy and fusion: a single-center contribution to the randomized controlled trial. Int J Spine Surg.

