# Supplementary material for: Adjacent segment degeneration or disease after cervical total disc replacement: a meta-analysis of randomized controlled trials
Source: J Orthop Surg Res. 2018 Oct 3;13:244. doi: 10.1186/s13018-018-0940-9 (PMC6169069; doi:10.1186/s13018-018-0940-9)
Supplement: Supplementary file 1 — Table S1. Excluded studies and main reason for exclusion. (PDF 101 kb) [file 13018_2018_940_MOESM1_ESM.pdf]

S1 Table. Excluded studies and main reason for exclusion

Author	Excluded Study	Main Reason
Boselie TF (2015)	Cervical spine kinematics after anterior cervical discectomy with or without implantation of a mobile cervical disc prosthesis; an RCT.	Follow up < 24 months
Karabag H (2014)	Arthroplasty versus fusion for single-level cervical disc disease.	The number of participants < 30
Kang L (2013)	Artificial disk replacement combined with midlevel ACDF versus multilevel fusion for cervical disk disease involving 3 levels.	The number of participants < 30
Zhang X (2012)	Randomized, controlled, multicenter, clinical trial comparing BRYAN cervical disc arthroplasty with anterior cervical decompression and fusion in China.	Undesired result on ASD
Nabhan A (2011)	Assessment of adjacent-segment mobility after cervical disc replacement versus fusion: RCT with 1 year's results.	Follow up < 24 months
Kelly MP (2011)	Adjacent segment motion after anterior cervical discectomy and fusion versus Prodisc-c cervical total disk arthroplasty: analysis from a randomized, controlled trial.	Undesired result on ASD
Coric D (2010)	Prospective study of cervical arthroplasty in 98 patients involved in 1 of 3 separate investigational device exemption studies from a single investigational site with a minimum 2-year follow-up. Clinical article.	The same patients and data(Coric D 2011)
Burkus JK (2010)	Long-term clinical and radiographic outcomes of cervical disc replacement with the Prestige disc: results from a prospective randomized controlled clinical trial.	The same patients and data(Burkus JK 2014)
Park DK (2011)	Index and adjacent level kinematics after cervical disc replacement and anterior fusion: in vivo quantitative radiographic analysis.	Follow up < 24 months
Garrido BJ (2010)	Clinical outcomes of Bryan cervical disc arthroplasty a prospective, randomized, controlled, single site trial with 48-month follow-up.	The number of participants < 30
Anakwenze OA (2009)	Sagittal cervical alignment after cervical disc arthroplasty and anterior cervical discectomy and fusion: results of a prospective, randomized, controlled trial.	Undesired result on ASD
Anderson PA(2008)	Comparison of adverse events between the Bryan artificial cervical disc and anterior cervical arthrodesis.	The same patients and data(Sasso RC 2011)
Sasso RC (2008)	Cervical kinematics after fusion and bryan disc arthroplasty.	The number of participants < 30

Hauerberg J (2008)	Anterior cervical discectomy with or without fusion with ray titanium cage: a prospective randomized clinical study.	Undesired result on ASD
Sekhon LH (2007)	Magnetic resonance imaging clarity of the Bryan, Prodisc-C, Prestige LP, and PCM cervical arthroplasty devices.	The number of participants < 30
Mummaneni PV(2007)	Clinical and radiographic analysis of cervical disc arthroplasty compared with allograft fusion: a randomized controlled clinical trial.	The same patients and data(Burkus JK 2014)
Nabhan A (2007)	Disc replacement using Pro-Disc C versus fusion: a prospective randomised and controlled radiographic and clinical study.	Follow up < 24 months
Bartels RH(2006)	Design of the PROCON trial: a prospective, randomized multi-center study comparing cervical anterior discectomy without fusion, with fusion or with arthroplasty.	The number of participants < 30
Coric D(2006)	Prospective randomized controlled study of the Bryan Cervical Disc: early clinical results from a single investigational site.	The same patients and data(Coric D 2011)
Porchet F(2004)	Clinical outcomes with the Prestige II cervical disc: preliminary results from a prospective randomized clinical trial.	The number of participants < 30
