# Supplementary material for: Adjacent segment degeneration or disease after cervical total disc replacement: a meta-analysis of randomized controlled trials
Source: J Orthop Surg Res. 2018 Oct 3;13:244. doi: 10.1186/s13018-018-0940-9 (PMC6169069; doi:10.1186/s13018-018-0940-9)
Supplement: Supplementary file 2 — File S1. Original data of 11 included articles. (ZIP 12 mb) [file 13018_2018_940_MOESM2_ESM.zip › 11 included articles and original data referred in this article/11 included articles/17 Jawahar, A(US).pdf]

ELSEVIER

The Spine Journal 10 (2010) 1043–1048

THE SPINE JOURNAL

2010 Outstanding Paper: Surgical Science

Total disc arthroplasty does not affect the incidence of adjacent segment degeneration in cervical spine: results of 93 patients in three prospective randomized clinical trials

Ajay Jawahar, MD, MS*, David A. Cavanaugh, MD, Eubulus J. Kerr III, MD, Elisa M. Birdsong, BS, Pierce D. Nunley, MD

Spine Institute of Louisiana, 1500 Line Ave., Shreveport, LA 71101, USA

Received 14 January 2010; revised 22 June 2010; accepted 22 August 2010

Abstract

BACKGROUND CONTEXT: Advancements in the philosophy of ''motion preservation'' have led to the use of total disc arthroplasty (TDA) as an alternative to fusion for degenerative disc disease (DDD) in the cervical spine. A commonly proposed theory is that TDA could reduce the incidence of adjacent segment disease. All the published clinical studies for TDA discuss the ''equal efficacy'' results of different investigational device exemption (IDE) trials between TDA and anterior cervical discectomy and fusion (ACDF) but have not addressed the issue of adjacent segment disease.

PURPOSE: To present the comparison of outcome data with respect to clinical success rates, symptom-free period, and incidence of adjacent segment disease in 93 patients with one- and two-level cervical DDD treated with TDA or ACDF in three different Food and Drug Administration (FDA) investigational trials.

STUDY DESIGN: Prospective, randomized, FDA IDE trials.

PATIENT SAMPLE: Ninety-three patients with established symptomatic one-or two-level cervical disc disease who failed to respond to conservative treatment were randomized to receive TDA (59) or ACDF (34) as part of clinical trials involving three different artificial discs at our institution. Subjects were blind to the assigned group until after the surgery.

OUTCOME MEASURES: Visual analog pain score (VAS), Neck Disability Index, and cervical spine radiographs were collected at 6 weeks and at 3, 6, 12, 24, 36, and 48 months after surgery.

METHOD: Success of the index surgery was assessed based on outcome measures at the seven data points. Success was defined as reduction by more than 30 points in both VAS and Neck Disability Index, absence of neurological deficits, and no further intervention at the index level. Adjacent segment disease was established by radiology, neurophysiology, and subsequent interventions administered to the patients.

RESULTS: At median follow-up of 37 months (range, 24–49 months), 64 (25 ACDF and 39 TDA) patients satisfied the criteria for clinical success. Neck Disability Index was a better predictor of outcome than pain score ($p<.05$). Sixteen percent of TDA patients and 18% ACDF patients developed adjacent segment degeneration and were treated actively ($p=.3$). Concurrent lumbar DDD significantly increased the risk of adjacent segment degeneration ($p=.01$). Age, gender, smoking habits, and number of levels at index surgery had no predictive value.

CONCLUSION: Total disc arthroplasty is equivalent to ACDF for providing relief from symptoms in the treatment of one- and two-level DDD of cervical spine. The risk of developing adjacent segment degeneration is equivalent after both procedures but is significantly higher in patients with concurrent DDD in lumbar spine. © 2010 Elsevier Inc. All rights reserved.

Keywords: Cervical spine; DDD; Disc arthroplasty; ACDF; Adjacent segment degeneration

FDA device/drug status: not applicable.

Author disclosures: PDN (royalties, BioMet, Osprey Biomedical, LDR Spine; stock ownership, including options and warrants, Amedica, K2M, Paradigm Spine, Spineology; speaking/teaching arrangements, K2M, NuVasive; scientific advisory board, K2M, SpineMark, Spinal Motion, Vertebral Technologies).

* Corresponding author. Spine Institute of Louisiana, 1500 Line Ave., Shreveport, LA 71101, USA. Tel.: (318) 629-5555; fax: (318) 629-5432.

E-mail address: ajawahar@louisianaspine.org (A. Jawahar)

1529-9430/$ - see front matter © 2010 Elsevier Inc. All rights reserved.
doi:10.1016/j.spinee.2010.08.014

EVIDENCE & METHODS

Context
Cervical total disc arthroplasty is being used as an alternative to fusion for degenerative conditions, and one promoted advantage for this strategy is to avoid clinically important, adjacent segment degeneration over time.

Contribution
The authors reviewed one center's experiences with 93 patients enrolled in three randomized trials comparing cervical disc replacement with discectomy and fusion. They found that, at a median of 3 years postsurgery, clinical outcomes and adjacent-level degeneration were similar between TDA and fusion groups. The observation of symptomatic adjacent-level disease was best predicted by concomitant lumbar disease, suggesting a strong native predisposition to symptomatic degeneration.

Implication
Whether replacement arthoplasty in the spine will achieve its primary patient-centered goals—improved outcomes and less adjacent segment degeneration—remains an open question. Longer-term follow-up will be needed to answer these questions and to determine whether late device failure will become a major problem. In the absence of long-term data, relative predictability, safety, and costs should be significant considerations in the decision-making process.

—The Editors

Introduction

Since its introduction by Caspar et al. [1], anterior cervical discectomy and fusion (ACDF) has been accepted and performed as the surgical procedure of choice to manage intractable symptoms resulting from degenerative disc disease (DDD) of the cervical spine. Several published series in the past have reported satisfactory clinical and radiological outcomes of the procedure in providing short- and long-term symptomatic relief and restoring neurological functions in adult patients regardless of age and gender [2,3]. Although the reputation of ACDF as a successful procedure remains unchallenged, several studies in the late 1990s raised the possibility and provided evidence that the procedure may increase the stress on the nonoperated adjacent cervical segments, thus adversely impacting the incidence of degeneration at these adjacent levels [4–6]. In the early years of the 21st century, therefore, innovation was directed toward devising implants and procedures that would have the ability to restore and maintain the motion, segmental anatomy, and function while successfully providing symptomatic relief to the patients. The concept of "motion preservation technology" was thus born in the field of spine surgery and subsequently led to the development of cervical total disc arthroplasty (TDA) [7,8]. Since then, several TDA implants have been developed and used for treating cervical DDD, and the clinical outcome results have been published in the literature [9,10]. Although all these studies have provided undisputed clinical and radiological evidence of motion preservation after TDA and equivalence of efficacy of cervical TDA procedure to provide symptomatic relief and good clinical outcomes in patients, none of these studies have attempted to address the issue of adjacent segment degeneration. To the best of our knowledge, no published data provide the incidence of documented adjacent segment degeneration after cervical TDA nor assess the actuarial adjacent segment disease-free survival rates in the patients receiving TDA for cervical DDD. The purpose of the present work was to discuss the comparison and outcome analysis with respect to clinical success rates, symptom-free period, and incidence of adjacent segment disease in 93 patients with one- and two-level cervical DDD treated with TDA or ACDF as a part of three different Food and Drug Administration (FDA) investigational, prospective, randomized clinical trials.

Methods

Study design

Since December 2005, our institution has participated as an investigational site for USFDA investigational device exemption (IDE) clinical trials to assess the clinical safety and efficacy of three different artificial cervical discs. All three trials were prospective randomized trials with participating subjects being blinded to the assigned treatment until after the surgical procedures. All trials were conducted under the oversight of the Western Institutional Review Board (Olympia, WA, USA) with strict adherence to the FDA guidelines of Good Clinical Practices.

Patient cohort

All patients who presented in our clinic with documented one- and/or two-level symptomatic cervical DDD and had failed at least 6 months of active conservative management were screened as potential participants for the

Table 1
Inclusion criteria for participants

Inclusion criteria for enrollment
Skeletally mature patients
Diagnosis of radiculopathy or myeloradiculopathy of the cervical spine in a specific nerve root distribution C3–C7
Neck and/or arm pain VAS at least 30 mm on a 100-mm scale
Neck Disability Index score ≥30 points
Unresponsive to conservative treatment for at least 6 months
Absence of any medical condition that would interfere with the proposed surgery
Subject provided informed consent and willing to comply with the protocol
No prior surgery at the index levels

TDA clinical trials. After informed consents were obtained, the patients were assessed for satisfaction of inclusion/exclusion criteria prerequisite for enrollment in the trials. These criteria were similar in all three trials and have been listed in Table 1. The cervical DDD was documented by clinical sign and symptoms, plain radiographs of the cervical spine, magnetic resonance imaging of the cervical spine, and electrophysiological studies. Active conservative treatment included medication (narcotics and nonnarcotics), physical therapy, chiropractic manipulations, and transforaminal epidural steroid injections.

Randomization scheme

The participating patients were assigned to receive TDA or ACDF by an off-site, unbiased, computer-generated randomization method. The patients remained blinded to the assigned treatment until after the surgery.

Devices and procedures

The three different TDA devices in the trial were Kineflex-C (SpinalMotion Inc., Mountain View, CA, USA), Mobi-C (LDR spine, Austin, TX, USA), and Advent Cervical Disc (Blackstone Inc., Parsippany, NJ, USA). All three devices had satisfied the FDA safety criteria for investigational clinical trials. The implanting surgeons were trained in the cadaver laboratory and certified by the manufacturers. Anterior cervical discectomy and fusion was performed using the modified Smith Robinson technique. Cortical bone allograft was used in all cases for fusion. The use of demineralized bone matrix or bone morphogenetic protein was not permitted in any of the protocols. Anterior plating was done in all procedures.

Outcome parameters

The clinical data collected were visual analog pain scores (VAS) from the patient, which were expressed in millimeters on a scale of 0–100; Neck Disability Index (NDI) assessed as per the patient's response; and quality-of-life data collected by Short Form-12 health survey. In addition, patients were subjected to a complete neurological examination by a qualified clinician. Radiological data included plain radiographs of cervical spine in six views (anteroposterior view in neutral, right, and left bending; lateral in neutral, flexion, and extension positions). Data at the baseline were collected at the time of enrollment. The patients were followed in the clinic at 6 weeks, 3 and 6 months, and then annually up to 4 years after their surgery. The clinical and radiological data were collected at every visit. Success of the index surgery was defined as reduction by more than 30 points in both VAS and NDI scores from baseline; absence of worsening or new neurological deficits, and no subsequent intervention at the index levels. All the criteria had to be satisfied for documenting clinical success. Additionally, patients developing new complaints pertaining to cervical spine were worked up for possible adjacent segment disease with repeat magnetic resonance imaging of the cervical spine and electrophysiological studies. The radiological criteria considered for diagnosing adjacent segment disease were the same as recommended by Hillibrand et al. [5] in their positional article. Additionally, to rule out confounding bias, only those patients who demonstrated clinical and radiological stigmata of adjacent segment disease and received active intervention for its management were included in the statistical analysis.

Table 2
The demographic characteristics of the participating patients

Characteristics	ACDF group, n (%)	TDA group, n (%)	p Value
Gender			
F	18 (52.9)	38 (64.4)	.13
M	16 (47.1)	21 (35.5)	
Smoking habits			
Smokers	11 (32.4)	29 (49.2)	.36
Nonsmokers	23 (67.6)	30 (50.8)	
Number of affected levels			
One level	28 (82.4)	43 (72.9)	.43
Two level	6 (17.6)	16 (27.1)	
Bone density			
Osteopenia	5 (14.7)	14 (23.7)	.77
Normal density	29 (85.3)	45 (76.3)	
Lumbar DDD			
Yes	9 (26.5)	17 (28.8)	.87
No	25 (73.5)	42 (71.2)	
Mean baseline scores			
VAS	76	80	.9
NDI	60	61	
Neurological deficit			
Present	13 (38.3)	24 (40.7)	.9
Absent	21 (61.8)	35 (59.3)	

ACDF, anterior cervical discectomy and fusion; TDA, total disc arthroplasty; F, female; M, male; DDD, degenerative disc disease; VAS, visual analog pain score; NDI, Neck Disability Index.

Statistics

All data management and statistical analysis were performed using SPSS version 15.0 (SPSS Inc., Chicago, IL, USA). Single-group comparisons were performed to measure intrapatient variance and differences on all clinical parameters. Descriptive frequencies and percentages were tabulated. The factors affecting the outcome were adjusted and weighted for their effect or influence. Paired sample t test was used to correlate clinical outcomes parameters (VAS and NDI) and general linear model analysis of variance with repeated measures was used to detect outcome differences between the two groups for TDA and ACDF. Statistically significant difference between comparative groups was considered at the 95% confidence interval ($p \leq .05$). Kaplan–Meier survival curves were plotted for patients in the two groups to predict the actuarial symptom-free periods and adjacent-level disease-free survival periods and study the effect of various factors that could possibly affect these survival rates.

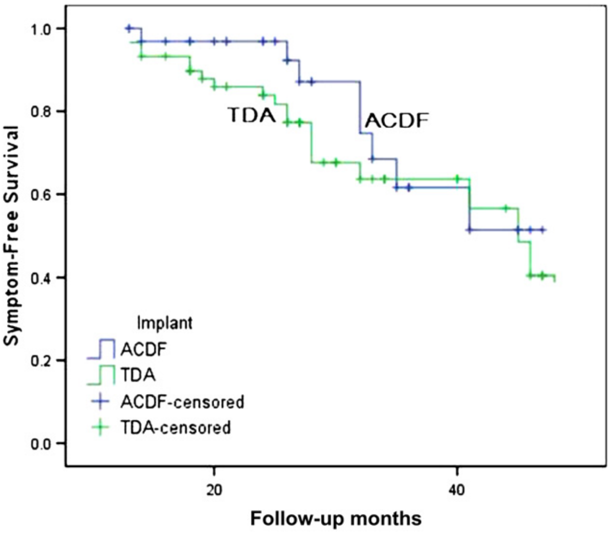

Fig. 1. Kaplan–Meier graphs freedom from symptoms in both groups. ACDF, anterior cervical discectomy and fusion; TDA, total disc arthroplasty.

Results

The demographic characteristics of the participating patients are noted in Table 2. There were no statistically significant differences between the distribution of these characteristics within the groups of ACDF and TDA. Although the protocols allowed exclusion of osteoporotic patients from participation (bone density ‘‘T’’ scores for spine <-2.5), they did not exclude the patients with osteopenia (bone density ‘‘T’’ scores for spine between -2.4 and -1.5). These 19 patients were also equally randomized in the two treatment groups.

Disease-free survival

The follow-up period ranged from 24 to 49 months (median, 36.4 months). At the last follow-up, 65 patients (69.8%) were free from symptoms and satisfied the criteria for success. The success rates for TDA (71%) were not statistically different from those of ACDF (73.5%). The actuarial median symptom-free survival period was 39.79±1.9 months for ACDF and 38.09±1.9 months for TDA patients. Fig. 1 depicts the Kaplan–Meier graphs for freedom from symptoms for both groups. The predicted disease-free survival rates at 36 months after surgery were 68.5%±1.1% for ACDF and 67.6%±0.7% for TDA procedures. We analyzed the effect of patient age, gender, smoking habits, number of affected levels (one vs. two), and osteopenia on disease-free survival. Although none of the factors affected the outcome with statistical significance ($p>.05$), smokers showed marginally worse survival rates ($p=.17$) for both procedures.

Visual analog pain scores and NDI scores

The final scores for VAS and NDI were similar for both groups with no statistical difference ($p=.693$). The mean

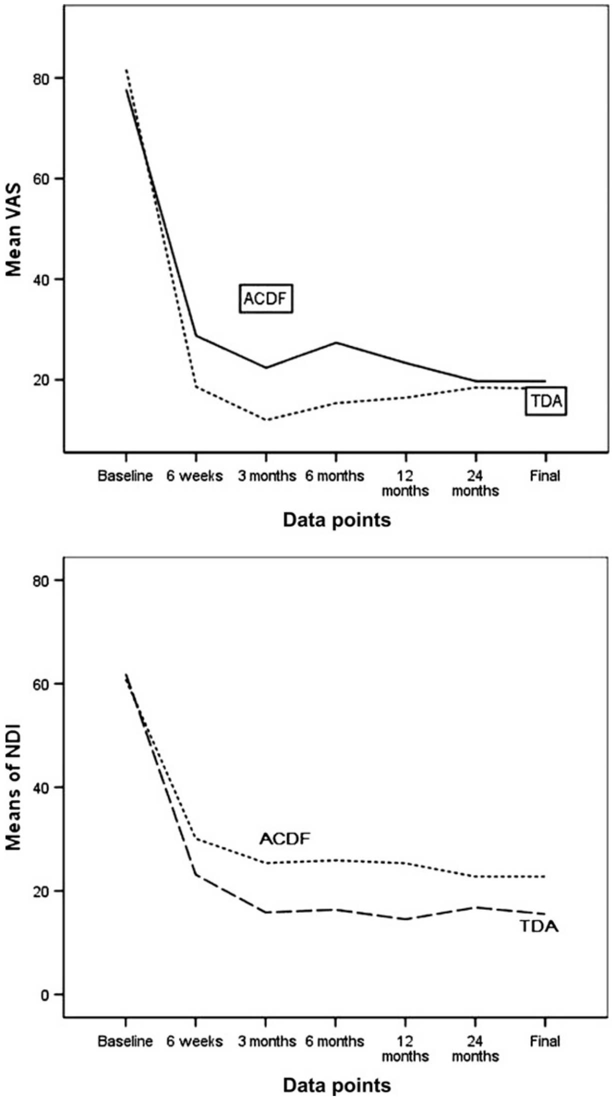

Fig. 2. (Top) Repeated-measures analysis of variance trends for VAS and (Bottom) NDI in patients of both groups. VAS, visual analog pain scores; NDI, Neck Disability Index; ACDF, anterior cervical discectomy and fusion; TDA, total disc arthroplasty.

improvement in NDI scores was 43±2.9 points for ACDF patients and 44.9±2.6 points for TDA patients. Similarly, mean improvement in VASs was 61.6±4.1 points for ACDF patients and 61.7±3.5 points for TDA patients. However, interesting trends were noted for these outcome scores with repeated-measures analysis of variance. Both scores showed a significantly better improvement at 6 weeks and 3 and 6 months after surgery in patients with TDA when compared with scores of those receiving ACDF ($p=.01$). These scores, however, tended to juxtapose for the two groups for longer follow-up at 1, 2, 3, and 4 years, respectively. These results showed that TDA definitely afforded a quicker recovery and improvement in symptoms when compared with ACDF in these patients. Fig. 2, Top and Bottom, graphically depicts the trends for these scores in the two groups. Another interesting observation was that for final outcome analysis,

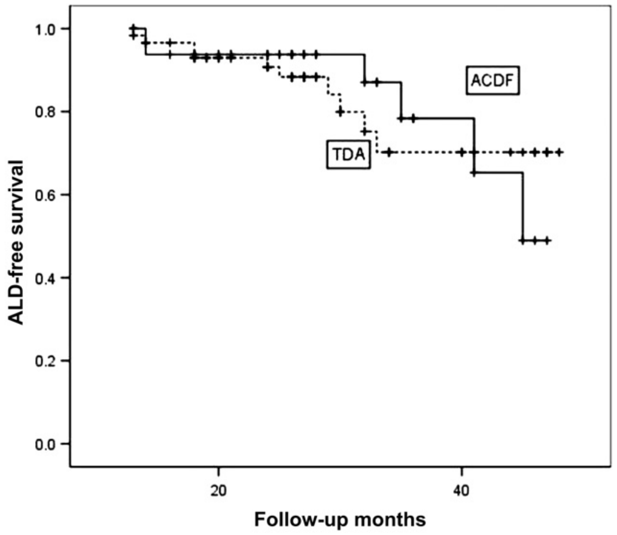

Fig. 3. Kaplan–Meier graphs depicting projected time of freedom from adjacent segment disease in both implant groups. NDI, Neck Disability Index; ACDF, anterior cervical discectomy and fusion; TDA, total disc arthroplasty.

absolute VASs tended to be higher than the NDI scores for the same data point. To check the validity of these parameters, we performed a paired sample *t* test that showed a lack of linear correlation between the VAS and NDI scores at the final follow-up data point. In conclusion, NDI scores rather than VASs were found to be better predictors of patient's clinical improvement.

Adjacent segment disease

The clinical and radiological parameters for establishing adjacent-level degeneration (ALD) in the participating patients were outlined earlier. Accordingly, 15% of ACDF patients and 18% of TDA patients were labeled as suffering from adjacent-level degenerative disease at the last follow-up. Hence, no statistical difference was noted for the incidence or risk of developing ALD in the two groups (p=.885). The mean period of freedom from adjacent-level disease was 37.8±4 months for ACDF group and 38.2±3.4 months for the TDA group; the actuarial survival rates for freedom from ALD at 36 months were 78.3%±1.0% and 84.1%±0.6% for the ACDF and TDA groups, respectively. Fig. 3 depicts the projected time of freedom from ALD in both implant groups. Again, we considered smoking habits, initial number of diseased levels (one vs. two), and presence of documented concurrent lumbar spine degeneration at the time of index surgery as possible risk factors for developing ALD. Table 3 highlights the calculated statistical significance of these risk factors for developing adjacent-level degenerative disease. Although smoking habit or number of initial levels treated did not affect ALD, presence of documented lumbar disease was a statistically significant risk factor for the development of adjacent segment degeneration in both groups (p=.016). In the presence of concurrent lumbar disease, the projected rates for freedom from ALD dropped down to 54.3%±1.9% for ACDF and 52.3%±1.5% for TDA patients. Fig. 4, Top and Bottom, depicts the Kaplan–Meier graphs for ALD-free survival periods as affected by the presence of concurrent lumbar spine disease for both groups.

Table 3
Statistical significance of these risk factors for developing adjacent level degenerative disease (log-rank tests)

Factors	Chi-square	Division factor	Significant p value
Concurrent lumbar DDD	1.959	1	.016
One vs. two levels	0.009	1	.926
Smokers vs. nonsmokers	0.156	1	.693

DDD, degenerative disc disease.

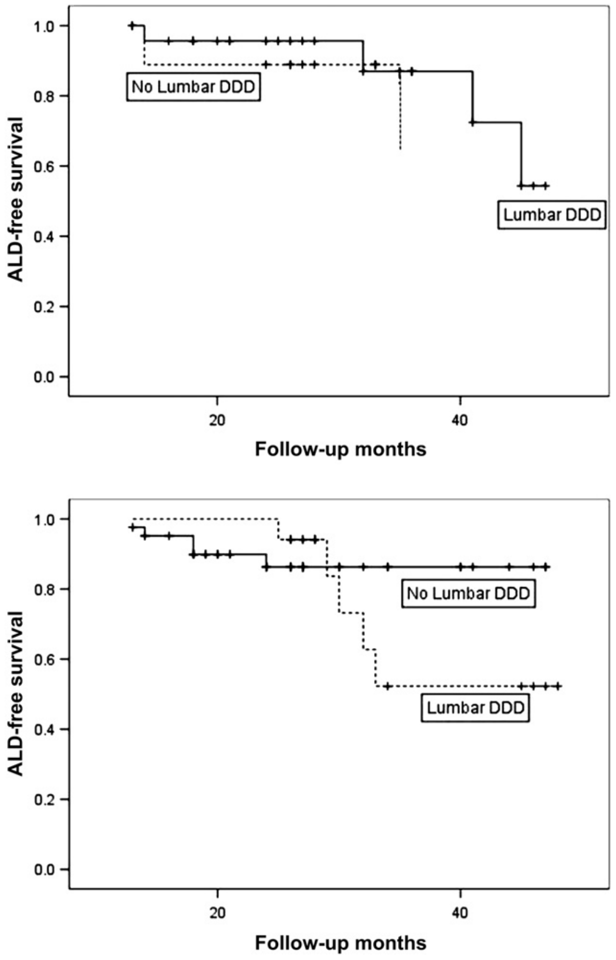

Fig. 4. (Top) Kaplan–Meier graphs for adjacent segment disease-free survival periods as affected by the presence of concurrent lumbar spine disease for ACDF and (Bottom) TDA patients. ALD, adjacent level degeneration; TDA, total disc arthroplasty; DDD, degenerative disc disease.

Discussion

As previously mentioned, several published clinical studies have supported the safety and efficacy of TDA in the treatment of symptomatic one- and two-level DDD of

the cervical spine. It has been shown that, for both short- and medium-term follow-up periods, TDA is as safe and effective as ACDF for providing symptomatic relief to the patients and improving neurological status [10–12]. However, the basis for arthroplasty was dependent on the altered mechanical forces being the key factor accelerating degenerative changes in the adjacent segments of the cervical spine. Cervical TDA was shown to reduce stresses in the adjacent annulus compared with motion segments stabilized by fusion [13]. Although no published study, to the best of our knowledge, has addressed the issue of ALD in patients with TDA, general consensus among spine surgeons has tended to support the theoretical assumption that TDA by virtue of motion preservation would reduce delay of the process of adjacent segment degeneration, thus reducing the risk of symptomatic adjacent-level disease.

The published results have focused on clinical outcome results for specific artificial discs tested in different European and USFDA clinical trials. The present work differs from all of these previous studies in the facts that the results include patients from three different device trials; the criteria for clinical success were devised more stringently for the purpose of statistical analysis and accurate predictions; attention was focused specifically on disease-free survival periods and rates for the index levels as well as adjacent levels; and potential predictors of outcome were statistically tested for their effect on failure of index surgery and development of adjacent segment degeneration. Consequently, our results are slightly different from the ones published earlier. The lower success rate (69%) at median 3-year follow-up for our study when compared with those published for both ACDF [14,15] and TDA [10,11] is easily attributed to the much more strict criteria for clinical success that we adopted for the present study and relatively longer period of follow-up considered. The longer follow-up, as previously noted, actually led to narrower difference between outcome scores in the two groups. Whereas the scores for TDA patients showed a slight rise during 2-, 3-, and 4-year follow-up data points, those for the ACDF patients showed a downward trend, thus effectively eliminating the bias that could be attributed to a short-term follow-up study. The longer follow-up also exposed the lack of linear correlation between the patient-reported pain scores and the actual NDIs. It was consistently noted that the patients tended to higher pain scores even in the presence of improving NDIs. This trend was particularly noticeable in patients with generalized or low back pain, although they were specifically counseled by the research personnel to mark the level of "neck and arm" pain only at every visit.

The strict criteria applied for establishing adjacent segment disease were again an attempt to eliminate any observer-related bias during analysis. Documentation by clinical examination, radiology, and electrodiagnostic studies corroborated by the fact that all patients went on to receive active intervention for symptoms' relief assured the exclusion of any questionable case in the statistical analysis.

Conclusions

Total disc arthroplasty demonstrates equivalence of safety and efficacy when compared with anterior cervical fusion in the management of symptomatic DDD of the cervical spine. Although TDA affords a significantly quicker symptomatic relief, the longer-term outcomes do not prove results superior to those with ACDF. The clinical evidence failed to corroborate the widely professed theory that TDA could potentially reduce the risk of developing adjacent segment disease in patients. The risk of developing symptomatic adjacent segment disease is considerably higher in patients with concurrent lumbar degenerative disease regardless of the surgery performed for cervical DDD.

References

[1] Caspar W, Barbier DD, Klara PM. Anterior cervical fusion and Caspar plate stabilization for cervical trauma. Neurosurgery 1989;25:491–502.

[2] Bohlman HH, Emery SE, Goodfellow DB, Jones PK. Robinson anterior cervical discectomy and arthrodesis for cervical radiculopathy. Long-term follow-up of one hundred and twenty-two patients. J Bone Joint Surg Am 1993;75:1298–307.

[3] Caspar W, Geisler FH, Pitzen T, Johnson TA. Anterior cervical plate stabilization in one and two level degenerative disease: overtreatment or benefit? J Spinal Disord 1998;11:1–11.

[4] Baba H, Furusawa N, Imura S, et al. Late radiographic findings after anterior cervical fusion for spondylotic myeloradiculopathy. Spine 1993;18:2167–73.

[5] Hillibrand AS, Carlson GD, Palumbo MA, et al. Radiculupathy and myelopathy at segments adjacent to the site of a previous anterior cervical arthrodesis. J Bone Joint Surg Am 1999;81:519–28.

[6] Hillibrand AS, Robbins M. Adjacent segment degeneration and adjacent segment disease: the consequences of spinal fusion? Spine J 2004;4:190S–4S.

[7] Bryan VE Jr. Cervical motion segment replacement. Eur Spine J 2002;11(Suppl 2):S92–7.

[8] Sekhon LH. Cervical arthroplasty in the management of spondylotic myelopathy. J Spinal Disord Tech 2003;16:307–13.

[9] Wigfield CC, Gill SS, Nelson RJ, et al. The new Frenchay artificial cervical joint: results from a two-year pilot study. Spine 2002;27:2446–52.

[10] Heller JG, Sasso RC, Papadopoulos SM, et al. Comparison of BRYAN cervical disc arthroplasty with anterior cervical decompression and fusion: clinical and radiographic results of a randomized, controlled, clinical trial. Spine 2009;34:101–7.

[11] Sasso RC, Smucker JD, Hacker RJ, Heller JG. Clinical outcomes of BRYAN cervical disc arthroplasty: a prospective, randomized, controlled, multicenter trial with 24-month follow-up. J Spinal Disord Tech 2007;20:481–91.

[12] Yang S, Wu X, Hu Y, et al. Early and intermediate follow-up results after treatment of degenerative disc disease with the Bryan cervical disc prosthesis: single- and multiple-level. Spine 2008;33:E371–7.

[13] Wigfield CC, Skrzypiec D, Jackowski A, Adams MA. Internal stress distribution in cervical intervertebral discs: the influence of an artificial cervical joint and simulated anterior interbody fusion. J Spinal Disord Tech 2003;16:441–9.

[14] Rengachary SS, Sanan A. Anterior stabilization of the cervical spine using locking plate and screw systems. In: Wilkins RH, Rengachary SS, eds. Neurosurgery, 2nd ed, vol 2. New York, NY: McGraw Hill, 1996:2983–6

[15] Wang JC, McDonough PW, Endow KK, et al. Increased fusion rates with cervical plating for two-level anterior cervical discectomy and fusion. Spine 2000;25:41–5.
