# Supplementary material for: Adjacent segment degeneration or disease after cervical total disc replacement: a meta-analysis of randomized controlled trials
Source: J Orthop Surg Res. 2018 Oct 3;13:244. doi: 10.1186/s13018-018-0940-9 (PMC6169069; doi:10.1186/s13018-018-0940-9)
Supplement: Supplementary file 2 — File S1. Original data of 11 included articles. (ZIP 12 mb) [file 13018_2018_940_MOESM2_ESM.zip › 11 included articles and original data referred in this article/11 included articles/22 Sasso, R C(US).pdf]

Copyright © 2011 by The Journal of Bone and Joint Surgery, Incorporated

Results of Cervical Arthroplasty Compared with Anterior Discectomy and Fusion: Four-Year Clinical Outcomes in a Prospective, Randomized Controlled Trial

Rick C. Sasso, MD, Paul A. Anderson, MD, K. Daniel Riew, MD, and John G. Heller, MD

Background: The published two-year results of the pivotal U.S. Food and Drug Administration investigational device exemption trial with the use of the Bryan cervical disc arthroplasty compared with anterior cervical discectomy with fusion for treating single-level degenerative cervical disc disease revealed a significantly superior overall success rate in the arthroplasty group. The purpose of this study was to evaluate the midterm safety and effectiveness of the Bryan disc as an alternative to arthrodesis following anterior cervical discectomy.

Methods: A prospective, multicenter randomized clinical trial was undertaken for the treatment of persistent radiculopathy or myelopathy due to single-level cervical disc herniations or spondylosis. Patients were randomized to treatment with either the Bryan disc (the arthroplasty group; 242 patients) or anterior cervical discectomy and fusion (the fusion group; 221 patients). Patients completed preoperative and postoperative self-assessment forms at specified intervals and had radiographs made preoperatively, at six weeks, and at three, six, twelve, twenty-four, and forty-eight months after surgery. The primary outcome measure was overall success, a composite variable of safety and efficacy measures. Numerous secondary measures were assessed. The follow-up data for up to twenty-four months have been previously published. We report in the present study the forty-eight-month data collected on 181 patients who received the Bryan disc and 138 patients who underwent anterior cervical discectomy and fusion.

Results: The study groups were demographically similar. Substantial reduction in Neck Disability Index scores occurred in both groups compared with preoperative values. The greater improvement in the Neck Disability Index score in the Bryan disc cohort persisted through the four-year follow-up period ($p < 0.001$). The four-year overall success rates were 85.1% and 72.5% for the arthroplasty and fusion groups, respectively ($p = 0.004$). The improvement in the arm pain score was substantial for both groups and significantly greater in the Bryan disc cohort ($p = 0.028$), and the neck pain scores showed persistently greater improvement in the Bryan disc group at forty-eight months of follow-up ($p = 0.001$). Short Form-36 physical component score improvement remained greater among the Bryan disc cohort ($p = 0.007$). The mean range of motion for the Bryan disc was 8.08° and 8.48° at twenty-four and forty-eight months, respectively. Total and serious adverse event rates were similar between the groups.

Conclusions: The forty-eight-month follow-up data in the present report showed consistent, sustained significantly superior outcomes for cervical spine arthroplasty compared with cervical spine fusion. The arthroplasty cohort continued to show significantly greater improvements in Neck Disability Index, neck pain score, arm pain score, and Short Form-36 physical component score, as well as the primary outcome measure, overall success, at forty-eight months following surgery.

Level of Evidence: Therapeutic Level I. See Instructions to Authors for a complete description of levels of evidence.

Disclosure: One or more of the authors received payments or services, either directly or indirectly (i.e., via his or her institution), from a third party in support of an aspect of this work. In addition, one or more of the authors, or his or her institution, has had a financial relationship, in the thirty-six months prior to submission of this work, with an entity in the biomedical arena that could be perceived to influence or have the potential to influence what is written in this work. Also, one or more of the authors has had another relationship, or has engaged in another activity, that could be perceived to influence or have the potential to influence what is written in this work. The complete **Disclosures of Potential Conflicts of Interest** submitted by authors are always provided with the online version of the article.

J Bone Joint Surg Am. 2011;93:1684-92 • http://dx.doi.org/10.2106/JBJS.J.00476

Anterior cervical discectomy and fusion is a common treatment for radiculopathy and myelopathy. The procedure is well tolerated and, in correctly selected patients, the results are highly successful[1-3]. Longer follow-up has revealed that up to 25% of patients may develop recurrent radicular symptoms from adjacent segment disc degeneration[4]. Furthermore, reoperations may be required to treat complications of fusion, such as nonunion and bone graft collapse or expulsion. In select patients, cervical arthroplasty is a potential substitute for cervical fusion after anterior neural decompression.

In the United States, a prospective, randomized controlled clinical trial was performed to evaluate the safety and effectiveness of the Bryan Cervical Disc (Medtronic Spinal and Biologics, Memphis, Tennessee) compared with fusion with allograft and plate stabilization. The indications for surgery were radiculopathy or myelopathy in patients with single-level cervical disc disease. The twenty-four-month results of this trial have been published[5]. The overall success and other patient-reported outcome measures indicated that treatment with the Bryan cervical disc arthroplasty achieved significantly superior results and allowed patients to return to work sooner. The current investigation was prompted by the relatively short follow-up period for the implanted motion-bearing device in the prior study. Further, to assess cost-effectiveness, knowledge of the durability of improvement in self-reported outcomes compared with preoperative states is essential. The purpose of this study was to evaluate clinical functional outcomes four years after either Bryan cervical disc arthroplasty or anterior cervical discectomy and fusion, with an emphasis on assessing any deterioration in outcomes over time.

Materials and Methods

Subjects and Study Design

The study design has been reported elsewhere[5]. Briefly, eligible patients were at least twenty-one years old and had radiculopathy or myelopathy from single-level cervical disc disease secondary to disc herniation or focal osteophytes that had not responded to at least six weeks of nonoperative management. All investigational sites had institutional review board approval, and all patients provided voluntary informed consent to participate in the study. Patients were randomly assigned in a 1:1 ratio to one of two treatment groups: arthroplasty with use of an artificial disc, the Bryan Cervical Disc (Medtronic Spinal and Biologics) (Fig. 1), or fusion with anterior cervical plate stabilization and bone allograft. The surgical procedures were performed at thirty investigational sites by sixty-five investigators and coinvestigator surgeons. The procedure in the fusion group was standardized by using both a commercially available allograft (Cornerstone; Medtronic Spinal and Biologics) and a single anterior cervical plating system (Atlantis; Medtronic Spinal and Biologics). The patients in the arthroplasty group were treated with a two-week postoperative course of a nonsteroidal anti-inflammatory drug of their surgeon's choice. Recommendations for immobilization with either soft or hard cervical collars, or the imposition of activity restrictions, were left to the discretion of the surgeon for both patient cohorts.

The initial study end point was twenty-four months for the investigational device exemption clinical trial. However, as a condition for approval of this device, the U.S. Food and Drug Administration (FDA) required the manufacturer to extend its follow-up of enrolled subjects to ten years after surgery. This required each investigative center to reapply for institutional review board approval. Furthermore, renewed informed consent was acquired from willing study participants. Patients were evaluated, according to protocol-defined intervals, preoperatively, at the time of surgery and discharge, at six weeks, and at three, six, twelve, twenty-four, and forty-eight months postoperatively.

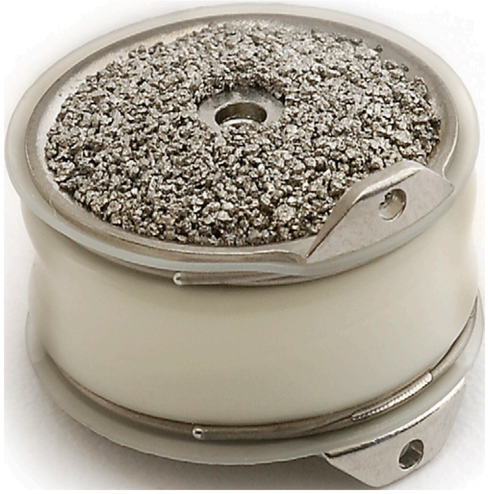

Fig. 1
The Bryan disc is composed of a polyurethane nucleus in a saline solution bath sandwiched between two titanium end plates wrapped by a polyurethane sleeve.

Outcomes Assessment

Pain and function were assessed with use of the Neck Disability Index (NDI)[6,7], the Short Form-36 (SF-36)[8], and numeric rating scales for neck and arm pain. Standardized neurological examinations, including motor and sensory function and reflexes, were recorded by the investigator or nursing staff. Neurological success was defined as maintenance or improvement of all three neurological parameters (motor and sensory function and reflexes). Radiographs were made preoperatively, prior to hospital discharge, and at three, six, twelve, twenty-four, and forty-eight months after surgery. All images were stored centrally and read by independent radiologists. All adverse events were recorded prospectively, categorized, evaluated for causality, and graded for severity with use of World Health Organization (WHO) criteria[9]. All were then reviewed for accuracy of categorization, causality, and severity by an independent physician.

The primary end point for the study was a composite measure termed overall success, consisting of the primary effectiveness and safety measures. To have the outcome be considered an overall success, patients had to achieve all of the following: an improvement of ≥15 points in the NDI, neurological improvement, no serious (WHO grade-3 or 4) adverse events related to the implant or surgical implantation procedure, and no subsequent surgery or intervention that would be classified as a treatment failure.

Patients were evaluated for flexion-extension motion of the cervical spine with use of the Cobb measurement technique on dynamic lateral radiographs of the cervical spine[10]. For each measurement, the means from two reviewers were calculated and used for analysis.

Statistical Methods

The primary analysis dataset consisted of all patients who received one of the study treatments. Statistical comparisons were primarily based on the observed and recorded follow-up data. A small number of patients required an additional surgical procedure (removal, revision, or supplemental fixation); their outcomes were recorded as a treatment failure for overall success—the primary study end point. For other outcome variables, the last-observation-carried-forward technique was used for all future evaluation periods.

To compare patients' demographic and preoperative measures, an analysis of variance was used for continuous variables and the Fisher exact test was used for categorical variables. For comparisons of postoperative mean

scores or mean score improvements measured in continuous scales, such as NDI scores, analysis of covariance was used with the preoperative score as the covariate. To assess the significance of improvement in the outcome measures within each treatment group, a paired t test was used. For comparison of success or event rates, the Fisher exact test was used to assess the superiority hypothesis.

One-sided p values were reported for most clinical outcomes as defined in the protocol except for surgery and return-to-work data, adverse events, and additional surgical procedures, which were two-sided. A p value of ≤0.05 was considered as significant.

Preoperative Comparison

From May 2002 to October 2004, a total of 463 enrolled subjects were randomly assigned to the study groups, with 242 assigned to the arthroplasty group and 221 to the fusion group. Two-year follow-up was achieved for 230 patients in the arthroplasty group and 194 in the fusion group whose data had been previously reported[5]. The current study involves the 181 arthroplasty and 138 fusion patients at the four-year follow-up (Fig. 2). Preoperative characteristics of the patients and preoperative clinical measures were similar in the two groups (see Appendix). There were no differences in demographics, disease severity (NDI and pain scores), and treated levels between the two groups preoperatively. Similarly, there were no differences between the treatment groups with regard to these preoperative variables for patients with complete forty-eight-month follow-up.

Source of Funding

Medtronic funded the investigational device exemption clinical trial and its continuation as a postapproval study. The clinical trials identification number is NCT00437190.

Results

Overall Success

At every time point postoperatively, the primary outcome measure of overall success was significantly superior for the arthroplasty group compared with the fusion cohort (Fig. 3). At the four-year postoperative mark, overall success was achieved in 85.1% of the patients in the arthroplasty group and

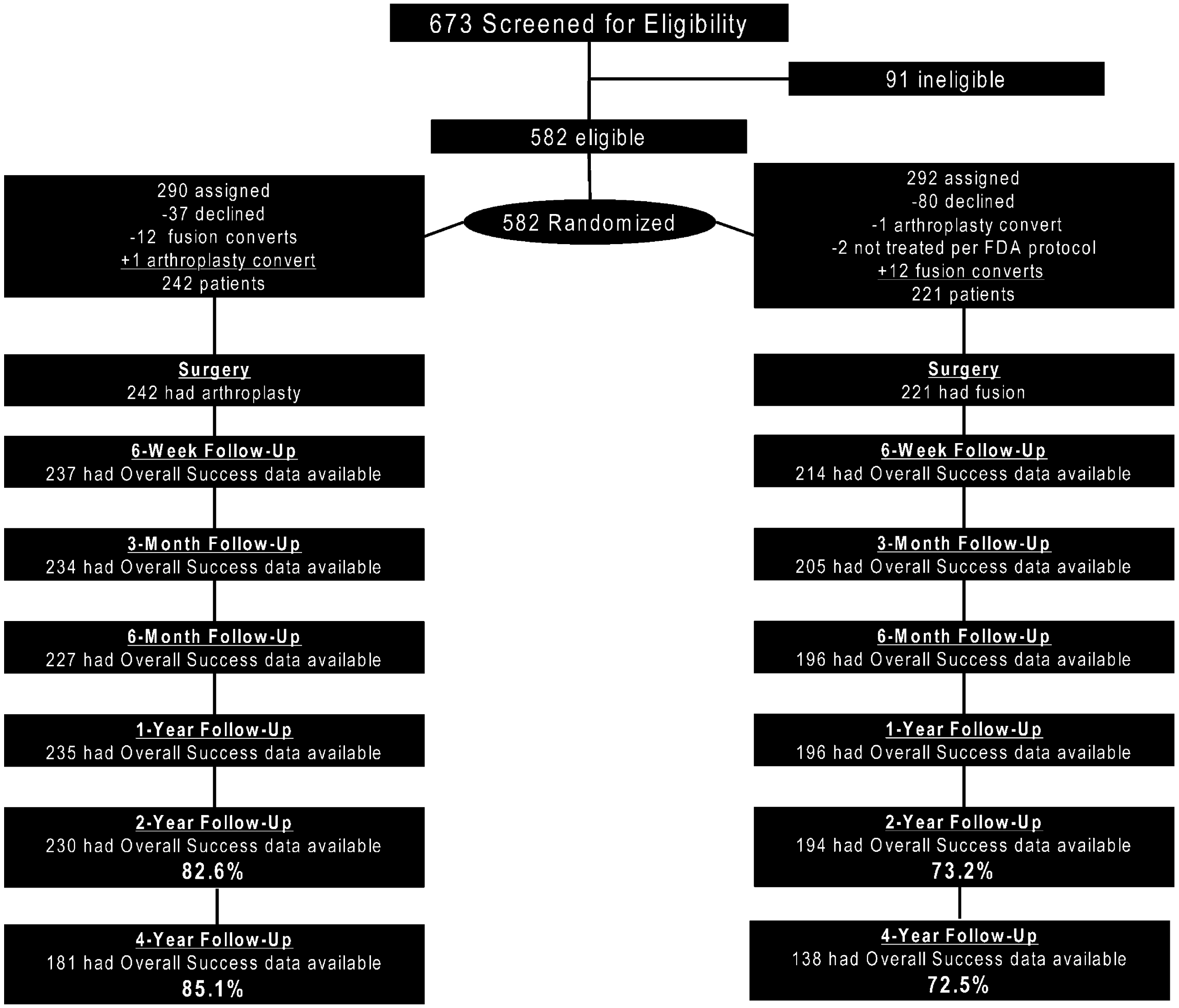

Fig. 2

Flow diagram of patient randomization and follow-up.

TABLE I Rates of Overall Success, Neck Disability Index Success, and Neurological Success at Forty-eight Months

Outcome	Arthroplasty Group*	Fusion Group*	P Value†
Overall success	154/181 (85.1)	100/138 (72.5)	0.004
Neck Disability Index success	164/181 (90.6)	109/138 (79.0)	0.003
Neurological success	167/180 (92.8)	124/138 (89.9)	0.234

*The values are given as the number with successful outcome/total number with available data, with the percentage in parentheses. †One-sided p values were from Fisher exact test.

72.5% in the fusion group (Table I) ($p = 0.004$). No deterioration over time was observed in either group.

Neck Disability Index

At every time point postoperatively, the two groups were significantly improved from their preoperative state and the arthroplasty group was significantly superior compared with the arthrodesis cohort (Fig. 4). At the four-year postoperative mark, the mean NDI was 13.2 (95% confidence interval [CI]: 10.9 to 15.6) in the arthroplasty group and 19.8 (95% CI: 16.5 to 23.2) in the arthrodesis group ($p < 0.001$). Improvement was seen rapidly within six weeks after surgery, and a plateau was reached by three months in the arthroplasty group and by six months in the fusion group.

Neck Disability Index Success

At every time point postoperatively, the percentage of the arthroplasty group who had a reduction of ≥15 points in NDI scores, a criterion for overall success, was significantly higher than that of the fusion group (Fig. 5). At the four-year postoperative mark, NDI success was achieved in 90.6% of the arthroplasty group and in 79.0% of the fusion group ($p = 0.003$).

Neurological Success

Neurological success rates at forty-eight months were similar to those observed at twenty-four months. The mean rates were 92.8% and 89.9% in the arthroplasty group and fusion group, respectively, and were not significantly different between the groups (Table I and Appendix).

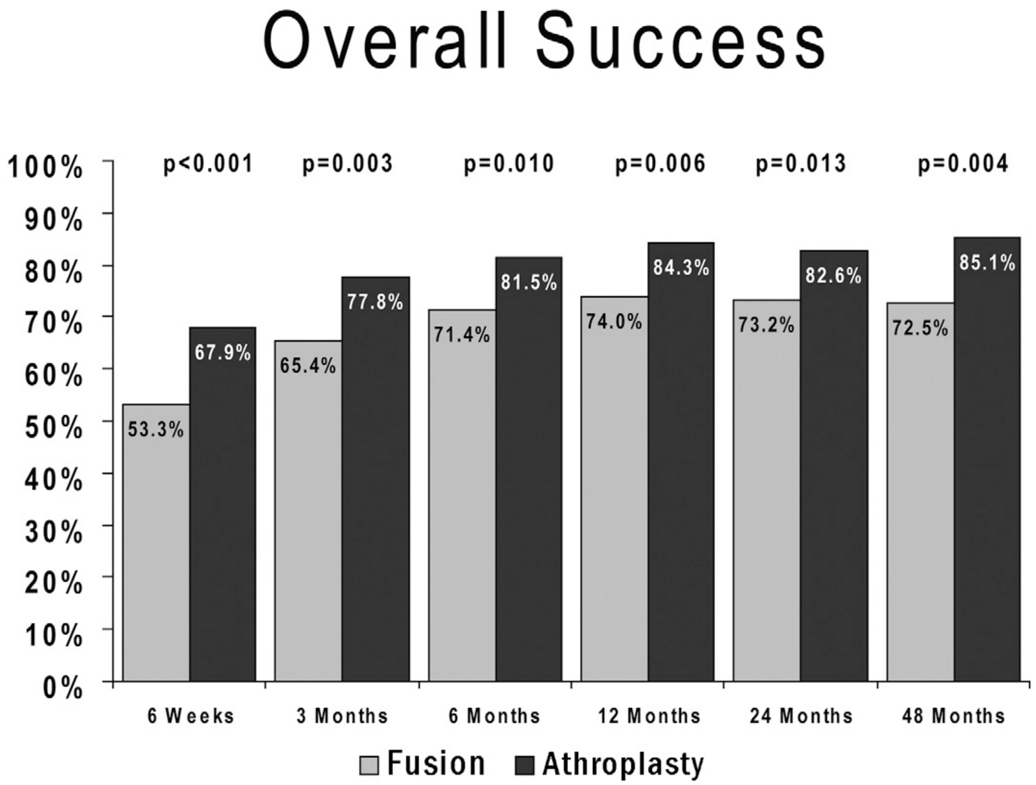

Fig. 3

Overall clinical success. The difference between the arthroplasty and fusion groups was significant at all time points.

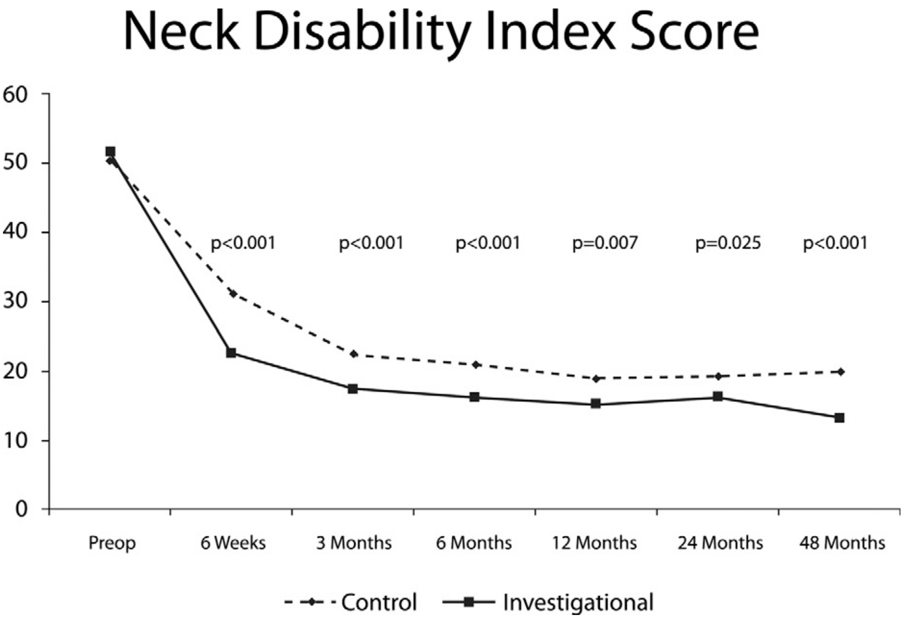

Fig. 4
Neck Disability Index (NDI) score. Significant improvement occurred from baseline in both groups. Furthermore, the difference in improvement between groups was significant at all time points. The results were durable for both groups and did not show significant change over time after three months.

Arm Pain
The score for arm pain improved rapidly from a mean preoperative score of 71.2 for both groups to 16.6 (95% CI: 13.1 to 20.2) and 22.4 (95% CI: 17.7 to 27.1) at forty-eight months of follow-up for the arthroplasty and fusion groups, respectively (p = 0.028). Small but significant differences in improvement of the arm pain score were detected between the groups at twelve and forty-eight months, favoring the arthroplasty group over the fusion group (see Appendix).

Neck Pain
The mean preoperative score for neck pain was 75.4 and 74.8 for the arthroplasty and fusion groups, respectively, while at forty-eight months, the mean neck pain score decreased to 20.7

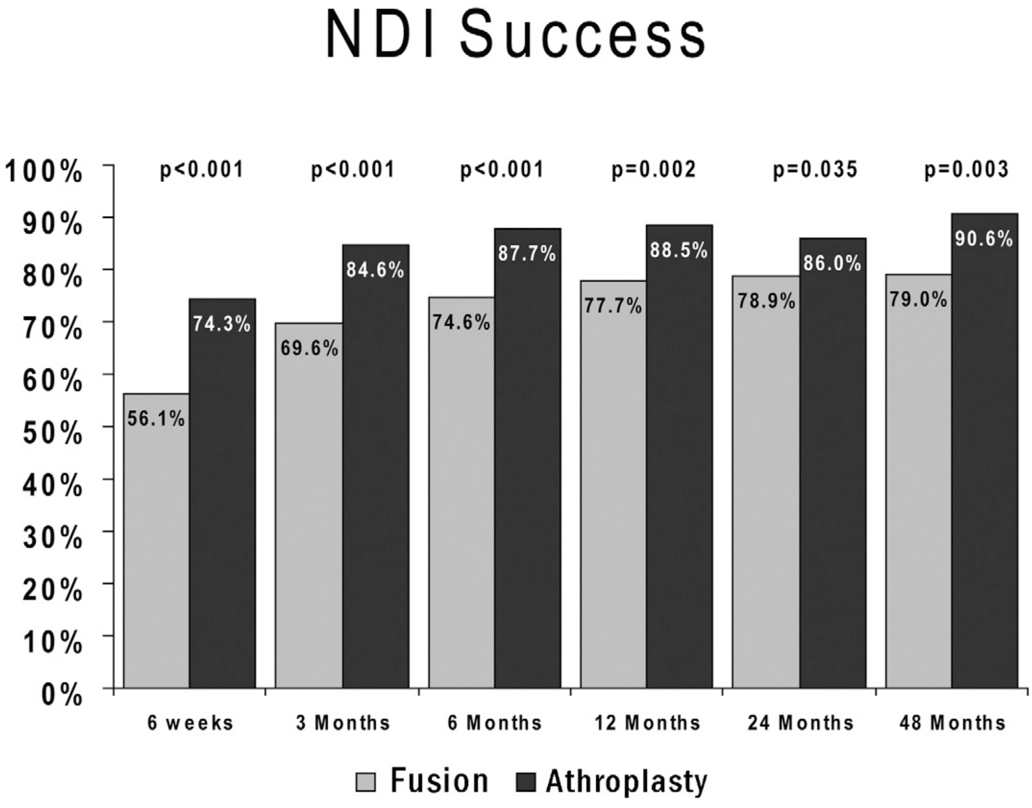

Fig. 5
Neck Disability Index (NDI) success is based on the achievement of a 15% improvement from baseline. Both groups had a high level of success (>75%) after three months, and no deterioration was seen over time. The arthroplasty group was significantly better than the fusion group at all time points.

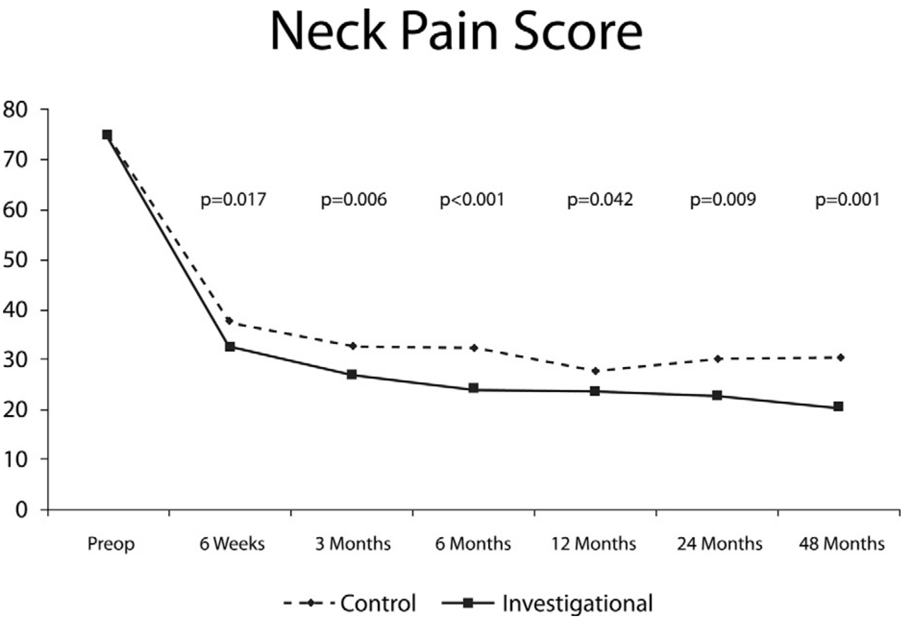

Fig. 6

The scores for neck pain on the visual analog scale. Improvement in the neck pain score for the arthroplasty group was significantly better than that in the fusion group at all time points.

(95% CI: 17.0 to 24.4) and 30.6 (95% CI: 25.5 to 35.8), respectively. Significant improvement in the neck pain score occurred by six weeks and was maintained at forty-eight months for both groups. The improvement was significantly greater in the arthroplasty group at all time points (Fig. 6).

SF-36 Summary Scores

Health-related quality of life was assessed by the SF-36 physical component and mental component summary scores. At forty-eight months, the mean postoperative SF-36 physical component and mental component scores had significantly improved

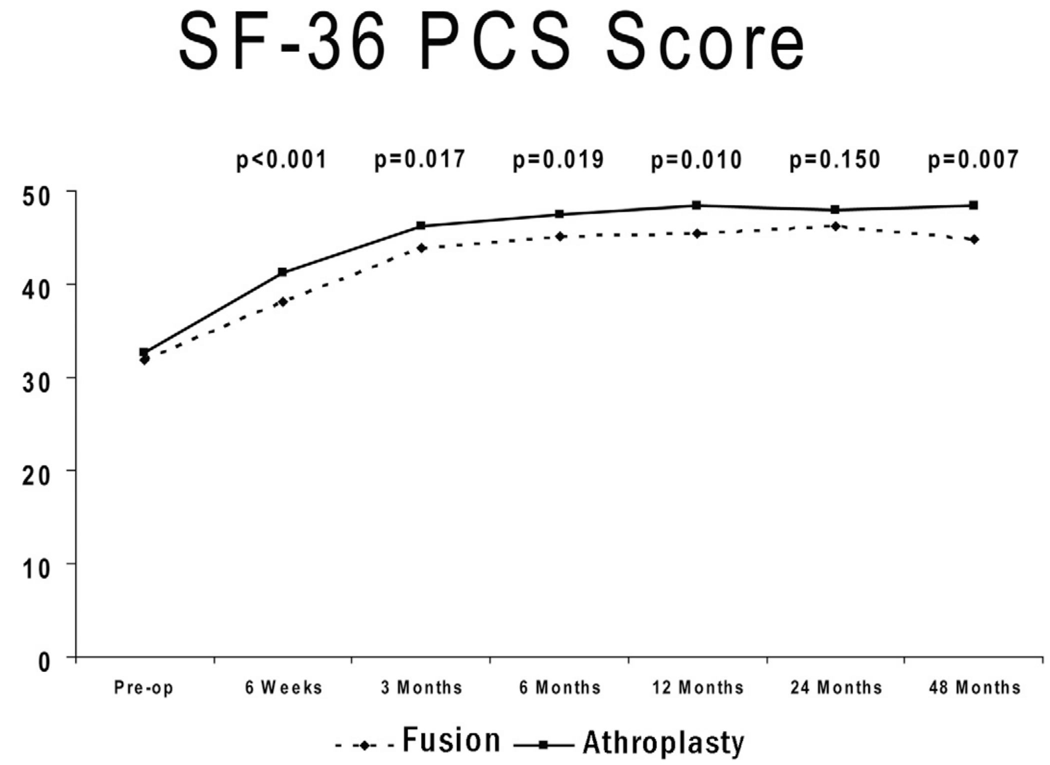

Fig. 7

SF-36 physical component summary (PCS) score. The physical functional outcome was significantly better in the arthroplasty group compared with the fusion group at forty-eight months postoperatively.

TABLE II Key Pain and Functional Scores Over Time

	Preoperative*		24 Months*			
	Arthroplasty (N = 242)	Fusion (N = 221)	Arthroplasty (N = 229)		Fusion(N = 194)	
	Score	Score	Score	Improvement	Score	Improvement
Neck Disability Index score	51.4 (15.3)	50.2 (15.9)	16.2 (18.5)	34.7 (20.5)	19.2 (19.3)	30.6 (19.8)
Arm pain score	71.2 (19.5)	71.2 (25.1)	19.1 (27.7)	52.3 (31.3)	21.5 (28.7)	50.3 (36.6)
Neck pain score	75.4 (19.9)	74.8 (23.0)	23.0 (27.7)	52.3 (29.9)	30.3 (39.7)	44.5 (41.3)
Short Form-36 physical component	32.6 (6.7)	31.8 (7.2)	47.9 (11.1)	15.1 (11.1)	46.3 (10.8)	14.5 (11.3)

*The values are given as the mean, with the standard deviation in parentheses.

for both treatment groups compared with preoperative levels. Furthermore, at forty-eight months, the mean SF-36 physical component score improvement was significantly better in the arthroplasty group compared with the fusion group ($p = 0.007$). The mean preoperative SF-36 physical component scores were 32.6 and 31.8 for the arthroplasty and fusion groups, respectively, increasing to 48.4 (95% CI: 46.8 to 49.9) and 44.9 (95% CI: 43.0 to 46.9) at forty-eight months (Fig. 7). The mean mental component score at the preoperative evaluation was 42.3 and 44.6 for the arthroplasty and fusion groups, respectively, increasing to 52.6 (95% CI: 51.1 to 54.0) and 51.9 (95% CI: 50.3 to 53.6) at forty-eight months.

The key pain and functional outcome scores at the preoperative evaluation and at two years and four years of follow-up are presented in Table II.

Return to Work

Preoperatively, 64.5% and 65% of the patients in the arthroplasty and fusion groups, respectively, were working. At six weeks after surgery, there were significantly more patients (49.2%) who had returned to work in the arthroplasty group than in the fusion group (39.4%). At forty-eight months, 74.7% and 67.9%, respectively, of the patients were working; the difference was not significant (see Appendix).

Range of Motion

The mean cervical spine motion in flexion-extension for the single-level arthroplasty group increased from 6.5° (95% CI: 6.0° to 6.9°) at baseline to 8.08° at twenty-four months and 8.5° (95% CI: 7.7° to 9.2°) at forty-eight months. This increase from baseline was significant at all time points after three months ($p < 0.05$). The fusion group showed a mean decrease of motion from 8.4° to 1.1° at forty-eight months.

Adverse Events

Adverse events that occurred up to two years postoperatively have been previously reported[5]. For this study, we report only the more severe WHO[9] grade-3 and 4 complications that occurred after twenty-four months and up to the forty-eight-month evaluation window. Forty-four patients in the arthroplasty

TABLE III Number of Patients Who Had Secondary Procedures and Interventions

	Up to 24 Months		24 to 48 Months		Total		
Second Procedures	Arthroplasty	Fusion	Arthroplasty	Fusion	Arthroplasty (N = 242)	Fusion (N = 221)	P Value*
Index level	6	8	3	2	9 (3.7%)	10 (4.5%)	0.816
Revisions	1	0	0	0	1	0	
Removals	3	3	1	1	4	4	
Supplemental fixations	0	4	0	1	0	5	
Reoperations	2	1	2	0	4	1	
External bone growth stimulators	0	2	0	0	0 (0.0%)	2 (0.9%)	0.227
Adjacent levels†	6	5	4	4	10 (4.1%)	9 (4.1%)	1.000
Other cervical levels	1	3	0	0	1 (0.4%)	3 (1.4%)	0.352

*Two-sided p values were from Fisher exact test. †Some secondary procedures were involved with both index and adjacent levels, and they were counted for index level only in this table.

TABLE II (*continued*)

48 Months*			
Arthroplasty (N = 181)		Fusion (N = 138)	
Score	Improvement	Score	Improvement
13.2 (16.1)	39.0 (19.1)	19.8 (20.0)	31.2 (21.3)
16.6 (24.4)	55.5 (27.5)	22.4 (28.2)	50.3 (35.9)
20.7 (25.3)	54.0 (29.3)	30.6 (30.8)	44.7 (33.6)
48.4 (10.6)	15.7 (11.1)	44.9 (11.7)	13.1 (12.0)

group had sixty-three adverse events classified as WHO grade 3 or 4 compared with thirty-six patients in the fusion group who had sixty-four adverse events; the difference was not significant. Most of these events were medical problems unrelated to the index surgery or the cervical spine. Severe episodes of neck and arm pain occurred in three and five patients in the arthroplasty and fusion groups, respectively. New neurologic deficits occurred in two patients in the fusion group.

Secondary Procedures

Cumulatively, up to the forty-eight-month evaluation window, nine patients (3.7%) in the arthroplasty group and ten (4.5%) in the fusion group had secondary surgical procedures involving the index cervical spine level (Table III); the difference is not significant. Secondary surgical procedures occurred in three patients in the arthroplasty group and two in the fusion group after twenty-four months and up to forty-eight months. Among them, one patient in each group had the device removed. The investigational device was explanted, and an arthrodesis performed, because of continued neck and shoulder pain. At adjacent levels, cumulatively up to the forty-eight-month evaluation window, the rates of secondary surgical procedures were the same (4.1%) in both treatment groups (Table III).

Discussion

A multicenter, prospective, randomized study with two-year follow-up of artificial cervical disc replacement compared with anterior cervical fusion for one-level degenerative disc disease showed significant differences between the groups[5]. For one-level cervical artificial disc replacement, improved functional outcomes were demonstrated for overall success, NDI, NDI success, and visual analog scale scores for neck pain. At four years postoperatively, we continued to see favorable outcomes for the artificial disc cohort, without any degradation of outcome measures between two and four years postoperatively. At forty-eight months, the arthroplasty cohort continued to show sustained, significantly superior outcomes, with a significantly higher rate of overall success and significantly greater improvements in NDI, neck pain scores, and SF-36 physical component scores. The improvement in arm pain scores was also significantly greater in the arthroplasty cohort at forty-eight months.

New technology such as disc arthroplasty requires long-term follow-up to assess durability, the biologic effects of wear, and the response of the prosthesis to its environment. Failure of other joint arthroplasty prostheses does not typically occur until at least five to ten years postoperatively. Spinal arthroplasties similarly need to have serial assessments to determine whether complications such as wear-related failures, device fatigue, or spinal instability have developed. The authors support the FDA requirement that the study sponsor be responsible for attempting to follow all willing study participants for up to eight years after the surgery.

Four years postoperatively, the arthroplasty prosthesis has proven quite durable, with few failures or explants and no change in neck motion over time. Few adverse events occurred in either group after twenty-four months. Spine or device-related events were primarily related to pain at the treated or adjacent disc levels. In the arthroplasty group, explantation occurred in one patient for continued pain. No arthroplasty device required removal for wear or wear-related failure. No new serious adverse event related to the device occurred. In the fusion group, reoperations occurred because of persistent pseudarthrosis and adjacent segment disc disease. Overall, the rates of reoperation are low in both the arthroplasty and fusion groups, with no significant difference detected.

The limitations of this four-year study mainly revolve around the relatively low rate of follow-up compared with the two-year study. At twenty-four months, 230 patients (95%) in the arthroplasty group were evaluated, while at forty-eight months, 181 (75%) were available. For the fusion group, 38% of the 221 enrolled patients failed to return for follow-up at forty-eight months. The lower rate may lead to attrition bias and affect the validity of our results. The actual results may therefore be at variance with the ones reported in this study.

This lower follow-up rate was caused by the original study design, which was set for only two years. A longer follow-up period was requested by FDA regulators; however, this required institutional review board approval at each treatment center and renewed consent and authorization from each patient. As a result, not all centers participated in the longer follow-up study and not all patients consented to the longer follow-up period. Additionally, even for the patients and sites who wished to participate in the longer follow-up period, logistical issues prevented many patients from completing the forty-eight-month follow-up. The follow-up rate was lower at forty-eight months because of the timing of FDA and institutional review board approvals. The forty-eight-month follow-up period was added to the protocol by an amendment. This required time to obtain FDA approval. After FDA approval, the sites had to submit to their institutional review boards, which often took much more time. During this time, the follow-up period for thirty-one patients had ended, and by the time approvals were obtained, the patients were in their sixtieth month of follow-up. Although it is a minor change in the wording of the initial study protocol, the stipulated period of allowed follow-up and the duration of informed consent should either be open-ended or at least be indicated as eight to ten years. This

would preempt the barriers with regard to institutional review boards and informed consent faced in this longer-term follow-up effort.

No deterioration of outcomes after anterior cervical plate stabilization and bone allograft was noticed at four years postoperatively. Clinical improvement, however, continued to be significantly better in the arthroplasty group compared with the fusion group in the primary outcome variables at the time of the four-year follow-up. An additional advantage to the arthroplasty group is that these benefits were obtained while preserving cervical spine motion. As for any motion-sparing device, however, longer-term follow-up is necessary for assessment of potential problems related to bearing surface wear.

Appendix

eA A table showing a summary of demographic data and figures showing data on neurological success, arm pain score, and the percentage of patients working are available with the online version of this article as a data supplement at jbjs.org. ■

Note: This study was possible only by the work of the following surgeons and research coordinators: **Surgeons:** Joseph Alexander, MD, Charles Branch, MD, Frederick Brown, MD, Joseph Cauthen, MD, Jeffrey Coe, MD, Domagoj Coric, MD, Richard Cunningham, MD, William Dobkin, MD, Scott Dull, MD, Richard Fessler, MD, Timothy Garvey, MD, Scott Gingold, MD, Robert Hacker, MD, Donald Johnson, MD, J. Patrick Johnson, MD, Mark Krinock, MD, Allen Levi, MD, James Lynch, MD, Patrick McCormick, MD, Luis Mignucci, MD, Paul Nottingham, MD, Glenn T. Pait, MD, Stephen Papadopoulos, MD, Daniel Resnick, MD, John Rhee, MD, K. Daniel Riew, MD, Richard Rovin, MD, Rick Sasso, MD, Michael Smith, MD, Matthew Songer, MD, Brian Sullivan, MD, Lee Thibodeau, MD, Donald Whiting, MD, Jeffrey Winfield, MD, and Seth Zeidman, MD. **Research Coordinators:** Heather Allerton, Anne Anderson, Lisa Armstrong, Rebecca Babcock, Terry Barker, Cheryl Black, Karen Blakely, Peggy Boltes, Helen Cambron, Diane Cantella, Gizelda Cassella, Mary Checovich, Michelle Cilento, Kelly Clinton, Wendy Cramer, Terry Crouse, Debbie Cushing, Tamra Davis, Jennifer Eclarina, Gina Falke, Peggy Fisher, Linda Foley, Shelly Garcia, Christopher Gilbert, Angela Gonzalez, Nancy Gugin, Carrie Gustafson, Amy Hanson, Nancy Holmes, Inge Howard, Pat Humphreys, Sonna Hunsley, Erin Hunt, Laila Ismail, Jennifer Jawahir, Brianna Johnson, Jolene Kjelshus, Selma Krone, Kimberly Levan, Lori Loftis, Debbie Love, Andrea Maser, Al Matus, Kimberly McCaughan, Brenda Miller, Charlotte Miller, Kathleen O'Brian, Mark Parent, Ruth Parks, Laura Parsons, Sandra Pritchett, Marc Pudlowski, George Rainey, Lisa Raw, Laurie Rice, Sherry Lea Rosenthal-Haag, Carole Rowell, Janice Murphy Schuller, Adam Simmons, Alta Skelton, Lucy Stephanian, Elizabeth Susskind, Jessica Sutton, Kim Vognet, Barb Warguleski, Allison Webb, Kathy Wharton, Kathy Wisnewski, and Julie Zazueta.

Rick C. Sasso, MD
Indiana Spine Group, 8402 Harcourt Road,
Suite 400, Indianapolis, IN 46260.
E-mail address: rsasso@indianaspinegroup.com

Paul A. Anderson, MD
Department of Orthopedic Surgery and Rehabilitation,
University of Wisconsin, 600 Highland Avenue K4/736,
Madison, WI 53792.
E-mail address: anderson@orthorehab.wisc.edu

K. Daniel Riew, MD
Washington University Orthopedics,
One Barnes-Jewish Hospital Plaza, Suite 11300,
St. Louis, MO 63110-1003.
E-mail address: riewd@msnotes.wustl.edu

John G. Heller, MD
Department of Orthopaedic Surgery,
Emory University School of Medicine,
59 Executive Park South, Atlanta, GA 30329.
E-mail address: john.heller@emoryhealthcare.org

References

1. Bohlman HH, Emery SE, Goodfellow DB, Jones PK. Robinson anterior cervical discectomy and arthrodesis for cervical radiculopathy. Long-term follow-up of one hundred and twenty-two patients. J Bone Joint Surg Am. 1993;75:1298-307.

2. Gore DR, Sepic SB. Anterior discectomy and fusion for painful cervical disc disease. A report of 50 patients with an average follow-up of 21 years. Spine (Phila Pa 1976). 1998;23:2047-51.

3. Garringer SM, Sasso RC. Safety of anterior cervical discectomy and fusion performed as outpatient surgery. J Spinal Disord Tech. 2010;23:439-43.

4. Hilibrand AS, Carlson GD, Palumbo MA, Jones PK, Bohlman HH. Radiculopathy and myelopathy at segments adjacent to the site of a previous anterior cervical arthrodesis. J Bone Joint Surg Am. 1999;81:519-28.

5. Heller JG, Sasso RC, Papadopoulos SM, Anderson PA, Fessler RG, Hacker RJ, Coric D, Cauthen JC, Riew DK. Comparison of BRYAN cervical disc arthroplasty with anterior cervical decompression and fusion: clinical and radiographic results of a randomized, controlled, clinical trial. Spine (Phila Pa 1976). 2009;34:101-7.

6. Vernon H, Mior S. The Neck Disability Index: a study of reliability and validity. J Manipulative Physiol Ther. 1991;14:409-15.

7. Westaway MD, Stratford PW, Binkley JM. The patient-specific functional scale: validation of its use in persons with neck dysfunction. J Orthop Sports Phys Ther. 1998;27:331-8.

8. McHorney CA, Ware JE Jr, Lu JF, Sherbourne CD. The MOS 36-item Short-Form Health Survey (SF-36): III. Tests of data quality, scaling assumptions, and reliability across diverse patient groups. Med Care. 1994;32:40-66.

9. World Health Organization. Toxic effects. WHO Handbook for reporting results of cancer treatment. No. 48. WHO offset publication. Geneva, Switzerland: World Health Organization; 1979. p 14-22.

10. Sasso RC, Best NM, Metcalf NH, Anderson PA. Motion analysis of Bryan cervical disc arthroplasty versus anterior discectomy and fusion: results from a prospective, randomized, multicenter, clinical trial. J Spinal Disord Tech. 2008;21:393-9.
