# Supplementary material for: Adjacent segment degeneration or disease after cervical total disc replacement: a meta-analysis of randomized controlled trials
Source: J Orthop Surg Res. 2018 Oct 3;13:244. doi: 10.1186/s13018-018-0940-9 (PMC6169069; doi:10.1186/s13018-018-0940-9)
Supplement: Supplementary file 2 — File S1. Original data of 11 included articles. (ZIP 12 mb) [file 13018_2018_940_MOESM2_ESM.zip › 11 included articles and original data referred in this article/11 included articles/23 Coric, D(US).pdf]

This article has been updated from its originally published version to correct an error in the *Disclosure*.

See the corresponding erratum notice, **DOI:** 10.3171/2011.11.SPINE10769a, for full details.

See the corresponding editorial in this issue, pp 345–347.

J Neurosurg Spine 15:348–358, 2011

Prospective, randomized, multicenter study of cervical arthroplasty: 269 patients from the Kineflex|C artificial disc investigational device exemption study with a minimum 2-year follow-up

Clinical article

Domagoj Coric, M.D.,[1] Pierce D. Nunley, M.D.,[2] Richard D. Guyer, M.D.,[3] David Musante, M.D.,[4] Cameron N. Carmody, M.D.,[5] Charles R. Gordon, M.D.,[6] Carl Lauryssen, M.D.,[7] Donna D. Ohnmeiss, Dr.Med.,[3] and Margaret O. Boltes, B.S.N.[1]

[1]Carolina Neurosurgery and Spine Associates, Charlotte; [4]Triangle Orthopedics Associates, Durham, North Carolina; [2]Spine Institute of Louisiana, Shreveport, Louisiana; [3]Texas Back Institute; [5]Plano Orthopedic Sports Medicine & Spine Center, Plano; [6]Gordon Spine Associates, Tyler, Texas; and [7]Olympia Medical Center, Los Angeles, California

Object. Cervical total disc replacement (CTDR) represents a relatively novel procedure intended to address some of the shortcomings associated with anterior cervical discectomy and fusion (ACDF) by preserving motion at the treated level. This prospective, randomized, multicenter study evaluates the safety and efficacy of a new metal-on-metal CTDR implant (Kineflex|C) by comparing it with ACDF in the treatment of single-level spondylosis with radiculopathy.

Methods. The study was a prospective, randomized US FDA Investigational Device Exemption (IDE) pivotal trial conducted at 21 centers across the US. The primary clinical outcome measures included the Neck Disability Index (NDI), visual analog scale (VAS) scores, and a composite measure of clinical success. Patients were randomized to CTDR using the Kineflex|C (SpinalMotion, Inc.) cervical artificial disc or ACDF using structural allograft and an anterior plate.

Results. A total of 269 patients were enrolled and randomly assigned to either CTDR (136 patients) or to ACDF (133 patients). There were no significant differences between the CTDR and ACDF groups when comparing operative time, blood loss, length of hospital stay, or the reoperation rate at the index level. The overall success rate was significantly greater in the CTDR group (85%) compared with the ACDF group (71%) ($p = 0.05$). In both groups, the mean NDI scores improved significantly by 6 weeks after surgery and remained significantly improved throughout the 24-month follow-up ($p < 0.0001$). Similarly, the VAS pain scores improved significantly by 6 weeks and remained significantly improved through the 24-month follow-up ($p < 0.0001$). The range of motion (ROM) in the CTDR group decreased at 3 months but was significantly greater than the preoperative mean at 12- and 24-month follow-up. The ROM in the ACDF group was significantly reduced by 3 months and remained so throughout the follow-up. Adjacent-level degeneration was also evaluated in both groups from preoperatively to 2-year follow-up and was classified as none, mild, moderate, or severe. Preoperatively, there were no significant differences between groups when evaluating the different levels of adjacent-level degeneration. At the 2-year follow-up, there were significantly more patients in the ACDF group with severe adjacent-level radiographic changes ($p < 0.0001$). However, there were no significant differences between groups in adjacent-level reoperation rate (7.6% for the Kineflex|C group and 6.1% for the ACDF group).

Conclusions. Cervical total disc replacement allows for neural decompression and clinical results comparable to ACDF. Kineflex|C was associated with a significantly greater overall success rate than fusion while maintaining motion at the index level. Furthermore, there were significantly fewer Kineflex|C patients showing severe adjacent-level radiographic changes at the 2-year follow-up. These results from a prospective, randomized study support that Kineflex|C CTDR is a viable alternative to ACDF in select patients with cervical radiculopathy.
(DOI: 10.3171/2011.5.SPINE10769)

Key Words • **cervical arthroplasty** • **anterior cervical discectomy and fusion** • **Kineflex|C Disc System** • **artificial disc** • **degenerative**

Cervical total disc replacement (CTDR) is a relatively novel technology that has moved beyond the investigational stage.[11,13,21,26,33–35] The era of cervical arthroplasty began in Europe in the late 1990s.[11,14,22–24] There have been several thousand patients enrolled in prospective, randomized FDA-regulated investigational

Abbreviations used in this paper: ACDF = anterior cervical discectomy and fusion; AP = anteroposterior; CTDR = cervical total disc replacement: IDE = investigational device exemption; NDI = neck disability index; ROM = range of motion; VAS = visual analog scale.

device exemption (IDE) trials in the US dating back to 2000.[26,34,35] Nine different CTDR devices have completed an FDA-regulated IDE pivotal study. Cervical arthroplasty offers several theoretical advantages over anterior cervical discectomy and fusion (ACDF) in the treatment of select patients with medically refractory cervical radiculopathy.[6,15,34] Anterior cervical discectomy and fusion results in loss of mobility at the operated level(s) and places increased stress on adjacent levels above and below the fusion, which may lead to a higher incidence of segmental degeneration.[3,17,28,29,31,32,39,42,48–52] There is a documented incidence of clinically symptomatic adjacent-level disc disease following ACDF ranging from 1.5% to 3% annually. [18,19,23,27] Hilibrand et al.[27] reported a rate of symptomatic adjacent-level degeneration following ACDF of 2.9% annually. Similarly, Robertson and associates[43] reported a symptomatic adjacent-segment degenerative disc disease rate of 7% in their anterior fusion series, which was significantly higher than their comparative CTDR series. Disc arthroplasty utilizes the familiar anterior cervical approach and achieves comparable neural decompression and clinical success to ACDF.[6,34] Furthermore, by preserving motion at the operated level, CTDR has the potential to decrease the occurrence of adjacent-segment degeneration.[9,16,41,43]

To establish CTDR as a viable therapeutic modality in the treatment of cervical radiculopathy, cervical artificial discs must show clinical efficacy and safety through the regulatory IDE process. This process produces prospective, randomized Level I data. Additionally, cervical arthroplasty must validate that the technique maintains segmental motion with concomitant decreased adjacent-level stresses compared with ACDF. The evidence-based rationale to adopt CTDR is further bolstered by the replication of individual clinical and radiographic success with several different devices. A total of 1213 patients were randomized in the clinical trials of the first 3 CTDR devices to receive regulatory approval.[26,34,35] After a technique is adopted, long-term data should be used to refine ideal indications for that procedure.[13]

We report the clinical and radiographic outcomes of 269 patients from a multicenter, prospective, randomized, controlled trial comparing Kineflex|C (SpinalMotion, Inc.), a metal-on-metal CTDR device, with ACDF with a minimum 2-year follow-up.

Methods

Patients with 1-level, symptomatic cervical disc disease with medically refractory radiculopathy and/or myelopathy were randomized prospectively (randomization 1:1) at 21 institutions as part of an FDA-regulated IDE pivotal trial to undergo ACDF with allograft and anterior plate or Kineflex|C artificial disc placement. All investigational centers used a standardized corticocancellous allograft (VG2, DePuy Spine) and anterior screw/plate (Slim-Loc, DePuy Spine) construct. Inclusion and exclusion criteria are listed in Table 1. Institutional review board approval was obtained at all 21 institutions, and all patients provided informed consent.

The patients were evaluated using pre- and postoperative serial neurological examinations and radiographs at 1.5, 3, 6, 12, and 24 months and completed clinical self-assessment indices at 12 and 24 months. Pain and function were assessed using the neck disability index (NDI) and a visual analog scale (VAS). These outcome measures were completed by the patient without assistance. The VAS was adapted to a 0–100 scale, and patients were instructed to consider neck, shoulder, and arm pain cumulatively. The neurological examination evaluated motor strength, sensory function, and reflexes.

Range of motion (ROM) was analyzed on dynamic radiographs by an independent core radiographic laboratory. In the ACDF group, fusion was defined using a composite measure consisting of 1) bridging trabecular bone; 2) angular motion less than 5°; 3) translational motion less than 3 mm; and 4) less than 50% radiolucency along the bone-implant interface. Also, a qualitative analysis of radiographic deterioration at the adjacent level above and below the index level was evaluated in both groups at all time points from preoperatively to the 24-month follow-up. The analysis of adjacent-segment degeneration was derived from a quantitative analysis of disc height and an independent radiologist's subjective assessments of osteophytes and endplate sclerosis based on a previously described qualitative and quantitative assessment of cervical disc degeneration.[47] The assessment of disc height, extent of osteophyte formation, and degree of endplate sclerosis is common to published degeneration grading systems.[47] Adjacent-level degeneration was classified as none, mild, moderate, or severe (Table 2). To document cases that clearly had adjacent-level disc degeneration, defined as a progression of disc degeneration at adjacent levels following treatment, only those cases that had at least a 2-grade increase in degeneration were classified as having adjacent-level deterioration. To account for cases that had moderate adjacent-level disc degeneration prior to treatment, only a 1-grade change was required to classify these cases as having adjacent-level deterioration.

Radiographs were evaluated by the core radiographic laboratory for evidence of migration, subsidence, and radiolucency of the devices in both groups. Device migration was assessed at each postoperative visit and was defined as greater than a 3-mm anteroposterior (AP) slip of the implant parallel to the vertebral endplates. Device subsidence was defined as bony penetration of the implant more than 3 mm into the superior and/or inferior endplates of the adjacent vertebral body.

Statistical Design/Statistical Analysis

The primary efficacy analysis was a noninferiority comparison of the Kineflex|C Disc to ACDF. The analysis was based on the 24-month results. The analysis was completed using the Blackwelder test. A delta value of 10.0% was used as the noninferiority margin. Two-sided 90% CI and 95% CI for the difference in response rates were shown as derived from the normal approximation to the binomial. The p value for the Blackwelder test for noninferiority also is presented for the noninferiority margin of 10.0% (0.1 in difference in proportions). For the observed success rate at each visit, a Fisher exact test was used to evaluate the null hypothesis that the success

TABLE 1: Inclusion and exclusion study criteria

Inclusion Criteria
btwn 18 & 60 yrs old
have symptoms of cervical degenerative disc disease/spondylosis at only 1 cervical level
level from C-3 to C-7 including ≥1 of the following:
degenerated/dark disc on MRI
disc height decreased by ≥1 mm when compared w/ adjacent levels on radiography, CT, or MRI
disc herniation on CT or MRI
have 1 of the following radiculopathy symptoms in neck, 1 or both shoulders, &/or 1 or both arms:
pain or paresthesias in a specific nerve root distribution from C-3 to C-7
decreased muscle strength of ≥1 level on the 0–5 scale
abnormal sensation, including hyper- or hypesthesia
have ≥1 of the following:
≥6 mos of prior conservative treatment (e.g., physical therapy &/or use of antiinflammatory medications & muscle relaxants)
presence of progressive symptoms (e.g., increasing numbness or tingling)
signs of nerve root compression
have an NDI ≥40 on a scale of 100 (moderate disability)
be appropriate for treatment using an anterior surgical approach
be likely to return for all follow-up visits
be willing & able to provide informed consent for study participation
Exclusion Criteria
marked cervical instability on resting lateral or flexion/extension radiograph (translation >3 mm or >11° rotation compared w/ adjacent levels)
nondiscogenic neck pain or nondiscogenic source of symptoms (e.g., tumor, rotator cuff injury, etc.)
radiographic confirmation of severe facet disease or facet degeneration
bridging osteophytes
<2° of motion at index level
prior surgery at the level to be treated, except laminotomy w/o accompanying facetotomy
prior fusion at any cervical level
≥1 neck surgery via anterior approach
previous trauma to the C3–7 levels resulting in compression or bursting
documented presence of a free nuclear fragment at cervical levels other than the study level
severe myelopathy (<3/5 muscle strength)
any paralysis
recent history (w/in previous 6 mos) of chemical or alcohol dependence
active systemic infection
infection at the site of surgery
prior disc space infection or osteomyelitis in the cervical spine
any terminal, systemic, or autoimmune disease
metabolic bone disease (e.g., osteoporosis, gout, osteomalacia, Paget disease)
any disease, condition, or surgery that might impair healing, such as:
insulin-dependent diabetes mellitus
active malignancy
history of metastatic malignancy
current or extended use (>6 mos) of any drug known to interfere with bone or soft-tissue healing
known metal allergy
arachnoiditis
currently experiencing an episode of major mental illness (psychosis, major affective disorder, or schizophrenia) or manifesting physical symptoms w/o a diagnosable medical condition to account for the symptoms
pregnancy at time of enrollment or planning to become pregnant
use of spinal stimulator at any cervical level prior to surgery

(continued)

TABLE 1: Inclusion and exclusion study criteria *(continued)*

Exclusion Criteria *(continued)*
currently a prisoner
currently involved in spinal litigation that may influence the patient's reporting of symptoms
participation in any other investigational drug, biological, or medical device study w/in the last 30 days prior to study surgery

rate was the same in both treatment arms. These analyses were performed for the protocol prespecified primary end point and for the alternate primary end point.

The individual components of success were summarized at 24 months using the available data. A Fisher exact test was used to compare the success rate in each of the 2 randomized arms. A secondary analysis of NDI data was performed using a 15-point threshold for improvement, in addition to the 20% criterion used in the primary end point evaluation.

Additionally, analysis of secondary end points and a comparison between the investigational and control groups were conducted. Covariates that had the potential to influence study outcome were also analyzed. These included sex, age, race, body mass index, level treated, baseline NDI, baseline VAS score, current smoker, previous surgery at treated level, work status, Workers' Compensation, baseline neurological status, and 24-month neurological status.

Device Design

Kineflex|C is a cobalt-chrome on cobalt-chrome alloy (metal-on-metal) semiconstrained disc (Fig. 1). It is a 3-piece design with 2 endplates and a mobile center core that can translate within a retention ring. The device is preassembled prior to insertion and applied in a single stage. A small midline keel on each endplate provides immediate fixation while the endplates are coated with titanium plasma spray to promote bony on-growth for long-term fixation. Figure 2A–D shows AP, lateral, flexion, and extension radiographs of the device.

Surgical Technique

The Kineflex|C surgical technique is similar to ACDF. The patient is positioned in a neutral supine position. Preoperative fluoroscopy is used to confirm the incision site and adequate visualization of the index level. A standard midcervical skin incision is used with exposure to the prevertebral space. Intraoperative fluoroscopy is used to confirm proper spinal alignment and localization. Discectomy is followed by symmetrical central decompression with resection of the posterior anulus and posterior longitudinal ligament. Care is taken to preserve the bony endplates; use of the high-speed drill is minimized or avoided altogether. As with other cervical arthroplasty devices, proximal foraminotomy is completed with symmetric proximal uncovertebral joint resection. Care is taken to preserve as much of the uncovertebral joint as possible while still effecting an adequate decompression. Midline verification is confirmed with AP fluoroscopy. The Kineflex|C disc is placed with a straightforward 4-step technique as follows: sizing (small and large), trialing/distraction (5, 6, and 7 mm), keel-cutting, and device placement. Attention is paid to proper sizing. Oversizing the disc can lead to limitation of motion, while undersizing can predispose to subsidence or heterotopic ossification. Trialing provides confirmation of proper disc height and maximal endplate coverage. A small keel cut is made in the midline to serve as a tract for device placement. After device placement, exposed bony surfaces are waxed, and AP and lateral fluoroscopy are used to confirm proper positioning.

TABLE 2: Radiographic classification of adjacent-level degeneration

Classification	Definition
0	none; negligible disc space narrowing, no osteophyte formation, no endplate sclerosis
1	mild; <33% disc space narrowing, mild osteophyte formation, no endplate sclerosis
2	moderate; 33–66% disc space narrowing, moderate osteophyte formation, mild to moderate endplate sclerosis
3	severe; >66% disc space narrowing, severe osteophyte formation, moderate to severe endplate sclerosis

Results

Patient Population and Study Design

Two hundred sixty-nine patients (136 with Kineflex|C and 133 with ACDF) were treated at 21 separate investigational sites. A minimum 24-month follow-up is avail-

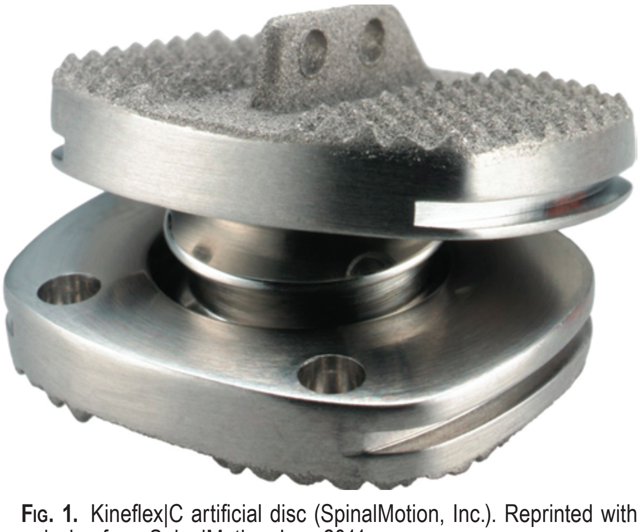

Fig. 1. Kineflex|C artificial disc (SpinalMotion, Inc.). Reprinted with permission from SpinalMotion, Inc., 2011.

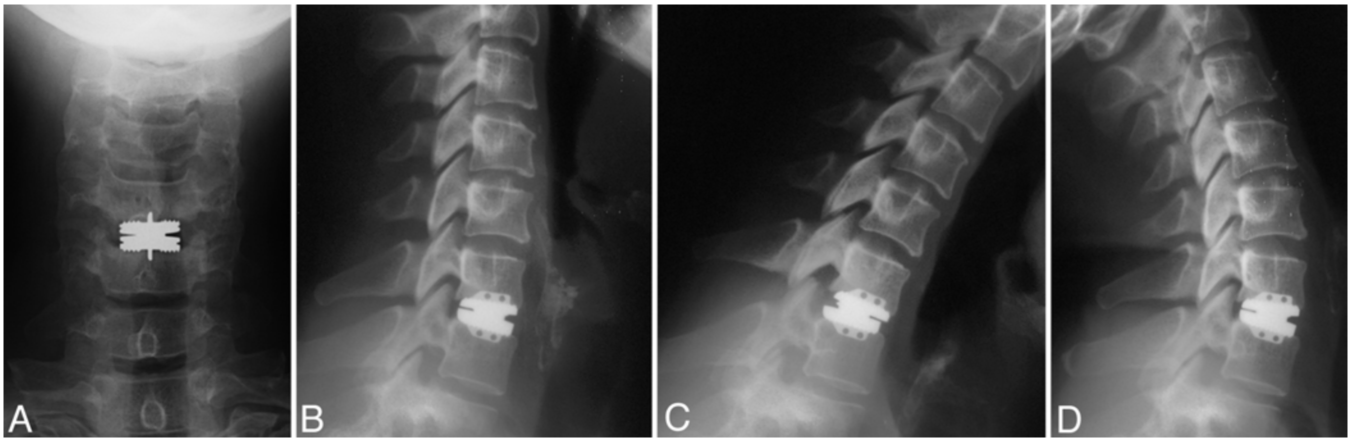

Fig. 2. Radiographs of the Kineflex|C device in AP **(A)**, lateral **(B)**, flexion **(C)**, and extension **(D)** views.

able for 234 patients (overall 87% follow-up: 119 patients in the Kineflex|C group [87.5% follow-up] and 115 patients in the ACDF group [86.5% follow-up]). The follow-up duration ranged from 24 to 34 months.

Demographics were similar between groups. The mean patient age in the Kineflex|C group was 43.7 years; that in the ACDF group was 43.9 years. There were no statistically significant differences in age between groups ($p = 0.88$). There were 51 men and 85 women in the Kineflex|C group and 59 men and 74 women in the ACDF group. Patient demographic data are shown in Table 3. The mean hospital stay, including the operative day, was 2.1 days in the Kineflex|C group and 2.1 days in the ACDF group; therefore, the vast majority of patients in both groups were discharged on postoperative Day 1. The mean operating room time was 80.2 minutes in the Kineflex|C group and 74.7 minutes in the ACDF group, and the mean blood loss was 40.6 ml in the Kineflex|C group and 41.1 ml in the ACDF group. There were no statistically significant differences in hospital stay ($p = 0.94$), operating room time ($p = 0.15$), or blood loss ($p = 0.83$). There were no blood transfusions in either group. There were no statistically significant differences between groups for tobacco use (52% in the Kineflex|C group and 47% in the ACDF group [$p = 0.46$]). Patient intraoperative data are shown in Table 4.

Overall clinical success was defined as a composite measure consisting of 5 separate components at the 24-month follow-up as follows: 1) maintenance or improvement in neurological evaluations including motor, sensory, and reflex examinations; 2) a minimum of 20% improvement in NDIs; 3) no device failure; 4) no reoperation at the index level; and 5) no major device-related adverse event. Patients who met all 5 of these criteria were considered clinical successes. Clinical success was significantly higher in the Kineflex|C group (85%) than in the ACDF group (71%) ($p = 0.05$; Fig. 3).

Clinical Indices

The NDI and VAS patient self-report scores were evaluated from preoperatively to 24 months follow-up.

Neck Disability Index. In the Kineflex|C group, the NDIs were as follows: 63.2 preoperatively, 32.2 at 1.5 months, 25.0 at 3 months, 22.5 at 6 months, 22.2 at 12 months, and 22.6 at 24 months. There was an average improvement of 65% from baseline with 91% showing a greater than 20% improvement at 24 months. In the ACDF group, the NDIs were as follows: 61.8 preoperatively, 33.3 at 1.5 months, 25.5 at 3 months, 22.8 at 6 months, 22.3 at 12 months, and 23.4 at 24 months. There was an average improvement of 62% from baseline, with 90% showing greater than 20% improvement at 24 months. Figure 4 shows preoperative through 24-month NDIs for the treatment and control groups. Figure 5 shows greater than 20% improvement in NDI rates at 24 months. Both groups at all follow-up times showed statistically significant improvement in NDIs from preoperative to a minimum 2-year follow-up ($p < 0.0001$). There was no statistical significance between groups.

Visual Analog Scale. In the Kineflex|C group, the

TABLE 3: Patient demographic data

Demographic Measure	Treatment Group			
	Kineflex|C	Fusion	Total	p Value*
no. of patients	136	133	269	
sex				0.2665
male	51 (37.5%)	59 (44.4%)	110 (40.9%)	
female	85 (62.5%)	74 (55.6%)	159 (59.1%)	
age (yrs)				0.8773
mean ± SD	43.7 ± 7.76	43.9 ± 7.39	43.8 ± 7.56	
median	44.0	44.0	44.0	
range	27–61	23–62	23–62	
height (in)				0.5819
mean ± SD	67.1 ± 3.70	67.3 ± 4.08	67.2 ± 3.88	
median	67.0	67.0	67.0	
range	60–77	57–79	57–79	
BMI (kg/m²)				0.1382
mean ± SD	27.5 ± 5.00	28.7 ± 5.70	28.1 ± 5.38	
median	27.2	27.3	27.3	
range	17.4–41.1	16.1–48.4	16.1–48.4	

* Fisher exact test for categorical variables and Wilcoxon rank-sum test for numeric variables.

TABLE 4: Patient intraoperative data*

	Treatment Group		
Surgery Measure	Kineflex|C	Fusion	p Value†
no. of patients	136	133	
op level			0.5093
C3–4	7 (5.1%)	3 (2.3%)	
C4–5	9 (6.6%)	6 (4.5%)	
C5–6	83 (61.0%)	83 (62.4%)	
C6–7	37 (27.2%)	41 (30.8%)	
LOS (days)			0.9373
mean ± SD	2.1 ± 0.43	2.1 ± 0.51	
median	2.0	2.0	
range	1.0–4.0	1.0–4.0	
total op time (min)			0.1467
mean ± SD	80.2 ± 28.93	74.7 ± 26.88	
median	72.0	69.0	
range	34.0–213.0	29.0–157.0	
EBL (ml)			0.8336
mean ± SD	40.6 ± 30.54	41.1 ± 32.42	
median	30.0	30.0	
range	0.0–200.0	0.0–200.0	

* EBL = estimated blood loss; LOS = length of stay.
† Fisher exact test for categorical variables and Wilcoxon rank-sum test for numeric variables.

VAS scores were as follows: 77.1 preoperatively, 26.3 at 1.5 months, 24.1 at 3 months, 25.0 at 6 months, 21.6 at 12 months, and 23.6 at 24 months. In the ACDF group, the VAS scores were as follows: 75.7 preoperatively, 27.8 at 1.5 months, 25.6 at 3 months, 24.2 at 6 months, 22.7 at 12 months postoperatively, and 24.2 at 24 months. Both groups at all follow-up times showed statistically significant improvement from preoperatively to a minimum 2-year follow-up ($p < 0.0001$). Figure 6 shows preoperative through 24-month pain self-report measures.

Patient Satisfaction. Patients were asked to self-report their level of satisfaction with their surgical procedure on the following scale: very satisfied, satisfied, somewhat satisfied, somewhat dissatisfied, dissatisfied, and very dissatisfied. At 24 months, 88% of Kineflex|C patients reported being very satisfied (75%) or satisfied (13%), with the remainder (12%) being somewhat satisfied or somewhat dissatisfied. At 24 months, 87% of ACDF patients reported being very satisfied (67%) or satisfied (20%), 9% being somewhat satisfied (6%) or somewhat dissatisfied (3%), and 4% being dissatisfied.

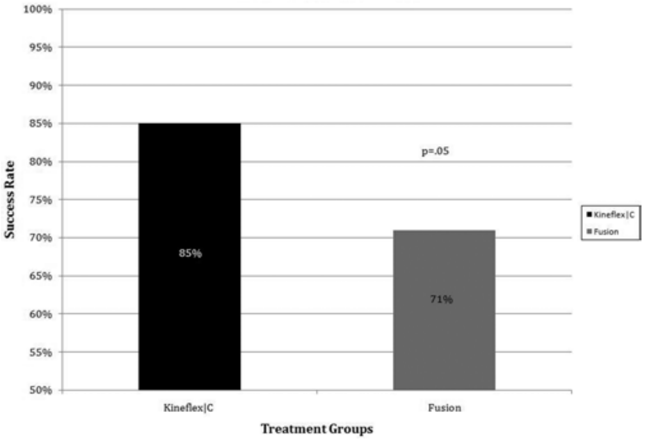

Fig. 3. Bar graph showing the composite measure of overall clinical success rates.

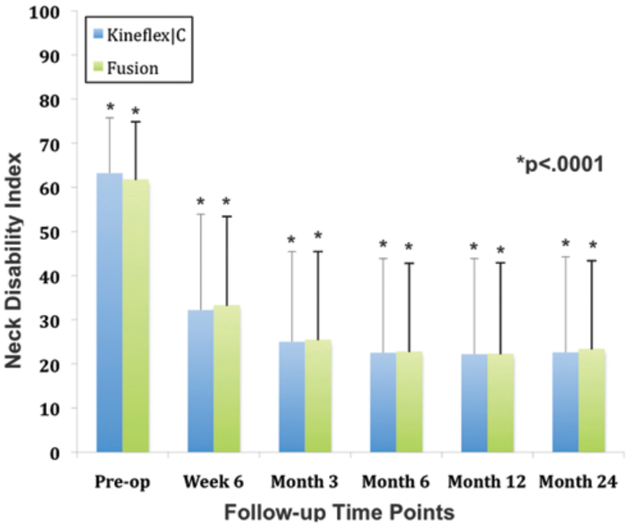

Fig. 4. Bar graph showing the patient self-report disability scores (NDI).

Neurological Status

Compared with baseline, neurological status (motor or sensory changes) was either stable (30%) or improved (65.5%) in 95% of patients in the Kineflex|C group. Neurological status was stable (35%) or improved (61%) in 95% of the ACDF group.

Radiographic Success

The range of motion (ROM) in the Kineflex|C group decreased at 3 months, but was significantly greater than the preoperative mean at the 12- ($p = 0.02$) and 24-month

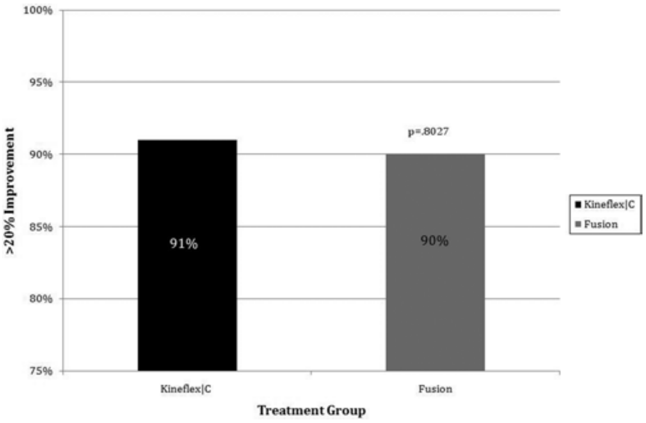

Fig. 5. Bar graph showing greater than 20% improvement in NDI scores.

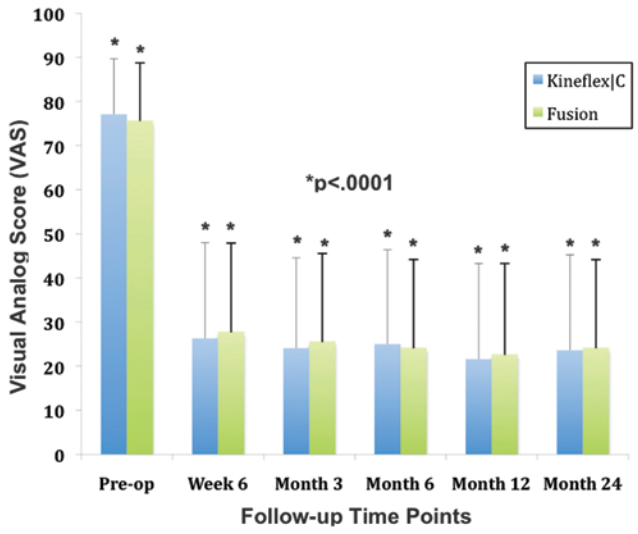

FIG. 6. Bar graph showing patient self-report pain scores (VAS).

(p = 0.001) follow-up times. In the Kineflex|C group, the angular motion was as follows: 8.2° preoperatively, 7.2° at 3 months, 8.9° at 6 months, 9.6° at 12 months, and 9.8° at 24 months. The ROM in the ACDF group was significantly reduced by 3 months and remained so throughout the follow-up (p < 0.0001). In the ACDF group, the angular motion was as follows: 7.6° preoperatively, 1.8° at 3 months, 1.5° at 6 months, 1.2° at 12 months, and 0.8° at 24 months. At the 2-year follow-up, 83% of Kineflex|C patients and 3% of ACDF patients showed at least 4° of motion at the index level (p < 0.0001). Figure 7 shows ROM from preoperatively to 24 months in both treatment groups.

Radiographic deterioration at the levels adjacent to the index level was independently graded as none, mild, moderate, or severe. Both groups, Kineflex|C and ACDF, showed no statistically significant difference in severe adjacent-level deterioration preoperatively (5.3% and 8.5%, respectively; p = 0.19). At the 24-month follow-up, there was significantly more severe adjacent-level deterioration evident in the ACDF group (24.8%) than in the Kineflex|C group (9%) (p < 0.0001). Figure 8 shows the assessment of adjacent-level deterioration in both groups at all time points.

In the ACDF group, there was a fusion rate of 82%. In the Kineflex|C group there was 1% incidence of bridging heterotopic ossifications.

Adverse Events

There were 4 cases (3.4%) of superficial infection in the Kineflex|C group and 5 (4.4%) in the ACDF group. There were no dural tears in the Kineflex|C group and 1 (0.9%) in the ACDF group. There were 2 patient self-report cases (1.7%) of persistent dysphagia/dysphonia in the Kineflex|C group and 7 (6.1%) in the ACDF group.

Reoperation

There were a total of 6 index-level reoperations (5.0%) in the Kineflex|C group. Four were for persistent or recurrent pain without device failure including 1 case in which the patient was found to have a delayed hyperreactivity to nickel. Two were for device migration, including one with vertebral body fracture. The first case of migration/fracture involved a 53-year-old woman with Kineflex|C placed at C6–7, which showed 6 mm of subsidence of the artificial disc into the C-7 vertebral body without trauma at the 6-week follow-up. The second case of migration involved a 39-year-old woman with Kineflex|C implanted at C5–6. At the 12-month follow-up, the patient showed 1.5 mm of anterolisthesis of C-5 on C-6, and she subsequently underwent revision 17 months postoperatively for recurrent neck and arm pain. All revision cases involved device removal with conversion to fusion. There were no complications in patients with revision surgery.

In the ACDF group, there was a total of 7 index-level reoperations (6.1%). Three were for pseudarthrosis revision and 4 were for instrumentation failure. Instrumentation failure was defined as removal or revision of the original anterior plate and screw construct.

There was a total of 9 adjacent level surgeries (7.6%) in the Kineflex|C group. Seven of the 9 underwent reoperation at the adjacent level within the first 12 months following the index-level surgery. Adjacent-level surgeries included 1 CTDR, 4 posterior cervical foraminotomies, and 4 ACDFs.

There was a total of 7 adjacent-level surgeries (6.1%) in the ACDF group. Six of the 7 underwent reoperation at the adjacent level after the first 12 months following index-level surgery. Adjacent level surgeries included 1 CTDR, 1 posterior cervical foraminotomy, and 5 ACDFs.

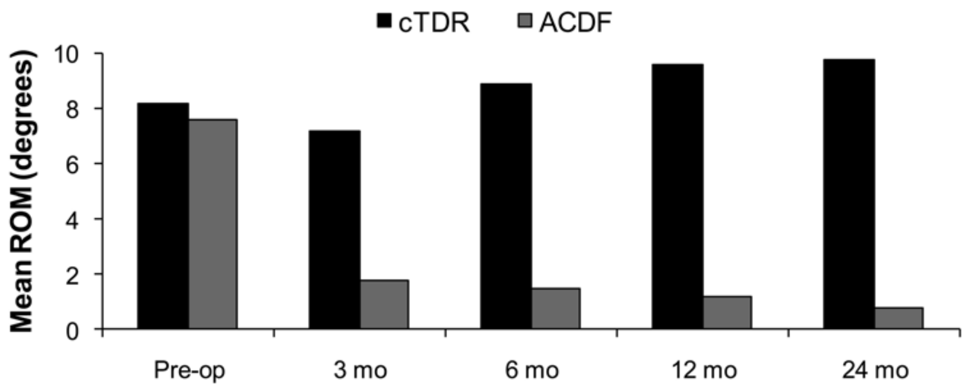

FIG. 7. Bar graph showing ROM.

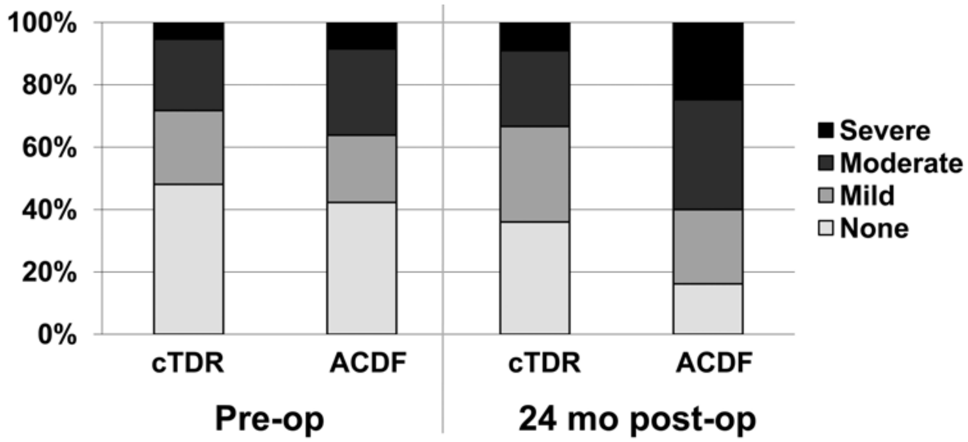

Fig. 8. Bar graph showing qualitative assessment of adjacent-level deterioration.

Activity Level

At baseline, 48% of Kineflex|C patients rated their activity level as active or moderately active (52% as light activities only or minimal activity possible). In the ACDF group at baseline, 45% of patients reported active/moderately active (54% light activities only/minimal activity possible). At the 24-month follow-up, 74% of Kineflex|C patients and 75% of ACDF patients reported their activity level as active or moderately active.

Discussion

As a relatively novel technology, there is wide variation in the design and biomechanical function of CTDR devices.[5,11,22,24,26,33,35] Kineflex|C is the first semiconstrained, metal-on-metal device with keel-based fixation to complete pivotal IDE study. In addition to safety and efficacy, an equally important consideration in evaluating new technology is the existing standard procedure to which it is being compared. One of the criticisms leveled at the lumbar total disc replacement IDE trials is that the fusion controls for the first devices to receive FDA approval were outmoded (BAK cages for Charité III, DePuy Spine) or inherently more complex than their comparison group (anterior/posterior 360° fusion for Prodisc-L [Synthes]). Conversely, ACDF with structural allograft and anterior plate stabilization is a relatively standardized procedure whose operative exposure is nearly identical to CTDR. Since ACDF is a well-established procedure with over 5 decades of clinical experience,[10,20,46] the noninferiority statistical design generally used by surgical device trials is justified.[35]

The composite overall success rates in the Kineflex|C (85%) and fusion (71%) groups reported in the current study are similar to the composite rates reported from the IDE studies of the first 3 cervical arthroplasty systems (79% for Prestige ST [Medtronic] and 68% for ACDF; 72% for Prodisc-C and 68% ACDF; and 83% for the Bryan Disc [Medtronic] and 73% for ACDF) to receive FDA approval.[26,33,35] Like the cervical artificial discs in all 3 of these previously published studies, the Kineflex|C group in the present study showed a greater overall success rate compared with the fusion control group. The Kineflex|C device also showed similar maintenance of segmental motion (degrees) at the treated level at 2 years (9.8°) as reported by the Prestige ST (7.7°), Bryan Disc (6.5°), and Prodisc-C (8.4°) devices.[26,33,35]

The radiographic success rate in the control ACDF group (82%) was lower than that reported from the IDE results of the first 3 CTDR systems (ACDF radiographic success rates: 97.5% in the Prestige LP study, 94% in the Bryan Disc study, and 90% in the Prodisc-C study).[26,33,35] The present study used a more stringent composite measure of radiographic fusion, which was prospectively defined by an independent core radiographic laboratory and reviewed by a single independent radiologist. Although the radiographic nonunion rate was higher than reported in the previous CTDR IDE studies, there were only 3 patients (2.6%) who underwent surgical revision for symptomatic failed fusion in the present study. Furthermore, if fusion was defined as an absence of motion utilizing the common standard of less than 2° of motion at the index level, the fusion rate of the control group would be 93.5%. The low rate of bridging heterotopic bone in the study Kineflex|C group (1%) was similar to reported rates of the Prestige ST (1%), Bryan (0%), and Prodisc-C (3%) artificial discs.[26,33,35]

In addition to superior clinical success rates, Kineflex|C also showed statistically significantly less severe adjacent-level radiographic changes compared with its ACDF control, even as early as the 2-year follow-up. Radiographic[3,29,31] and clinically[7,24,27,36] significant adjacent-level degeneration following ACDF has been well documented. Controversy exists as to the etiology of adjacent-level degeneration, but it has been established that fusion places increased stresses on adjacent levels compared with arthroplasty.[15–17,40,42,48,51] The results of the present study confirm a statistically higher rate of radiographically severe adjacent-level degenerative change in the ACDF group (25%) compared with the arthroplasty group (9%) at the 2-year follow-up. Recently, Kim and associates[30] also reported decreased degenerative change in patients treated with artificial discs. Given the relatively low number of patients enrolled in each individual arm of surgical device trials (~ 100–250 patients)[26,33,35] as well as the generally low expected incidence of adjacent-level surgery following ACDF (2–3%),[27] it would be difficult to observe statisti-

cally significant differences in adjacent-level surgery at 2-year follow-up. It is interesting to note that the majority of adjacent-level reoperations (78%) in the CTDR group occurred within the first 12 months following index-level surgery. Since only patients with single-level surgery were candidates for this IDE study, the investigators could not enroll patients and address what were deemed to be asymptomatic adjacent-level degenerated segments. Early (< 1 year postoperatively) adjacent-level surgery may involve degenerated segments that, in retrospect, were symptomatic. Long-term follow-up will establish whether the statistically higher rate of adjacent-level deterioration will result in higher rates of adjacent level reoperation.

There was a trend (not statistically significant) toward less patient self-report of persistent dysphagia/dysphonia in the Kineflex|C group (1.7%) compared with the control ACDF group (6.1%). Although the incidence of dysphagia/dysphonia following any anterior cervical approach is likely multifactorial, Kineflex|C is a zero-profile device and, as such, may positively influence this potential complication. Further research focusing on formal quantification and qualification of dysphagia and dysphonia with zero-profile devices is warranted.

Anterior cervical discectomy and fusion is a common surgical procedure with a straightforward exposure and an established record of safety.[2,7,25] Therefore, ideally the application of any CTDR device should closely mimic the ease and safety of the ACDF procedure. Intraoperative fluoroscopy is required for any CTDR device, and it generally requires greater attention to detail. For instance, with CTDR devices it is imperative to symmetrically decompress the disc space with resection of proximal uncovertebral joints, the anulus, and the posterior longitudinal ligament. Symmetrical decompression provides a posterior release to facilitate mobilization of the motion segment as well as proper artificial disc sizing and placement.[38,44] The procedure to place Kineflex|C adds only a keel-cutting step to the standard ACDF procedure. Bryan Disc placement requires substantial departure from the standard ACDF procedure with a frame-based application and endplate milling technique. The Prestige ST requires placement of anterior screw fixation. The similarity of the Kineflex|C placement to standard ACDF procedure is reflected in the nearly identical operative times for the 2 procedures reported in the present study (80 minutes for Kineflex|C and 75 minutes for ACDF), which did not demonstrate statistical significance ($p = 0.1467$). In fact, the Kineflex|C procedure time in the current study was shorter than the ACDF control procedures in earlier CTDR IDE studies.[26,33,35] Conversely, both Prodisc-C and Prestige ST showed statistically longer operating room times when compared with their fusion controls (107 minutes versus 99 minutes [$p = 0.0078$] and 96 minutes versus 84 minutes [$p < 0.001$], respectively) in their IDE pivotal studies.[33,35] In a separate study evaluating the results of the Bryan Disc at a single IDE investigational site, Coric et al.[13] reported a significantly greater operating room time for the Bryan disc (103 minutes) than for ACDF (79 minutes).

Challenges typically associated with artificial disc placement include subsidence, expulsion/dislocation, heterotopic ossification, and wear debris.[5,26,34,37,38,44] Specifically, the issue of wear debris causing osteolysis with any metal-on-metal arthroplasty device is a concern. There were no cases of osteolysis in the present study; this is consistent with the absence of osteolysis seen with the Prestige ST disc, also a metal-on-metal CTDR device.[33] There was a low reoperation rate (5%) in the Kineflex|C group in the present study. The majority of these (4 of 6 cases) were for persistent neck pain without device failure. Only 3 reoperations (2.5%) were device-related. ACDF is considered a more definitive procedure; therefore, all reoperations were directly related to failure of fusion or instrumentation. Typically, if a patient is solidly fused following ACDF, but with ongoing clinical symptoms (for example, persistent neck pain), another surgical procedure is not contemplated. This may explain the disassociation between the fusion success rate (82%) and the more modest overall clinical success rate (71%) reported in the current study. Conversely, with CTDR there remains a surgical alternative, namely ACDF, for patients with persistent clinical symptoms and no evidence of device failure.

The composite success measures used in IDE studies generally represent a stringent standard for success. This is evidenced by the considerably higher rate of patient self-report satisfaction (87% in the ACDF group and 88% in the Kineflex|C group) compared with the composite success rate (71% in the ACDF group and 85% in the Kineflex|C group). The more stringent composite success measure also explains the discrepancy between success rates reported in retrospective studies of ACDF[7,25] and those reported in prospective, randomized IDE studies.[8,26,33,35]

The safety and efficacy of cervical arthroplasty have been established with a growing body of Level I evidence. Anterior cervical discectomy and fusion,[4,7,10,13] CTDR,[8,13,26,33,35,45] and posterior cervical foraminotomy[1,12] are all proven treatments for cervical radiculopathy. Long-term follow-up and future studies will continue to define the ideal indications for these respective procedures as well as establish the role of cervical arthroplasty in expanded indications such as adjacent to fusion and multilevel disease.

Conclusions

Anterior cervical discectomy and fusion has an established record of clinical and radiographic efficacy, yet the procedure sacrifices the function of the motion segment.[2,4,7,10,16] The data reported in this prospective, randomized, multicenter study support the safety and efficacy of Kineflex|C and add to the growing body of Level I data that are compelling enough to no longer consider CTDR investigational.[13] Patients treated with Kineflex|C showed statistically superior overall clinical outcomes compared with ACDF while maintaining segmental motion. Furthermore, there were significantly fewer instances of severe adjacent-level radiographic degenerative changes in patients treated with Kineflex|C. Long-term follow-up will establish whether maintenance of motion and less radiographic degenerative change will ultimately result in fewer reoperations. The practical benefits of su-

perior clinical results and maintenance of motion do not require the potential added benefit of decreased adjacent level reoperations in order for CTDR to be viewed as a viable alternative to ACDF in select patients with cervical radiculopathy.

Disclosure

SpinalMotion, Inc. was responsible for the design and conduct of this multicenter, prospective, randomized FDA IDE pivotal trial (IDE No. G050035; Clinical Trial No. NCT00374413, available from www.clinicaltrials.gov) and provided clinical research support for it. SpinalMotion, Inc. provided statistical analysis for the study but did not provide any writing or editing assistance for this paper. The authors were provided with all the raw data from the study and had complete independence in deciding what data to present, including negative results.

Dr. Coric is a consultant for, received assistance with statistical analysis for the study from, and has direct stock ownership in SpinalMotion, Inc.; has direct stock ownership and is a consultant for Pioneer Surgical; has direct stock ownership in and is a consultant for Spinal Wave; and is a consultant for DePuy Spine. Dr. Guyer has direct stock ownership in and is a consultant for SpinalMotion, Inc. Dr. Musante received clinical or research support for the study described from SpinalMotion, Inc. Dr. Lauryssen has direct ownership in Spinal Kinetics and SpinalMotion, Inc.; is a patent holder with DePuy Spine; is a consultant for SpinalMotion, Inc.; and has ownership in Amedica and Replication Medical. Dr. Ohnmeiss received statistical analysis for the study from SpinalMotion, Inc. Dr. Nunley is a patent holder with, has direct stock ownership in, and is a consultant for K2M; has direct stock ownership in Amedica and Orthovita; and is a consultant for SpinalMotion, Inc., LDR Spine, and Nanovis, Inc. He also reports that his institution (Spine Institute of Louisiana, Shreveport, Louisiana) received research support for conduct of the IDE trial.

Author contributions to the study and manuscript preparation include the following. Conception and design: Coric, Nunley, Guyer, Musante. Acquisition of data: all authors. Analysis and interpretation of data: Coric, Guyer, Musante, Ohnmeiss, Boltes. Drafting the article: Coric. Critically revising the article: Coric, Guyer, Musante.

Acknowledgments

The authors thank the other principal investigators for their contributions to the study: John Abrahams, M.D., Ravinder Bains, M.D., Fabien D. Bitan, M.D., Scott Blumenthal, M.D., Rudolph Buckley, M.D., Thomas Dimmig, M.D., Gary Dix, M.D., Jeffrey Donner, M.D., Frederick E. Finger, M.D., Kevin T. Foley, M.D., Peter C. Gerszten, M.D., Harry N. Herkowitz, M.D., Reginald Q. Knight, M.D., James Rappaport, M.D., John Rhee, M.D., William Taylor, M.D., P. Justin Tortolani, M.D., and David Bradford, M.D., Medical Monitor. The principal author would also like to thank Ben Rogers, Raquel Scharkopf, and Charles Gilbride for help with manuscript preparation.

References

1. Adamson TE: Microendoscopic posterior cervical laminoforaminotomy for unilateral radiculopathy: results of a new technique in 100 cases. **J Neurosurg 95 (1 Suppl):**51–57, 2001
2. Aronson N, Filtzer DL, Bagan M: Anterior cervical fusion by the Smith-Robinson approach. **J Neurosurg 29:**396–404, 1968
3. Baba H, Furusawa N, Imura S, Kawahara N, Tsuchiya H, Tomita K: Late radiographic findings after anterior cervical fusion for spondylotic myeloradiculopathy. **Spine (Phila Pa 1976) 18:**2167–2173, 1993
4. Bailey RW, Badgley CE: Stabilization of the cervical spine by anterior fusion. **J Bone Joint Surg Am 42-A:**565–594, 1960
5. Bartels RH, Donk R: Fusion around cervical disc prosthesis: case report. **Neurosurgery 57:**E194, 2005
6. Bertagnoli R, Yue JJ, Pfeiffer F, Fenk-Mayer A, Lawrence JP, Kershaw T, et al: Early results after ProDisc-C cervical disc replacement. **J Neurosurg Spine 2:**403–410, 2005
7. Bohlman HH, Emery SE, Goodfellow DB, Jones PK: Robinson anterior cervical discectomy and arthrodesis for cervical radiculopathy. Long-term follow-up of one hundred and twenty-two patients. **J Bone Joint Surg Am 75:**1298–1307, 1993
8. Burkus JK, Haid RW, Traynelis VC, Mummaneni PV: Long-term clinical and radiographic outcomes of cervical disc replacement with the Prestige disc: results from a prospective randomized controlled clinical trial. Clinical article. **J Neurosurg Spine 13:**308–318, 2010
9. Chang UK, Kim DH, Lee MC, Willenberg R, Kim SH, Lim J: Changes in adjacent-level disc pressure and facet joint force after cervical arthroplasty compared with cervical discectomy and fusion. **J Neurosurg Spine 7:**33–39, 2007
10. Cloward RB: The anterior approach for removal of ruptured cervical disks. **J Neurosurg 15:**602–617, 1958
11. Coric D, Adamson TE: Minimally invasive cervical microendoscopic laminoforaminotomy. **Neurosurg Focus 25(2):**E2, 2008
12. Coric D, Cassis J, Carew JD, Boltes MO: Prospective study of cervical arthroplasty in 98 patients involved in 1 of 3 separate investigational device exemption studies from a single investigational site with a minimum 2-year follow-up. Clinical article. **J Neurosurg Spine 13:**715–721, 2010
13. Coric D, Finger F, Boltes P: Prospective randomized controlled study of the Bryan Cervical Disc: early clinical results from a single investigational site. **J Neurosurg Spine 41:**31–35, 2006
14. Cummins BH, Robertson JT, Gill SS: Surgical experience with an implanted artificial cervical joint. **J Neurosurg 88:**943–948, 1998
15. DiAngelo DJ, Roberston JT, Metcalf NH, McVay BJ, Davis RC: Biomechanical testing of an artificial cervical joint and an anterior cervical plate. **J Spinal Disord Tech 16:**314–323, 2003
16. Dmitriev AE, Cunningham BW, Hu N, Sell G, Vigna F, McAfee PC: Adjacent level intradiscal pressure and segmental kinematics following a cervical total disc arthroplasty: an in vitro human cadaveric model. **Spine (Phila Pa 1976) 30:**1165–1172, 2005
17. Eck JC, Humphreys SC, Lim TH, Jeong ST, Kim JG, Hodges SD, et al: Biomechanical study on the effect of cervical spine fusion on adjacent-level intradiscal pressure and segmental motion. **Spine (Phila Pa 1976) 27:**2431–2434, 2002
18. Emery SE, Bohlman HH, Bolesta MJ, Jones PK: Anterior cervical decompression and arthrodesis for the treatment of cervical spondylotic myelopathy. Two to seventeen-year follow-up. **J Bone Joint Surg Am 80:**941–951, 1998
19. Fountas KN, Kapsalaki EZ, Nikolakakos LG, Smisson HF, Johnston KW, Grigorian AA, et al: Anterior cervical discectomy and fusion associated complications. **Spine (Phila Pa 1976) 32:**2310–2317, 2007
20. Fraser JF, Härtl R: Anterior approaches to fusion of the cervical spine: a metaanalysis of fusion rates. **J Neurosurg Spine 6:**298–303, 2007
21. Garrido BJ, Taha TA, Sasso RC: Clinical outcomes of Bryan cervical disc arthroplasty a prospective, randomized, controlled, single site trial with 48-month follow-up. **J Spinal Disord Tech 23:**367–371, 2010
22. Goffin J, Casey A, Kehr P, Liebig K, Lind B, Logroscino C, et al: Preliminary clinical experience with the Bryan Cervical Disc prosthesis. **Neurosurgery 51:**840–847, 2002
23. Goffin J, Geusens E, Vantomme N, Quintens E, Waerzeggers Y, Depreitere B, et al: Long-term follow-up after interbody fusion of the cervical spine. **J Spinal Disord Tech 17:**79–85, 2004

24. Goffin J, Van Calenbergh F, van Loon J, Casey A, Kehr P, Liebig KE, et al: Intermediate follow-up after treatment of degenerative disc disease with the Bryan Cervical Disc Prosthesis: single-level and bi-level. **Spine (Phila Pa 1976) 28:**2673–2678, 2003
25. Gore DR, Sepic SB: Anterior cervical fusion for degenerated or protruded discs. A review of one hundred forty-six patients. **Spine (Phila Pa 1976) 9:**667–671, 1984
26. Heller JG, Sasso RC, Papadopoulos SM, Anderson PA, Fessler RG, Hacker RJ, et al: Comparison of BRYAN cervical disc arthroplasty with anterior cervical decompression and fusion: clinical and radiographic results of a randomized, controlled, clinical trial. **Spine (Phila Pa 1976) 34:**101–107, 2009
27. Hilibrand AS, Carlson GD, Palumbo MA, Jones PK, Bohlman HH: Radiculopathy and myelopathy at segments adjacent to the site of a previous anterior cervical arthrodesis. **J Bone Joint Surg Am 81:**519–528, 1999
28. Hunter LY, Braunstein EM, Bailey RW: Radiographic changes following anterior cervical fusion. **Spine (Phila Pa 1976) 5:**399–401, 1980
29. Ishihara H, Kanamori M, Kawaguchi Y, Nakamura H, Kimura T: Adjacent segment disease after anterior cervical interbody fusion. **Spine J 4:**624–628, 2004
30. Kim SW, Limson MA, Kim SB, Arbatin JJ, Chang KY, Park MS, et al: Comparison of radiographic changes after ACDF versus Bryan disc arthroplasty in single and bi-level cases. **Eur Spine J 18:**218–231, 2009
31. Kulkarni V, Rajshekhar V, Raghuram L: Accelerated spondylotic changes adjacent to the fused segment following central cervical corpectomy: magnetic resonance imaging study evidence. **J Neurosurg 100 (1 Suppl Spine):**2–6, 2004
32. Matsunaga S, Kabayama S, Yamamoto T, Yone K, Sakou T, Nakanishi K: Strain on intervertebral discs after anterior cervical decompression and fusion. **Spine (Phila Pa 1976) 24:** 670–675, 1999
33. Mummaneni PV, Burkus JK, Haid RW, Traynelis VC, Zdeblick TA: Clinical and radiographic analysis of cervical disc arthroplasty compared with allograft fusion: a randomized controlled clinical trial. **J Neurosurg Spine 6:**198–209, 2007
34. Mummaneni PV, Haid RW: The future in the care of the cervical spine: interbody fusion and arthroplasty. **J Neurosurg Spine 1:**155–159, 2004
35. Murrey D, Janssen M, Delamarter R, Goldstein J, Zigler J, Tay B, et al: Results of the prospective, randomized, controlled multicenter Food and Drug Administration investigational device exemption study of the ProDisc-C total disc replacement versus anterior discectomy and fusion for the treatment of 1-level symptomatic cervical disc disease. **Spine J 9:**275–286, 2009
36. Park JB, Cho YS, Riew KD: Development of adjacent-level ossification in patients with an anterior cervical plate. **J Bone Joint Surg Am 87:**558–563, 2005
37. Parkinson JF, Sekhon LH: Cervical arthroplasty complicated by delayed spontaneous fusion. Case report. **J Neurosurg Spine 2:**377–380, 2005
38. Pickett GE, Sekhon LH, Sears WR, Duggal N: Complications with cervical arthroplasty. **J Neurosurg Spine 4:**98–105, 2006
39. Pospiech J, Stolke D, Wilke HJ, Claes LE: Intradiscal pressure recordings in the cervical spine. **Neurosurgery 44:**379–385, 1999
40. Puttlitz CM, Rousseau MA, Xu Z, Hu S, Tay BK, Lotz JC: Intervertebral disc replacement maintains cervical spine kinetics. **Spine (Phila Pa 1976) 29:**2809–2814, 2004
41. Rabin D, Bertagnoli R, Wharton N, Pickett GE, Duggal N: Sagittal balance influences range of motion: an in vivo study with the ProDisc-C. **Spine J 9:**128–133, 2009
42. Reitman CA, Hipp JA, Nguyen L, Esses SI: Changes in segmental intervertebral motion adjacent to cervical arthrodesis: a prospective study. **Spine (Phila Pa 1976) 29:**E221–E226, 2004
43. Robertson JT, Papadopoulos SM, Traynelis VC: Assessment of adjacent-segment disease in patients treated with cervical fusion or arthroplasty: a prospective 2-year study. **J Neurosurg Spine 3:**417–423, 2005
44. Sekhon LH: Cervical arthroplasty in the management of spondylotic myelopathy. **J Spinal Disord Tech 16:**307–313, 2003
45. Sekhon LH, Sears W, Duggal N: Cervical arthroplasty after previous surgery: results of treating 24 discs in 15 patients. **J Neurosurg Spine 3:**335–341, 2005
46. Smith GW, Robinson RA: The treatment of certain cervical-spine disorders by anterior removal of the intervertebral disc and interbody fusion. **J Bone Joint Surg Am 40-A:**607–624, 1958
47. Walraevens J, Liu B, Meersschaert J, Demaerel P, Delye H, Depreitere B, et al: Qualitative and quantitative assessment of degeneration of cervical intervertebral discs and facet joints. **Eur Spine J 18:**358–369, 2009 (Erratum in **Eur Spine J 18:** 370, 2009)
48. Wang JC, McDonough PW, Endow K, Kanim LE, Delamarter RB: The effect of cervical plating on single-level anterior cervical discectomy and fusion. **J Spinal Disord 12:**467–471, 1999
49. Weinhoffer SL, Guyer RD, Herbert M, Griffith SL: Intradiscal pressure measurements above an instrumented fusion. A cadaveric study. **Spine (Phila Pa 1976) 20:**526–531, 1995
50. Wigfield C, Gill S, Nelson R, Langdon I, Metcalf N, Robertson JT: Influence of an artificial cervical joint compared with fusion on adjacent-level motion in the treatment of degenerative cervical disc disease. **J Neurosurg 96 (1 Suppl):**17–21, 2002
51. Wigfield CC, Skrzypiec D, Jackowski A, Adams MA: Internal stress distribution in cervical intervertebral discs: the influence of an artificial cervical joint and simulated anterior interbody fusion. **J Spinal Disord Tech 16:**441–449, 2003
52. Yue WM, Brodner W, Highland TR: Long-term results after anterior cervical discectomy and fusion with allograft and plating: a 5- to 11-year radiologic and clinical follow-up study. **Spine (Phila Pa 1976) 30:**2138–2144, 2005

Manuscript submitted November 1, 2010.
Accepted May 10, 2011.
Please include this information when citing this paper: published online June 24, 2011; DOI: 10.3171/2011.5.SPINE10769.
Address correspondence to: Dom Coric, M.D., Carolina Neurosurgery & Spine, 225 Baldwin Avenue, Charlotte, North Carolina 28207. email: dom@cnsa.com.
