# Supplementary material for: Adjacent segment degeneration or disease after cervical total disc replacement: a meta-analysis of randomized controlled trials
Source: J Orthop Surg Res. 2018 Oct 3;13:244. doi: 10.1186/s13018-018-0940-9 (PMC6169069; doi:10.1186/s13018-018-0940-9)
Supplement: Supplementary file 2 — File S1. Original data of 11 included articles. (ZIP 12 mb) [file 13018_2018_940_MOESM2_ESM.zip › 11 included articles and original data referred in this article/11 included articles/24 Nunley, P D(US).pdf]

Spine

SPINE Volume 37, Number 6, pp 445–451
©2012, Lippincott Williams & Wilkins

RANDOMIZED TRIAL

# Factors Affecting the Incidence of Symptomatic Adjacent-Level Disease in Cervical Spine After Total Disc Arthroplasty

## *2- to 4-Year Follow-up of 3 Prospective Randomized Trials*

Pierce D. Nunley, MD,* Ajay Jawahar, MD, MS,* Eubulus J. Kerr III, MD,* Charles J. Gordon, MD,† David A. Cavanaugh, MD,* Elisa M. Birdsong, BS,* Marolyn Stocks, RN, BSN,† and Guy Danielson, MD†

**Study Design.** Prospective randomized clinical trials.

**Objective.** To compare the outcome data with respect to clinical success rates and incidence of adjacent level disease (ALD) in patients after total disc arthroplasty (TDA) or anterior cervical fusion (ACDF) for 1- and 2-level cervical disc disease.

**Summary of Background Data.** Previously published studies have provided evidence that ACDF procedure for cervical degenerative disc disease (DDD) may increase the stress on the nonoperated adjacent cervical segments, thus possibly increasing the risk of degeneration at these levels. The theoretical assumption that TDA may reduce the incidence of future ALD by preserving motion at the affected segments has, however, never been validated by clinical evidence.

**Methods.** One hundred seventy patients with established symptomatic cervical disc disease at 1 or 2 levels participated in 3 prospective randomized clinical trials at 2 institutions. Participating subjects were randomized to receive TDA (treatment; n = 113) or ACDF (control; n = 57) by 6 independent investigating surgeons. Visual analogue pain scores (0–100), Neck Disability Index, neurological examination, and cervical spine radiographs were collected at enrollment and then 6 weeks and 3, 6, 12, 24, 36, and 48 months after surgery. Patients with persistent symptoms during the follow-up period were investigated for adjacent segment disease (ASD) with computed tomography/magnetic resonance imaging of the cervical spine, neurophysiology, and subsequent active interventions.

**Results.** At the median follow-up of 42 months (range: 28–54 months), 9 (14.3%) ACDF and 19 (16.8%) TDA patients developed and were actively treated for ASD. Osteopenia dust energy x-ray absorptiometry T scores of −1.5 to −2.4) (*P* = 0.04; 95% confidence interval [CI]: 0.007–0.223) and concurrent lumbar degenerative disease (*P* = 0.02; 95% CI: 0.003–0.196) significantly increased the risk of ASD.

**Conclusion.** The risk of developing adjacent segment degeneration was equivalent at median 38 months after both ACDF and TDA procedures in cervical DDD. Osteopenia and concurrent lumbar DDD significantly increase the risk of ALD.

**Key words:** arthroplasty, adjacent level disease, cervical spine, total disc. **Spine 2012;37:445–451**

Since its introduction by Caspar *et al*,[1] instrumented anterior cervical discectomy and fusion (ACDF) has been commonly performed and considered as the surgical standard of care to manage intractable radiculopathic and/or myelopathic symptoms resulting from herniated nucleus pulposus (HNP) and degenerative disc disease (DDD) of the cervical spine.[2] Previously published series have reported clinical and radiological outcomes of the procedure for symptomatic relief and restoring neurological functions in adult patients regardless of age and sex.[3–5] However, as longer-term results of the procedure became available, the outcome studies increasingly emphasized the adverse effects of this procedure on the cervical spine.[6,7] One of the areas specifically investigated was the effects of fusion biomechanically[8] and clinically[9,10] on the intervertebral discs adjacent to the level fused. Hilibrand *et al*[9] established that symptomatic adjacent segment disease (ASD) occurred in a fourth of the patient population within the first 10 years after ACDF. These results led scientists and surgeons to focus their attention toward developing alternative procedures for DDD that would maintain the basic motion of the intervertebral segments, hence protecting the adjacent segments against nonphysiological loading. This concept led to the development of cervical total disc arthroplasty (TDA).[11–13] Since then, several TDA implants have been used for treating cervical DDD, and the clinical outcomes have been published in the literature.[14–19]

From the *Spine Institute of Louisiana, Shreveport, Louisiana; and †Texas Spine and Joint Institute, Tyler, Texas.

Acknowledgment date: February 18, 2011. First revision date: April 18, 2011. Acceptance date: April 25, 2011.

The device(s)/drug(s) that is/are the subject of this manuscript is/are being evaluated as part of an ongoing FDA-approved investigational protocol (IDE) or corresponding national protocol for cervical radiculopathy.

No funds were received in support of this work. No benefits in any form have been or will be received from a commercial party related directly or indirectly to the subject of this manuscript.

Address correspondence and reprint requests to Ajay Jawahar, MD, MS, Spine Institute of Louisiana, 1500 Line Ave, Ste 200, Shreveport, LA 71101; E-mail: ajawahar@louisianaspine.org

DOI: 10.1097/BRS.0b013e31822174b3



Copyright © 2012 Lippincott Williams & Wilkins. Unauthorized reproduction of this article is prohibited.

The purpose of the present work is to examine the incidence of symptomatic ASD at 3 and 4 years' follow-up in 170 patients with 1- and 2-level cervical DDD who received either TDA or ACDF in 3 different prospective, randomized clinical trials.

## MATERIALS AND METHODS

### Study Design

The trials were conducted as a part of the United States Food and Drug Administration (USFDA) investigational device exemption (IDE) pivotal protocols for the total disc replacement implants. Since December 2005, the 2 collaborating institutions have participated as investigational sites to assess the clinical safety and efficacy of 3 different artificial cervical discs. A total of 6 qualified surgeons were involved as clinical investigators in the trials. All trials were prospective randomized trials with participating subjects being blinded to the assigned treatment until after the surgical procedures. They were conducted under the oversight of the institutional review board with strict adherence to the guidelines of good clinical practices (GCP).

### Patient Cohort

All patients with documented 1- and/or 2-level symptomatic cervical DDD who had failed at least 6 months of active conservative management were screened as potential participants for the clinical trials. The cervical DDD was documented by clinical signs and symptoms, plain radiographs of the cervical spine, magnetic resonance imaging (MRI) of the cervical spine, and electrophysiological studies. Active conservative treatment included pain medication (narcotics and nonnarcotics), physical therapy, chiropractic manipulations, and transforaminal epidural steroid injections. A total of 664 patients presenting in the outpatient clinics were screened during the active enrollment phase over a 2-year period (2005–2007). The preoperative images (radiographs and MRIs) of the cervical spine were very carefully scrutinized by the participating investigators. Any radiological evidence of degeneration as evidenced in radiographs and/or MRI at levels other than the "index levels" to be treated caused the screened subjects to be excluded from participation in the trials. As per the inclusion/exclusion criteria, 182 patients that qualified were consented and enrolled. The inclusion/exclusion criteria were similar in all 3 trials and have been listed in Table 1. The participating patients were assigned to receive TDA or ACDF by an offsite, unbiased, computerized randomization method. The randomization scheme was 1:1 in 1 trial and 2:1 in favor of the TDA for the other 2 trials.

At the time of screening for enrollment, one of the questions asked to all patients was "In the past 5 years, have you been actively worked-up/treated for low back pain?" If the patients answered "yes" to the question, all documentation regarding their low back pain was collected. This review identified 51 patients who had documented DDD of the lumbar spine as recorded at the time of screening and enrollment. These patients suffered from symptoms (low back pain or radicular) associated with lumbar spine disease. Their lumbar DDD had been further documented by the preexisting MRI scans of the lumbar spine and documentation of active treatment or intervention for symptoms related to lumbar DDD.

**TABLE 1. Inclusion/Exclusion Criteria for Enrollment Into the Investigational Device Exemption Trials**

| |
|---|
| **Inclusion Criteria** |
| Skeletally mature patients |
| Diagnosis of radiculopathy or myeloradiculopathy of the cervical spine in a specific nerve root distribution C3 through C7 |
| Neck and/or arm pain VAS at least 30 mm on 100-mm scale |
| Neck Disability Index Score ≥ 30 points |
| Unresponsive to conservative treatment for at least 6 months |
| Absence of any medical condition that would interfere with the proposed surgery |
| Subject provided informed consent and is willing to comply with the protocol |
| No prior surgery at the index levels |
| **Exclusion Criteria** |
| More than 1 immobile vertebral level between C1 and C7 from any cause including but not limited to congenital abnormalities and osteoarthritic "spontaneous" fusions |
| Previous trauma to the C3 to C7 levels resulting in significant bony or discoligamentous cervical spine injury |
| Axial neck pain in the absence of other symptoms of radiculopathy or myeloradiculopathy justifying the need for surgical intervention |
| Radiographic confirmation of severe facet joint disease or degeneration |
| Symptomatic degenerative disc disease or significant cervical spondylosis at more than 2 levels |
| Spondylolysis |
| Marked cervical instability on resting lateral or flexion/extension radiographs demonstrated by translation greater than 3.5 mm, and/or greater than 11° angular difference to that of either adjacent level |

The protocols mandated exclusion of osteoporotic patients from participation. According to the FDA guidelines, osteoporosis was defined as composite "T score" of negative 2.5 or less on the bone density scan (dual-energy x-ray absorptiometry [DEXA]) for lumbar spine. However, 28 patients demonstrating *osteopenic* bone density status (T scores for spine between −2.4 and −1.5) were included and equally randomized in the 2 treatment groups.

### Devices and Procedures

All 3 TDR devices had satisfied the FDA safety criteria for investigational clinical trials. The implanting surgeons were trained in the cadaver laboratory and certified by the manufacturers. ACDF was performed using the modified Smith Robinson Technique.[2] Cortical bone allograft and anterior



Copyright © 2012 Lippincott Williams & Wilkins. Unauthorized reproduction of this article is prohibited.

plates were used in all cases for fusion. The use of BMP was not permitted in any of the protocols.

### Primary Outcome Parameters

Because these were FDA device exemption trials to prove noninferiority of the TDA to ACDF, the clinical outcome data pertaining to the patient's index surgery level were collected periodically and provided to the sponsors. These data consisted of visual analogue scores (VAS) for neck and arm pain, Neck Disability Index (NDI), SF-12 health survey, a complete neurological examination by a qualified clinician, and plain radiographs of cervical spine in 6 views (anteroposterior view in neutral, right, and left bending; lateral in neutral, flexion, and extension positions). Analysis of these data, although important from the primary study point of view, does not form the basis for the presented work.

### Adjacent Segment Disease

The specific identification of ASD was not required as a primary or secondary end point for any of the IDE protocols. However, as with any other IDE trial, all patients who were reported to have new clinical signs or symptoms had to be documented as "adverse events." As a standard of clinical care, the patients who presented with recurrent clinical stigmata of cervical radiculopathy/myelopathy had repeat MRI or computed tomography (CT) scans of the cervical spine in addition to the plain radiographs performed as a part of the protocol. The imaging studies were carefully analyzed for evidence of degeneration at the nonoperated levels according to the criteria previously validated by Hilibrand *et al* in 1999.[9] Upon detection of radiological evidence of ASD, the investigators compared the imaging studies with those performed at the baseline to rule out preexisting degeneration. The patients who demonstrated a clinicoradiological correlation between their symptoms and imaging studies had electrophysiological (electromyography and nerve conduction velocity) studies to rule out peripheral nerve pathologies and corroborate the diagnosis of adjacent segment pathology. Once the existence of ASD was established, a careful record was maintained for subsequent surgery or medical management for their ASD with prescription pain medications, physical therapy, and at least 1 epidural steroid injection at the time of finalizing the presented data. Because it was our intention to identify the true incidence of symptomatic ASD after the procedures, only those patients who demonstrated clinicoradiological stigmata of ASD and received active intervention for its management were included in the statistical analysis.

### Statistical Analysis

All data management and statistical analysis were performed using SPSS version 15.0 (SPSS Inc, Chicago, IL). The primary outcome of interest was the duration for which the patients remained free from ALD (ALD-free survival). ALD-free survival was calculated as the time from surgery to the diagnosis of ALD or the last follow-up. The association of the index procedure (ACDF *vs.* TDA); number of levels fused (1 *vs.* 2); increasing age; sex; osteopenia (as calculated by the DEXA bone scans); smoking habits; and concurrent presence of lumbar DDD at the time of cervical procedure were assessed *via* proportional hazards regression analysis. Variables independently associated with ALD-free survival in multivariate analysis were incorporated to generate Kaplan-Meier survival curves to predict the actuarial (estimated) time of freedom from ASD in each randomized group (ACDF and TDA). The analysis was performed with observed power values of all tests at the 0.05 significance level.

## RESULTS

### Demographics

One hundred twenty (66.5%) patients were randomized to receive TDA, and 62 (34%) received ACDF. Mean patient age was 44.5 years (range: 22–67 years) with marginal female predominance (55%). Sixty patients (32.9%) were habitual smokers. All characteristics were equally distributed for both groups (ACDF and TDA), as is evident in Table 2. The protocols for all 3 IDE's required a minimum follow-up of 24 months. Twelve patients (7 with TDA and 5 with ACDF) did not complete the follow-up. Complete follow-up was thus available in 170 enrolled subjects. Of these, 113 had TDA, and 57 received ACDF as the index surgical procedure. Figure 1 represents a CONSORT flow diagram for the trial.

### Adjacent Segment Disease

The overall follow-up period ranged from 32 to 54 months (median 38 months). ASD was established in 28 (16.5%) patients during this follow-up period according to the clinical and radiological criteria described earlier. Seven patients (4.1%) were categorized as ALD grade IV (severe disease) according to the Hilibrand criteria.[9] These seven patients underwent second surgery at the adjacent level. Five patients underwent ACDF, whereas the other 2 had posterior decompression. The remaining 21 patients (12.3%) have been categorized as ALD grades II (n = 12) or grade III (n = 9) by their respective assessing surgeons and continue to be managed

**TABLE 2. Demographic Comparison of Patients in the TDA and ACDF Groups: *$P > 0.5$ for All Categories**

| Characteristic | ACDF Group | TDA Group |
|---|---|---|
| Mean age ± SD (years) | 43 ± 7.9 | 45 ± 5.02* |
| Females (%) | 52.6 | 56.6* |
| Smokers (%) | 29.8 | 38.1* |
| Osteopenia (%) | 14 | 15* |
| Lumbar DDD (%) | 29 | 31* |
| Mean VAS ± SD | 79 ± 17.9 | 77 ± 16.9* |
| Mean NDI ± SD | 59 ± 13.3 | 58 ± 13.6* |
| Levels—ratio (single: 2 levels) | 71:29 | 70:30* |

*TDA indicates total disc arthoplasty; ACDF, anterior cervical fusion; DDD, degenerative disc disease; VAS, visual analogue store; NDI, Neck Disability Index.*



Copyright © 2012 Lippincott Williams & Wilkins. Unauthorized reproduction of this article is prohibited.

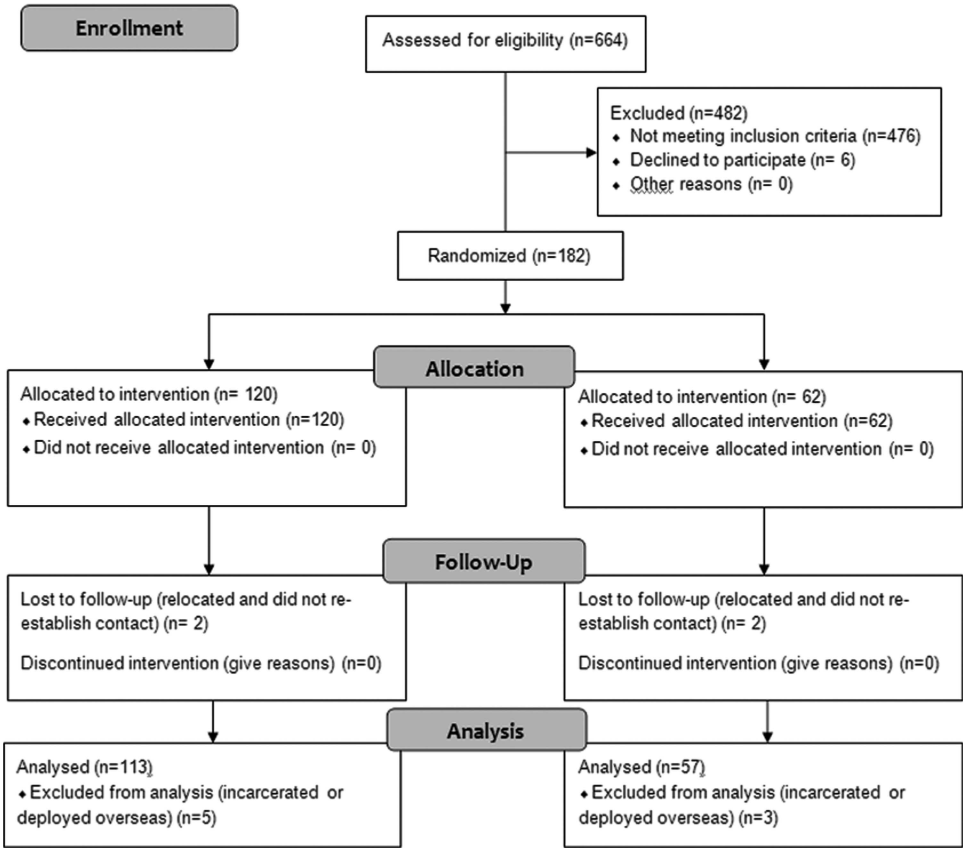


**Figure 1.** CONSORT flow diagram for the trials.

conservatively with prescription pain medications, physical therapy, and at least 1 epidural steroid injection at the time of finalizing the presented data.

Life tables and Kaplan-Meier survival computations project the overall ALD-free survival rates at 3 and 4 years to be 86.8% ± 2.9% and 77.6% ± 6.3%, respectively, for the whole cohort. The mean ALD-free period after anterior cervical surgery for 1- or 2-level DDD is estimated at 49.4 months (95% confidence interval of 47.8–50.9 months) in the present series (Figure 2).

## Effect of the Index Procedure

At the last follow-up, 94 (83.2%) patients with TDA and 49 (86%) patients with ACDF were free of ALD. There was no significant difference in the incidence of ALD in the 2 groups. The actuarial FLD survival rate at 4 years was 78.3% ± 0.85% for the TDA group and 76.7% ± 0.56% for the ACDF group ($P$ = 0.24). Similarly, the mean period of freedom from ALD was 46.04 ± 0.6 months after ACDF and 48.7 ± 1.04 months after TDA. Figure 3 shows the comparative ALD-free survival curves for both groups. No statistical significance could be established for the type of procedure (fusion *vs*. disc arthroplasty) influencing freedom from adjacent segment degeneration at a mean follow-up of 42 months.

## Effect of Osteopenia

Survival analysis for the ALD-free period shows the actuarial rate for nonosteopenic group as 82.3% ± 0.42% and for the osteopenic group (T score > −1.5) as 54.0% ± 1.76% ($P$ = 0.04; 95% CI: 0.007–0.223). This difference is statistically significant, thus giving the projected mean period of freedom from ALD as 42.0 ± 1.7 months (95% CI: 38.6–45.4 months) for the osteopenic group and 49.8 ± 0.8 months (95% CI: 48.1–51.4 months) for the nonosteopenic group (Figure 4).

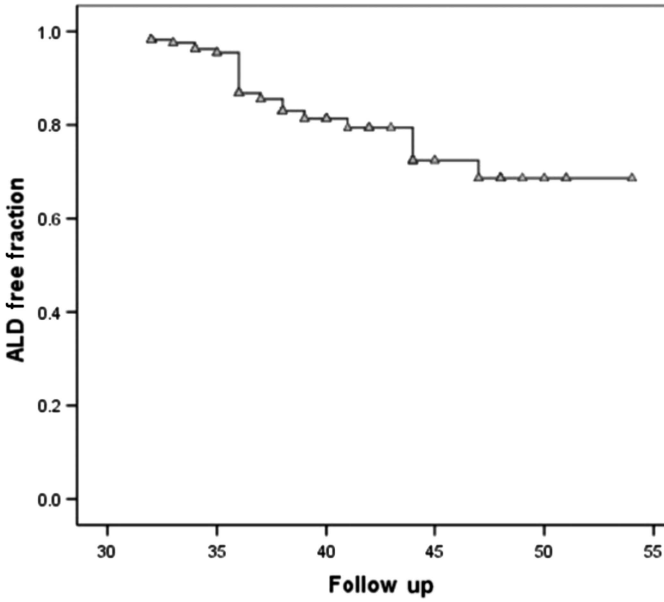


**Figure 2.** Kaplan-Meier survival plot for overall incidence of adjacent level disease (ALD).


Copyright © 2012 Lippincott Williams & Wilkins. Unauthorized reproduction of this article is prohibited.

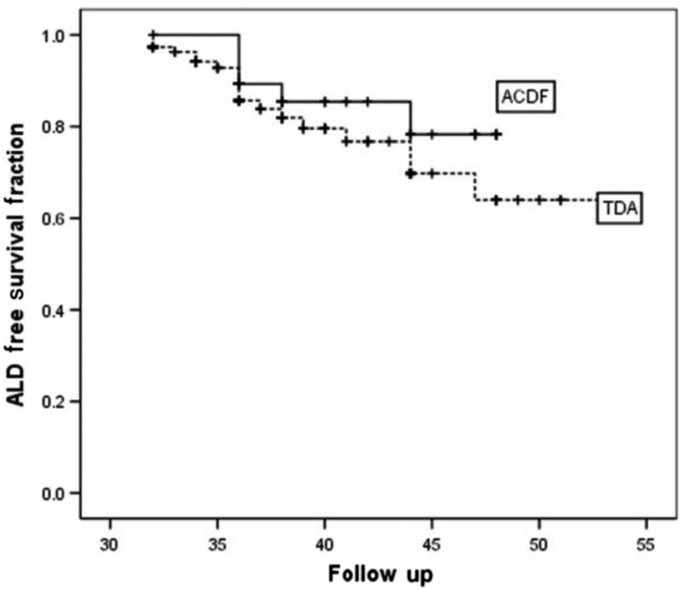


**Figure 3.** Kaplan-Meier survival plot for effect of index procedure on the incidence of adjacent level disease (ALD).

## Effect of Coexisting Lumbar Degenerative Disease

Analysis for ALD shows a 4-year ALD-free survival rate of 74.5% ± 0.6% for patients with no lumbar disease and 55.5% ± 0.12% for those with lumbar DDD. This statistically significant difference ($P = 0.023$; 95% CI: 0.003–0.196) is also reflected in the mean actuarial ALD-free survival times, which are 50.3 ± 0.8 months (95% CI: 48.6–52.3 months) for patients without lumbar disease and 45.7 ± 1.2 months (95% CI: 43.2–48.2 months) for those with lumbar DDD (Figure 5).

Table 3 shows the 4-year disease-free survival rates and the projected mean periods of freedom from ALD in the different patient groups discussed earlier.

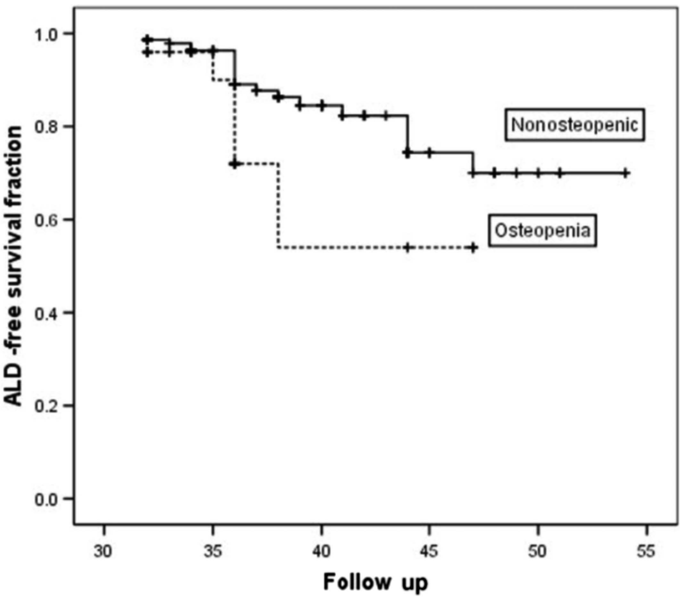


**Figure 4.** Kaplan-Meier survival plot for effect of osteopenia on the incidence of adjacent level disease (ALD).

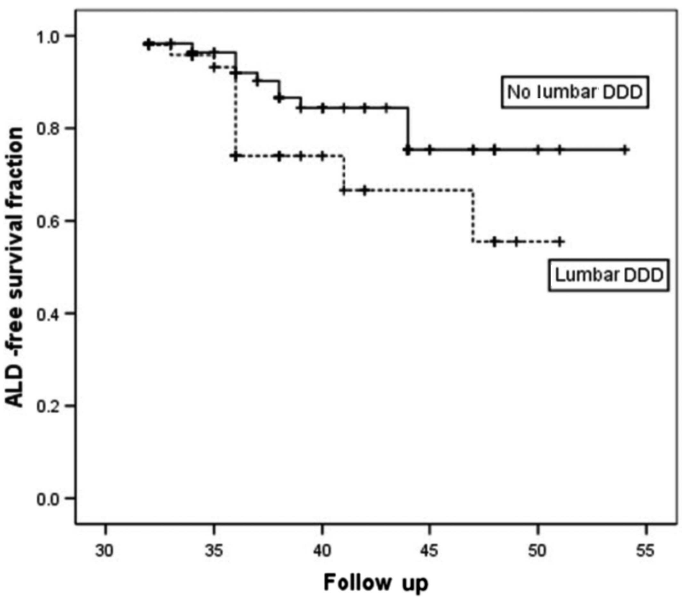


**Figure 5.** Kaplan-Meier survival plot for effect of concurrent lumbar spine degenerative disc disease (DDD) on the incidence of adjacent level disease (ALD).

## Miscellaneous Factors

As mentioned in the previous sections, the other factors that were considered to possibly influence the incidence of ALD in the patient population were increasing age (>50 years); sex; number of levels fused (1 *vs.* 2); smoking habit; and the affected levels at the index procedures. None of these factors showed any statistically significant effect on the period of freedom from adjacent level degeneration in patients undergoing anterior surgery for 1- and 2-level DDD of the cervical spine. Table 4 depicts the results of the Cox proportional hazard model that was run to predict adjusted hazard ratios (with 95% CI) for developing ASD in the current analysis.

## DISCUSSION

The problem of symptomatic ASD after anterior cervical surgery was first studied in detail by Hilibrand *et al*.[9] They assessed the symptomatic patients and classified them into 4 different categories according to the evidence of adjacent segment degeneration. Although their system continues to remain unchallenged and widely accepted, spine surgeons have increasingly utilized the MRI of the cervical spine in association with the clinical findings and electrophysiologic studies as adjuncts to clinical examination for establishing ASD. For the present analysis, therefore, we chose as diagnostic parameters for ALD clinical symptoms, MRI, and electrophysiological findings. In addition, to avoid the confounding bias, we considered only those patients who received active treatment for ALD during their follow-up periods. The treatment could be conservative or surgical. Regardless of the difference in the diagnostic parameters, the annual incidence of ALD for the present study was within the range described by Hilibrand *et al* for ACDF alone (0%–4.8%). Wigfield *et al*,[8] in their bench study, showed that artificial disc in the cervical spine resulted in reduced stresses in the annulus of the neighboring segments compared with



Copyright © 2012 Lippincott Williams & Wilkins. Unauthorized reproduction of this article is prohibited.

**TABLE 3. Comparison of Various Groups With Regard to Adjacent Level Disease (ALD)**

| Group | Incidence (%) | Annual Incidence (%) | 4-year ALD-Free Rate (%) | Actuarial ALD-Free Period (months) | 95% CI (months) |
|---|---|---|---|---|---|
| ACDF | 14 | 3.23 | 78.3 ± 0.8 | 46.04 ± 0.6 | 44.7–47.3 |
| TDA | 17 | 3.77 | 76.7 ± 0.5 | 48.1 ± 1.0 | 46.7–50.8 |
| Osteopenic | 24* | 5.88%* | 54.0 ± 1.0* | 42.08 ± 1.7* | 38.6–45.4* |
| Normal DEXA | 15 | 3.33% | 74.4 ± 0.5* | 49.8 ± 0.8 | 48.1–51.4 |
| Concurrent lumbar DDD | 23.5* | 5.4%* | 55.5 ± 1.2* | 45.7 ± 1.2* | 43.2–48.2* |
| Normal lumbar spine | 12.7 | 2.82% | 75.4 ± 0.6 | 50.3 ± 0.8 | 48.6–52.0 |

**Significant for $P < 0.05$.*

*ACDF, indicates anterior cervical fusion; TDA, total disc arthroplasty; DEXA, dual-energy x-ray absorptiometry; DDD, degenerative disc disease.*

spines with a simulated fusion. This led to the formulation of the theory that restriction of physiological motion at the fused segments causes predisposition to degeneration at the adjacent levels because of increased mechanical stresses.[20]

Hilibrand *et al*,[10] in a follow-up study to their original work, admitted that the scientific literature was unclear as to whether the ALD is a result of spinal fusion with iatrogenic motion restriction or whether it represents a progression of the natural history of degeneration. Regardless, the first decade of the 21st century saw introduction of several artificial cervical discs in the field of spine surgery. All of the published studies proved the safety and efficacy of these devices but have not claimed improved clinical outcomes for the patients when compared with ACDF.[21] In addition, no published data thus far provide or assess the incidence of ASD after the procedure. There are 2 possible reasons for the lack of literature. Firstly, most of these studies were done as prospective clinical trials assessing the safety and efficacy of the cervical disc replacement procedure. The primary end points in such trials are focused on improvements in patients' symptoms attributable to the index level. Secondly, the published results are mostly focused on the outcomes at 24-month follow-up, a period that is too short to assess ASD. In the current trials also, the investigators were mandated to record the occurrence of ASD, and the data were pooled as a part of general adverse event reporting to the FDA rather than assessing the incidence of and analyzing the influencing factors for ALD. We accept the fact that, considering the prevalent belief[20] and previously published studies,[8,14] it would have come as no surprise if we had found the incidence of ALD significantly lower in the subgroup receiving TDA as compared with those with ACDF. Nevertheless, the patients included in the analysis are the ones who had been randomized without bias and have been followed closely even after the 24-month period required by the protocols. The data thus collected are unbiased and well balanced to draw conclusions and/or make predictions.

## Shortcomings

FDA IDE trials have very stringent inclusion and exclusion criteria including but not limited to age, duration of the disease, absence of other concurrent medical conditions, and so on. A common criticism is that these patients do not necessarily represent the cross-section of the whole population. On the contrary, such trials also are the only sources to obtain truly randomized, carefully collected, and validated data for the purpose of analysis. Another shortcoming that we realize is the relatively shorter follow-up period of up to 52 months in the present study as compared with the 10-year data for the ACDF procedure.[9] Although we continue to collect longer-term data on these patients, we feel confident that our statistical methods are truly predictive of even the longer-term results as they will become available. We also acknowledge that the data were collected from only 2 collaborating institutions and cannot be truly labeled as

**TABLE 4. Cox Proportional Hazard Ratios for Miscellaneous Factors Considered to Impact ALD**

| Factor | Std. Error | Significance | Expected Hazard Ratio | 95.0% CI for Expected Hazard Ratio | |
|---|---|---|---|---|---|
| | | | | Lower | Upper |
| Age | 0.037 | 0.364 | 0.967 | 0.899 | 1.040 |
| Levels | 0.469 | 0.340 | 1.564 | 0.624 | 3.921 |
| Implant | 0.474 | 0.540 | 0.748 | 0.296 | 1.892 |
| Sex | 0.491 | 0.287 | 2.530 | 0.966 | 3.621 |
| Smoker | 0.491 | 0.826 | 1.114 | 0.425 | 2.916 |



Copyright © 2012 Lippincott Williams & Wilkins. Unauthorized reproduction of this article is prohibited.

"multi-institutional." We have already initiated an effort to collaborate with several other investigating institutions to possibly analyze longer-term results from a much larger patient pool with the cooperation of multiple investigating institutions.

## CONCLUSION

At a projected follow-up of up to 54 months, the risk of developing symptomatic ASD after anterior surgery for 1 or 2 levels of the cervical spine does not significantly vary between patients receiving TDA or anterior fusion. Other factors including bone mineral density and presence of concurrent lumbar degeneration have a more significant effect in the incidence of adjacent segment degeneration.

### ➢ Key Points

- ❑ The 3- and 4-year incidence of symptomatic ASD is similar after fusion or total disc replacement in cervical spine.
- ❑ Presence of osteopenia at the time of cervical surgery increases the risk of adjacent segment degeneration.
- ❑ Concurrent lumbar spine degeneration at the time of cervical surgery carries higher risk of adjacent segment degeneration.

### References

1. Caspar W, Barbier DD, Klara PM. Anterior cervical fusion and caspar plate stabilization for cervical trauma. *Neurosurgery* 1989;25:491–502.
2. Bohlman HH, Emery SE, Goodfellow DB, et al. Robinson anterior cervical diskektomy and arthrodesis for cervical radiculopathy. *J Bone Joint Surg Am* 1993;75:1298–1307.
3. Caspar W, Geisler FH, Pitzen T, et al. Anterior cervical plate stabilization in one and two level degenerative disease: overtreatment or benefit? *J Spinal Disord* 1998;11:1–11.
4. Eck JC, Humphreys SC, Hodges SD, et al. A comparison of outcomes of anterior cervical discectomy and fusion in patients with and without radicular symptoms. *J Surg Orthop Adv* Spring 2006; 15:24–6.
5. Gray MJ, Biyani A, Smith A. A retrospective analysis of patient perceived outcomes in patients 55 years and older undergoing anterior cervical discectomy and fusion. *J Spinal Disord Tech* 2010;23: 157–61.
6. Goofin J, Geusens E, Vantomme N, et al. Long term follow-up after interbody fusion of the cervical spine. *J Spinal Disord Tech* 2004;17:79–85.
7. Baba H, Furusawa N, Imura S, et al. Late radiographic findings after anterior cervical fusion for spondylotic myeloradiculopathy. *Spine* 1993;18:2167–73.
8. Wigfield CC, Skrzypiec D, Jackowski A, et al. Internal stress distribution in cervical intervertebral discs: the influence of an artificial joint and simulated anterior interbody fusion. *J Spinal Disord Tech* 2003;16:441–9.
9. Hilibrand AS, Carlson GD, Palumbo MA, et al. Radiculopathy and myelopathy at segments adjacent to the site of a previous anterior cervical arthrodesis. *J Bone Joint Surg Am* 1999;81:519–28.
10. Hilibrand AS, Robbins M. Adjacent segment degeneration and adjacent segment disease: the consequences of spinal fusion? *Spine J* 2004;4:190S–4S.
11. Cummins BH, Robertson JT, Gill SS. Surgical experience with an implanted artificial cervical joint. *J Neurosurg* 1998;88:943–8.
12. Bryan VE Jr. Cervical motion segment replacement. *Eur Spine J* 2002;11:S92–7.
13. Sekhon LH. Cervical arthroplasty in the management of spondylotic myelopathy. *J Spinal Disord Tech* 2003;16:307–13.
14. Wigfield CC, Gill SS, Nelson RJ, et al. The new Frenchay artificial cervical joint: results from a two-year pilot study. *Spine* 2002;27:2446–52.
15. Heller JG, Sasso RC, Papadopoulos SM, et al. Comparison of BRYAN cervical disc arthroplasty with anterior cervical decompression and fusion: clinical and radiographic results of a randomized, controlled, clinical trial. *Spine* 2009;34:101–7.
16. Sasso RC, Smucker JD, Hacker RJ, et al. Clinical outcomes of BRYAN cervical disc arthroplasty: a prospective, randomized, controlled, multicenter trial with 24-month follow-up. *J Spinal Disord Tech*. 2007;20:481–91.
17. Yang S, Wu X, Hu Y, et al. Early and intermediate follow-up results after treatment of degenerative disc disease with the Bryan cervical disc prosthesis: single- and multiple-level. *Spine* 2008;33:E371–7.
18. Riew KD, Buchowski JM, Sasso R, et al. Cervical disc arthroplasty compared with arthrodesis for the treatment of myelopathy. *J Bone Joint Surg Am* 2008;90:2354–64.
19. Garrido BJ, Taha TA, Sasso RC. Clinical outcomes of Bryan cervical disc arthroplasty: a prospective, randomized, controlled, single site trial with 48-month follow-up. *J Spinal Disord Tech* 2010;23: 367–71.
20. McAfee PC. The indications for lumbar and cervical disc replacement. *Spine J* 2004;4:177S–81S.
21. Bartels RH, Donk R, VerBeek AL. No justification for cervical disc prostheses in clinical practice: a meta-analysis of randomized controlled trials. *Neurosurgery* 2010;66:1153–60.



Copyright © 2012 Lippincott Williams & Wilkins. Unauthorized reproduction of this article is prohibited.
