# Supplementary material for: Adjacent segment degeneration or disease after cervical total disc replacement: a meta-analysis of randomized controlled trials
Source: J Orthop Surg Res. 2018 Oct 3;13:244. doi: 10.1186/s13018-018-0940-9 (PMC6169069; doi:10.1186/s13018-018-0940-9)
Supplement: Supplementary file 2 — File S1. Original data of 11 included articles. (ZIP 12 mb) [file 13018_2018_940_MOESM2_ESM.zip › 11 included articles and original data referred in this article/11 included articles/25 Wei, TIAN(CHA).pdf]

·临床论著·

Bryan 人工间盘置换与前路减压融合治疗颈椎退行性疾病的中期随访研究

田伟 阎凯 韩骁 于杰 靳培浩 韩晓光

【摘要】 目的 评价人工椎间盘置换术治疗颈椎退行性疾病的中期疗效，并探讨其是否可以减少邻近节段退变的发生。**方法** 前瞻性对比分析接受颈椎人工间盘置换术（置换组，45 例）与颈椎前路减压融合术（融合组，48 例）治疗的颈椎退行性疾病患者的随访 6 年临床疗效和影像学资料。临床疗效评价指标为日本骨科协会评分（Japanese Orthopaedic Association Scores，JOA）、颈椎功能残障指数量表（neck disability index，NDI）和 Odom 评分。影像学评价指标为矢状位曲度、活动度、邻近节段退变。**结果** 28 例置换组患者和 35 例融合组患者完成随访。两组患者末次随访的 JOA 评分和 NDI 均较术前有明显改善，组间比较差异无统计学意义。92.9%的置换组患者和 97.1%的融合组患者 Odom 评分获得很好或较好的结果。两组患者颈椎矢状位曲度末次随访较术前均得到保持。颈椎整体活动度置换组末次随访与术前无明显差异，而融合组则是明显降低。置换组置换节段活动度术前为 9.5°±3.7°，术后 3 个月为 7.0°±3.0°，末次随访为 6.6°±4.1°，末次随访较术后 3 个月无明显改变。邻近节段退变评估采用侧位 X 线片和 MRI T_2 加权像，置换组上、下邻近节段退变均明显少于融合组。**结论** Bryan 人工椎间盘置换术6 年的随访结果基本满意，能更好地保留颈椎生理活动及生物力学环境，从而降低邻近节段退变的发生率。

【关键词】 颈椎；椎间盘；假体植入；脊柱融合术；随访研究

【证据等级】 治疗性研究 Ⅱ 级

Comparison of the mid-term follow-up results between Bryan cervical artificial disc replacement and anterior cervical decompression and fusion for cervical degenerative disc disease *TIAN Wei, YAN Kai, HAN Xiao, YU Jie, JIN Pei-hao, HAN Xiao-guang. Department of Spine Surgery, Beijing Jishuitan Hospital, Beijing 100035, China*

【Abstract】 Objective To evaluate the mid-term follow-up results of cervical artificial disc replacement (CADR) for cervical degenerative disc disease, and to explore whether it can reduce the occ[illegible]ce of adjacent segment degeneration (ASD). **Methods** A prospective comparative study of 93 patients[illegible] underwent CADR or anterior cervical decompression and fusion (AC[illegible]r cervical degenerative disc disease were conducted. All patients were followed up for more than 6 year[illegible]e Japanese Orthopaedic Association (JOA) score, neck disability index (NDI), Odom′s scale, X-rays and magnetic resonance imaging (MRI) were used to evaluate the clinical and radiologic results. **Results** Twenty eight patients who underwent CADR and 35 patients who underwent ACDF had complete follow-up data. At final follow-up, the JOA score and NDI improved significantly in both groups. Between the two groups, there was no significant difference in terms of JOA score, NDI and Odom′s scale. The sagittal alignment was well maintained in both groups. The total cervical spine range of motion (ROM) had no significant change for the CADR group, whereas, it significantly decreased for the ACDF group. The ROM at the replacement level of CADR patients decreased from 9.5° ± 3.7° before operation to 7.0° ± 3.0° 3 months after operation, and it was maintained to 6.6° ± 4.1° at final follow-up without significant decrease. Lateral radiographs and T_2-weighted MRI showed the incidence of ASD in CADR group was significantly lower than that in ACDF group. **Conclusion** The six-year follow-up results of CADR are basically satisfactory. Compared with ACDF, it could better preserve physiological motion and biomechanics of cervical spine, and reduce the incidence of ASD.

【Key words】 Cervical vertebrae; Intervertebral disk; Prosthesis implantation; Spinal fusion; Follow-up studies

近 50 年来，颈椎前路减压融合术已成为治疗颈椎退行性疾病的金标准。然而，随着随访时间的延长以及病例的积累，很多学者提出邻近节段退变的问题[1-3]。Hilibrand 等[2]回顾性分析 374 例颈椎前路减压融合术后患者的资料，发现每年大约有 2.9%的患者会出现伴有相关症状的邻近节段退变，10 年的累积发生率为 25%。但邻近节段退变究竟是融合造成的还是自然病程的结果，至今仍有很大争议。

DOI：10.3760/cma.j.issn.0253-2352.2013.02.001

作者单位：100035 北京积水潭医院脊柱外科

万方数据

颈椎人工椎间盘置换术设计的理论基础是其能保留颈椎的生理活动及生物力学环境，进而可以减少邻近节段退变的发生以及避免融合术相关的其他并发症。目前，颈椎人工椎间盘置换术已在脊柱外科领域中广泛应用，尽管其短期疗效已被认可[4-6]，但中、远期疗效还有待观察。

本研究采用前瞻性对比随访研究的方法，研究对象为颈椎退行性疾病并有脊髓压迫症表现的患者（颈椎间盘突出症或退行性颈椎管狭窄症），对接受 Bryan 颈椎人工椎间盘置换术和颈椎前路减压融合术患者的术前和术后 6 年以上（随访时间超过 68 个月）的随访资料进行对比研究，比较此两种术式的临床效果及影像学结果，目的在于：(1)评价颈椎人工椎间盘置换术治疗颈椎退行性疾病的中期疗效，(2)探讨颈椎人工椎间盘置换术是否降低邻近节段退变的发生率。

资 料 与 方 法

一、临床资料

（一）纳入标准及排除标准

纳入标准：(1)脊髓型颈椎间盘突出症；(2)脊髓型退行性颈椎管狭窄症；(3)至少经过 3 个月的保守治疗；(4)手术节段≤3 个，且适合颈椎前路手术。

排除标准：(1)严重的椎间关节退变（桥状骨赘、椎间隙高度丢失>50%、椎间隙活动度<2°）；(2)小关节骨关节炎；(3)发育性颈椎管狭窄；(4)后纵韧带骨化；(5)明显的颈椎不稳定；(6)骨质疏松症；(7)颈椎畸形；(8)颈椎肿瘤；(9)感染；(10)骨关节炎性疾病（如类风湿性关节炎、强直性脊柱炎）；(11)既往有颈椎手术史。

本研究取得医院伦理委员会的批准[illegible]两组患者的入选并非随机，所有患者均适合采用颈椎人工椎间盘置换术和颈椎前路减压融合术，但考虑到相关的伦理问题及患者的经济能力，最终术式的选择于术前谈话时向患者充分阐释后由患者决定。

（二）一般资料

2003 年 12 月至 2005 年 12 月，共 93 例患者入组接受 Bryan (M[illegible]ronic Sofamor Danek 公司，美国)颈椎人工椎间盘置换手术（置换组，45 例[illegible]31 例、女 14 例）或颈椎前路减压融合手术（融合组，48 例，男 35 例，女 13 例）。其中置换组 28 例患者和融合组 35 例患者完成随访并获得完整的影像学资料，随访率为 67.7%。

置换组 28 例，男 19 例，女 9 例；年龄 2[illegible]70 岁，平均 45.0 岁。单节段者 20 例，双节段者 7 例，三节段者 1 例。中位随访时间为 80.5 个月（68~90 个月）。手术节段分布 $C_{3,4}$ 2 例，$C_{4,5}$ 8 例，$C_{5,6}$ 22 例，$C_{6,7}$ 5 例。

融合组 35 例，男 25 例，女 10 例；年龄 32~69 岁，平均 48.7 岁。单节段者 23 例，双节段者 12 例[illegible]中位随访时间为 77.0 个月（68~100 个月），手术节段分布 $C_{3,4}$ 8 例，$C_{4,5}$ 9 例，$C_{5,6}$ 22 例，$C_{6,7}$ 8 例。

两组患者的人口学资料及基线资料的差异均无统计学意义（表 1），两组具有可比性。所有手术均由同一术者完成（本文第一作者）。

二、手术方法与术后处理

（一）颈椎人工椎间盘置换术

采用全身麻醉，患者取仰卧位，头部中立位颈颌吊带牵引。X 线透视测量置换间隙与铅垂线的夹角后，按照 Bryan 颈椎人工间盘假体的标准操作规程选择入路。首先切除椎间盘，两侧到达钩椎关节，深部尽可能接近椎体后缘；使用椎间撑开器撑开椎间隙；安装定位器与导轨，使试模与椎间隙平行，在“C”型臂 X 线机透视下仔细测量椎间隙前缘和后缘的距离以确定假体型号；继续在 X 线透视下用磨钻制备上、下椎体的母床；按照经测量确定的型号选择相应的人工椎间盘凹形窝磨削钻，X 线透视下进行适当的磨削，制备上、下椎体的凹形窝；拆除导向器后，用椎体撑开器适当撑开椎间隙，仔细切除椎体后缘的椎间盘组织和骨赘，必要时切除后纵韧带，充分

表 1　颈椎人工椎间盘置换组与颈椎前路减压融合组患者的人口学资料及基线资料

组别	节段分布（单/双/三，例）	性别（男/女，例）	年龄［$\bar{x}\pm s$（范围），岁］	随访时间［中位数（范围），月］	诊断（例） 颈椎间盘突出症	诊断（例） 退行性颈椎管狭窄	手术节段（例） $C_{3,4}$	手术节段（例） $C_{4,5}$	手术节段（例） $C_{5,6}$	手术节段（例） $C_{6,7}$
置换组（28 例）	20/ 7/1	19/9	45.0±9.5（25~70）	80.5(68~94)	13	15	2	8	22	5
融合组（35 例）	23/12/0	25/10	48.7±10.8（32~69）	77.0(68~100)	19	16	8	9	22	8
统计值	P=0.408[a]	χ^2=0.094，P=0.759[b]	t=−1.423，P=0.160[c]	Z=−0.693，P=0.488[d]	χ^2=0.384，P=0.535[b]		P=0.382[a]			

注：[a] 双尾 Fisher 精确概率法；[b] 双尾卡方检验；[c] 独立样本 t 检验；[d] 秩和检验(Mann-Whitney U test)

万方数据

减压。仔细止血和冲洗后，按照确定的型号植入人工椎间盘。正、侧位 X 线透视确认假体位置良好后逐层缝合。术后颈托制动 2 周，术后第 2 天下地活动。

(二)颈椎前路减压融合术

常规颈前路入路，切除椎间盘，椎体间撑开器适当撑开椎间隙，仔细切除椎体后缘的椎间盘组织、骨赘及后纵韧带，充分减压。取相应大小修整后的珊瑚人工骨置入椎间隙，"C" 型臂 X 线机透视确认位置满意后，选择合适的钛板及螺钉固定，逐层缝合。术后颈托制动 3 个月，术后第 2 天下地活动。

三、随访及疗效评价

所有患者均于术后 3、6、12 个月以及以后每 6 个月进行门诊随访。临床疗效及影像学评估的时间点为术前、术后第 3 天、术后 3 个月和末次随访(术后超过 68 个月)，患者均于我院完成临床疗效评估和影像学检查。

(一)临床疗效评价

临床疗效评价采用日本骨科协会评分[Japanese Orthopaedic Association Scores，JOA(17 分法，1994 年修订版)][7-8]、颈椎功能残障指数量表(neck disability index，NDI)[9-10]和 Odom 评分[11]，评估时间为术前和末次随访。

JOA 评分包含对主观症状、神经体征和生活能力的评估，主要用于评价患者颈髓症方面的神经功能障碍，患者神经功能改善情况采用 JOA 评分改善率表达。

$$\text{JOA 评分改善率}=\frac{\text{术后评分}-\text{术前评分}}{17-\text{术前评分}}\times 100\%$$

中文版 NDI 为问卷形式，包含 10 项问题用来评估颈痛及功能障碍 (2 项关于疼痛、1 项关于注意力、7 项关于生活能力)，指数的计算结果为所得分数占满分的百分比，主要用以评价患者的生活功能障碍。

Odom 评分结果分为：(1)很好，患者无任何有关颈椎疾病的不适，日常工作不受限；(2)较好，患者存在间断性有关颈椎疾病的不适，对日常工作无明显影响；(3)一般，患者症状有改善，但日常生活明显受限；(4)差，患者与术前相比症状无改善甚至加重。Odom 评分能很好地反映患者对于手术的满意程度，得以评价整体的疗效。

(二)影像学评价

影像学评价的资料包括颈椎中立位侧位 X 线片，过屈位、过伸位 X 线片及和 MRI。X 线片的检查时间为术前、术后 3 天、术后 3 个月和末次随访，MRI 检查的时间为术前和末次随访。影像学测量通过 PACS 工作站(Rogan-Delft，Netherlands)完成。每个测量结果均为两名医生独立测量的结果取平均值。

评价指标包括：矢状位曲度(C_2~C_7 角、间盘角和脊柱功能单位角)、颈椎整体活动度(C_2~C_7)、置换组患者置换节段活动度以及邻近节段活动度。

邻近节段退变是通过侧位 X 线片和 MRI T_2 加权像来评估。邻近节段退变的 X 线表现为椎体前缘骨赘形成、骨赘增大、椎间隙变窄(>30%)和前纵韧带钙化[12]。邻近节段退变的 MRI 表现为新发的椎间盘突出和椎间盘信号改变 (采用颈椎间盘退变的 Miyazaki 分级[13])。所有用来评估邻近节段退变的 X 线片和 MRI 都经过处理以隐去手术节段，即对评估者使用盲法，共有 3 名医生分别独立地评估邻近节段退变。

四、统计分析

采用 SPSS 19.0(SPSS 公司，美国)统计软件包进行统计分析，符合正态分布的连续变量组间比较使用 *t* 检验，组内比较使用配对 *t* 检验；分类变量的比较使用卡方检验或 Fisher 精确概率法；等级变量或者不符合正态分布的连续变量组间比较使用秩和检验(Mann-Whitney *U* test)；组内比较使用配对秩和检验 (Wilcoxon signed-rank test)，检验水准 α=0.05。

结　果

一、临床疗效评价结果

置换组与融合组患者末次随访时的 JOA 评分和 NDI 均较术前有明显改善，92.9%的置换组患者和 97.1%的融合组患者 Odom 评分获得很好或较好的结果。两组 JOA 评分、JOA 评分改善率和 Odom 评分的差异均无统计学意义(表 2)。两组患者均未出现感染、食管损伤、血肿、假体移位及神经功能损害等并发症，两组均无再次手术者。

二、影像学评价结果

(一)矢状位曲度

矢状位曲度是由中立位侧位 X 线片测量得出，C_2~C_7 角为 C_2 和 C_7 椎体下终板所形成的夹角，间盘角为目标椎间隙上、下终板形成的夹角，脊柱功能单位角(functional spinal unit angle)为上位椎体的上终板和下位椎体的下终板形成的夹角(图 1)。角度为正表示前凸，为负表示后凸。

两组术前的颈椎整体前凸(C_2~C_7 角)与局部前凸(间盘角和脊柱功能单位角)的差异均无统计学意义

万方数据

义(P >0.05,表 3)。末次随访时,融合组的整体前凸与局部前凸均明显大于置换组(P< 0.05,表 3)。置换组患者末次随访时的整体前凸与局部前凸基本保持术前的水平（略有减小，但差异无统计学意义,P > 0.05,表 3)。而融合组患者末次随访时的整体前凸与局部前凸较术前均有增大，其中脊柱功能单位角的差异有统计学意义(P< 0.05,表 3)。

(二)活动度

活动度是由过屈位与过伸位 X 线片测量得出，过屈位与过伸位 C_2~C_7 角度的变化即为颈椎整体活动度。

两组术前的颈椎整体活动度的差异无统计学意义(P=0.279,表 4),然而在末次随访时,置换组患者的整体活动度明显大于融合组(P< 0.001,表 4)。置换组患者末次随访时的颈椎整体活动度与术前相比差异无统计学意义(P=0.477,表 4);融合组患者末次随访时的颈椎整体活动度较术前有明显减小（P< 0.001,表 4),单节段融合者整体活动度减小 15.8%,

表 2　颈椎人工椎间盘置换组与颈椎前路减压融合组患者的临床疗效

组别	JOA 评分[中位数(四分位间距)]			NDI[中位数(四分位间距),%]		Odom 评分[例(%)]			
	术前(分)	末次随访(分)	改善率(%)	术前	末次随访	很好	较好	一般	差
置换组(28 例)	14.3(4.4)	16.5(0.9)	75.0(36.4)	28.4(18.3)	8.0(14.3)	12(42.9)	14(50.0)	2(7.1)	0
融合组(35 例)	13.0(4.0)	16.5(1.0)	93.8(28.6)	30.0(12.0)	8.9(8.9)	19(54.3)	15(42.9)	1(2.9)	0
统计值[a]	Z=−1.825<br>P=0.068	Z=−1.314<br>P=0.189	Z=−1.937<br>P=0.053	Z=−1.034<br>P=0.301	Z=−0.423<br>P=0.672	Z=−1.000,P=0.317			

注:[a] 秩和检验(Mann-Whitney U test)

表 3　颈椎人工椎间盘置换组与颈椎前路减压融合组患者的矢状位曲度($\bar{x}\pm s$,°)

组别		C_2~C_7 角	间盘角	脊柱功能单位角
置换组(28 例)	术前	11.0±12.6	1.0±3.6	2.0±4.9
	末次随访	10.5±10.3	1.0±4.7	0.6±6.8
融合组(35 例)	术前	15.0±11.9	2.5±4.7	3.5±6.6
	末次随访	18.8± 8.5	3.1±2.7	6.0±5.9
统计值	两组术前比较[a]	t=−1.284, P=0.204	t=−1.624, P=0.108	t=−1.193, P=0.236
	两组末次随访比较[a]	t=−3.517, P<0.001	t=−2.433, P=0.018	t=−3.895, P<0.001
	置换组内比较[b]	t= 0.261,P=0.796	t=−0.045,P=0.965	t= 1.408,P=0.168
	融合组内比较[b]	t=−1.869,P=0.070	t=−0.881,P=0.383	t=−2.347,P=0.023

注:[a] 组间比较采用 t 检验;[b] 组内采用配对 t 检验

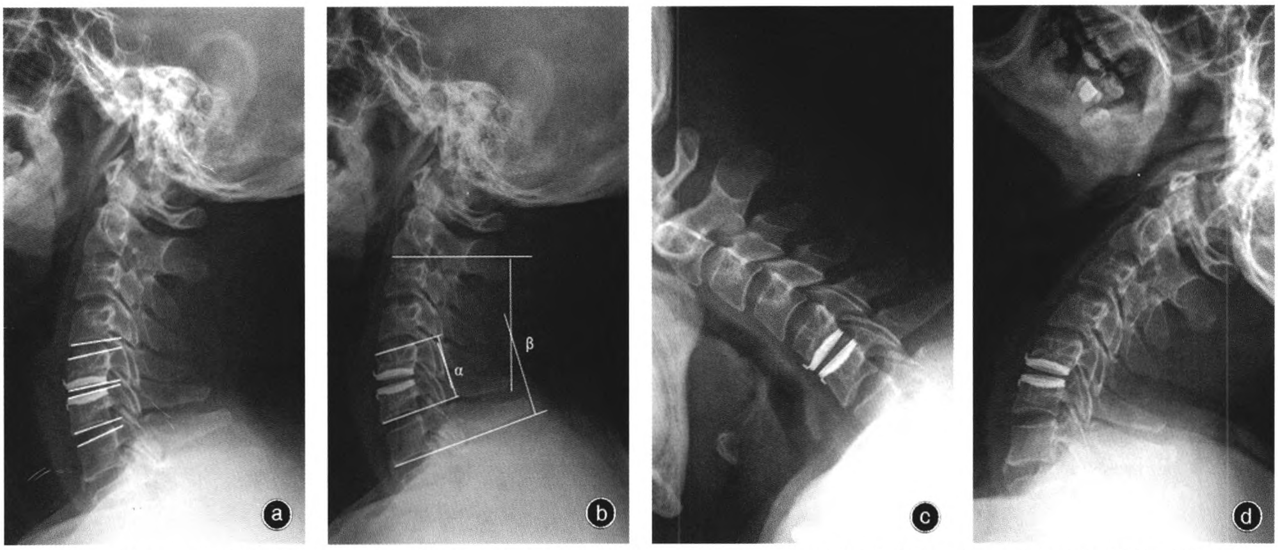

图 1　Bryan 颈椎人工间盘置换术后矢状位曲度和颈椎整体活动度的测量方法　a　中立位侧位 X 线片,间盘角为目标椎间隙上、下终板形成的夹角　b　中立位侧位 X 线片,脊柱功能单位角为上位椎体的上终板和下位椎体的下终板形成的夹角(α),C_2~C_7 角为 C_2 和 C_7 椎体下终板所形成的夹角(β)　c,d　过屈位和过伸位侧位 X 线片,C_2~C_7 角度的变化即为颈椎整体活动度

万方数据

双节段者减小 35.6%。置换组患者置换节段的活动度术前为 9.5°±3.7°，术后 3 个月为 7.0°±3.0°，末次随访为 6.6°±4.1°，末次随访较术后 3 个月无明显降低（t=–0.768，P=0.448）。末次随访时有 4 个人工椎间盘置换节段（10.8%，4/37）的活动度< 2°。融合组所有患者的手术节段均完全骨性融合。两组患者邻近节段活动度术前与末次随访时的差异均无统计学意义（表 4）。

表 4 颈椎人工椎间盘置换组与颈椎前路减压融合组患者的颈椎整体活动度和邻近节段活动度（$\bar{x}\pm s$，°）

组别		颈椎整体活动度	上邻近节段活动度	下邻近节段活动度
置换组（28 例）	术前	43.2±15.8	8.9±3.5	9.2±3.7
	末次随访	45.0±10.4	7.6±3.8	8.3±3.5
融合组（35 例）	术前	47.5±13.7	11.2±5.0	9.9±5.3
	末次随访	34.2± 9.3	11.4±6.4	10.2±4.6
统计值	两组术前比较[a]	t=–1.094，P=0.279	t=–2.028，P=0.048	t=–0.505，P=0.616
	两组末次随访比较[a]	t= 4.185，P<0.001	t=–2.715，P=0.009	t=–1.629，P=0.110
	置换组内比较[b]	t=–0.721，P=0.477	t= 1.379，P=0.179	t= 1.226，P=0.231
	融合组内比较[b]	t= 5.193，P< 0.001	t=–0.120，P=0.905	t=–0.324，P=0.749

注：[a] 组间比较采用 t 检验；[b] 组内采用配对 t 检验

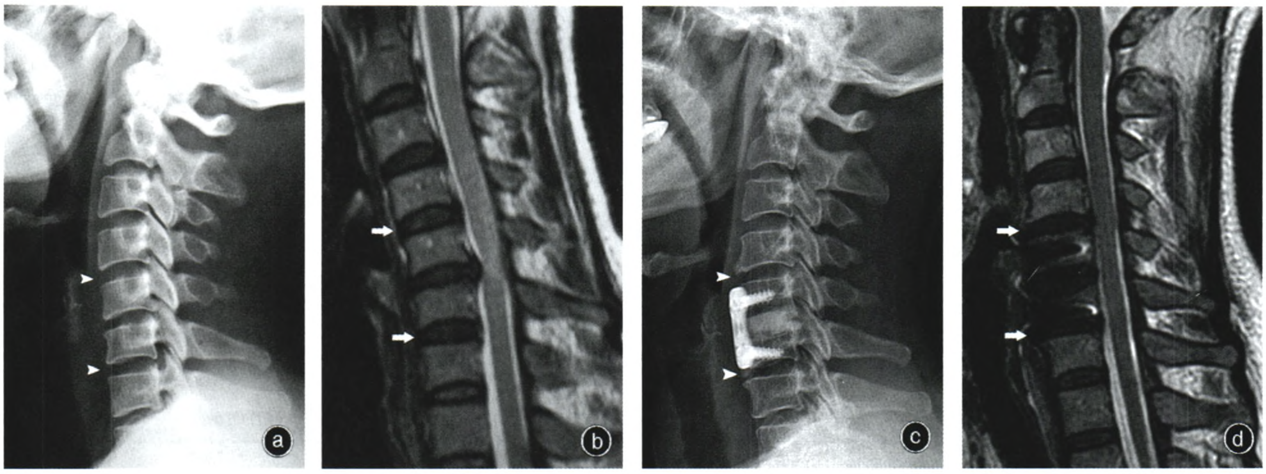

图 2 女，44 岁，$C_{5,6}$ 椎间盘突出症 a 术前侧位 X 线片示邻近节段并无退变（箭头所示） b 术前正中矢状位 MRI T_2 加权相像示邻近节段间盘信号为 Miyazaki Ⅲ度（箭头所示） c 颈椎前路减压融合术后 76 个月，侧位 X 线片示邻近节段退变（箭头所示） d 术后 76 个月，正中矢状位 MRI T_2 加权相像示邻近节段间盘信号加重为 Miyazaki Ⅳ度（箭头所示）

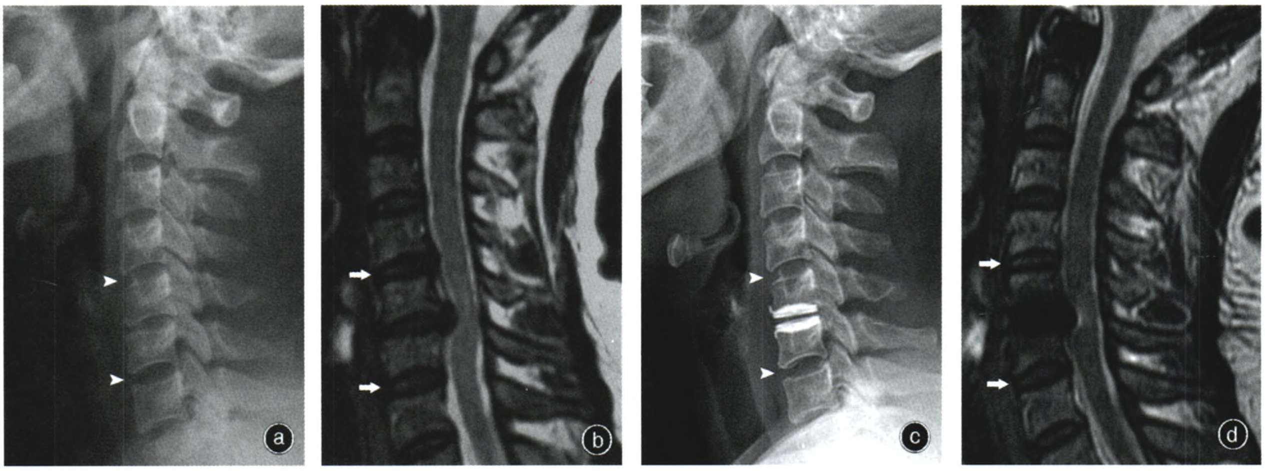

图 3 女，47 岁，$C_{5,6}$ 椎间盘突出症 a 术前侧位 X 线片示邻近节段术前并无退变（箭头所示） b 术前正中矢状位 MRI T_2 加权相像示邻近节段间盘信号为 Miyazaki Ⅲ度（箭头所示） c Bryan 颈椎人工间盘置换术后 73 个月，侧位 X 线片未发现邻近节段退变（箭头所示） d 术后 73 个月，正中矢状位 MRI T_2 加权相像示邻近节段间盘信号仍为 Miyazaki Ⅲ度（箭头所示）

万方数据

（三）邻近节段退变

本研究采用颈椎侧位 X 线片和 MRI T_2 加权像评估邻近节段退变（图 2,3）。在上邻近节段，置换组中 3 例([illegible]7%,3/28)在 X 线片表现邻近节段退变，3 例[illegible].7%,3/28)在 MRI 表现邻近节段退变；而融合组中 20 例(57.1%,20/35)在 X 线片表现邻近节段退变，17 例(48.6%,17/35)在 MRI 表现邻近节段退变。下邻近节段，置换组中有 6 例[illegible].4%,6/28)在 X 线片表现邻近节段退变，3 例 (10.7%,3/28)在 MRI 表现邻近节段退变；而融合组中有 21 例(60.0%,21/35) 在 X 线片表现邻近节段退变，18 例(51.5%,18/35)在 MRI 表现邻近节段退变。置换组患者的邻近节段退变的发生率明显低于融合组(P<0.01,表 5,6)。

讨　论

一、人工椎间盘置换术的中期临床疗效

本研究结果显示人工椎间盘置换术的临床疗效满意，在神经功能改善及生活质量提高方面，与颈椎前路减压融合术的差异无统计学意义。文献报道（临床随机对照研究）均显示人工椎间盘置换术的中、短期疗效不次于颈椎前路减压融合术[4,6,14-16]，这些结果与本研究结果相似，说明神经功能恢复主要与术中减压是否彻底有关，而与术式选择关系不大。

二、矢状位曲度

文献报道表明颈椎的矢状位曲度对颈痛、脊髓压迫症及邻近节段退变有着十分重要的影响[17-18]。Pickett 等[19]和 Johnson 等[20]报告 Bryan 人工椎间盘置换术后置换节段会出现局部后凸的现象。在本研究中 Bryan 人工椎间盘置换术后置换节段的局部前凸略有减小，但差异无统计学意义，而颈椎前路减压融合术后局部前凸则增大。与颈椎前路减压融合术相比，Bryan 人工椎间盘置换没有恢复、重建置换节段局部前凸的作用，然而，如果在椎间盘置换手术中

表 5　颈椎人工椎间盘置换组与颈椎前路减压融合组患者 X 线片的邻近节段退变总结(例,%)

组别		新发骨赘形成	骨赘增大	椎间隙变窄(>30%)	前纵韧带新发钙化	前纵韧带钙化增大	邻近节段退变(左侧数据汇总)
置换组(28 例)	上邻近节段	3(10.7)	0	0	0	1(3.6)	3(10.7)
	下邻近节段	0	5(17.9)	0	1(3.6%)	0	6(21.4)
融合组(35 例)	上邻近节段	14(40.0)	6(17.1)	1(2.9)	1(2.9%)	2(5.7)	20(57.1)
	下邻近节段	9(25.7)	4(11.4)	2(5.7)	4(11.4%)	7(20.0)	21(60.0)
统计值	上邻近节段组间比较	χ^2=6.771,P=0.009[a]	P=0.029[b]	P=1.000[b]	P=1.000[b]	P=1.000[b]	χ^2=14.466,P< 0.001[a]
	下邻近节段组间比较	P=0.003[b]	χ^2=0.131,P=0.717[a]	P=0.498[b]	P=0.371[b]	P=0.014[b]	χ^2= 9.450,P=0.002[a]

注：[a] 双尾卡方检验；[b] 双尾 Fisher 精确概率法

表 6　颈椎人工椎间盘置换组与颈椎前路减压融合组患者 MRI T_2 加权像的邻近节段退变总结(例,%)

组别			颈椎间盘退变的 Miyazaki 分级					椎间盘 MRI 信号加重(Miyazaki 分级)	新发椎间盘突出	邻近节段退变(左侧数据汇总)
			Ⅰ度	Ⅱ度	Ⅲ度	Ⅳ度	Ⅴ度			
置换组(28 例)	上邻近节段	术前	0	1	16	11	0	1 (3.6)	2 (7.1)	3 (10.7)
		末次随访	0	1	15	12	0			
	下邻近节段	术前	0	2	13	13	0	2 (7.1)	1 (3.6)	3 (10.7)
		末次随访	0	2	11	15	0			
融合组(35 例)	上邻近节段	术前	0	2	18	14	1	13(37.1)	8 (22.9)	17 (48.6)
		末次随访	0	0	9	24	2			
	下邻近节段	术前	1	3	22	7	2	18(51.4)	1 (2.9)	18 (51.5)
		末次随访	0	1	10	22	2			
统计值	上邻近节段组间比较		Z=−2.708, P=0.007[a]					χ^2=10.143 P=0.001[b]	χ^2=1.820 P=0.177[b]	χ^2=10.289 P=0.001[b]
	下邻近节段组间比较		Z=−1.475, P=0.140[a]					χ^2=14.080 P< 0.001[b]	P=1.000[c]	χ^2=11.604 P=0.001[b]

注：[a] 秩和检验(Mann-Whitney U test)；[b] 双尾卡方检验(Two-sided Chi-Square test)；[c] 双尾 Fisher 精确概率法(two-sided fisher's exact test)

万方数据

采用改良的手术技术，注意以下问题：(1)手术体位要保持为中立位，避免过伸；(2)充分切除上位椎体前下缘骨赘，避免椎间撑开器置入椎间隙后造成前凸加大；(3)X 线透视下仔细测量椎体前缘与后缘的距离以确定假体型号；(4)假体置入方向应保证与椎间隙平行；(5) 椎体凹形窝磨削时不应用力过大，避免过度磨削，尤其避免对背侧终板的过度磨削，Bryan 人工椎间盘置换完全可以保持局部前凸[21-23]。

三、活动度

置换节段的远期活动度对于人工椎间盘置换术来说至关重要。人工椎间盘置换自 2000 年开始在临床广泛应用，目前国外已有 8 年以上的随访报告，临床效果及颈椎活动良好[24-25]。Quan 等[24]报告了Bryan 人工椎间盘在体内 8 年后 78%的患者仍保持着良好的活动度。在本研究中人工椎间盘置换节段的活动度术后较术前平均减小 2.4°，之后这样的活动度基本保持到术后 6 年。在 37 个置换节段中有 4 个节段末次随访时发现活动度< 2°。经仔细阅读术后影像学资料，我们发现 1 例出现后纵韧带骨化症，2 例出现钩椎关节融合和 1 例出现异位骨化（Ⅳ度）[26]。因此，对人工椎间盘置换患者的选择须非常仔细[27]，后纵韧带骨化症具有融合的趋势，应该被认为是人工椎间盘置换术的绝对禁忌证，但早期的后纵韧带骨化症有时很难诊断。钩椎关节融合的原因，我们考虑可能是术中刮出软骨时损害了外侧的钩椎关节面，因此，术中保护钩椎关节的完整性非常重要。在髋、膝关节置换手术中异位骨化是一种很常见的并发症[28-29]。在本研究中有 53.6%的人工椎间盘置换患者出现异位骨化。尽管异位骨化发生率较高，但明显影响局部活动度的Ⅲ度、Ⅳ度异位骨化并不多见，而且异位骨化的发生似乎并不影响临床疗效[30-31]。异位骨化的临床意义还有待于进一步的研究。

颈部僵硬感及活动受限是颈椎前路减压融合术后患者最常见的不适。在文研究中有 20%的融合组患者术后有明显的颈部僵硬感，单节段者颈椎整体活动度减小 15.8%，双节段减小 35.6%。而本中期随访结果也显示，只要病例选择正确，颈椎人工椎间盘置换可以很好地解决此问题。

四、邻近节段退变

尸体的生物力学研究已经证实人工椎间盘置换可以保留颈椎正常生理的生物力学性质。Cunningham 等[32]发现颈椎人工椎间盘置换后，手术节段与邻近节段的生物力学性质与术前相近，而颈椎前路减压融合术后则会发生很大的变化。Dmitriev 等[33]的研究表明颈椎人工椎间盘置换后邻近节段的椎间盘内压力和动力学特性均与术前接近。然而遗憾的是，关于人工椎间盘置换是否可以减少邻近节段退变，目前所有的临床研究尚未达成一致。Robertson 等[12]的临床研究认为保留活动度的椎间盘置换相对于融合术可以明显减少邻近节段退变的症状表现及影像学表现。而 Nunley 等[34]的临床研究则显示椎间盘置换术与融合术后 38 个月，邻近节段退变的发生率相同。在本研究中根据侧位 X 线片及 MRI T_2 加权像，人工椎间盘置换术相对于颈椎前路减压融合术有着更低的邻近节段退变发生率。不同的研究结论不同，甚至截然相反，究其原因我们认为有以下两点：(1)人工椎间盘置换作为一个相对较新的技术，病例选择不当及手术操作不规范，均会导致置换节段活动度的丧失，如此人工椎间盘置换术对于邻近节段的影响与颈椎前路减压融合术已无差别；(2)导致邻近节段退变的因素复杂多样，包括邻近节段的活动度、手术的节段以及矢状位曲度等，而各个因素间存在相互影响并且某一因素常与其有相反作用的因素共存，当各因素所占权重不同时就可能得到完全相反的结果。因此，我们还需要进一步更加细化的研究，对上述各种因素加以控制，避免其相互干扰。

两组病例的选择并非随机，入组的患者均适合采用这两种术式，最终术式的选择是由患者决定。非随机入组势必会造成一定的选择偏倚，但两组患者的人口学资料以及基线资料差异均无统计学意义，可见两组间仍具有较好的可比性。此外，由于中国人口流动性较大的特点，很多患者失访，导致本研究的随访率偏低(仅为 67.7%)。

综上所述，Bryan 颈椎人工椎间盘置换术能够维持矢状位曲度并保留置换节段的活动度，6 年随访的临床效果和影像学结果基本满意，能更好地保留颈椎的生理活动及生物力学环境，从而减少邻近节段退变的发生。

参 考 文 献

[1] Baba H, Furusawa N, Imura S, et al. Late radiographic findings after anterior cervical fusion for spondylotic myeloradiculopathy. Spine (Phila Pa 1976), 1993, 18(15): 2167-2173.

[2] Hilibrand AS, Carlson GD, Palumbo MA, et al. Radiculopathy and myelopathy at segments adjacent to the site of a previous anterior cervical arthrodesis. J Bone Joint Surg Am, 1999, 81(4): 519-528.

[3] Goffin J, Geusens E, Vantomme N, et al. Long-term follow-up after interbody fusion of the cervical spine. J Spinal Disord Tech, 2004, 17(2): 79-85.

万方数据

[4] Heller JG, Sasso RC, Papadopoulos SM, et al. Comparison of BRYAN cervical disc arthroplasty with anterior cervical decompression and fusion: clinical and radiographic results of a randomized, controlled, clinical trial. Spine (Phila Pa 1976), 2009, 34(2): 101-107.
[5] McAfee PC, Reah C, Gilder K, et al. A meta-analysis of comparative outcomes following cervical arthroplasty or anterior cervical fusion: results from 4 prospective multicenter randomized clinical trials and up to 1226 patients. Spine (Phila Pa 1976), 2012, 37 (11): 943-952.
[6] Zhang X, Zhang X, Chen C, et al. Randomized, controlled, multicenter, clinical trial comparing BRYAN cervical disc arthroplasty with anterior cervical decompression and fusion in China. Spine (Phila Pa 1976), 2012, 37(6): 433-438.
[7] Yonenobu K, Abumi K, Nagata K, et al. Interobserver and intraobserver reliability of the japanese orthopaedic association scoring system for evaluation of cervical compression myelopathy. Spine (Phila Pa 1976), 2001, 26(17): 1890-1894.
[8] Fukui M, Chiba K, Kawakami M, et al. An outcome measure for patients with cervical myelopathy: Japanese Orthopaedic Association Cervical Myelopathy Evaluation Questionnaire (JOACMEQ): Part 1. J Orthop Sci, 2007, 12(3): 227-240.
[9] Vernon H, Mior S. The Neck Disability Index: a study of reliability and validity. J Manipulative Physiol Ther, 1991, 14 (7): 409-415.
[10] Wu S, Ma C, Mai M, et al. Translation and validation study of Chinese versions of the neck disability index and the neck pain and disability scale. Spine (Phila Pa 1976), 2010, 35(16): 1575-1579.
[11] Odom GL, Finney W, Woodhall B. Cervical disk lesions. J Am Med Assoc, 1958, 166(1): 23-28.
[12] Robertson JT, Papadopoulos SM, Traynelis VC. Assessment of adjacent-segment disease in patients treated with cervical fusion or arthroplasty: a prospective 2-year study. J Neurosurg Spine, 2005, 3(6): 417-423.
[13] Miyazaki M, Hong SW, Yoon SH, et al. Reliability of a magnetic resonance imaging-based grading system for cervical intervertebral disc degeneration. J Spinal Disord Tech, 2008, 21 (4): 288-292.
[14] Sasso RC, Anderson PA, Riew KD, et al. Results of cervical arthroplasty compared with anterior discectomy and fusion: four-year clinical outcomes in a prospective, randomized controlled trial. J Bone Joint Surg Am, 2011, 93(18): 1684-1692.
[15] Nabhan A, Ahlhelm F, Pitzen T, et al. Disc replacement using Pro-Disc C versus fusion: a prospective randomised and controlled radiographic and clinical study. Eur Spine J, 2007, 16(3): 423-430.
[16] Murrey D, Janssen M, Delamarter R, et al. Results of the prospective, randomized, controlled multicenter Food and Drug Administration investigational device exemption study of the ProDisc-C total disc replacement versus anterior discectomy and fusion for the treatment of 1-level symptomatic cervical disc disease. Spine J, 2009, 9(4): 275-286.
[17] Katsuura A, Hukuda S, Saruhashi Y, et al. Kyphotic malalignment after anterior cervical fusion is one of the factors promoting the degenerative process in adjacent intervertebral levels. Eur Spine J, 2001, 10(4): 320-324.
[18] Ferch RD, Shad A, Cadoux-Hudson TA, et al. Anterior correction of cervical kyphotic deformity: effects on myelopathy, neck pain, and sagittal alignment. J Neurosurg, 2004, 100 (1 suppl Spine): 13-19.
[19] Pickett GE, Mitsis DK, Sekhon LH, et al. Effects of a cervical disc prosthesis on segmental and cervical spine alignment. Neurosurg Focus, 2004, 17(3): E5.
[20] Johnson JP, Lauryssen C, Cambron HO, et al. Sagittal alignment and the Bryan cervical artificial disc. Neurosurg Focus, 2004, 17 (6): E14.
[21] Yanbin Z, Yu S, Zhongqiang C, et al. Sagittal alignment comparison of Bryan disc arthroplasty with ProDisc-C arthroplasty: a prospective, randomized controlled clinical trial. J Spinal Disord Tech, 2011, 24(6): 381-385.
[22] 田伟，韩骁. 颈椎人工间盘置换术. 中华骨科杂志,2010, 30 (11): 1161-1164.
[23] 郝定均,贺宝荣,许正伟,等. Bryan 人工颈椎间盘置换术与椎间融合术治疗颈椎病的中期疗效比较. 中华骨科杂志,2011, 31(1): 18-23.
[24] Quan GM, Vital JM, Hansen S, et al. Eight-year clinical and radiological follow-up of the Bryan cervical disc arthroplasty. Spine (Phila Pa 1976), 2011, 36(8): 639-646.
[25] Walraevens J, Demaerel P, Suetens P, et al. Longitudinal prospective long-term radiographic follow-up after treatment of single-level cervical disk disease with the Bryan Cervical Disc. Neurosurgery, 2010, 67(3): 679-687.
[26] McAfee PC, Cunningham BW, Devine J, et al. Classification of heterotopic ossification (HO) in artificial disk replacement. J Spinal Disord Tech, 2003, 16(4): 384-389.
[27] 申勇, 张英泽, 徐佳欣, 等. Bryan 颈人工间盘置换术失败原因分析. 中华骨科杂志, 2009, 29(7): 639-643.
[28] Suchomel P, Jurák L, Benes V 3rd, et al. Clinical results and development of heterotopic ossification in total cervical disc replacement during a 4-year follow-up. Eur Spine J, 2010, 19(2): 307-315.
[29] Yi S, Kim KN, Yang MS, et al. Difference in occurrence of heterotopic ossification according to prosthesis type in the cervical artificial disc replacement. Spine (Phila Pa 1976), 2010, 35(16): 1556-1561.
[30] Lee JH, Jung TG, Kim HS, et al. Analysis of the incidence and clinical effect of the heterotopic ossification in a single-level cervical artificial disc replacement. Spine J, 2010, 10(8): 676-682.
[31] Chen J, Wang X, Bai W, et al. Prevalence of heterotopic ossification after cervical total disc arthroplasty: a meta-analysis. Eur Spine J, 2012, 21(4): 674-680.
[32] Cunningham BW, Gordon JD, Dmitriev AE, et al. Biomechanical evaluation of total disc replacement arthroplasty: an in vitro human cadaveric model. Spine (Phila Pa 1976), 2003, 28(20): S110-S117.
[33] Dmitriev AE, Cunningham BW, Hu N, et al. Adjacent level intradiscal pressure and segmental kinematics following a cervical total disc arthroplasty: an in vitro human cadaveric model. Spine (Phila Pa 1976), 2005, 30(10): 1165-1172.
[34] Nunley PD, Jawahar A, Kerr EJ 3rd, et al. Factors affecting the incidence of symptomatic adjacent-level disease in cervical spine after total disc arthroplasty: 2-to 4-year follow-up of 3 prospective randomized trials. Spine (Phila Pa 1976), 2012, 37(6): 445-451.
（收稿日期:2012-08-09）
（本文编辑:万瑜）
万方数据

Bryan人工间盘置换与前路减压融合治疗颈椎退行性疾病的中期随访研究

万方数据 WANFANG DATA 文献链接

作者： 田伟， 阎凯， 韩骁， 于杰， 靳培浩， 韩晓光， TIAN Wei， YAN Kai， HAN Xiao， YU Jie， JIN Pei-hao， HAN Xiao-guang
作者单位： 100035,北京积水潭医院脊柱外科
刊名： 中华骨科杂志 ISTIC PKU
英文刊名： Chinese Journal of Orthopaedics
年，卷(期)： 2013,33(2)
被引用次数： 7次

参考文献(34条)

1. Baba H;Furusawa N;Imura S Late radiographic findings after anterior cervical fusion for spondylotic myeloradiculopathy 1993(15)

2. Hilibrand AS;Carlson GD;Palumbo MA Radiculopathy and myelopathy at segments adjacent to the site of a previous anterior cervical arthrodesis 1999(04)

3. Goffin J;Geusens E;Vantomme N Long-term follow-up after interbody fusion of the cervical spine 2004(02)

4. Heller JG;Sasso RC;Papadopoulos SM;Anderson PA;Fessler RG;Hacker RJ;Coric D;Cauthen JC;Riew DK Comparison of BRYAN cervical disc arthroplasty with anterior cervical decompression and fusion: clinical and radiographic results of a randomized, controlled, clinical trial.[外文期刊] 2009(2)

5. McAfee PC;Reah C;Gilder K A meta-analysis of comparative outcomes following cervical arthroplasty or anterior cervical fusion:results from 4 prospective multicenter randomized clinical trials and up to 1226 patients 2012(11)

6. Zhang X;Chen C Randomized,controlled,multicenter,clinical trial comparing BRYAN cervical disc arthroplasty with anterior cervical decompression and fusion in China 2012(06)

7. Yonenobu K;Abumi K;Nagata K Interobserver and intraobserver reliability of the japanese orthopaedic association scoring system for evaluation of cervical compression myelopathy 2001(17)

8. Fukui M;Chiba K;Kawakami M;Kikuchi S;Konno S;Miyamoto M;Seichi A;Shimamura T;Shirado O;Taguchi T;Takahashi K;Takeshita K;Tani T;Toyama Y;Wada E;Yonenobu K;Tanaka T;Hirota Y An outcome measure for patients with cervical myelopathy: Japanese Orthopaedic Association Cervical Myelopathy Evaluation Questionnaire (JOACMEQ): Part 1.[外文期刊] 2007(3)

9. Vernon H;Mior S The Neck Disability Index:a study of reliability and validity 1991(07)

10. Wu S;Ma C;Mai M;Li G Translation and validation study of Chinese versions of the Neck Disability Index and the Neck Pain and Disability Scale.[外文期刊] 2010(16)

11. Odom GL;Finney W;Woodhall B Cervical disk lesions 1958(01)

12. Robertson JT;Papadopoulos SM;Traynelis VC Assessment of adjacent-segment disease in patients treated with cervical fusion or arthroplasty:a prospective 2-year study 2005(06)

13. Miyazaki M;Hong SW;Yoon SH Reliability of a magnetic resonance imaging-based grading system for cervical intervertebral disc degeneration 2008(04)

14. Sasso RC;Anderson PA;Riew KD Results of cervical arthroplasty compared with anterior discectomy and fusion:four-year clinical outcomes in a prospective,randomized controlled trial 2011(18)

15. Nabhan A;Ahlhelm F;Pitzen T Disc replacement using Pro-Disc C versus fusion:a prospective randomised and controlled radiographic and clinical study 2007(03)

16. Murrey D;Janssen M;Delamarter R Results of the prospective,randomized,controlled multicenter Food and Drug Administration investigational device exemption study of the ProDisc-C total disc replacement versus anterior discectomy and fusion for the treatment of 1-level symptomatic cervical disc disease 2009(04)

17. Katsuura A;Hukuda S;Saruhashi Y Kyphotic malalignment after anterior cervical fusion is one of the factors promoting the degenerative process in adjacent intervertebral levels 2001(04)

18. Ferch RD;Shad A;Cadoux-Hudson TA Anterior correction of cervical kyphotic deformity:effects on myelopathy,neck pain,and sagittal alignment 2004(1 suppl Spine)

19. Pickett GE;Mitsis DK;Sekhon LH Effects of a cervical disc prosthesis on segmental and cervical spine alignment 2004(03)

20. Johnson JP;Lauryssen C;Cambron HO Sagittal alignment and the Bryan cervical artificial disc 2004(06)

21. Yanbin Z;Yu S;Zhongqiang C Sagittal alignment comparison of Bryan disc arthroplasty with ProDisc-C arthroplasty:a prospective,randomized controlled clinical trial 2011(06)

22. 田伟,韩骁 颈椎人工间盘置换术[期刊论文]-中华骨科杂志 2010(11)

23. 郝定均,贺宝荣,许正伟,郭华,昌震 Bryan人工颈椎间盘置换术与椎间融合术治疗颈椎病的中期疗效比较[期刊论文]-中华骨科杂志 2011(1)

24. Quan GM;Vital JM;Hansen S Eight-year clinical and radiological follow-up of the Bryan cervical disc arthroplasty 2011(08)

25. Walraevens J;Demaerel P;Suetens P;Van Calenbergh F;van Loon J;Vander Sloten J;Goffin J Longitudinal prospective long-term radiographic follow-up after treatment of single-level cervical disk disease with the Bryan Cervical Disc.[外文期刊] 2010(3)

26. McAfee PC;Cunningham BW;Devine J Classification of heterotopic ossification (HO) in artificial disk replacement 2003(04)

27. 申勇,张英泽,徐佳欣,王林峰,杨大龙,丁文元 Bryan颈人工椎间盘置换术失败原因分析[期刊论文]-中华骨科杂志 2009(7)

28. Suchomel P;Jurák L;Benes V 3rd Clinical results and development of heterotopic ossification in total cervical disc replacement during a 4-year follow-up 2010(02)

29. Yi S;Kim KN;Yang MS;Yang JW;Kim H;Ha Y;Yoon do H;Shin HC Difference in occurrence of heterotopic ossification according to prosthesis type in the cervical artificial disc replacement.[外文期刊] 2010(16)

30. Lee JH;Jung TG;Kim HS Analysis of the incidence and clinical effect of the heterotopic ossification in a single-level cervical artificial disc replacement 2010(08)

31. Chen J;Wang X;Bai W Prevalence of heterotopic ossification after cervical total disc arthroplasty:a meta-analysis 2012(04)

32. Cunningham BW;Gordon JD;Dmitriev AE Biomechanical evaluation of total disc replacement arthroplasty:an in vitro human cadaveric model 2003(20)

33. Dmitriev AE;Cunningham BW;Hu N;Sell G;Vigna F;McAfee PC Adjacent level intradiscal pressure and segmental kinematics following a cervical total disc arthroplasty: an in vitro human cadaveric model.[外文期刊] 2005(10)

34. Nunley PD;Jawahar A;Kerr EJ 3rd Factors affecting the incidence of symptomatic adjacent-level disease in cervical spine after total disc arthroplasty:2-to 4-year follow-up of 3 prospective randomized trials 2012(06)

引证文献(7条)

1. 尹华,赵银必,周忠华,刘俊,张文超 两种内固定方式治疗单节段颈椎间盘突出症的临床比较[期刊论文]-中华临床医师杂志（电子版） 2013(18)

2. 崔泳,王武 人工颈椎间盘置换术后假体位置不良原因分析[期刊论文]-中国矫形外科杂志 2014(19)

3. 李玉伟,王海蛟,严晓云,崔巍,张永辉,李程 颈前路减压零切迹椎间融合器与钉板系统内固定治疗脊髓型颈椎病的疗效比较[期刊论文]-中华骨科杂志 2015(11)

4. 谭荣,马华松,吴继功,陈晓明,周建伟,汪东,杨滨,刘涛 PCM人工颈椎间盘置换术后7年疗效观察[期刊论文]-中国骨与关节杂志 2013(06)

5. 姜东杰,顾庆国,王占超,王新伟,袁文 颈椎人工椎间盘置换术与前路减压融合固定术再手术率的Meta分析[期刊论文]-中华骨科杂志 2015(11)

6. 薛翔 颈椎人工椎间盘置换术研究进展[期刊论文]-中华实用诊断与治疗杂志 2014(03)

7.王威,王利民,王卫东,谭洪宇,刘屹林,张书豪 单节段颈椎人工椎间盘置换与前路椎间融合内固定：维持颈椎活动度和稳定性的比较[期刊论文]-中国组织工程研究 2014(44)

引用本文格式：田伟.阎凯.韩骁.于杰.靳培浩.韩晓光.TIAN Wei.YAN Kai.HAN Xiao.YU Jie.JIN Pei-hao.HAN Xiao-guang Bryan人工间盘置换与前路减压融合治疗颈椎退行性疾病的中期随访研究[期刊论文]-中华骨科杂志 2013(2)
