# Supplementary material for: Adjacent segment degeneration or disease after cervical total disc replacement: a meta-analysis of randomized controlled trials
Source: J Orthop Surg Res. 2018 Oct 3;13:244. doi: 10.1186/s13018-018-0940-9 (PMC6169069; doi:10.1186/s13018-018-0940-9)
Supplement: Supplementary file 2 — File S1. Original data of 11 included articles. (ZIP 12 mb) [file 13018_2018_940_MOESM2_ESM.zip › 11 included articles and original data referred in this article/11 included articles/26 Tao,guan(CHA).pdf]

分类号： R6 单位代码：10752
密 级：公 开 学 号：20110331

# 宁夏医科大学

# 硕士研究生学位论文

## 颈椎人工间盘置换术
## 与颈椎前路减压融合术对相邻节段退变的影响

## Influence of Cervical Disc Arthroplasty and Anterior cervical decompression and fusion on adjacent segment degeneration


学 位 申 请 人： 关涛

指 导 教 师： 金群华教授

申请学位门类级别： 医学

专 业 名 称： 外科学

研 究 方 向： 脊柱外科

所 在 学 院： 临床医学院


论 文 完 成 日 期： 二〇一四年三月

宁夏医科大学研究生院



万方数据

# Ningxia Medical University

# Thesis for Application of Master's Degree

## Influence of Cervical Disc Arthroplasty and Anterior cervical decompression and fusion on adjacent segment degeneration


Student's Name: Guan Tao

Supervisor： Professor Jin Qun Hua


Subject Category: Medicine

Major: Surgery

Specialty: Orthopedic

School: Ningxia Medical University

Completion Date: Mar. 2014

万方数据

# 宁夏医科大学学位论文独创性声明

本人郑重声明：所呈交的学位论文，是个人在导师的指导下，独立进行研究工作所取得的成果，无抄袭及编造行为。除文中已经特别加以注明引用的内容外，本论文不含任何其他个人或集体已经发表或撰写过的作品成果。对本文的研究做出重要贡献的个人和集体，均已在文中以明确方式标明并致谢。本人完全意识到本声明的法律结果由本人承担。

论文作者签名__________　　　　论文导师签名__________

年　月　日　　　　　　年　月　日

# 宁夏医科大学关于学位论文使用授权的声明

宁夏医科大学有权保留使用本人学位论文，同意学校按规定向国家有关部门机构送交论文的复印件和电子版，允许被查阅和借阅。本人授权宁夏医科大学可以将本学位论文的全部或部分内容编入有关数据库进行检索，可以采用影印、缩印或其他复印手段保存和汇编本学位论文。可以公布（包括刊登）论文的全部或部分内容。

（保密论文在解密后应遵守此规定）

论文作者签名__________　　　　论文导师签名__________

年　月　日　　　　　　年　月　日

万方数据

# 颈椎人工间盘置换术与颈椎前路减压融合术对相邻节段退变的影响


## 摘 要

**目的** 比较颈椎人工间盘置换（Cervical Disc Arthroplasty,CDA）与颈椎前路减压融合术(Anterior cervical decompression and fusion,ACDF)治疗颈椎病的临床疗效及邻近节段退变的程度。

**方法** 将 2009 年 8-2012 年 10 月，在宁夏医科大学总医院骨科因单节段颈椎间盘退变疾病进行手术治疗的 66 例患者，随机分为 CDA 组 32 例，ACDF 组 34 例,通过颈椎功能障碍指数 (NDI)和日本骨科学会颈椎治疗评估分数( JOA)评价两组术后的临床疗效。摄颈椎过伸过屈位 X 片及颈椎 MRI，测量邻近节段活动度，应用 KellgrenX 线分级、Miyazaki 颈椎退变分级评价相邻节段椎间盘退变程度。

**结果** 随访时间 19-50 个月，平均 34 个月。ACDF 组 NDI 由术前 38.37±5.32 改善至末次随访 6.31±5.61，JOA 改善率为 75.6%；CDA 组 NDI 由术前 37.75±5.94 改善至末次随访 2.85±2.66，JOA 改善率为 80.4%；相邻节段活动度：ACDF 组相邻上节段平均活动度术前 9.74±2.2°，末次随访 9.98±2.2° ($P$=0.07)，相邻下节段平均活动度术前 7.56±1.2°，末次随访 7.72±1.3° ($P$=0.11)，CDA 组相邻上节段平均活动度术前 9.53±1.7°，末次随访 9.46±1.6° ($P$=0.06)，相邻下节段平均活动度术前 7.26±1.1°，末次随访 7.23±1.2° ($P$=0.14).两组临近节段活动度均未见明显差异；Kellgren 评级中 ACDF 组末次随访共 10 个节段退变（发生率 18.5%）；CDA 组共 7 个节段退变(发生率 14.6%)，两组间无统计学差异。Miyazaki 颈椎间盘退变分级：ACDF 组术后相邻节段发生退变 21 例。CDA 组术后相邻节段发生退变 13 例，两组间无统计学差异。

**结论** CDA 和 ACDF 两组手术方式均能取得良好的临床疗效，尽管相邻节段退变通过影像资料的比较 ACDF 组较 CDA 组差，但两组间无明显统计学差异，即 CDA 并




万方数据

不能明显减缓邻近节段间盘的退变

**关键词** 颈椎前路减压融合术,颈椎人工间盘置换术,相邻节段退变,核磁共振成像



万方数据

# Influence of Cervical Disc Arthroplasty and Anterior cervical decompression and fusion on adjacent segment degeneration

## ABSTRACT

**Objective** Compare curative effect and adjacent segment degeneration after Cervical Disc Arthroplasty and Anterior cervical decompression and fusion.

**Methods** Between August 2009 and October 2012 . There were 66 cases with single segmental cervical disc degeneration disease In the Department of orthopedics, General Hospital of Ningxia Medical University. randomly divided into 34 patents received CDA and 32 patents received ACDF ,The neck disability index (NDI) and JOA to evaluate clinical efficacy of two groups after operation, Adjacent segment degeneration was evaluated on X-ray and magnetic resonance imaging（MRI）. Cervical disc degeneration was evaluated according to Kellgren′ s grading system based on X-RAY and Miyazaki′ s grading system based on T2 -weighted MRI.

**Results** Follow-up time of 19-50 months, mean 34 months. NDI of ACDF group 38.37 ± 5.32 preoperatively improved to 6.31 ± 5.61, JOA improvement rate was 75.6%; NDI of CDA group improved from 37.75 ± 5.94 to 2.85 ± 2.66, JOA improvement rate was 80.4% .Rom of Adjacent segment ：there were no change significantly. Kellgren′ s grading ：there were 10 segments evaluated as degeneration in ACDF group last follow-up (incidence 18.5%); 7 segments in CDA group (incidence of 14.6%), there was no significant difference between the two groups, there were 21 segments evaluated as degeneration in ACDF group last follow-up with Miyazaki，and 13 segments in CDA group，there was no significant difference between the two groups.



万方数据

**Conclusions** ACDF group was higher than CDA group comparis on adjacent segment degeneration by X-ray and MRI during follow-up, but there was no significant difference between the two groups, CDA did not significantly slow down the degeneration of adjacent segment disc.

.

**KEY WORDS** Cervical disc arthroplasty,Anterior cervical decompression and fusion ,Adjacent segment degeneration,Magnetic resonance imaging



万方数据

# 符号说明

| 缩略词 | 英文全称 | 中文名称 |
| --- | --- | --- |
| CDA | Cervical disc arthroplasty | 颈椎人工间盘置换术 |
| ACDF | Anterior cervical decompression and fusion | 颈前路减压植骨融合术 |
| ASD | Adjacent segment degeneration | 相邻节段退变 |
| MRI | Magnetic resonance imaging | 核磁共振 |
| ROM | Range of motion | 活动度 |



万方数据

# 目 录

前言 ……………………………………………………………………………………1
材料与方法 ……………………………………………………………………………5
结果 ……………………………………………………………………………………9
讨论 ……………………………………………………………………………………13
结论 ……………………………………………………………………………………17
参考文献 ………………………………………………………………………………18
附图 ……………………………………………………………………………………22
综述 ……………………………………………………………………………………24
综述参考文献 …………………………………………………………………………33
致谢 ……………………………………………………………………………………38
攻读学位期间发表的学术论文 ………………………………………………………39
个人简历 ………………………………………………………………………………40



万方数据

# 前 言

随着日益增长的生活压力，工薪一族伏案工作时间逐渐延长；随着电子产品推陈更新，“低头族”这个新新人类种群的出现，颈椎病的发病趋势逐渐年轻化发展。什么是颈椎病呢？颈椎病影响成人的脊柱活动的疾病中一种常见的病理状态，其病变的基础是椎间盘退行性病理性改变，常见于中老年患者，并且随着年龄的增长其患病率越高。颈椎病可以表现为多种方式，往往无症状的患者较多，许多患者经历了一个相对良性的疾病发生；然而，随着时间的推移有些患者的症状无法自发改善。如果脊髓一旦受累，就可能引起颈部疼痛或局部疼痛，并可能导致相对应的括约肌，躯干或四肢的神经缺陷，其疾病的发展过程和最终预后可变性极高，无法预测。颈椎病发生发展的主要原因是由于成年累月的颈椎活动不良，导致负荷过载出现颈椎劳损、颈椎椎体上下缘骨赘形成、或颈椎间盘退变，纤维环破裂导致髓核突出或脱出、韧带增厚，致使压迫相对应的脊髓、神经根或椎动脉等组织，引起一系列临床症候群。一旦出现体征的改变，患者往往自发的寻医问药，而对于症状较轻颈椎病患者来说，可以接受单纯保守治疗的方式来得以恢复，可以暂时能缓解病患的不适。而对于诊断明确、症状严重表现为相应运动功能的减退甚至可能形成运动障碍，神经功能的缺失导致相对应支配的区域痛、触觉减低，而且经过正规保守治疗后无明显改善、或者满意度极差的患者可选用适当的手术方法治疗。选择颈椎病手术治疗的目的是为了恢复颈椎正常的生理曲度，纠正颈椎的失稳，维持病椎间的高度，充分解除颈髓神经的压迫，松解挤压的神经根，从而有效减压释放神经周围的空间，挽救颈髓残留的功能，有利于修复颈髓神经功能，阻止病情进一步加重。目前手术方式从入路的选择不同分为前路和后路手术，而最常见的而且广大医师较为关注的手术方式有颈椎前路减压植骨融合术和颈椎间盘切除人工间盘置换术。关于两种手术方式之间优劣，在同时满足两中手术的手术适应证的情况下，选择什么手术方式一直以来都是临床医师较为关注的问题。

经过大半个世纪的时间的临床经验及临床研究结果表示，颈椎融合术是治疗颈椎病的一种有效的手术方式之一,由于其良好的临床效果，患者满意度，手术的安全性受到国



万方数据

际上脊柱外科医生一致认可，而其中颈椎前路减压植骨融合术（Anterior cervical decompression and fusion, ACDF）具有手术暴露方便视野清楚，术中出血较少等优点，甚至有学者认为是治疗合并椎间盘退变性疾病的脊髓型颈椎病及神经根型颈椎病的标准术式[1-2]。该手术方式成功率较高，能够直视下充分减压并植骨融合，同时应用颈前路钢板内固定可即刻提供牢固的颈椎稳定性，并且对椎体后方的任何骨赘连同退变的椎间盘全部去除，能在短时间内显著缓解症状，恢复受压神经的残余功能。据报道的研究结果显示 ACDF 不但增强了颈椎的稳定性，而且减轻神经根性疼痛，脊髓型颈椎病患者改善脊髓功能可能性大于 90％。而且与单纯颈椎前路椎间盘切除术等手术方式想比，颈椎前路减压植骨融合术有良好的长期的缓解症状的结果，而且经过长时间的临床研究表示，目前的所采用的手术材料，包括钢板等材料均是安全的、有效地。而且有研究表明在多节段的手术中其手术结果亦是可以预测的，可以达到良好的术后预期。但 ACDF 也有其局限性，颈椎手术固定后的病椎的融合导致的后果主要就是限制了一个功能性脊椎节段的活动范围，表现在颈椎生物力学的改变，理论上可能在一定程度上使邻近的椎体分担它所带来的代偿性的负担，有可能表现为活动度的改变，甚至可能增加了邻近的功能节段产生持续应力增加，导致邻近间盘功能性退变，从而导致邻近节段疾病的发生。尽管颈椎前路椎间盘切除减压植骨融合术有着优异的临床结果和长期随访资料支持。但是融合手术一个潜在的并发症是病椎相邻节段水平的椎间盘加速退化，是一个促进加速相邻节段退变的因素，导致相邻节段退变的可能。有些文献提出颈椎前路融合术与相邻节段的退行性改变发展水平的相关联，但具体的退变程度报道结果不一。所以 ACDF 术后相邻节段退变的问题已经持续被广大临床医师所关注。而基于颈椎融合手术对相邻节段的影响，颈椎减压术后保留节段活动的理念应运而生，而且渐渐地临床医师对颈椎的运动保存技术产生相当大的兴趣，人工间盘置换术是前路手术中满足保持节段运动的设定，是一种较新的技术，是脊柱外科手术迈了一大步的革命性成果。而且目前的研究文献表明应用颈椎人工间盘的手术，其手术的结果与已行颈椎前路椎间盘切除减压植骨融合术的患者的手术效果相似，颈椎人工间盘开发者们甚至预期颈椎人工间盘可以降低病椎邻近节段退变的速率。近十年来随着手术适应症的日渐明确，假体设计的日益理想，



万方数据

颈椎人工间盘置换术（Cervical Disc Arthroplasty ,CDA）逐步推广应用 [3-5]。

人工颈椎间盘置换术（CDA）应用于临床最重要的理论依据是保留颈椎置换节段的运动功能，还原颈椎的正常生理、解剖形态和生物力学环境，从而有效避免脊柱融合术带来的缺陷。目前不少的临床观察和研究结果均证实，CDA 治疗颈椎退行性疾患早期疗效满意，甚至可以与 ACDF 相媲美, Goffin 等[6]报道单节段和两节段 Bryan 假体间盘置换术后随访 1 年的结果，发现其对于患者神经症状和体征的改善与颈椎前路减压融合术基本相同，影像学资料亦证实其保持了椎体间的运动功能。Hallab 等[7]认为，颈椎人工间盘置换术有以下优点：能够维持病椎的运动功能，在手术后能保证原有的颈椎间高度，而且在置换后的生物力学无明显改变，与周围的组织相融性较好无明显排斥反应，术后随访假体固定位置牢靠，手术成功率较高，对于需要翻修的患者来说提供了二次机会，而且相对于 ACDF 来说执行翻修手术较容易一些。目前已发表的文献和我们自己的经验的分析使我们相信，在广大患者群体颈椎前路椎间盘切除融合术（ACDF）和总椎间盘置换（CDA）后，可以明显改善患者临床症状、神经功能，并恢复到更好的生活质量。但是非融合技术和融合技术之间的研究结果目前仍存在不确定性，人们还难以根据这些结果对脊柱融合术和非融合技术的利弊和优劣作出准确判断。

迄今为止，ACDF 比较 CDA 的邻近节段的临床及影像学检查结果，多家报导结论不一[8]。而且关于邻近节段退变的研究大多都是基于 X 线的比较，而关于较为直观的核磁共振的影像学比较的论文较少，我们需要寻找比较直观的办法。邻近节段退变亦是椎间盘退变的病理生理改变，我们需要从核磁共振对邻近节段椎间盘的评估才是较为直观的，它对于颈椎间盘的髓核及纤维环是否破裂的观察较为明显。而且是对与评价颈脊髓的压迫程度相对直接的评估方式。根据颈椎椎间盘本身的特质，可利用 MRI 评估椎间盘的成分，因椎间盘髓核内主要的成分是水，核磁上 T2 加权像对水的成像较为敏感，鉴于椎间盘的退变必定会导致髓核内的水分或蛋白多糖逐渐减少，也就是说 T2 信号的改变可以成为退变的一个相关性依据。另一方面，从相邻节段椎间盘的高度评估其退变的程度，如果高度发生改变往往提示退变程度较重，且伴随着相应的神经压迫症状。但是目前对颈椎退变性疾病的核磁评估标准有很多，国际上暂未能给予一个统一的评估标



万方数据

准。有些研究应用 Pfirrmann 退变分级系统评价邻近节段椎间盘退变程度，此评价系统最早是应用于腰椎退变的分级。虽然在观察着与被观察者之间有着高度的一致性，但是对于老年病患者椎间盘退变的早期情况分级效力较差[9]。而 Miyazaki 等综合髓核的信号强度，髓核的结构，髓核与纤维环的界限，椎间盘的高度四个方面建立了颈椎退变程度的新的分级系统。通过 1.5T 的核磁共振可以辨别几个级别之间的不同，在一些研究中提示颈椎的退变至颈椎不稳，伴随运动能力的显著下降与 Miyazaki 的研究结果相类似，证明这个评级系统与颈椎退变的相关程度较高[10]。本研究通过影像学中 X 线片结合 MRI 分析两种手术方式术后相邻节段的影像学资料，比较颈椎人工间盘置换与颈椎前路减压融合术对邻近节段退变的影响。



万方数据

# 材料与方法

## 1.1 一般资料

2009 年 8 月至 2012 年 10 月间，在宁夏医科大学总医院骨科需行手术治疗的 66 例颈椎病患者，利用随机数字表的方法将 66 位患者随机分为两组，其中颈椎人工间盘置换术组（32 例）和颈椎前路减压融合术组（34 例），本次研究获得本院伦理委员会的批准，所有患者在手术前签署知情同意书。

## 1.2 纳入标准和排除标准

纳入标准：①病变节段范围为 C3-C7 单节段病变引起的脊髓型颈椎病患者、神经根型颈椎病患者；②病程至少 6 周以上经保守治疗无效或加重，年龄在 30~70 岁；③肢体感觉和/或运动症状逐渐加重；。

排除标准：(l)外伤性脱位骨折；(2)明显的颈椎不稳定。颈椎过伸过屈 X 线可见水平位移大于 3mm;（3）病变椎间隙活动成角大于 11°。

## 1.3 手术方法及术后处理：所有手术均由同一组医生完成。

ACDF 组：麻醉成功后，患者仰卧位，头略后伸。沿右侧胸锁乳突肌行颈前路斜切口或纵行切口暴露。依次切开各层，剥离至椎前筋膜。C 型臂 X 线侧位透视确认颈部各椎体、椎间隙。放置撑开器，用咬骨钳将椎体及椎体后缘对脊髓的致压物咬除，显露并切开、咬除后纵韧带，椎管充分减压。当后纵韧带与硬膜囊粘连致剥离子无法将其分离时，应留置后纵韧带，将椎间盘组织刮除，潜行扩大减压，刮除上下软骨终板，咬除椎体后上下缘增生骨赘。若患者上肢有神经根症状，需刮除部分钩椎关节，沿椎体边缘刮除至两侧椎弓根内缘。在刮除椎间盘组织的间隙植入自体骨块，将大小合适的颈前路钛板（Zephir Anterior Cervical Plate，枢法模.丹力公司）用螺钉分别固定于上下椎体。C 型臂 X 线侧位透视内固定物位置满意，置引流管后冲洗缝合。术后 24 小时拔出引流。术后 2 ～ 3 天 可在颈托保护下地活动。术后颈托佩戴 4 周。

CDA 组：患者全麻后平卧，头颈中立自然后仰，根据病变节段选择侧前横切口或胸锁乳突肌前缘纵切口。切开颈阔肌，肌间隙间钝性分离，将气管、食管拉向左侧，将



万方数据

颈动脉鞘及胸锁乳突肌牵向右侧，达到颈椎前沿。切开椎前筋膜，C 型臂 X 线机透视确定病变的椎间盘。安放颈椎椎体撑开器，椎间隙适度撑开。切开椎间盘纤维环。用髓核钳及刮勺彻底清除纤维及髓核组织，不破坏椎体上、下终板，达后纵韧带，在没有明显硬化和骨赘改变的情况下尽量保留后纵韧带，用刮勺或椎板咬骨钳刮除或咬除椎体后缘增生骨赘，检查并彻底去除突入后纵韧带的髓核及纤维环组织，直至显露硬脊膜，减压除从上下去除骨赘外，左右还应有足够的宽度，尤其是神经根型颈椎病患者，以双侧的钩椎关节为边界，直至脊髓清楚显露，彻底减压。术中边减压边用生理盐水冲洗，使视野清楚及减少骨屑残留，减少异位骨化发生。再次调节颈椎椎体撑开器张力，使颈椎生理前凸及椎间隙高度得以恢复，选择假体试模轻轻打入直到安全限深器碰到前方椎体，此时由 C 型臂 X 线确认试模深度及高度是否合适，试模没有必要过后放置，因为 activ C 的旋转中心已经是偏后设计，安装开槽导向器，开槽，移出试模，植入人工颈椎间盘假体（Active-c Cervical Disc Prosthesis，synthes 公司），再次 C 型臂 X 线透视，见假体位置好，此时移出假体送入器及颈椎椎体撑开器，再次确认假体位置好，逐层缝合切口。术后 24 小时拔出引流。术后 1 天在颈托保护下地活动。术后颈托佩戴 1-2 周起到保护作用。

1.4 疗效评估

颈椎功能障碍指数 (NDI)：颈椎功能障碍指数通过问卷方式评价疼痛对常见生活（疼痛强度，提重物，睡眠，个人阅读，头痛，驾驶能力等）的影响来了解颈椎功能的障碍的程度，分数越高表示功能障碍程度越重，分别记录于术前、术后 3 月、6 月、12 月、 24 月及最后一次随访记录 。

日本骨科学会颈椎治疗评估分数( JOA，17 分)：通过问卷方式评估上下肢神经及运动功能的情况，将得分统计后，计算 JOA 改善率 = ( 术后评分 － 术前评分) /( 17 － 术前评分) × 100%评估患者恢复情况， 75% ～ 100% 为优，50% ～ 74% 为良，25% ～ 49% 为一般，<25% 为差。分别于术前、术后 3 月、6 月、12 月、 24 月及最后一次随访记录。

1.5 影像学评估



万方数据

X线：邻近节段活动度：术前及末次随访时常规检查颈椎正侧位、过屈、过伸位X片。所得X线片资料利用量角器测量相邻节段术前、术后的Cobb角,过屈位与过伸位Cobb角之和即为该节段活动度(ROM)。（见图5

Kellgren X线颈椎退变分级[11]：通过评估邻近节段的椎间隙，有无骨赘情况分为轻、中、重度评估手术邻近节段退变的情况（见表1）。

表 1.KellgrenX 线颈椎退变分级

Kellgren grading standards

| 分级 | |
|---|---|
| 0 级 | 关节间隙正常，无骨赘 |
| 1 级 | 关节间隙可疑变窄，可能有骨赘 |
| 2 级 | 关节间隙可疑变窄，有明显骨赘 |
| 3 级 | 关节间隙变窄明确，有硬化性改变，中等量骨赘 |
| 4 级 | 关节间隙明显变窄，严重硬化性改变，大量骨赘 |

注：0 级表示无退变，1-2 级表示轻度退变，3 级表示中度退变，4 级表示中度退变

MRI: 术前及末次随访检查MRI(1.5T)。并测量矢状正中位T2加权像手术节段相邻节段椎间盘高度。

Miyazaki颈椎间盘退变分级[12]：通过评估邻近节段术前术后的髓核信号强度，髓核的结构，纤维环结构及椎间盘高度评价病椎邻近节段退变的情况（见表2）。



万方数据

表 2. 颈椎间盘 Miyazaki 颈椎间盘退变分级

| 级别 | 髓核信号强度 | 髓核结构及分布 | 髓核与纤维环界限 | 椎间盘高度 |
|---|---|---|---|---|
| I | 高信号 | 均一，白色 | 清晰 | 正常 |
| II | 高信号 | 不均一，中间伴横条纹出现，白色 | 清晰 | 正常 |
| III | 中等信号 | 不均一， 呈灰色至黑色 | 不清晰 | 正常或降低 |
| IV | 低信号 | 不均一， 呈灰色至黑色 | 消失 | 正常或降低 |
| V | 低信号 | 不均一， 呈灰色至黑色 | 消失 | 塌陷 |

所得影像学资料由2位高年资的非手术医师分别独立地进行阅片并评估分级并得出平均值，退变评级如得到不同结果再请1位高年资医师阅片评判。

1.6 统计分析

采用 SPSS17.0 统计软件包进行统计分析。计量资料以均数 ± 标准差表示，每例患者及组内术前、术后的临床疗效评分比较运用配对 t 检验，组间应用重复测量方差分析。两组退变分级应用 Mann-Whitney Test 秩和检验对比。$P<0.05$ 有显著性意义。



万方数据

# 结 果

术后随访时间 19-50 个月，平均 34 个月，失访 6 例。最后随访 CDA 组 28 例，其中脊髓型颈椎病 17 例，神经根型颈椎病 11 例，ACDF 组 32 例，其中脊髓型颈椎病 8 例，神经根型颈椎病 24 例。其中两组患者的平均年龄分别为颈椎前路椎间盘切除减压植骨融合术组 52.63±7.06，颈椎人工间盘置换组 49.61±6.29 岁，P 值为 0.44，男女比例分别为颈椎前路椎间盘切除减压植骨融合术组男 11 例，女 21 例。颈椎人工间盘置换组男 15 例，女 13 例，P 值为 0.15.分别保守治疗的时间，颈椎前路椎间盘切除减压植骨融合术组 1.75±0.54，颈椎人工间盘置换组 2.02±0.71，p 值为 0,19，术前 NDI 指数，颈椎前路椎间盘切除减压植骨融合术组 38.37±5.32，颈椎人工间盘置换组 37.75±5.94，p 值为 0.67.两组神经根型患者与脊髓性患者比例：颈椎前路椎间盘切除减压植骨融合术组，神经根颈椎病患者 8 例，脊髓型颈椎病患者 24 例。颈椎人工间盘置换组，神经根颈椎病患者 11 例，脊髓型颈椎病患者 17 例，*P* 值为 0.24。病变节段比例：颈椎前路椎间盘切除减压植骨融合术组，C3-4 者 5 例， C4-5 者 6 例，C5-6 者 19 例， C6-7 者 3 例。颈椎人工间盘置换组，C3-4 者 3 例， C4-5 者 4 例，C5-6 者 15 例， C6-7 者 6 例，两组比较后的 *P* 值为 0.58，两组间各组数据的比较均未见明显的统计学差异，具有可比性。

## 2.1 疗效评估

颈椎功能障碍指数 (NDI)评分：ACDF 组术前 38.37±5.32，末次随访 6.31±5.61；CDA 组 (NDI)术前 37.75±5.94，末次随访 2.85±2.66。两组患者术后 1 年后均有明显的改善，但根据重复测量方差分析，*P*>0.05 接受两组间 NDI 评分无差别。（详见表 3）



万方数据

表 3 两组患者 NDI 评分对比

| | 术前 | 术后 3 月 | 术后 6 月 | 术后 1 年 | 术后 2 年 | 末次 |
|---|---|---|---|---|---|---|
| ACDF 组 | 38.37±5.32 | 33.93±6.39 | 28.31±6.51 | 13.28±2.48 | 10.12±2.47 | 6.31±5.61 |
| CDA 组 | 37.75±5.94 | 34.36±5.31 | 29.04±5.98 | 13.5±2.52 | 10.25±2.71 | 2.85±2.66 |
| t | 0.51 | 0.539 | 0.45 | 0.517 | 0.00 | 2.36 |
| *P* 值 | 0.67 | 0.592 | 0.655 | 0.607 | 1.00 | 0.22 |

注：组间比较 F=0.775 *P*=0.382>0.05

日本骨科学会颈椎治疗评估分数( JOA，17 分)：ACDF 组术前 9.97±2.13，末次随访 15.18±1.76，其末次平均 JOA 改善率 75%为优；CDA 组术前 10.14±1.97，末次随访 15.64±1.41，其末次平均 JOA 改善率 80%为优。两组间比较根据重复测量方差分析，*P*>0.05 接受两组间 JOA 评分无差别。（详见表 4）

表 4 两组患者 JOA 评分对比

| | 术前 | 术后 3 月 | 术后 6 月 | 术后 1 年 | 术后 2 年 | 末次 |
|---|---|---|---|---|---|---|
| ACDF 组 | 9.97±2.13 | 9.5±2.11 | 10.82±2.09 | 11.64±1.49 | 12.86±1.63 | 15.18±1.76 |
| CDA 组 | 10.14±1.97 | 8.53±1.48 | 10.62±1.12 | 12.34±1.93 | 13.47±1.43 | 15.64±1.41 |
| t | 0.41 | 2.07 | 0.46 | 2.21 | 1.56 | 0.23 |
| *P* 值 | 0.68 | 0.04 | 0.65 | 0.03 | 0.13 | 0.82 |

注：组间比较 F=0.011 *P*=0.917>0.05

2.2 影像学评估。（本研究典型、清晰影像学资料见图 1、图 2、图 3、图 4）



万方数据

相邻节段活动度：ACDF 组相邻上节段平均活动度术前 9.74±2.2°，末次随访 9.98±2.2°(P=0.07)，相邻下节段平均活动度术前 7.56±1.2°，末次随访 7.72±1.3°(P=0.11)，CDA 组相邻上节段平均活动度术前 9.53±1.7°，末次随访 9.46±1.6°(P=0.06)，相邻下节段平均活动度术前 7.26±1.1°，末次随访 7.23±1.2°(P=0.14).两组上下相邻节段活动度术后较术前未见明显统计学差异。（详见表 5）

表 5 两组相邻节段活动度对比（°）

| | | 术前 | 术后 | t 值 | P 值 |
|---|---|---|---|---|---|
| ACDF | 邻近上节段 | 9.74±2.2 | 9.98±2.2 | 1.84 | 0.07 |
| | 邻近下节段 | 9.53±1.7 | 9.46±1.6 | 1.67 | 0.11 |
| CDA | 邻近上节段 | 7.56±1.2 | 7.72±1.3 | 1.94 | 0.06 |
| | 邻近下节段 | 7.26±1.1 | 7.23±1.2 | 1.53 | 0.14 |

注：组间比较 F=0.017 *P*=0.89

Kellgren 颈椎退变分级：ACDF 组：术前 4 个临近节段评价轻度改变，末次随访时 10 个邻近节段评价为退变有（发生率 33.3%），轻度 6 个，中度 3 个，重度 1 个。CDA 组术前 3 个邻近节段评价轻度改变，末次随访时 7 个邻近节段评价为退变(发生率 25%)，轻度 4 例，中度 3 例，重度 0 例。

Miyazaki 颈椎间盘退变分级: 两组未能完善核磁检查的各两例。ACDF 组术前邻近节段评定为退变有 8 个节段，术后相邻节段评定为退变的有 21 个节段。其中术前分级为Ⅰ级，末次随访为Ⅱ级 13 例；术前分级为Ⅱ级，末次随访为Ⅲ级 6 例；术前分级为Ⅲ级，末次随访Ⅳ级 1 例。其中相邻上节段 15 例，下节段 5 例。CDA 组术前 5 个相邻节段评定为退变，术后相邻节段评定为退变 13 例，其中术前分级为Ⅰ级，末次随访为Ⅱ级 8 例；术前分级为Ⅱ级，末次随访为Ⅲ级 5 例，其中上节段 9 例，下节段 4 例。两组相邻节段分级均未发生Ⅴ级退变(详见表 6)



万方数据

表6两组影像资料退变分级比较

| | | Kellgren X 线椎间盘退变分级 | | | Miyazaki颈椎间盘退变分级 | | | |
|---|---|---|---|---|---|---|---|---|
| | | 轻度 | 中度 | 重度 | I级 | II级 | III级 | IV级 |
| ACDF | 术前 | 4 | 0 | 0 | 52 | 7 | 1 | 0 |
| | 末次 | 6 | 3 | 1 | 39 | 14 | 6 | 1 |
| CDA | 术前 | 3 | 0 | 0 | 47 | 5 | 0 | 0 |
| | 末次 | 4 | 3 | 0 | 34 | 13 | 5 | 0 |

注：Kellgren X 线椎间盘退变分级两组间比较: Z值为0.056，*P*值为0.955

Miyazaki颈椎间盘退变分级两组间比较:Z值0.543，*P*值为0.895

## 2.3 并发症

两组患者切口均I期愈合，两例ACDF组患者术后出现吞咽困难，三天后自行缓解。末次随访均无并发症的发生，如假体移位、松动，钢板断裂等。



万方数据

# 讨 论

颈椎前路减压融合术其缓解疼痛及坚固的融合效果得到一致的肯定，并广泛的应用于颈椎退行性疾病的治疗[13]。ACDF 在随访 20 年的结果是成功的，Bohlman 等[14]关于单节段或者双节段的 ACDF 手术术后 20 年甚至 30 年的患者中 67%没有再次发现颈肩部的疼痛。而且发现 88%的患者术后随访中无功能性障碍。此外，80%的患者在手术后恢复工作能力，保持其生活的能力。而相对于融合手术较为受关注的就是颈椎人工间盘置换手术，其主要目标是消除病理椎间盘脱出，同时保持运动，恢复良好的颈椎生理功能，可以取得较好的临床效果，大多数已发表的研究中做了随机对照试验为评估颈椎间盘置换手术的安全性和有效性。Sasso [15]等通过前瞻对照研究发现应用人工颈椎间盘置换手术治疗与融合减压手术对比，4 年随访患者的满意度、肢体疼痛评分表现出持续的优越性。而有关两种手术的比较的已发表文献中，2-4 年的研究表示两种术式手术的成功率结果相似，而且强调了人工间盘置换术患者满意度不低于 ACDF 组患者，甚至提出 CDA 替代 ACDF 的可能。而在本次研究中，两组颈椎病患者末次 NDI 评分及 JOA 评分较术前均有明显改善，而且两组平均术后 JOA 改善率均达到优，说明两种手术方式治疗颈椎病均安全有效，同以往的研究结果相一致，而且在本次研究中我们得出 NDI,JOA 改善率在数值上 CDA 组有优势，但是两组间比较未见明显统计学差异，也就是说在中短期两种手术方式在术后功能恢复方面无明显差异。Coric 等[16]独立分析 269 例单节段颈椎病患者比较了两组视觉模拟评分（VAS ）疼痛评分及颈部残疾指数（NDI）的分数，患者在第 6 周和 3 个月后的随访时 CDA 组较 ACDF 组所得评分都明显降低，但 CDA 组更显著，也就是说在术后镇痛药物的使用方面 CDA 组明显的减少，而且术后恢复更快。但随着时间的推移，在 1 年和 2 年的随访，两种术式的评分基本相同，成功率一致。虽然两者中短期的临床评估均表现优异，但是不可避免的要提到大家所关注的关于术后邻近节段退变的问题。Hilibrand[17]等人通过随访 374 例接受 ACDF 手术的神经根型及脊髓型患者，前十年平均每年相邻节段退变疾病的发生率为 2.9%，总的发生率在 14%左右。有的文献报道认为 ACDF 术后颈椎的运动单元减少，相邻节段运动方



万方数据

式改变，导致邻近节段力学的改变，可能导致邻近节段加速退变[18,19]。而缘于避免手术导致力学的改变，保留病变节段活动度的 CDA，能否减少邻近节段退变的发生呢？

我们通过比较两组患者在 X 射线评估相邻节段发生退变的情况，X 线作为常规的术后复查方式，而且较为直观测量方便，我们测量相邻节段的活动度并应用 Kellgren X 线颈椎退变分级评价其退变情况，在随访期间两组相邻上、下节段的末次随访的活动度较术前均有不同程度的改变，是得到的统计值较术前未见明显差异。这与一些文献的早期临床结果相似，而在一些对比的研究中，CDA 组相邻节段的活动度较术前未见明显改变，优于 ACDF 组的结果[20,21]，而我们两组患者手术节段相邻节段的活动度术前术后的比较未见明显的统计学差异，所得结果的不同可能因为随访时间短、测量角度产生的的误差或摄 X 线位置不够标准导致。颈椎活动度的对比还需要长时间大样本的研究进一步证实。而 Kellgren X 线颈椎退变分级的结果显示。ACDF 组评定为退变发生率 18.5%,CDA 组退变发生率为 14.6%，两组间退变程度通过 Mann-Whitney Test 检验得 Z 值为 0.056，p 值为 0.955 即两组 KellgrenX 线颈椎退变分级未见差别。与我们结果相同的是 Kim [22]通过影像学证据的对比研究发现，人工椎间盘组退变发生率为 12.8%，而融合组为 23.1%。与我们的研究相似，ACDF 组的退变发生率较高，但是两组退变分级的比较未发现明显的差异。

MRI 对与评价颈椎间盘退变最为敏感，可以清晰地看到颈椎间盘内髓核、纤维环。而 MRI 对退变的分析暂时还没有一个统一的标准。我们应用 Miyazaki 颈椎间盘退变分级从间盘信号，纤维环的完整，椎间盘高度等方面综合评价邻近节段退变的程度。经过我们的研究，但两组退变情况经 Mann-Whitney Test 检验得 Z 值 0.543，P 值为 0.895，即两组 Miyazaki 退变分级未见差异。Ajay 等[23]做了一项前瞻性研究将 93 例患者通过计算机随机分配进行 CDA(59 例)和 ACDF(34 例),术后平均随访时间 37 个月,通过影像学评估 CDA 组和 ACDF 组患者发生邻近节段退变分别为 16%、18%未见明显的差异，这与我们的研究结果相符合。而且有关影响学定性定量分析的研究中，经单一的放射医师通过颈椎间高度主观评价分析颈椎人工间盘置换术与 ACDF 相比，CDA 组相邻节段退变的较少，但两组从在手术率的比较上未见明显差异。从我们的研究结果上看，相邻节段



万方数据

退变程度两组间未见明显差异，退变的节段多数发生在术前就评价有退变的节段。Sugawara[24]认为在初次手术前已存在椎间盘退变在术中未处理可能会导致再次手术的可能性。所以我们推测，术前相邻节段有退变的发生，术后容易更进一步发生退变。

然而在颈椎病的治疗方面肯定要涉及到年龄因素,相邻节段退变的问题肯定也涉及到年龄的因素。有人认为手术后相邻节段退变的结果其实是椎间盘退变的自然进程。Herkowitz 等[ 25]进行了一项随机对照研究，随机 44 例神经根型颈椎病的患者随机分配颈椎前路椎间盘切除植骨融合组和后路椎间孔减压组分别手术治疗。经过 4.5 年的影像学随访 ACDF 组 41 ％的患者发现相邻节段退变。然而，令人惊奇的是后路椎间孔切开非融合手术组 50％的患者有相邻节段退化的迹象。 Hilibrand 和 Robbins 调查结果发现，邻近节段病变的确是一个常见的问题，而且有关相邻节段退变的影像学资料与其临床体征之间没有相关性，可能反映了相邻节段颈椎病的自然病程[26] 。Mummaneni[29]等认为的 ACDF 并不是发生相邻节段退变的主要原因。与之相同的是，有些学者认为还有一个核心问题，邻近节段颈椎间盘退变是由于颈椎自然退变过程引起，而与手术方式无关[30]。最近，关于年龄对邻近节段退变的影响的一篇报道说，他们根据年龄将需行前路融合手术的颈椎病患者分为两组。年龄小于 50 岁的患者 38 例，大于 50 岁的患者 49 例。两组之间观察到新的退行性改变的数目没有统计学差异（P = 0.83 ）[27]，也就是说年龄在相邻节段退变的影响不大，所以我们的研究中由于样本量较少未能在年龄方面进一步的分析。

在本次中短期的随机对照研究中，经过对影像学资料的分析，未发现 CDA 组在减少手术邻近节段退变率方面优于 CDA 的有力证据。近期发表的一篇文献关于一个时经 2 年的多中心前瞻性随机对照研究显示表明，CDA 是一个可行的替代 ACDF 的术式，其理论优势是减少相邻级别的压力，从而降低相邻节段退变的风险，但没有一项随机临床试验研究提供可靠证据表明颈椎间盘置换术可以明确减少相邻节段退变的发生[28]。而关于邻近节段的退变有的学者提出新的质疑，Jawahar[31] 等人通过研究发现发生邻近节段退变与个体的差异相关，存在腰椎间盘退变的患者者其发病率尤为明显。究竟手术相邻节段归咎于自然退变还是个体差异，还是别的因素目前仍需要进一步的探索。



万方数据

本次研究的局限在于，随访时间较短，样本数量较少，导致未能从年龄、手术节段具体分析两组患者发生退变的不同，只是比较了两组患者术后相邻节段退变的情况，要想比较两组间邻近节段间盘退变的差异，还需要更大样本、较长期的随访。



万方数据

# 结 论

经过我们的研究，颈椎人工间盘置换术与颈椎前路椎间盘切除融合手术中短期内均能取得良好的临床结果，是治疗颈椎病的安全有效的手术方法。本次研究通过影像学退变分级比较相邻节段退变的情况，两组间未发现明显的统计学差异，认为在中短期两种手术方式对邻近节段的影响无差异，即 CDA 并不能明显减缓邻近节段间盘的退变。



万方数据

# 参考文献

[1] Eck JC，Humphreys SC，Lim TH et al. Biomechanical study on the effect of cervical spine fusion on adjacent level intradiscal pressure and segmental motion[J]. Spine，2002，27 (22): 243l-2434.

[2] Matsunaga S, Kabayama S, Yamamoto T, Yone K, Sakou T, Nakanishi K. Strain on intervertebral discs after anterior cervical decompression and fusion[J]. Spine. 1999;24:670-675.

[3] Sekhon LH. Cervical arthroplasty in the management of spondylotic myelopathy[J]. J Spinal Disord Tech. 2003 Aug;16(4):307-313

[4] Wigfield CC, Gill S, Nelson R, Langdon I, Metcalf N, Robertson J. Influence of an artificial cervical joint compared with fusion on adjacent-level motion in the treatment of degenerative cervical disc disease[J]. J Neurosurg. 2002;96(1 Suppl):17-21.

[5] DiAngelo DJ, Roberston JT, Metcalf NH, McVay BJ, Davis RC. Biomechanical testing of an artificial cervical joint and an anterior cervical plate[J]. J Spinal Disord Tech. 2003;16:314-323.

[6]. Goffin J, Loon J van, Calenbergh F van. et al. A clinical analysis of 4- and 6-year follow-up results after cervical disc replacement surgery using the Bryan Cervical Disc Prosthesis[J]. J Neurosurg Spine. 2010;3(Suppl 1):261-269.

[7] Hallab N, Link HD, McAfee PC. Biomaterial optimization in total disc arthroplasty [J]. Spine, 2003, 28(20): S139-S152.

[8] Baohui Yang; Haopeng Li; Ting Zhang,et al. The incidence of adjacent segment degeneration after cervical disc arthroplasty (CDA): a meta analysis of randomized controlled trials[J]. PLoS One. 2012;7(4)

[9] Pfirrmann CW, Metzdorf A, Zanetti M, et al. Magnetic resonance classification of lumbar intervertebral disc degeneration [J]. Spine, 2001, 26(17): 1873-1878.



万方数据

[10] Miyazaki M, Hong SW, Yoon SH, et al. Reliability of a magnetic resonance imaging -based grading system for cervical intervertebral disc degeneration [J]. J Spinal Disord Tech, 2008, 21(4): 288-292.

[11] Kellgren JH, Jefrey MR, Ball J. The epidemiology of chronic rheumatism. vol II: Atlas of standard radiographs of arthritis[M]. Oxford: Blackwell Scientiic Publications, 1963: 14-19.

[12] Miyazaki M, Hong SW, Yoon SH, et al. Kinematic analysis of the relationship between the grade of disc degeneration and motion unit of the cervical spine[J]. Spine, 2008, 33(2):187-193.

[13] Lin Q, Zhou X, Wang X, Cao P, Tsai N, Yuan W (2011) A comparison of anterior cervical discectomy and corpectomy in patients with multilevel cervical spondylotic myelopathy.［J］Eur Spine J .2012,21(3):474-481.

[14] Bohlman H H, Emery S E, Goodfellow D B, Jones P K. Robinson anterior cervical discectomy and arthrodesis for cervical radiculopathy. Long-term follow-up of one hundred and twenty-two patients. J Bone Joint Surg.1993;75:1298-307.

[15] Sasso RC, Anderson PA, Riew KD, Heller JG. Results of cervical arthroplasty compared with anterior discectomy and fusion: four-year clinical outcomes in a prospective, randomized controlled trial. ［J］J Bone Joint Surg Am ,2011,93: 1684-1692.

[16] Coric D Nunley P D Guyer R D et al. Prospective, randomized, multicenter study of cervical arthroplasty: 269 patients from the Kineflex|C artificial disc investigational device exemption study with a minimum 2-year follow-up: clinical article [J].J Neurosurg Spine 2011. 2348-358.

[17] Hilibrand AS, Carlson GD, Palumbo MA, et al.Radiculopathy and myelopathy at segments adjacent to the site of a previous anterior cervical arthrodesis［J］.J Bone Joint Surg Am 1999;81(4):519-528

[18] Seok Woo Kim Comparison of radiographic changes after ACDF versus Bryan disc



万方数据

arthroplasty in single and bi-level cases[J].Eur Spine,2009,18:218-231.

[19] Bastian L，Lange U，Knop C，et al. Evaluation of the mobility of adjacent segments after posterior thoracolumbar fixation:a biomechanical study［J］. Eur Spine J，2001，10(4):295-300.

[20] Burkus J K, Haid R W, Traynelis V C. et al. Long-term clinical and radiographic outcomes of cervical disc replacement with the Prestige disc: results from a prospective randomized controlled clinical trial. ［J］ Neurosurg Spine. 2010;3:308-318.

[21]Seong Yi, MD, Comparison of anterior cervical foraminotomy vs arthroplasty for unilateral cervical radiculopathy [J].Surgical Neurology, 2009,71:677-680.

[22]Kim SW,Limson MA,Kim SB,et al.Comparison of radiographic changes after ACDF versus Bryan disc arthroplasty in single and bi-level cases[J].Eur Spine,2009,18 (2) :218-231.

[23] Ajay Jawahar.et al.Total disc arthroplasty does not affect the incidence of adjacent segment degeneration in cervical spine: results of 93 patients in three prospective randomized clinical trials[J].Spine,2010,10:1043-1048.

[24] Sugawara T, Itoh Y, Hirano Y, et al. Long term outcome and adjacent disc degeneration after anterior cervical discectomy and fusion with titanium cylindrical cages [J]. Acta Neurochir (Wien), 2009, 151(4): 303-309.

[25] Herkowitz HN, Kurz LT, Overholt DP. Surgical management of cervical soft disc herniation: a comparison between the anterior and posterior approach. Spine.1990;15: 1026-1030.

[26] Gore DR, Sepic SB. Anterior cervical fusion for degenerated or protruded discs: a review of one hundred forty-Six[J]. Spine. 1984:667-71.

[27] Song KJ, Choi BW, Jeon TS, Lee KB, Chang H, et al. Adjacent segment degenerative disease: is it due to disease progression or a fusion-associated phenomenon? Comparison between segments adjacent to the fused and non-fused segments[J].Eur



万方数据

Spine. 2011;20:1940-1945.

[28] Heller JG, Sasso RC, Papadopoulos SM, Anderson PA, Fessler RG, Hacker RJ, et al. Comparison of BRYAN cervical disc arthroplasty with anterior cervical decompression and fusion: clinical and radiographic results of a randomized, controlled clinical trial[J]. Spine.2009;34:101-107.

[29]. Mummaneni PV, Burkus JK, Haid RW, Traynelis VC, Zdeblick TA. Clinical and radiographic analysis of cervical disc arthroplasty compared with allograft fusion: a randomized controlled clinical trial[J]. J Neurosurg Spine. 2007;6:198-209.

[30] McCormick PC. The adjacent segment[J]. J Neurosurg Spine. 2007;6:1-4.

[31] Jawahar A, Cavanaugh DA, Kerr EJ, Birdsong EM, Nunley PD, et al. Total disc arthroplasty does not affect the incidence of adjacent segment degeneration in cervical spine: results of 93 patients in three prospective randomized clinical trials[J]. Spine J. 2010 Dec;10(12):1043-8.



万方数据

# 附　图

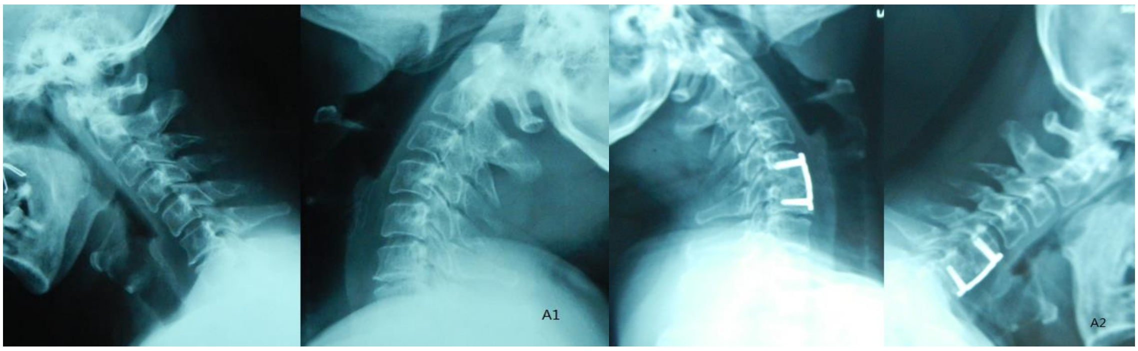


图1ACDF患者术前术后X线表现。老年女性，A1为患者术前过伸过屈位x片，A2为患者末次随方时过伸过屈位片，5.6间隙已经融合，相邻节段活动度较术前无明显变化，无明显骨赘形成。

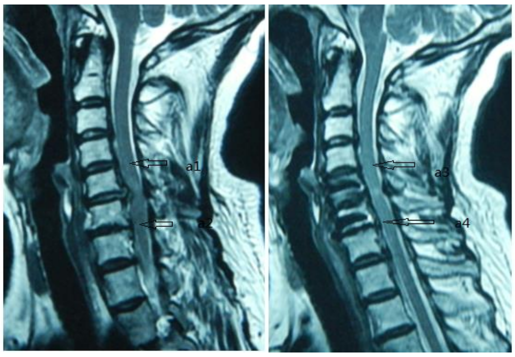


图2示为该老年患者MRI表现，病变节段C5-6,a1为术前病椎相邻上节段C4-5MRI矢状正中位T2加权像Miyazaki评级均为II级、a3末次随访时，Miyazaki评级均为II级，a2为术前病椎相邻下节段，Miyazaki评级均为III级a4为末次随访的MRI矢状正中位T2加权像，Miyazaki评级均为IV级

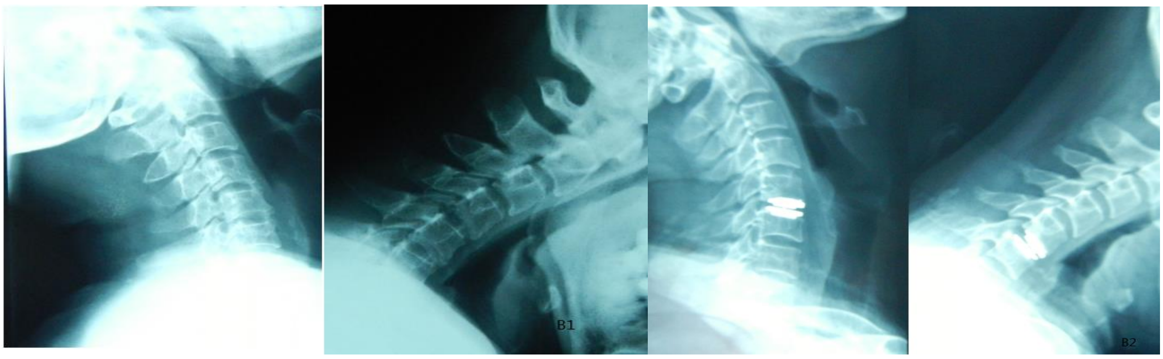


图3 CDA患者术前术后X线表现。中年男性，B1为患者术前过伸过屈位x片，



万方数据

B2为患者末次随方时过伸过屈位片，可见人工间盘假体位置良好，相邻节段活动度较术前无明显变化，无明显骨赘形成。

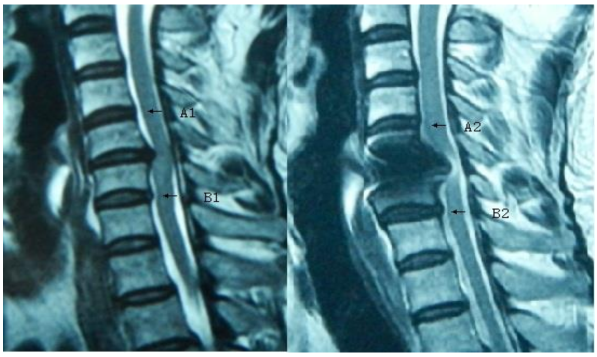


图 4 示为 CDA 组该患者 MRI 表现，病变节段 C5-6.A1，A2 本别为相邻上节段 C4-5 术前、末次随访的 MRI 矢状正中位 T2 加权像，Miyazaki 评级均为 I 级,B1，B2 为相邻下节段 C5-6 术前、末次随访的 MRI 矢状正中位 T2 加权像，Miyazaki 评级均为 II 级

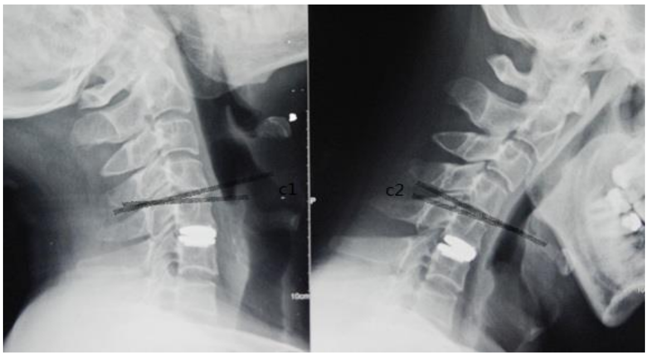


图 5 所示：以颈椎人工间盘置换术后患者的过伸过屈位 X 线测量病椎临近上节段 ROM 为例，过屈位 Cobb 角为 C1，过伸位为 C2，取两者的和为邻近上节段的活动度。



万方数据

# 综 述

## 前路融合手术与非融合手术对邻近节段退变的影响

颈椎病是临床上的常见病和多发病，是由于颈椎间盘退变及其继发性改变导致的神经根、脊髓、椎动脉等临近组织受到刺激和压迫所引起的一系列临床症候群。对于诊断明确、症状严重且经过正规非手术治疗无效的患者可选用适当的手术方法治疗。颈椎病手术治疗的目的是为了解除颈髓神经的压迫，恢复颈椎的稳定性，维持椎间的高度，从而获得颈椎正常的生理曲度及与颈髓相适应的椎管容量，挽救颈髓残留的功能，利于颈髓神经功能的修复，阻止病情进一步加重[1]。目前最常见的手术方式有颈前路间盘切除减压植骨融合术和颈前路椎间盘切除人工间盘置换术等。

半个多世纪以来,颈椎融合术一直是治疗颈椎病变有效且可靠的手段之一,因其良好的临床效果受到广大骨科医生的肯定,特别是颈前路间盘切除减压植骨融合术(ACDF),被有些学者认为是治疗退行性颈椎病的“金标准”[2-3]。但是前路融合术后相邻节段易产生退变和不稳,出现关节活动度增加,造成原有症状复发或加重,而且颈椎总体活动度减小，部分患者有颈椎活动受限症状及临近节段退变。[4]随着医疗技术的发展、临床资料的丰富,融合术后的并发症越来越得到人们的重视,尤其是在融合术后邻近椎体退变方面。融合术改变了原有脊柱的力学行为，必然造成邻近椎体的应力分布及运动模式的改变，相邻节段应力集中、活动代偿增大和稳定性丢失的生物力学改变导致退变加速[5]。与此同时脊柱非融合技术，特别是人工椎间盘置换（CDA）吸引了脊柱专业人士的注意力。相对于融合技术而言，颈椎间盘置换是一种新的技术，是目前世界上治疗颈椎病较为先进的非融合技术的方法之一。随着人工椎间盘置换术的逐渐推广应用,其优良的近期疗效已经得到众多临床研究的证实,也为颈椎间盘突出的手术治疗提供了一个新的选择[6]。在颈椎前路减压后，置入椎间隙，能够保持颈椎原有生理活动，已经有多种人工间盘产品在临床上使用，例如 Byran, PCM,Prodisc-c 等产品。颈椎人工间盘由于能够在术后保持病变节段的活动，



万方数据

理论上能够避免内固定后造成的相邻节段退变[7-9]。但是随着颈椎间盘置换术病例数增加及随访时间延长，其带来的并发症也日益引起学者们的注意[10]。欧洲多中心研究报告 12 个月随访结果，异位骨化发生率达 17.8%，而 Mehren 等报告异位骨化发生率高达 49.4%。还有许多学者报告了假体脱出、下沉等并发症，部分甚至需要行翻修手术。而近期国外的相关报道，更对颈椎间盘置换术能否真正地起到“防止相邻段退变”的问题提出质疑。有些统计结果表明，CDA 与 ACDF 手术后发生相邻节段退变的几率无统计学差异[11、12]。

邻近节段疾病（ASD）以下的配备工具的颈椎前路椎间盘切除融合术（ACDF）据报道，有每年 2.9％的发病率，并在 10 years.1 发生病例约 25％，而它并不完全清楚有多少，这增幅超过椎间盘退变，对椎间盘置换术已经为减少相邻节段退变的可能是最令人信服的论据之一的自然史。的理由是，人工间盘置换术有助于保持正常的脊柱的生物力学的操作水平，从而有助于维持正常的生物力学环境在相邻的水平，从而减少手术后相邻节段疾病的人数。可是结果没有一项有关邻近节段退变的随机试验证实颈椎间盘置换术可以达到[13]预期的效果。本文批判性地探讨了证据表明，人工颈椎间盘置换没有辜负减少相邻节段疾病的承诺。

Hallab 等[14]认为，颈椎人工间盘置换术有以下优点：能够维持病椎的运动功能，在手术后能保证原有的颈椎间高度，而且在置换后的生物力学无明显改变，与周围的组织相融性较好无明显排斥反应，术后随访假体固定位置牢靠，手术成功率较高，对于需要翻修的患者来说提供了二次机会，而且手术相对容易，便于监测，使用寿命可达 50 年。人工间盘置换术相对于 ACDF 能否减缓相邻节段退行性改变的基础和临床上的结果仍存在争议。本文将对手术治疗颈椎病相邻节段退变影响的相关研究作一系统回顾。

1 生物力学研究

CDA 应用于临床最重要的理论依据是保留颈椎置换节段的运动功能，还原颈椎的正常生理、解剖形态和生物力学环境，从而有效避免脊柱融合术带来



万方数据

的缺陷。Goffin 等[15]报道单节段和两节段 Bryan 假体 CDA 后随访 1 年的结果，发现其对于患者神经症状和体征的改善与颈椎前路减压融合术基本相同，影像学资料亦证实其保持了椎体间的运动功能，但对邻近节段的影响和远期效果尚需至少 5 年以上的随访。因此，融合后相邻节段应力是否增加，增加的幅度如何，目前还未有定论。诸多针对颈椎椎体间固定融合术后相邻节段应力变化的生物力学测试也未得出相同的结果，这可能与试验条件、方法不同等因素有关。可以说，非融合技术的生物力学研究结果目前仍存在不确定性，迄今为止人们还难以根据这些结果对脊柱融合术和非融合技术的利弊和优劣作出准确判断。

1.1 运动学

Puttlitz 等[16]采用 6 具新鲜冰冻尸体的颈椎（C2～C7）标本，比较颈椎完整状态以及 Prodisc-C 假体 CDA（手术节段为 C4～C5）术后状态的生物力学参数，利用视觉追踪系统测量屈曲、背伸、轴向旋转 3 个平面运动的颈椎活动度（range of motion，ROM），结果显示，Prodisc-C 颈椎人工椎间盘系统基本模仿了颈椎生理运动形式，耦合运动 ROM 值在两种状态下无显著差异。HaSK 等[17]基于有限元模型对 CDA 术后颈椎进行生物力学研究，也得到相似的结论。Wigfield 等[18]报道人工间盘和模拟前路椎体内融合对颈椎间盘内部应力分布的影响，观察 4 种不同姿势下颈椎间盘受静态负荷的应力结果，从而证实了人工间盘置换具有良好的运动学特性，然而内固定对颈椎相邻节段的影响的生物力学实验结果也不尽相同。Gore 等[19]发现在进行了颈椎前路融合内固定术后，相邻节段的间盘压力及活动度都没有明显增加。他认为颈椎内固定融合后相邻节段的退变可能是颈椎病的一种自然病程。

1.2 力学

颈椎人工间盘保留了颈椎节段活动，可能对相邻节段的应力改变起到预防作用。Dmitriev 等[20]进行了人工间盘(PCM)与颈椎融合术的对比实验，他发现使用颈椎人工间盘组与固定相比，相邻节段的应力明显下降。Chang 等[21]使用



万方数据

了 Prodisc-C 和 Prestiage 人工间盘对比了颈椎融合与间盘置换的效果，他发现融合后，在上一相邻节段，屈曲时颈椎纤维环前方压力增大明显，而后伸时后纤维环压力较对照组有明显改变，间盘置换组则与对照组类似。本文结果同样表明，颈椎人工间盘在一定范围内缓解了相邻节段的压力改变，可减少颈椎相邻节段的负荷。内固定后相邻节段的应力有所增加，但是增加幅度并非那么巨大。相邻节段的病变的起因可能不能够单用生物力学改变这一单一因素解释。脊柱的生物力学测定方法仍然存在许多争议的地方，控制位移还是控制扭矩更加能够符合颈椎在人体内的实际运动状况，仍然不能确定，不同的测量方法可能导致不同的结论。因此，如何能够更真实的模拟人体内颈椎的运动仍然是一个值得研究的课题。同时，使用间盘压力测量的方法也只能反映颈椎运动单元内的间盘应力变化情况，颈椎的其余结构包括前后方的韧带，小关节等的应力变化可能也在颈椎相邻节段的退化中起到作用，准确的测量颈椎各个部分的应力变化情况应该是今后的一个努力方向。

2 ACDF 并发症

2.1 邻近节段退变：邻近节段退变：颈椎前路减压融合内固定术后相关的远期并发症主要为融合部位相邻上下节段发生退行性变，包括形成椎体前、后方骨赘、椎间隙变窄、椎间失稳、椎间盘退变突出等，其发生率高达 50% ～ 60%[22]。Heino 等认为融合术后邻近节段的不稳定性增加了[23]。本组患者中有 51 例出现不同程度的邻近节段退变，发生率为 28. 8%，其中有 5 例因出现临床症状行手术翻修，占随访患者 2. 8%。目前认为脊柱相邻椎间融合术后颈椎的运动单元减少，运动范围减少，运动方式改变，导致邻近节段应力集中，是造成发生邻近节段退变的主要原因[24]。

2.2 植骨不融会造成假关节形成，是植骨融合术后常见的并发症。它的形成很大程度上决定于融合节段的稳定性，即充分的稳定是获得牢固的骨性融合的条件。随着金属板等内固定技术的发展，其发生率已经显著下降[25]。颈椎前路锁定钢板的应用，为术后颈椎提供了即刻的稳定性，能有效地防止植骨块的滑



万方数据

出、促进骨融合、防止假关节形成。假关节的发生与技巧、植骨方法、骨来源、术后活动等多方面因素相关，而且内固定物不确切也是形成假关节的一大诱因，术后内固定不确切可导致植骨块与骨床之间微动，最终出现假关节。

2.3 内固定相关并发症：虽然颈前路钢板的应用在防止植骨块脱出、假关节形成、维持椎间高度、提高植骨融合率等方面的作用已得到肯定，钛网的应用也直接避免了自体取骨所致的髂骨供区并发症[26]，但不可吸收的内固定物存在其特有的并发症。颈前路金属板可出现螺钉松动、断裂、甚至钢板断裂，文献报道发生率为 5%，与操作技术、融合节段和骨质疏松等有关[27]。鉴于传统颈椎手术内固定的诸多并发症，很多学者认为对无明显后凸畸形和节段不稳的单节段颈椎间盘突出或颈椎病，行钛板内固定既无必要又可能带来螺钉退出、钛板松动等一系列并发症。所以，在使用颈椎钛板内固定时，要对金属内植物本身以及使用时带来的相关并发症有足够的认识，严格地掌握适应证，加强手术技巧和操作规范，才能减少相关并发症的发生。如果有一种可吸收的固定板，且其强度足够，则是代替钛板，因而避免相关并发症发生的良好选择。

3 CDA 的并发症

除了颈前路手术常见的术后并发症外，CDA 还有一些特有的并发症，包括减压不彻底、HO、假体位置不佳、椎体骨折等[28-32]。人工间盘的正常磨损及部分碎片的形成对间盘的稳定性基本未构成影响[33]，磨损试验显示人工间盘有很长的使用寿命[34]，但具体情况仍有待长期的临床随访证实。至今尚无人工椎间盘塌陷的临床报道[35]。尼泊尔 BP Koirala 健康研究协会的 Jacob 等[36]过对大量文献的回顾性研究，分析术后并发症出现的原因并提出具体的预防和处理方法。

（1）减压不彻底。

颈椎前路融合术即使减压不彻底，也会因术后神经根无动态刺激而导致根性刺激症状较轻。而 CDA 后因神经根减压不彻底而需要翻修手术的发生率为



万方数据

1.4%～2.5%，高于颈椎前路融合术。翻修手术可通过颈后路的神经根管减压进行，彻底的神经根管减压需要适当剥离两侧颈长肌，以留出足够的减压宽度，从而保证钩椎关节的骨赘得到彻底切除。

（2）异位骨化。

Yi 等[37]对采用 Bryan（81 例）、Mobi-C（61 例）和 ProDisc-C（28 例）3 种假体行 CDA 的 170 例患者进行回顾性研究，HO 的总发生率为 40.6%（69/170），其中 Bryan 组 21.0%，Mobi-C 组 52.5%，ProDisc-C 组 71.4%；对假体生存率的分析表明，所有患者假体生存期平均为（27.1 ± 3.7）个月，Bryan 组较其他组生存期更长，为（48.4 ± 7.4）个月。结果证实 HO 的发生是 CDA 术后不可避免的并发症之一，HO 的发生率较预期为高；不同假体类型之间 HO 的发生率有明确的不同。然而 Ryu 等[38]对 Prodisc-C（17 例）和 Bryan（19 例）应用于单节段 CDA 后至少 24 个月的影像学随访结果显示，HO 发生率较高，但与假体类型无关，而与术前手术节段后纵韧带的钙化有高度相关性。由此可见，CDA 术后如何减少 HO 发生是非常重要的，目前主要的预防措施包括：①彻底清除术中切除的骨屑；②用骨蜡封闭打磨后的骨面和椎间牵开器的钉孔；③术后早期应用 COX-2 类抗炎药。

（3）假体位置不佳。

CDA 术后假体位置不佳包括颈椎节段性后凸，假体位置偏前、偏后或偏离中线，假体向前脱出及向后脱出等。术后节段性后凸的原因是终板的后半部分打磨过度，在术中保持颈椎中立位并平行撑开椎间隙可以避免此类情况的发生。假体位置偏前的原因是由于椎间隙前缘的骨赘清除不足，通过术中透视可以保证假体位置居中；位置偏后的情况较为少见。假体向前和向后脱出的常见原因是选择的假体型号过小，术前对终板的精确测量及术中对椎间隙平行适当撑开可以有助于选择最适合患者的假体型号。

（4）椎体骨折。

CDA 术中椎体骨折见于有中央嵴的假体，改用电动开槽器可以减少其发



万方数据

生。

4 核磁共振关于退变的评级系统

颈椎前、后路手术对于解除颈脊髓或神经根压迫的效果明确，但术后生物力学的改变可能会导致邻近椎间盘退变的速度加快，尤以颈椎前路手术最为明显；而已存在退变且初次手术未处理的椎间盘可能会在随访期间导致再次手术[39]。因此，术前明确椎间盘退变程度与颈椎术后再手术风险的相关性对于初次手术方式和范围的确定具有重要意义。MRI 对于颈椎间盘内髓核、纤维环退变最为敏感，但目前临床多用于评价颈脊髓、神经根的压迫范围和程度。

基于 MRI 评价腰椎间盘退变程度的 Pfirrmann 系统综合了髓核信号高低、髓核分布情况、髓核与纤维界限是否清晰及椎间盘高度四个可量化指标，是目前关于腰椎间盘退变程度分级的主要标准[40]。尽管该系统在观察者间和观察者内的一致性较高，但是其对于老年椎间盘退变和早期退变的区别能力较差。Griffith 等[41]与 Vaga 等[42]对椎间盘退变程度进一步细化，将其分为 8 个级别，但构成评价标准的仍是四种因素。Cuellar 等[43]比较了 28 例髓核成形术失败患者退变椎间盘手术前后的 Pfirrmann 评分，发现约 32%的患者术后 1 年内评分明显增加，该结果与既往关于髓核成形术后邻近椎间盘退变加速的理论一致。Clouet 等[44]在兔椎间盘退变模型中发现 Pfirrmann 分级与 Boos 组织学分级的一致性，而髓核细胞内分子的表达亦与 MRI 分级相关。



万方数据

Pfirrmann 颈椎间盘 MRI 表现的退变评价系统

| 级别 | 髓核信号强度 | 髓核结构及分布 | 髓核与纤维环界限 | 椎间盘高度 |
|---|---|---|---|---|
| I | 高信号 | 均一，亮白色 | 清晰 | 正常 |
| II | 高信号 | 不均一，可有水平带 | 清晰 | 正常 |
| III | 中等信号 | 不均一， 呈灰色 | 不清晰 | 轻度降低 |
| IV | 中到低信号 | 不均一， 呈灰色至黑色 | 消失 | 中度降低 |
| V | 低信号 | 不均一，黑色 | 消失 | 重度降低 |

Miyazaki 等[45]合既往研究结果，将 MRI 上髓核信号强度、髓核结构、髓核与纤维环的界限及椎间盘高度组合后，建立了颈椎间盘退变程度的分级系统，并由 4 名手术者分别独立评价该系统的可靠性，研究者内一致性达 87.3%，研究者间的一致性为 62%，而总体一致性为 72.1%。既往各类报道提示 ，颈椎间盘由正常退变至不稳定、并最终演变为僵硬伴稳定状态的过程中往往伴随着运动能力的下降，而严重退变的颈椎节段往往与运动时前凸角的改变能力显著下降相关[46]。 这与 Miyazaki 的研究结果类似， 同时也证明了该颈椎间盘 MRI 退变分级系统的有效性。



万方数据

Miyazaki 颈椎间盘 MRI 表现的退变评价系统

| 级别 | 髓核信号强度 | 髓核结构及分布 | 髓核与纤维环界限 | 椎间盘高度 |
|---|---|---|---|---|
| I | 高信号 | 均一，白色 | 清晰 | 正常 |
| II | 高信号 | 不均一，中间伴横条纹出现，白色 | 清晰 | 正常 |
| III | 中等信号 | 不均一， 呈灰色至黑色 | 不清晰 | 正常或降低 |
| IV | 低信号 | 不均一， 呈灰色至黑色 | 消失 | 正常或降低 |
| V | 低信号 | 不均一， 呈灰色至黑色 | 消失 | 塌陷 |

5 对手术治疗颈椎病临床作用的认识及展望

随着人工间盘置换技术的研发及应用于颈椎退行性疾患在骨科业界存在争议。目前不少的临床观察和研究结果均证实，CDA 在颈椎退行性疾患早期治疗中患者满意度较高，甚至可以与 ACDF 相媲美[47-49]；运动学和生物力学检测亦表明该技术可降低邻近节段的应力。因此近年来赞同和支持的声音越来越多，甚至有人认为，非融合时代已经到来，它将是脊柱融合技术的终结者[50]。但是，该技术在临床开展的时间还不长，故仍需大样本的长期随访资料来证实其减少或避免邻近节段新发病变的有效性[51，52]；短期内关于相邻节段退变的观点也各不相同。此外，CDA 的临床应用范围较为局限，在颈椎退行性疾病的发生发展过程中，尤其是较严重阶段，脊柱融合技术仍将发挥其重要作用，这种作用是难以被取代的。在颈椎病手术治疗方面还是需临床医师严格把握手术适应症选择最合适的才是关键。



万方数据

# 综述参考文献


[1 ]贾连顺，李家顺 ．颈椎外科手术学〔M〕．上海: 上海远东出版社，2001: 139.

[2] Heller J G，Sasso R C，Papadopoulos S M，et al. Comparison of Bryan cervical disc arthroplasty with anterior cervical decomparess-ion and fusion：clinical and radiographic results of a randomized，controlled，clinicaltrial[J]. Spine，2009，34（2）：101-107.

[3] Korinth M C. Treatment of cervical degenerative disc disease-current status and trends[J]. Zentralbl Neurochir，2008，69（3）：113-124.

[4]曾岩,党耕町,马庆军.颈椎前路融合术后颈部运动功能的评价[J].中华外科杂志,2004,42:1451-1454.

[5]王义生. 脊柱融合术与非融合术不是相互替代而是互补？[J]中国脊柱脊髓杂志, 2011, 2.1(1): 7-8.

[6]孙宇,潘胜发,张风山,等. Bryan 人工椎间盘置换术治疗颈椎病的近期临床效果及出现的问题[ J].中国脊髓病杂志, 2008, l8(1): 13-17.

[7]田伟,刘波,李勤,等.人工颈椎间盘置换手术的临床初步应用体会[J].中华医学杂志,2005,85(1)：37-40.

[8]田伟.人工椎间盘技术预示了脊柱融合手术的终结[J].中华医学杂志,2005,85(1):9.

[9] Robertson JT, Papadopoulos SM, Traynelis VC. Assessment of adjacent-segment disease in patients treated with cervical fusion or arthroplasty: a prospective 2-year study. [J] Neurosurg Spine 2005;3(6):417-423.

[10] 任先军，王卫东，初同伟，等.人工颈推间盘置换术后早中期临床疗效及并发症评价. 中国骨与关节损伤杂志，[J]2009，24（10）：865-868.

[11] Burkus JK, Haid RW, Traynelis VC, et al. Long-term clinical and radiographic outcomes of cervical disc replacement with the Prestige disc: results from a prospective randomized controlled clinical trial[J]. J Neurosurg Spine, 2010, 13(3): 308-318.

[12] Jawahar A, Cavanaugh DA, Kerr EJ 3rd, et al. Total disc arthroplasty does not affect the




万方数据

incidence of adjacent segment degeneration in cervical spine: results of 93 patients in three prospective randomized clinical trials[J]. Spine J, 2010, 10(12): 1043-1048.

[13]Bartels RH, Donk R, Verbeek AL. No justification for cervical disk prostheses in clinical practice: a meta-analysis of randomized controlled trials. Neurosurgery.2010;66(6):1153–60.

[14] Hallab N, Link HD, McAfee PC. Biomaterial optimization in total disc arthroplasty [J]. Spine, 2003, 28(20): S139-S152.

[15] Goffin J, Van-Calenbergh F, van-Loon J, et al. Intermediate follow-up after treatment of degenerative disc disease with the Bryan Cervical Disc Prosthesis: single-level and bi-level [J]. Spine, 2003, 28(24): 2673-2678.

[16] Puttlitz CM, Rousseau MA, Xu Z, et al. Intervertebral discreplacement maintains cervical spine kinetics [J]. Spine,2004, 29(24): 2809-28l4.

[17] HaSK. Finite element modeling of multi-level cervical spinalsegments(C3-C6) and biomechanical analysis of an elastomer-type prosthetic disc [J]. Med Eng Phys, 2006, 28(6): 534-541.

[18] Wigfield CC, Skrzypiec D, Jackowski A, et al. Internal stressdistribution in cervical intervertebral discs: the influence of an artificial cervical joint and simulated anterior interbody fusion [J]. J Spinal Disord Tech, 2003, 16(5): 441-449.、

[19] Gore DR, Sepic SB. Anterior cervical fusion for degenerated or protruded discs: a review of one hundred forty-Six[J]. Spine. 1984:667-71.

[20]Dmitriev AE, Cunningham BW, Hu N, et al. Adjacent level intradiscal pressure and segmental kinematics following a cervical total disc arthroplasty: an in vitro human cadaveric model[J]. Spine. 2005:1165-1172.

[21]Chang UK, Kim DH, Lee MC, et,al.Changes in adjacent-level disc pressure and facet joint force after cervical arthroplasty compared with cervical discectomy and fusion[J] . J Neurosurg Spine 2007;7(1):33-39



万方数据

[22]侯铁胜,赵杰,傅强,等.PCB 系统在颈椎间盘突出症中的应用[J]. 中国脊柱脊髓杂志,2003,3:177-179

[23]Heino K,Michael K,David H,et al.Integrated outcome assessment after anterior cervical discectomy and fusion[J].Spine,2004,22:2501-2509.

[24]Sungjae L,Kurt GH,Vijay KG,et al.Spinal motion after cervical fu-sion[J].Spine,1994,20:2336-2342.

[25]Farey ID,McAfee PC,Davis RF,et al.Pseudoarthrosis of the cervical spine after anterior arthrosis[J].J Bone Joint Surg(Am),1990,72:1171-1177.

[26]Bose B.Anterior cervical fusion using Caspar plating:analysis of re-sults and review of the literature[J].Surg Neural,1998,49:25-31.

[27]Fujibayashi S,Shikata J,Kamiya N,et al.Missing anterior cervical plate and screw:a case report[J].Spine,2000,25:2258-2261.

[28] 苏庆军, 康南, 王庆一. 人工椎间盘置换术的并发症及预防[J] . 中国脊柱脊髓杂志, 2004, l4(6): 374-376.

[29] Ryu KS, Park CK, Jun SC, et al. Radiological changes of the operated and adjacent segments following cervical arthroplasty after a minimum 24-month follow-up: comparisonbetween the Bryan and Prodisc-C devices [J]. J Neurosurg Spine, 2010, 13(3): 299-307.

[30] Stulik J, Kryl J, Sebesta P, et al. ProDisc-C mobile replacement of an intervertebral disc. A prospective mono-centric two-year study [J]. Acta Chir Orthop Traumatol Cech, 2008,75(4): 253-261.

[31] Lee JH, Jung TG, Kim HS, et al. Analysis of the incidence and clinical effect of the heterotopic ossification in a single level cervical artificial disc replacement [J]. Spine J, 2010, 10(8): 676-682.

[32] Suchomel P, Jurak L, Benes V 3rd, et al. Clinical results and development of heterotopic ossification in total cervical disc replacement during a 4-year follow-up [J]. Eur Spine J,



万方数据

2010,19(2): 307-315.

[33] Sekhon LH, Ball JR. Artificial cervical disc replacement:principles, types and techniques [J]. Neurol India, 2005, 53(4): 445-450.

[34] Pracyk JB, Traynelis VC. Treatment of the painful motionsegment: cervical arthroplasty [J]. Spine, 2005, 30(16 Suppl):523-532.

[35] Acosta FL Jr, Ames CP. Cervical disc arthroplasty: generalintroduction [J]. Neurosurg Clin N Am, 2005, 16(4): 603-607.

[36] Jacob M, Broekhuizen FF, Castro W, et al. Experience using cryotherapy for treatment of cervical precancerous lesions in low-resource settings [J]. Int J Gynaecol Obstet, 2005, 89( 2): 513-520.

[37] Yi S, Kim KN, Yang MS, et al. Difference in occurrence of cervical artificial disc replacement [J]. Spine, 2010, 35(16):1556-1561.

[38] Sugawara T, Itoh Y, Hirano Y, et al. Long term outcome and adjacent disc degeneration after anterior cervical discectomy and fusion with titanium cylindrical cages [J]. Acta Neurochir (Wien), 2009, 151(4): 303-309.

[39] Pfirrmann CW, Metzdorf A, Zanetti M, et al. Magnetic resonance classification of lumbar intervertebral disc degeneration[J]. Spine, 2001, 26(17): 1873-1878.

[40] Griffith JF, Wang YX, Antonio GE, et al. Modified Pfirrmann grading system for lumbar intervertebral disc degeneration[J]. Spine, 2007, 32(24): E708-712.

[41] Vaga S, Brayda-Bruno M, Perona F, et al. Molecular MR imaging for the evaluation of the effect of dynamic stabilization on lumbar intervertebral discs [J]. Eur Spine J,2009, 18 (Suppl 1): 40-48.

[42] Cuellar VG, Cuellar JM, Vaccaro AR, et al. Accelerated degeneration after failed cervical and lumbar nucleoplasty[J].J Spinal Disord Tech, 2010, 23(8): 521-524.

[43] Clouet J, Pot-Vaucel M, Grimandi G, et al. Characterizationof the age dependent intervertebral disc changes in rabbit by correlation between MRI, histology and gene



万方数据

expression[J]. BMC Musculoskelet Disord, 2011, 12: 147.

[44] Miyazaki M, Hong SW, Yoon SH, et al. Kinematic analysis of the relationship between the grade of disc degeneration and motion unit of the cervical spine[J]. Spine, 2008, 33(2):187-193.

[45] Axelsson P, Karlsson BS. Intervertebral mobility in the progressive degenerative process: a radiostereometric analysis[J].Eur Spine J, 2004, 13(6): 567-572.

[46] Bryan VE. Cervical motion segment replacement [J]. EurSpine J, 2002, 11(Suppl 2): S92-S97.

[47] Wang Y, Zhang X, Xiao S, et a1. Clinical report of cervical arthroplasty in management ofspondylotic myelopathy in Chinese.J Orthop Surg. 2006, 11(41): l3.

[48] 孙宇. 颈椎人工椎间盘置换术-我们的未来？[J] . 中国脊柱脊髓杂志, 2008, 18(1): 11.

[49] 王岩. 颈椎人工椎间盘置换相对于传统前路融合手术的优势[J] . 中国脊柱脊髓杂志, 2008, 18(1): 8-9.

[50] Guyer RD, Ohnmeiss DD. Intervertebral disc prostheses [J].Spine, 2003, 28(15 Suppl): S15-S23.

[51] Bertagnoli R, Yue JJ, Pfeiffer F, et al. Early results afterProDisc-C cervical disc replacement [J]. J Neurosurg Spine,2005, 2(4): 403-410.

[52] Peng-Fei S, Yu-Hua J. Cervical disc prosthesis replacementand interbody fusion: a comparative study [J]. Int Orthop,2008, 32(1): 103-106.



万方数据

# 致 谢

首先，感谢我的导师金群华教授。感谢他在我三年的硕士求学生涯中，从学业和生活上给予我的悉心培养和无微不至的关怀；在课题的整个设计到最后的完成，文章的撰写、修改等方面导师倾注了很多心血；导师高尚的品德，渊博的学识，严谨的作风，丰富的科研经验使我受益匪浅，并将影响着我今后的科研生涯。我衷心感谢导师对我的辛勤培养。

感谢骨三科全体老师在我临床实践过程中给予的细心指导和教悔，他们对待科学严谨求实的作风，废寝忘食的敬业精神永远是我学习的榜样，衷心地感谢这个温暖大家庭的每一位成员。

感谢宁夏医科大学放射科朱凯老师在核磁拍摄及校对完成过程中给予的指导和帮助。

感谢我的家人及亲人给予我无限的支持和鼓励，正是他们默默的奉献和无私的资助才使我顺利完成了学业，使我能勇敢的面对困难，不断成长进步。

最后，向在我研究生学习生活中给予关心和帮助人致以最诚挚的谢意和美好的祝福！

# 攻读学位期间发表的学术论文



万方数据

关涛,胡志伏,修磊,李楠.颈椎人工间盘置换术与颈椎前路减压融合术对相邻节段退变的影响.中国修复重建杂志.2014（8）



万方数据

# 个 人 简 历

## 一般情况：

姓　　名　　　　　　　关涛

性　　别　　　　　　　男

年　　龄　　　　　　　30 岁

民　　族　　　　　　　满

籍　　贯　　　　　　　宁夏

## 学习经历：

2002 年～2007 年　　　黑龙江省佳木斯大学　　　　攻读本科

2011 年～2014 年　　　宁夏医科大学　　　　　　　攻读硕士



万方数据

# 颈椎人工间盘置换术与颈椎前路减压融合术对相邻节段退变的影响

万方数据 WANFANG DATA 文献链接

作者： 关涛
学位授予单位： 宁夏医科大学

引用本文格式：关涛 颈椎人工间盘置换术与颈椎前路减压融合术对相邻节段退变的影响[学位论文]硕士 2014
