# Supplementary material for: Adjacent segment degeneration or disease after cervical total disc replacement: a meta-analysis of randomized controlled trials
Source: J Orthop Surg Res. 2018 Oct 3;13:244. doi: 10.1186/s13018-018-0940-9 (PMC6169069; doi:10.1186/s13018-018-0940-9)
Supplement: Supplementary file 2 — File S1. Original data of 11 included articles. (ZIP 12 mb) [file 13018_2018_940_MOESM2_ESM.zip › 11 included articles and original data referred in this article/11 included articles/27 Li, Zhonghaiú¿CHAú⌐.pdf]

Clinical and radiologic comparison of dynamic cervical implant arthroplasty versus anterior cervical discectomy and fusion for the treatment of cervical degenerative disc disease

- Zhonghai Li[a, 1],
- Shunzhi Yu[b, 1],
- Yantao Zhao[a],
- Shuxun Hou[a, ,] ,
- Qiang Fu[c],
- Fengning Li[c],
- Tiesheng Hou[b],
- Hongbin Zhong[a]

Show more

doi:10.1016/j.jocn.2013.09.007

Get rights and content

Abstract

This study compared the clinical and radiological outcomes of dynamic cervical implant (DCI; Scient'x, Villers-Bretonneux, France) arthroplasty *versus* anterior cervical discectomy and fusion (ACDF) for the treatment of cervical degenerative disc disease. This prospective cohort study enrolled patients with single-level cervical degenerative disc disease who underwent DCI arthroplasty or ACDF between September 2009 and June 2011. Patients were followed up for more than 2 years. Clinical evaluation included the Medical Outcomes Study 36-Item Short Form Health Survey (SF-36), Neck Disability Index (NDI), Japan Orthopedic Association (JOA) score, and visual analog scale (VAS) scores for neck and arm pain. Radiological assessments included segmental range of motion (ROM), overall ROM (C2–C7), disc height (DHI), and changes in adjacent disc spaces. The VAS, SF-36, JOA, and NDI scores improved significantly after surgery in both the DCI and ACDF groups. The VAS, JOA, and SF-36 scores were not significantly different between the DCI and ACDF groups at the final follow-up. The segmental ROM at the treated level and overall ROM increased significantly after surgery in the DCI group, but the ROM in the adjacent cephalad and caudal segments did not change significantly. The mean DHI at the treated

level was significantly restored after surgery in both groups. Five patients (12.8%) in the DCI group showed new signs of adjacent segment degeneration. These results indicate that DCI is an effective, reliable, and safe procedure for the treatment of cervical degenerative disc disease. However, there is no definitive evidence that DCI arthroplasty has better intermediate-term results than ACDF.

Keywords

- Adjacent segment degeneration;
- Anterior cervical discectomy and fusion;
- Cervical degenerative disc disease;
- Complications;
- Dynamic cervical implant;
- Surgical outcome

---

1. Introduction

The anterior approach to surgical treatment of cervical degenerative disease was first described by Robinson and Smith and popularized by Cloward in the 1950s. Currently, anterior cervical discectomy and fusion (ACDF) is considered to be the definitive surgical treatment for symptomatic, single-level, cervical degenerative disc disease (DDD). Many studies have reported that ACDF is highly effective in terms of resolving symptoms, improving nerve function, and restoring the physiological curvature of the cervical spine[1], [2], [3] and [4]. However, fusion alters the normal biomechanics of the spine, which may result in acceleration of adjacent segment degeneration (ASD) and a need for subsequent reoperation. Limitations and problems with ACDF have led some investigators to explore motion-preserving procedures such as artificial cervical disc arthroplasty [5], [6], [7] and [8]. In recent years, dynamic or non-fusion stabilization of the cervical spine has attracted attention as a possible treatment for cervical DDD [9],[10] and [11].

The dynamic cervical implant (DCI; Scient'x, Villers-Bretonneux, France) is a new device designed to achieve anterior decompression without cervical fusion, and is mainly used to treat cervical DDD (Fig. 1) [12]. The first generation DCI products were developed in 2002, but the clinical efficacy of these products has not been reported. Paradigm Spine (New York, NY, USA) made improvements to the first generation products in 2005, and the second generation DCI products have been used in clinical practice since 2008.

Three heights and four models are available. The main features of the device are as follows: (1) implantation does not result in the generation of debris; (2) it fits well on thevertebral endplate, resulting in immediate postoperative stability; (3) placement is relatively non-invasive, thereby avoiding heterotopic ossification; (4) it maintains the height of the intervertebral gap; (5) the axial compliance and ability to absorb vibrations can avoid accelerated degeneration of the intervertebral discs of the adjacent segments; and (6) the inverted teeth on the leading edges are embedded in the upper and lower vertebral bodies to achieve axial stability and reduce the tension and pressure forces during flexion and extension of the neck. The device results in some limitation of rotation and translation, thereby preventing further degeneration of the small joints. DCI arthroplasty has developed over the last two decades to enable normal motion and preserve biomechanics in an attempt to overcome the disadvantages of fusion, while providing sufficient stability to restore normal segmental kinematics, control abnormal motion, enable greater physiological load transmission, and reduce or eliminate ASD. DCI arthroplasty could potentially replace cervical fusion for the treatment of selected patients with cervical DDD.

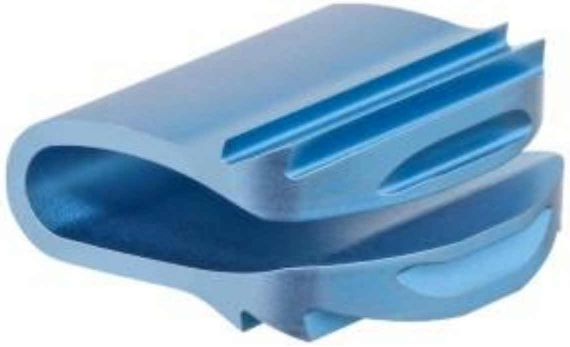

Fig. 1.

Photograph showing the dynamic cervical implant (Scient'x, Villers-Bretonneux, France).

Figure options

From September 2009 to June 2011, we performed resection of the anterior cervical intervertebral discs and DCI arthroplasty in 39 patients. To our knowledge, this is the first reported study of clinical and radiological outcomes in patients who underwent DCI arthroplasty for cervical DDD. The aims of this study were to compare the safety and efficacy of DCI arthroplasty *versus* ACDF in patients with single-level cervical DDD by evaluation of the clinical and radiological data, and to assess the role and limitations of DCI arthroplasty for the treatment of cervical DDD.

2. Materials and methods

2.1. Patient population

This prospective cohort clinical trial compared ACDF and DCI arthroplasty. The study included 86 consecutive patients who underwent surgery for single-level cervical DDD by a single surgeon in our spine surgery center. Five patients were excluded because their 1 year follow-up data were incomplete. All patients were diagnosed based on preoperative radiograph, CT scan and MRI findings.

All patients were older than 18 years and had single-level symptomatic DDD between C3 and C7 with intractable radiculopathy or myelopathy. Thirty-nine patients (48.2%) had radicular pain, 18 (22.2%) had myelopathy, and 24 (29.6%) had both radiculopathy and myelopathy. Most patients had a history of incapacitating neck and arm pain lasting longer than 6 weeks which was unresponsive to non-surgical management such asphysical therapy and anti-inflammatory medication, or had a new neurological deficit resulting from myelopathy. The exclusion criteria were ossification of the posterior longitudinal ligament, severe facet arthritis, lack of motion or instability at the level of surgery, narrowing of the spinal canal, fracture, infection, tumor, and osteoporosis. All patients enrolled in the study were suitable candidates for both DCI and ACDF. Finally, 81 patients (44 men and 37 women) were deemed eligible for inclusion in the study. The mean age of patients was 47.8 years (range 36–61 years) and the mean duration of symptoms was 25.2 months (range 2–86 months).

Thirty-nine patients underwent resection of the anterior cervical intervertebral disc and DCI arthroplasty (DCI group), including three at C3–C4, 15 at C4–C5, 18 at C5–C6, and three at C6–C7. The remaining 42 patients underwent ACDF (ACDF group) (Table 1). After surgery, radiological investigations were performed at 1 week, 3 months, 6 months, and at 6 month intervals thereafter.

Table 1.

Demographic data of patients who underwent surgery for single-level cervical degenerative disc disease

	DCI group	ACDF group
Patients, n	39	42
Male, %	53.8	54.8
Age in years, mean ± SD (range)	45.3 ± 8.6 (36–55)	49.5 ± 9.3 (41–61)
Operated level		
C3–C4	3	3
C4–C5	15	18

	DCI group	ACDF group
C5–C6	18	20
C6–C7	3	1
Follow-up in months, mean (range)	26.7 (24–36)	35.4 (24–45)

ACDF = anterior cervical discectomy and fusion, DCI = dynamic cervical implant.

Table options

2.2. Surgical technique

All patients received preoperative intravenous antibiotics. All procedures were performed through a transverse skin incision on the right side of the neck. Discectomy and decompression were performed using a surgical approach similar to that described by Smith and Robinson [11], with preservation of the uncovertebral joints to minimize soft tissue damage and bleeding and to avoid damage to the bony end-plates. To reduce new bone formation at bleeding sites, soft tissue bleeding was meticulously controlled, and damaged bone was covered with bone wax. The posterior longitudinal ligaments were completely removed only when they were found to be torn preoperatively. ACDF procedures were performed using a titanium mesh cage and Slim-Loc plate (DePuy Spine, Johnson & Johnson, Piscataway, NJ, USA). Operations were performed underfluoroscopic guidance. All patients were immobilized in a Philadelphia collar for 4 weeks postoperatively.

2.3. Data collection and outcome evaluation

The data collected included age, sex, operative segment, intraoperative blood loss, operation time, complications, and clinical and radiological parameters. Perioperative information was collected from the anesthesia records.

The self-reported measures used were the Medical Outcomes Study 36-Item Short Form Health Survey (SF-36) [13], Neck Disability Index (NDI) [14], and visual analog scale (VAS) scores for neck and arm pain. All patients were asked to complete questionnaires before surgery and at each follow-up examination. The NDI and VAS scores ranged from 0 to 100. Odom’s grading system (poor, fair, good, or excellent) was used to evaluate patient satisfaction with the surgery [15]. Outcomes were graded as excellent if all preoperative symptoms were relieved and patients were able to perform their daily activities without impairment; good if they had minimal persistence of preoperative symptoms and were able to perform their daily activities without significant impairment; fair if they had relief of some preoperative symptoms, but their physical activities were significantly limited; and poor if their

symptoms and signs were unchanged or worse. Myelopathy was graded using the Japanese Orthopedic Association (JOA) score [16]. Anteroposterior, lateral, and flexion-extension radiographs were taken before surgery, within 1 week after surgery, and at 3 and 6 months after surgery. Subsequent follow-up examinations were performed every 6 months. Two-dimensional CT scan with sagittal and coronal reconstructions was performed preoperatively to detect ossification of the posterior longitudinal ligament. MRI was also routinely performed to evaluate preoperative spinal cord compression. The segmental (cephalad, treated, and caudal disc levels) and overall (C2–C7) range of motion (ROM) were measured on the dynamic full flexion and extension radiographs, and the angle of the implant was measured on the immediate postoperative lateral radiograph (Fig. 2) [17]. A kyphotic angle was considered to be a positive value. The disc height (DHI) was measured on the lateral radiograph as the distance from the highest portion of the lower end-plate of the cephaladvertebra to the closest portion of the upper end-plate of the caudal vertebra (Fig. 3). Degenerative changes in the adjacent segments were evaluated on MRI at 12 or 24 months postoperatively. Disc degeneration was graded on T2-weighted sagittal and axial images using the five-point scale as described by Miyazaki [18]. To correct for intra-observer and inter-observer differences in radiological measurements, three experienced observers independently evaluated radiological outcomes.

Fig. 2.

Method of range of motion (ROM) measurement. Segmental ROM was measured using dynamic flexion (A) and extension (B) radiographs. The segmental ROM at the treated level is b1–b2.

Figure options

Fig. 3.

Method of disc height (DHI) measurement. (A, B) DHI was measured on a lateral radiograph, and was defined as the distance from the highest portion of the lower end-plate in the cephalad vertebra to the closest portion of the upper end-plate in the caudal vertebra (line). 3 = C3, 4 = C4.

Figure options

2.4. Statistical analyses

All analyses were performed using the Statistical Package for the Social Sciences, version 17.0 (SPSS Inc., Chicago, IL, USA). Intergroup comparisons were performed using the *t*-test, Wilcoxon signed-rank test, Pearson's chi-squared test, or Fisher's exact test. Clinical and radiological data before and after surgery were compared using the Wilcoxon signed-rank test or paired *t*-test. Results are expressed as the mean ± standard deviation, with a p value of <0.05 considered statistically significant.

3. Results

3.1. Surgical outcomes

All patients were followed up for more than 2 years postoperatively (mean 32 months, range 24–45 months). The operated levels included C3–C4, C4–C5, and C5–C6 in both groups. There were no significant differences in age, number of decompressed segments, or sex between the DCI and ACDF groups. There were also no significant differences between the two groups in operation time (52.4 ± 12.6 minutes *versus*55.3 ± 13.2 minutes, $p > 0.05$) or blood loss (32.5 ± 10.4 mL *versus* 35.3 ± 11.6 mL,$p > 0.05$) (Table 2).

Table 2.

Clinical outcomes of patients in the DCI and ACDF groups

	DCI group (n = 39)	ACDF group (n = 42)
Operation time, minutes	52.4 ± 12.6	55.3 ± 13.2
Blood loss, mL	32.5 ± 10.4	35.3 ± 11.6
Preoperative VAS for neck	57.4 ± 5.8	59.7 ± 16.7
Preoperative VAS for arm	69.5 ± 19.7	70.3 ± 17.5
Final follow-up VAS for neck	11.6 ± 10.9[*]	13.2 ± 11.7[*]
Final follow-up VAS for arm	17.2 ± 9.6[*]	19.5 ± 10.2[*]
Preoperative NDI score	19.8 ± 7.2	21.8 ± 6.9
Final follow-up NDI score	5.8 ± 2.9[*]	10.2 ± 3.4[*#]
Preoperative SF-36 score	28.4 ± 6.3	29.5 ± 7.2
Final follow-up SF-36 score	46.6 ± 8.7[*]	43.8 ± 9.1[*]
Preoperative JOA score	9.4 ± 2.1	9.8 ± 2.2
Final follow-up JOA score	14.8 ± 2.0[*]	14.9 ± 2.1[*]
Odom's scale (excellent/good/fair/poor)	9/24/5/1	8/26/7/1

Data are presented as mean ± standard deviation.

ACDF = anterior cervical discectomy and fusion, DCI = dynamic cervical implant, JOA = Japanese Orthopedic Association, NDI = Neck Disability Index, SF-36 = Medical Outcomes Study 36-Item Short Form Health Survey, VAS = visual analog scale.

*
p < 0.05 compared with preoperative.

#
p < 0.05 compared with the DCI group.

Table options

3.2. Clinical outcomes

The VAS score for neck pain decreased significantly from 57.4 ± 15.8 to 11.6 ± 10.9 in the DCI group and from 59.7 ± 16.7 to 13.2 ± 11.7 in the ACDF group (both $p < 0.05$, Table 2). The VAS score for arm pain decreased significantly from 69.5 ± 19.7 to 17.2 ± 9.6 in the DCI group and from 70.3 ± 17.5 to 19.5 ± 10.2 in the ACDF group (both $p < 0.05$). The SF-36 score increased significantly from 28.4 ± 6.3 to 46.6 ± 8.7 in the DCI group and from 29.5 ± 7.2 to 43.8 ± 9.1 in the ACDF group (both $p < 0.05$). The JOA score increased significantly from 9.4 ± 2.1 to 14.8 ± 1.8 in the DCI group and from 9.8 ± 2.2 to 14.9 ± 2.1 in the ACDF group (both $p < 0.05$). There were no significant differences between the two groups in the SF-36, VAS, and JOA scores at the final follow-up (all $p > 0.05$). The NDI score decreased significantly from 19.8 ± 7.2 to 5.8 ± 2.9 in the DCI group and from 21.8 ± 6.9 to 10.2 ± 3.4 in the ACDF group (both $p < 0.05$). There was a significant difference in the change in NDI score at the final follow-up between the DCI and ACDF groups ($p < 0.05$). Using Odom's criteria, 84.5% of patients in the DCI group and 81.0% of patients in the ACDF group rated their level of satisfaction with the surgery as excellent or good, which was not a significant difference between groups ($p > 0.05$).

3.3. Radiological outcomes

In the DCI group, the overall ROM increased significantly from 41.1 ± 12.6° before surgery to 47.5 ± 19.8° at the final follow-up ($p < 0.05$, Table 3). In the ACDF group, the overall ROM decreased significantly from 43.5 ± 11.2° before surgery to 35.8 ± 17.6° at the final follow-up ($p < 0.05$). In the DCI group, there was a significant difference between the segmental ROM before surgery and at the last follow-up at the treated level, but not at the cephalad

and caudal levels. In the ACDF group, the segmental ROM at the treated level decreased significantly from 7.8 ± 4.1° to 0.8 ± 0.7° ($p < 0.05$), but there was no significant difference between the segmental ROM before surgery and at the last follow-up at the cephalad and caudal levels. The preoperative DHI at the treated level was 6.1 ± 1.3 mm in the DCI group and 6.2 ± 1.2 mm in the ACDF group. At the final follow-up, the DHI was significantly increased to 7.2 ± 1.1 mm in the DCI group and 7.3 ± 1.4 mm in the ACDF group (both $p < 0.05$). There was no significant difference in the change in DHI at the final follow-up between the DCI and ACDF groups ($p > 0.05$). Two patients in the ACDF group developed pseudarthrosis, both of which were asymptomatic fibrous non-unions that required no further treatment. The overall fusion rate was 94.9%. Radiological evidence of ASD was observed in 6/42 patients (14.3%) in the ACDF group and 5/39 patients (12.8%) in the DCI group, which was not a significant difference between groups ($p > 0.05$). A typical case of DCI arthroplasty is shown in Fig. 4.

Table 3.

Radiological outcomes of patients in the DCI and ACDF groups

	DCI group (n = 39)	ACDF group (n = 42)
Preoperative overall ROM, °	41.1 ± 12.6	43.5 ± 11.2
Final follow-up overall ROM, °	47.5 ± 19.8[*]	35.8 ± 17.6[*#]
Preoperative segmental ROM, °		
Treated	7.2 ± 3.8	7.8 ± 4.1
Cephalad	8.7 ± 4.7	8.9 ± 5.1
Caudal	6.5 ± 4.3	6.6 ± 4.9
Final follow-up segmental ROM, °		
Treated	8.9 ± 4.4[*]	0.8 ± 0.7[*#]
Cephalad	8.8 ± 5.1	9.2 ± 5.3
Caudal	6.7 ± 4.7	6.8 ± 5.1
Preoperative DHI, mm	6.1 ± 1.3	6.2 ± 1.2
Final follow-up DHI, mm	7.2 ± 1.1[*]	7.3 ± 1.4[*]
Adjacent segment degeneration, % (n)	12.8 (5/39)	14.3 (6/42)

ACDF = anterior cervical discectomy and fusion, DCI = dynamic cervical implant, DHI = disc height, ROM = range of motion.

*

$p < 0.05$ compared with preoperative.

#

$p < 0.05$ compared with the DCI group.

Table options

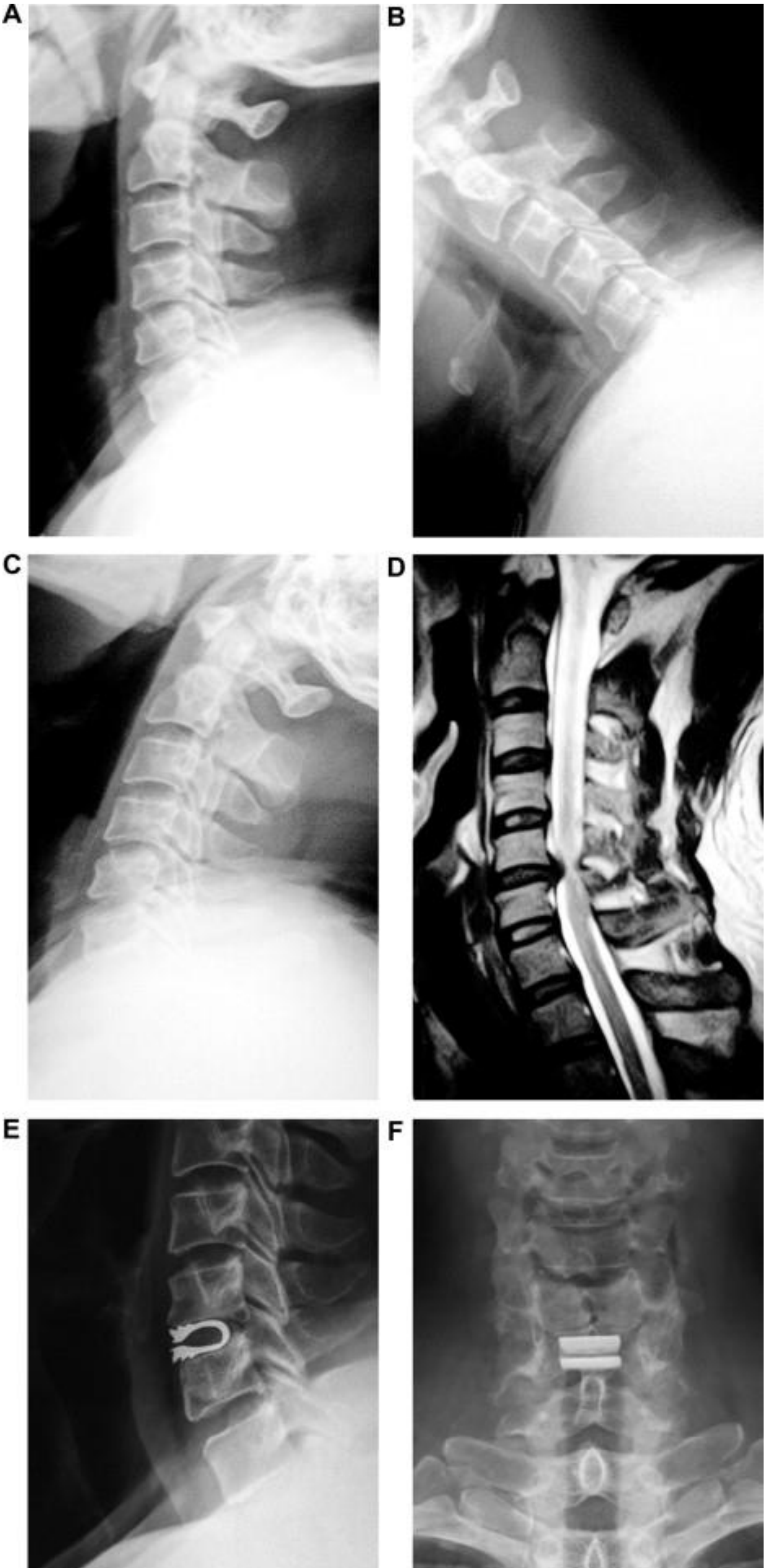
A
B
C
D
E
F

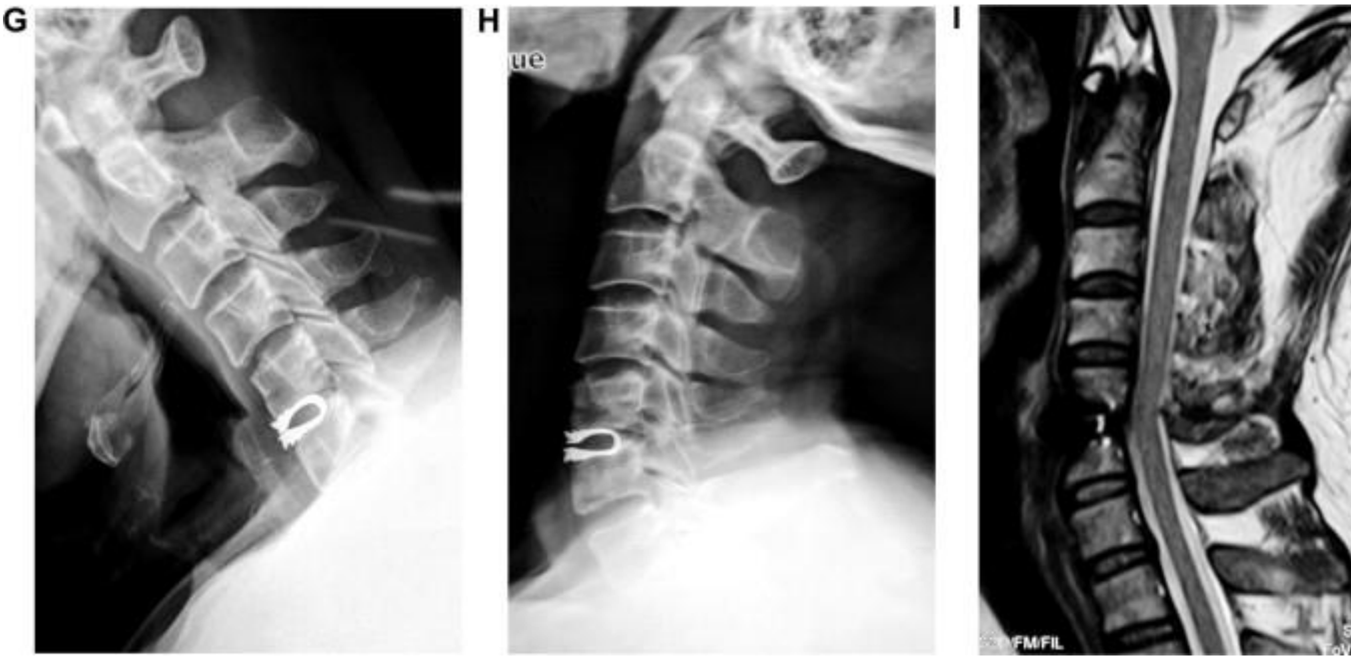

Fig. 4.

Illustrative patient. A 47-year-old man presented with a 2 year history of progressive numbness in both hands and weakness in all four extremities. (A) The preoperative disc height at C5–C6 was 6.2 mm on the lateral radiograph. (B, C) The preoperative range of motion (ROM) at C4–C5, C5–C6, and C6–C7 was 7.9°, 7.3°, and 7.5° on flexion-extension lateral radiographs, respectively. (D) Preoperative midsagittal T2-weighted MRI showed disc herniation at C5–C6, with Miyazaki grade II degeneration at C4–C5 and grade I at C6–C7. (E, F) Postoperative lateral and posteroanterior radiographs show the dynamic cervical implant (DCI; Scient'x, Villers-Bretonneux, France) in a good position. (G, H) The ROM at C4–C5, C5–C6, and C6–C7 was 8.1°, 7.7°, and 8.1°, respectively, on flexion-extension lateral radiographs at 24 months after DCI arthroplasty. (I) T2-weighted midsagittal MRI at 24 months after surgery showed no change in disc degeneration at the caudal level and worsening of disc degeneration at the cephalad level (from grade II to grade III).

Figure options

3.4. Complications

There were no patients with heterotopic ossification around the DCI. Anterior migration of the prosthesis by 2 mm was detected in one patient in the DCI group at the 12 month follow-up. This was caused by a deficiency in the endplate milling process. This patient did not develop neurological or vascular complications or dysphagia. The prosthesis was noted to have regained stability at the 18 month follow-up, and continued to be stable until the most recent follow-up at 49 months after surgery. Cage subsidence of

more than 1 mm was observed in two patients in the ACDF group at the final follow-up. Prosthesis subsidence of more than 1 mm was observed in two patients in the DCI group after 9 and 12 months. The causes of prosthesis subsidence were not identified, but these prostheses were noted to have regained stability at later follow-up. No other complications were observed in either group.

4. Discussion

ACDF is an effective and safe procedure for the surgical treatment of patients with radiculopathy and myelopathy. The goals of ACDF are to decompress the neural elements, provide permanent segmental stabilization, maintain the physiological lordosis, and preserve the anatomical disc-space height. However, increased motion and increased intradiscal pressure have been reported in the untreated levels adjacent to fused levels [19] and [20]. Some investigators have postulated that these changes may lead to an increased risk of ASD [21], [22] and [23]. ACDF has a high rate of clinical success for the treatment of cervical DDD, but the rigid fixation may result in ASD. Hilibrand et al. reported that approximately 25% of patients who underwent single-level ACDF developed ASD within 10 years [22] and [23].

The limitations and problems associated with ACDF have led some investigators to explore motion-preserving surgery such as artificial cervical disc arthroplasty [9], [10],[11], [24] and [25]. DCI is a new type of implant that enables anterior decompression without cervical fusion, and is mainly used to treat cervical DDD. Compared with artificial cervical disc arthroplasty, which has been used for many years, DCI arthroplasty has the following theoretical advantages: (1) it can be adapted to a wider scope with relatively simple surgery; (2) the U-shaped structure absorbs vibrations; (3) it restricts excessive flexion, extension, and rotation, thereby protecting the small cervical joints; and (4) as there is no grinding of metal, polyethylene, or ceramic, there is no local or systemic reaction to debris. In the last two decades, DCI arthroplasty has been used to preserve motion, avoid the limitations of fusion, and allow patients to quickly return to routine activities, while providing sufficient stability to restore normal segmental kinematics, control abnormal motion, enable greater physiological load transmission, and reduce or eliminate ASD. This procedure avoids the morbidity associated with bone graft harvesting and anterior cervical plating, and prevents pseudarthrosis and adverse effects caused by cervical immobilization.

DCI arthroplasty has not been widely used and there are few reports describing this procedure in the literature. In this study, we compared the

clinical and radiological outcomes of DCI arthroplasty *versus* ACDF for the treatment of single-level cervical DDD. There were no significant differences in operation time or blood loss between the two groups. The VAS, SF-36, NDI, and JOA scores improved significantly after surgery in both the DCI and ACDF groups, and there was no significant difference between the two groups in the VAS, SF-36, and JOA scores at the final follow-up. However, the NDI scores were significantly lower in the DCI group than in the ACDF group at the final follow-up. According to the Odom criteria, 84.6% of patients in the DCI group and 81% of patients in the ACDF group had excellent or good clinical outcomes. The proportion of patients who reported their level of satisfaction with the surgery as excellent or good was not significantly different between the two groups.

One of the major concerns regarding ACDF is that it does not preserve the normal kinematics of the spine and might therefore result in ASD, which could eventually lead to the need for additional treatment. Theoretically, DCI arthroplasty should be associated with less stress at the adjacent levels, which may decrease the risk of ASD. However, this has not been shown in a randomized clinical trial. In this study, the segmental ROM at the treated level was significantly higher in the DCI group than in the ACDF group. In the DCI group, the treated segment was still mobile at the final follow-up, and there was no significant change in the ROM of the adjacent cephalad and caudal segments. At the final follow-up, the overall cervical ROM had increased significantly in the DCI group and decreased significantly in the ACDF group. The DHI at the treated level was significantly restored in both the DCI and ACDF groups in the mid-term, and there was no significant difference in the change in DHI between the two groups. MRI at the final follow-up showed new signs of degeneration adjacent to the treated segment in five patients (12.8%). The rate of ASD was similar after DCI arthroplasty and ACDF. We consider that there is still no definitive evidence for the hypothesis that DCI arthroplasty may be associated with a lower risk of adjacent level effects compared with instrumented fusion. To our knowledge, no long-term data for DCI arthroplasty are available in the current literature. Long-term, controlled studies are needed to support recommendations for the use of DCI arthroplasty to treat cervical DDD.

This study is limited by the relatively small number of patients. We did not compare the surgical outcomes in patients with cervical DDD who underwent DCI arthroplasty with patients who underwent artificial cervical disc arthroplasty. In addition, the mean follow-up period was too short to evaluate the long-term efficacy of DCI arthroplasty for the treatment of cervical DDD.

However, we feel that this study provides useful information regarding the surgical treatment of cervical DDD because there are currently few reports describing outcomes after DCI arthroplasty. Further large-scale, prospective,randomized studies with long-term follow-up periods are needed to overcome these limitations and definitively determine whether DCI arthroplasty has advantages over ACDF.

5. Conclusions

The results of this study do not show significant differences between DCI arthroplasty and ACDF for cervical DDD in terms of improvement in clinical symptoms, blood loss, operation time, or improvement in DHI; but DCI arthroplasty was associated with better postoperative NDI scores than ACDF. DCI arthroplasty also resulted in better overall cervical ROM and segmental ROM at the treated level than ACDF. Overall, the results show that DCI arthroplasty is an effective, reliable, and safe procedure for the treatment of cervical DDD. However, there is no definitive evidence that DCI arthroplasty has better intermediate-term outcomes than ACDF. Selection of suitable surgical candidates and determination of valid indications for operative treatment are very important.

Conflicts of interest/disclosures

The authors declare that they have no financial or other conflicts of interest in relation to this research and its publication.

Acknowledgements

The authors are grateful for the support of NSFC 81201380 and Hospital Project QN201104. The authors also gratefully acknowledge the Beijing Engineering Technology Center of Orthopedics Implantable Medical Device.

References

1.
  - [1]
  - G.W. Smith, R.A. Robinson
  - **The treatment of certain cervicalspine disorders by anterior removal of the intervertebral disc and interbody fusion**
  - J Bone Joint Surg Am, 40-A (1958), pp. 607–624
  - View Record in Scopus

  |

Citing articles (734)

2.

- [2]
- W.M. Yue, W. Brodner, T.R. Highland
- **Long-term results after anterior cervical discectomy and fusion with anterior plating:a 5 to 11-year radiologic and clinical follow-up study**
- Spine, 30 (2005), pp. 2138–2144
- View Record in Scopus
  |
  Full Text via CrossRef
  |
  Citing articles (139)

3.

- [3]
- J. Goffin, E. Geusens, N. Vantomme, *et al.*
- **Long-term follow-up after interbody fusion of the cervical spine**
- J Spinal Disord Tech, 17 (2004), pp. 79–85
- View Record in Scopus
  |
  Full Text via CrossRef
  |
  Citing articles (211)

4.

- [4]
- H.H. Bohlman, S.E. Emery, D.B. Goodfellow, *et al.*
- **Robinson anterior cervical discectomy and arthrodesis for cervical radiculopathy. Long-term follow-up of one hundred and twenty-two patients**
- J Bone Joint Surg Am, 75 (1993), pp. 1298–1307
- View Record in Scopus
  |
  Citing articles (461)

5.

- [5]
- Y. Chen, Z. He, H. Yang, *et al.*
- **Anterior cervical diskectomy and fusion for adjacent segment disease**
- Orthopedics, 36 (2013), pp. e501–e508

- View Record in Scopus
  |
  Full Text via CrossRef
  |
  Citing articles (4)

6.
- [6]
- Y. Gao, M. Liu, T. Li, *et al.*
- **A meta-analysis comparing the results of cervical disc arthroplasty with anterior cervical discectomy and fusion (ACDF) for the treatment of symptomatic cervical disc disease**
- J Bone Joint Surg Am, 95 (2013), pp. 555–561
- View Record in Scopus
  |
  Full Text via CrossRef
  |
  Citing articles (35)

7.
- [7]
- M. Matsumoto, E. Okada, D. Ichihara, *et al.*
- **Anterior cervical decompression and fusion accelerates adjacent segment degereration comparison with asymptomatic volunteers in a ten-year magnetic resonance imaging follow-up study**
- Spine, 35 (2010), pp. 36–43
- View Record in Scopus
  |
  Full Text via CrossRef
  |
  Citing articles (57)

8.
- [8]
- J.S. Schwab, D.J. Diangelo, K.T. Foley
- **Motion compensation associated with single-level cervical fusion:where does the lost motion go**
- Spine, 31 (2006), pp. 2439–2448
- View Record in Scopus
  |

Full Text via CrossRef
|
Citing articles (55)
9.
- [9]
- S.W. Kim, J.H. Shin, J.J. Arbatin, *et al.*
- **Effects of a cervical disc prosthesis on maintaining sagittal alignment of the functional spinal unit and overall sagittal balance of the cervical spine**
- Eur Spine J, 17 (2008), pp. 20–29
- View Record in Scopus
  |
  Full Text via CrossRef
  |
  Citing articles (48)
10.
- [10]
- J. Goffin, J. van Loon, F. Van Calenbergh
- **Cervical arthroplasty with the Bryan Disc:4-year results**
- Spine J, 6 (2006), pp. 62–63
- View Record in Scopus
  |
  Citing articles (7)
11.
- [11]
- G.E. Pickett, L.H. Sekhon, W.R. Sears, *et al.*
- **Complications with cervical arthroplasty**
- J Neurosurg Spine, 4 (2006), pp. 98–105
- View Record in Scopus
  |
  Full Text via CrossRef
  |
  Citing articles (107)
12.
- [12]
- G. Matgé, M. Eif, J. Herdmann, *et al.*
- **Dynamic cervical implant (DCITM): clinical results from an international multicenter prospective study**

- Paradigm Spine, 1 (2009), pp. 1–3
- View Record in Scopus
  |
  Citing articles (4)

13.
- [13]
- J.T. King Jr, M.S. Roberts
- **Validity and reliability of the Short Form-36 in cervical spondylotic myelopathy**
- J Neurosurg, 97 (2002), pp. 180–185
- View Record in Scopus
  |
  Full Text via CrossRef
  |
  Citing articles (38)

14.
- [14]
- H. Vernon, S. Mior
- **The Neck Disability Index: a study of reliability and validity**
- J Manipulative Physiol Ther, 14 (1991), pp. 409–415
- View Record in Scopus
  |
  Citing articles (1123)

15.
- [15]
- G.L. Odom, W. Finney, B. Woodhall
- **Cervical disk lesions**
- J Am Med Assoc, 166 (1958), pp. 23–28
- View Record in Scopus
  |
  Full Text via CrossRef
  |
  Citing articles (293)

16.
- [16]

- Yonenobu K, Abumi K, Nagata K, et al. Interobserver and intraobserver reliability of the Japanese Orthopedic Association scoring system for evaluation of cervical compression myelopathy. Spine 2001;26:1890–4; discussion 1895.
-

17.
- [17]
- M.R. Lim, F.P. Girardi, K. Zhang, *et al.*
- **Measurement of total disc replacement radiographic range of motion: a comparison of two techniques**
- J Spinal Disord Tech, 18 (2005), pp. 252–256
- View Record in Scopus
  |
  Citing articles (26)

18.
- [18]
- M. Miyazaki
- **Hong SW, Yoon SH, et a1. Reliability of a magnetic resonance imaging-based grading system for cervical intervertebral disc degeneration**
- J Spinal Disord Tech, 21 (2008), pp. 288–292
- View Record in Scopus
  |
  Full Text via CrossRef
  |
  Citing articles (36)

19.
- [19]
- Z.N. Irwin, A. Hilibrand, M. Gustavel, *et al.*
- **Variation in surgical decision making for degenerative spinal disorders**
- Part II: cervical spine. Spine, 30 (2005), pp. 2214–2219
- View Record in Scopus
  |
  Full Text via CrossRef
  |
  Citing articles (41)

20.
- [20]
- M. Gallucci, N. Limbucci, A. Paonessa, *et al.*

- **Degenerative disease of the spine**
- Neuroimaging Clin N Am, 17 (2007), pp. 87–103
- Article
  |
- Purchase PDF

|
View Record in Scopus
|
Citing articles (28)

1.
   - [21]
   - J.C. Bartolomei, N. Theodore, V.K. Sonntag
   - **Adjacent level degeneration after anterior cervical fusion: a clinical review**
   - Neurosurg Clin N Am, 16 (2005), pp. 575–587
   - Article
     |
   - Purchase PDF

|
View Record in Scopus
|
Citing articles (37)

2.
   - [22]
   - A.S. Hilibrand, J.U. Yoo, G.D. Carlson, *et al.*
   - **The success of anterior cervical arthrodesis adjacent to a previous fusion**
   - Spine, 22 (1997), pp. 1574–1579
   - View Record in Scopus
     |
     Full Text via CrossRef
     |
     Citing articles (109)

3.
   - [23]
   - A.S. Hilibrand, G.D. Carlson, M. Palumbo, *et al.*
   - **Radiculopathy and myelopathy at segments adjacent to the site of a previous anterior cervical arthrodesis**

- J Bone Joint Surg Am, 81 (1999), pp. 519–528
- View Record in Scopus
  |
  Citing articles (732)

4.
- [24]
- Mummaneni PV, Amin BY, Wu JC, et al. Cervical artificial disc replacement versus fusion in the cervical spine: a systematic review comparing long-term follow-up results from two FDA trials. Evid Based Spine Care J 2012;3:59–66.
-

5.
- [25]
- D. Coric, P.K. Kim, J.D. Clemente, *et al.*
- **Prospective randomized study of cervical arthroplasty and anterior cervical discectomy and fusion with long-term follow-up: results in 74 patients from a single site**
- J Neurosurg Spine, 18 (2013), pp. 36–42
- View Record in Scopus
  |
  Full Text via CrossRef
  |
  Citing articles (31)
