# Supplementary material for: Adjacent segment degeneration or disease after cervical total disc replacement: a meta-analysis of randomized controlled trials
Source: J Orthop Surg Res. 2018 Oct 3;13:244. doi: 10.1186/s13018-018-0940-9 (PMC6169069; doi:10.1186/s13018-018-0940-9)
Supplement: Supplementary file 2 — File S1. Original data of 11 included articles. (ZIP 12 mb) [file 13018_2018_940_MOESM2_ESM.zip › 11 included articles and original data referred in this article/11 included articles/28 Burkus, J K(US).pdf]

J Neurosurg Spine 21:516–528, 2014
©AANS, 2014

Clinical and radiographic analysis of an artificial cervical disc: 7-year follow-up from the Prestige prospective randomized controlled clinical trial

Clinical article

J. Kenneth Burkus, M.D.,[1] Vincent C. Traynelis, M.D.,[2] Regis W. Haid Jr., M.D.,[3] and Praveen V. Mummaneni, M.D.[4]

[1]Staff Physician, Spine Service, Wilderness Spine Services, Columbus; [3]Atlanta Brain and Spine Care, Atlanta, Georgia; [2]Department of Neurosurgery, Rush University Medical Center, Chicago, Illinois; and [4]Department of Neurological Surgery, University of California, San Francisco, California

Object. The authors assess the long-term safety and efficacy of cervical disc replacement with the Prestige Cervical Disc in a prospective, randomized, multicenter trial at 7 years of follow-up.

Methods. At 31 investigational sites, 541 patients with single-level cervical disc disease with radiculopathy were randomized to 1 of 2 treatment groups: 276 investigational group patients underwent anterior cervical discectomy and arthroplasty with the Prestige disc, and 265 control group patients underwent anterior cervical discectomy and fusion. Clinical outcomes included Neck Disability Index, the 36-Item Short-Form Health Survey, and neck and arm pain scores. Radiographs were assessed for angle of motion and fusion. Clinical and radiographic outcomes were evaluated preoperatively, intraoperatively, and at 1.5, 3, 6, 12, 24, 36, 60, and 84 months.

Results. Of the 541 patients treated, 395 patients (73%; 212 investigational and 183 control patients) completed 7 years of clinical follow-up. Significant improvements achieved by 1.5 months in both groups were sustained at 7 years. In the investigational group, mean Neck Disability Index improvements from preoperative scores were 38.2 and 37.5 at 60 and 84 months, respectively. In the control group, the corresponding means were 33.8 and 31.9. The differences between the investigational and control groups at the 60-month and 84-month periods were significant ($p = 0.014$ and 0.002, respectively). The overall rates of maintenance or improvement in neurological status in the investigational group were significantly higher: 92.2% and 88.2% at 60 months and 84 months, respectively, compared with 85.7% and 79.7% in the control group ($p = 0.017$ and 0.011, respectively). At 84 months, the percentage of working patients in the investigational group was 73.9%, and in the control group, 73.1%. Postoperatively, the implant effectively maintained average angular motion of 6.67° at 60 months and 6.75° at 84 months. Cumulative rates for surgery at the index level were lower ($p < 0.001$) in the investigational group (11 [4.8%] of 276) when compared with the control group (29 [13.7%] of 265) (based on life-table method), and there were statistical differences between the investigational and control groups with specific regard to the rate of subsequent revision and supplemental fixation surgical procedures. Rates for additional surgical procedures that involved adjacent levels were lower in the investigational group than in the control group (11 [4.6%] of 276 vs 24 [11.9%] of 265, respectively).

Conclusions. Cervical disc arthroplasty has the potential for preserving motion at the operated level while providing biomechanical stability and global neck mobility and may result in a reduction in adjacent-segment degeneration. The Prestige Cervical Disc maintains improved clinical outcomes and segmental motion after implantation at 7-year follow-up. Clinical trial registration no. NCT00642876 (ClinicalTrials.gov).
(http://thejns.org/doi/abs/10.3171/2014.6.SPINE13996)

Key Words • **cervical spine** • **degenerative disc disease** • **cervical disc arthroplasty** • **Prestige Cervical Disc replacement**

Clinical and radiographic outcomes have been reported from several prospective studies of patients undergoing cervical disc replacement with a metal-on-metal implant.[5,6,19,20] An early clinical prospective observational cohort study of 15 patients reported successful clinical and radiographic outcomes in the treatment of symptomatic cervical radiculopathy.[25] An optimized ball-and-trough designed implant (Prestige Disc, Medtronic) was then developed and used in a prospective randomized trial.[19] Fifty-five patients were enrolled in this pilot study at 4 investigational sites. At the 24-month follow-up, Neck Disability Index (NDI) scores showed improvement that favored the disc replacement group

Abbreviations used in this paper: FSU = functional spinal unit; IDE = investigational device exemption; NDI = Neck Disability Index; PCS = Physical Component Summary; RCT = randomized controlled trial; SF-36 = 36-Item Short Form Health Survey.

over the control group, and motion was maintained at the treated level. In 2002, a large-scale, prospective, randomized, multicenter study was initiated in which this metal-on-metal disc prosthesis was compared with anterior cervical discectomy and fusion in patients who had symptomatic single-level cervical degenerative disc disease.[15] Investigators reported the interim data from this randomized controlled trial (RCT) in previous publications.[4,15] These early clinical reports have shown that the prosthesis maintained segmental spinal motion and was associated with improved neurological success, clinical outcomes, and reduced secondary surgical procedures when compared with anterior cervical discectomy and fusion. These improvements in outcomes were similar to other early clinical and radiographic findings for the Bryan Cervical Disc (Medtronic),[8] the ProDisc-C (Synthes Spine Co.),[16] Kineflex|C (SpinalMotion),[5] and the Mobi-C (LDR)[3,18] devices. At 24 months in these cervical disc replacement RCTs, the postoperative data showed improvement in all clinical outcome measures, and patients treated with the artificial disc had a statistically greater overall success rate.[12,23]

The safety and efficacy of cervical disc prostheses beyond the initial 24-month follow-up has not been as widely reported. Reports from RCTs with 4 years of follow-up have been published for the Bryan disc[21] and with 5 years of follow-up for the ProDisc-C.[27] The final 2-year and interim 5-year data from the Prestige disc RCT have been published.[4,15] The purpose of this paper is to report the final 7-year clinical and radiographic outcomes from the Prestige disc RCT.

Methods

Study Design

This prospective, randomized, nonblinded study was conducted under an approved investigational device exemption (IDE). Patients in the IDE trial were followed in this FDA-regulated postapproval study for an additional 5 years, resulting in a total of 7 years of follow-up. Institutional review board approval was obtained from all participating centers, and informed consent was obtained for all patients enrolled in the follow-up studies. The clinical trial registration number for this study is NCT00642876 (www.ClinicalTrials.gov).

Between October 2002 and August 2004, 541 patients were enrolled at 31 investigational sites and underwent surgery. The patients were randomly assigned to 1 of 2 treatment groups: the investigational group received the Prestige disc and the control group underwent an interbody fusion using allograft with plate fixation. Data were collected preoperatively, intraoperatively, and at 1.5, 3, 6, 12, 24, 36, 60, and 84 months postoperatively. Adverse events and secondary surgeries were recorded at each follow-up visit.

Patient Demographics

The 2 treatment groups were similar demographically, and there were no statistically significant differences ($p < 0.05$) for the variables of age, sex, smoking, or work status (Table 1). All patients were between the ages of 22 and 73 years and had symptomatic degenerative cervical disc disease between the C3–4 or C6–7 levels. For at least 6 weeks before their surgery, all patients had neck and arm pain that was recalcitrant to nonoperative treatment modalities, such as physical therapy, reduced activities, and antiinflammatory medications.

All patients were considered candidates for a single-level anterior cervical decompression and interbody fusion and had plain radiographic findings that documented single-level cervical disc disease and at least one additional confirmatory neuroradiographic study, such as MRI or CT-enhanced myelography that showed findings consistent with clinical findings and complaints.

Patients were excluded from the study if they had cervical spinal conditions other than single-level symptomatic degenerative disc disease or evidence of instability. Other exclusion criteria were symptomatic disc disease at level C2–3 or C7–T1, a history of discitis, or a medical condition that required medication, such as steroids or nonsteroidal antiinflammatory medications that could interfere with fusion.

Patient Follow-Up

A total of 541 patients were treated in the RCT; 276 were assigned to the investigational group, and 265 patients were assigned to the control treatment group (Fig. 1). One center declined to participate in the long-term follow-up study after the initial 24-month evaluation period was completed. As a result of the nonparticipating site, 8 patients (4 each in the investigational group and control group) were excluded from this study, leaving 533 patients who were eligible for the FDA postapproval follow-up.

Clinical Outcome Measures

Overall success was the primary clinical outcome measure for this clinical trial. A patient's outcome was considered an overall success if all of the following conditions were met: 1) postoperative NDI score improvement of at least a 15-point increase from preoperative score; 2) maintenance or improvement in neurological status; 3) disc height success (the functional spinal unit [FSU] was measured to assess for any loss of disc height due to subsidence); 4) no serious adverse event classified as implant associated or implant/surgical procedure associated; and 5) no additional surgical procedure classified as a "failure."

When overall success was assessed, it was apparent that in some instances the radiographic images did not allow clear visualization of the FSU, and, therefore, adequate disc height measurements were unobtainable. For this reason, overall success rates were computed with and without inclusion of disc height success as a component of overall success. When disc height success was included as a component of overall success, patients in whom the radiographic images did not permit disc height measurement were excluded from the analysis.

Secondary and additional clinical outcome measures included the Physical Component Summary (PCS) of the 36-Item Short Form Health Survey (SF-36), neck and arm pain scores (100-point scale, which was the product of

TABLE 1: Patient demographic data

Variable	Investigational Group	Control Group	p Value*
no. of patients	276	265	
age (yrs)			
mean	43.3	43.9	0.435
range	25–72	22–73	
mean weight (lbs)	181.7	184.7	0.389
male (%)	46.4	46.0	1.000
workers' compensation (%)	11.6	13.2	0.603
litigation (%)	10.9	12.1	0.687
alcohol use (%)	43.5	53.2	0.025
tobacco use (%)	34.4	34.7	1.000
work status (%)	65.9	62.6	0.473

* For continuous variables, p values are from ANOVA, and for categorical variables, they are from the Fisher's exact test.

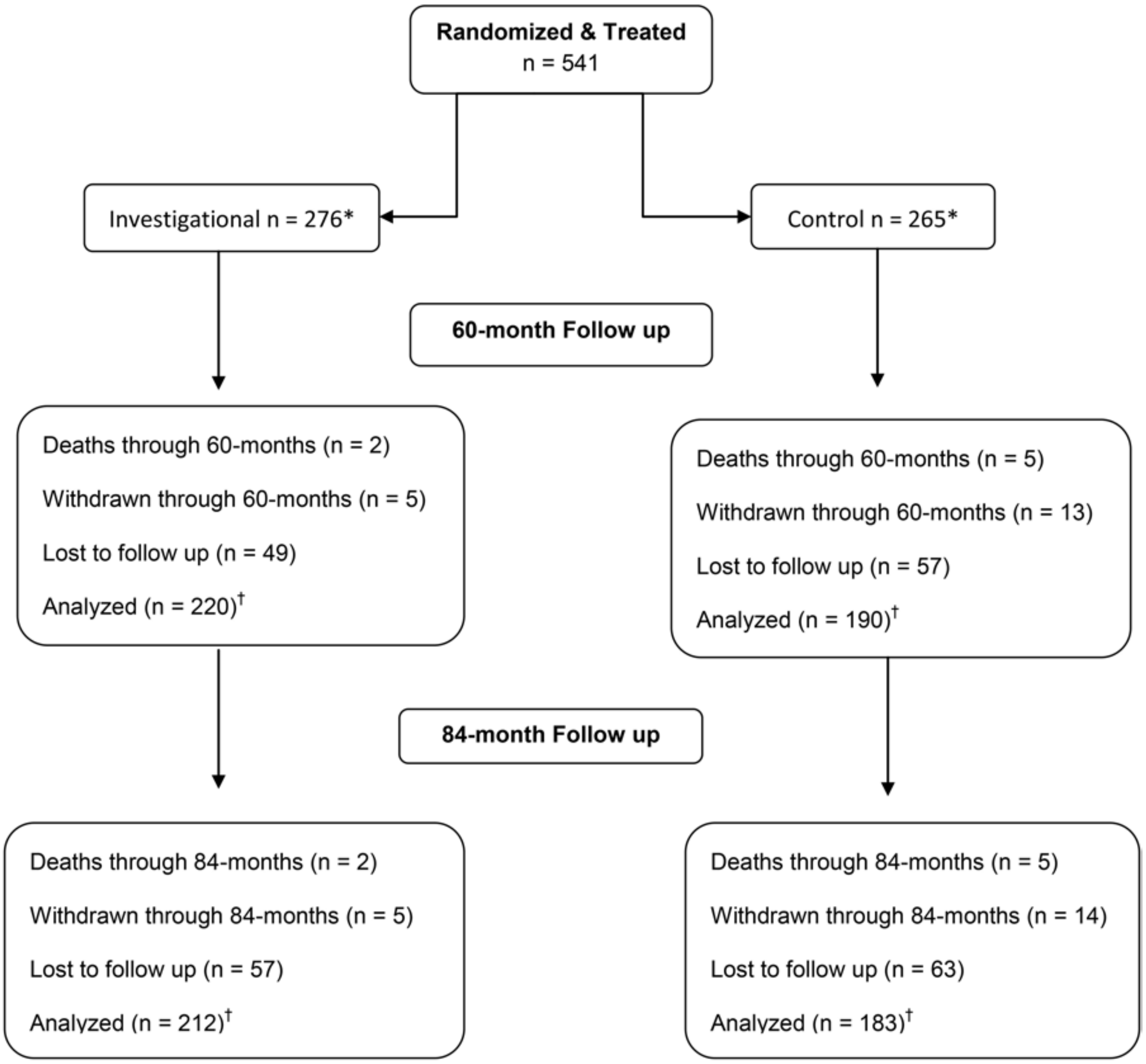

FIG. 1. Flowchart of participants throughout the 7-year study. *Cumulative adverse event rates include adverse events from patients who withdrew from the study. †Patients had at least 1 complete outcome measure.

duration [1- to 10-point scale] multiplied by intensity [1- to 10-point scale]), and return-to-work status.

Radiographic Assessments

Plain radiographic studies were obtained preoperatively, intraoperatively, and at 1.5, 3, 6, 12, 24, 36, 60, and 84 months postoperatively. Neutral anteroposterior and lateral radiographs and dynamic flexion-extension lateral radiographs were obtained at each study point. Sagittal plane angulation motion was measured on dynamic lateral radiographs using Cobb's technique. Subsidence was measured by comparing the FSU height from 1.5 months after surgery. This calculation required that the entire vertebral body above and below the index surgical level could be visualized. For centralization of assessments and measurements, radiographs taken at each investigational site were evaluated at the same core-imaging laboratory (SYNARC) through all study periods. Two independent radiographic reviewers and an adjudicator determined radiographic findings in both treatment groups. Some radiographs could not be interpreted by radiologists because the prominent shoulders obscured critical portions of the radiographic images. For these reasons, the numbers of complete radiographic assessments varied from the numbers of clinical assessments at each of the study's follow-up intervals.

Adverse Events

An adverse event was defined as any clinically adverse sign, symptom, syndrome, or illness that occurred or worsened during either the operative or postoperative observation periods, regardless of causality, that was not being measured otherwise in the study. The adverse event information recorded was based on the following: 1) signs or symptoms detected during the physical examination, 2) the clinical evaluation of the subject, 3) the subject interview, and 4) the medical charts monitored during the study. Adverse events were collected for the duration of the entire study.

Secondary Surgical Procedures

Secondary surgical procedures at the level of the index procedure were classified according to the IDE trial protocol as revisions, removals, supplemental fixations, or reoperations. In this investigation, a revision surgery was defined as any procedure that adjusts or modifies the original implant configuration (implant repositioning). A removal surgery was defined as a procedure in which 1 or more components of the original implant was removed and replaced with a different type of implant. For example, removal of the Prestige implant and replacement with an interbody cage and anterior plate would be classified as a removal surgery. A supplemental fixation procedure, such as posterior wiring or plating, provided additional stabilization to the index surgical site. This definition included the application of an external bone growth stimulator as a supplemental fixation procedure; however, data are summarized separately in this paper. A secondary surgical procedure was classified as a reoperation if the procedure was carried out at the index level and was not classified as a revision, removal, or supplemental fixation. An example of a reoperation would be a posterior foraminotomy to relieve persistent nerve root pressure at the index surgical level.

Statistical Analysis

Statistical comparisons were based primarily on the observed and recorded follow-up data. Missing values for patients who were lost to follow-up were not imputed. For outcomes of the patients requiring an additional surgical procedure that was classified as a failure (removal, revision, or supplemental fixation), the observations immediately before the second surgery were carried over for all future evaluation periods.

For statistical comparisons of demographic differences between the groups, ANOVA was used for continuous variables and the Fisher's exact test was used for categorical data. Assessment of the statistical significance of postoperative improvement from preoperative values within each treatment group was performed using a paired t-test.

For the binary outcome variables, such as overall success, the success rate of the investigational group and the control group were compared using a Z-test with the standard deviation derived using the Farrington and Manning[7] method. Analysis of covariance was used with the preoperative score as the covariate for comparing postoperative continuous measurements and improvements such as NDI. One-sided p values were reported for most clinical outcomes, except for additional surgical procedures and adverse events. Time-to-event analysis was performed for comparing adverse events between the treatment groups, and the log-rank test was used to determine statistical significance. A p value < 0.05 was used to evaluate statistical significance without adjusting for multiple comparisons. The primary study objective for the postapproval study was to assess noninferiority (with a 10% margin) in overall success at 7 years comparing the investigational group to the control group. If noninferiority was established, superiority was also assessed. Noninferiority p values are not presented in this paper.

Results

Follow-up rates at 60 and 84 months were 79.7% and 76.8%, respectively, for the investigational group and 71.7% and 69.1%, respectively, for the control group (Fig. 1). The overall follow-up rate at 84 months was 73% (395 of 541 patients). There were 212 patients in the investigational group and 183 in the control group. Follow-up rates were based on the number of patients who had at least 1 complete outcome measure at that interval. Note that more patients reached the 60-month follow-up time point by 2012 than reported in our previous publication.[4]

Overall Success

At 60 and 84 months, noninferiority in overall success comparing the investigational and control groups was confirmed. At 60 months, the overall success rates with the FSU measure were 71.3% for the investigational group and 65.2% for the control group, a 6.1% difference that was not statistically significant ($p = 0.129$) (Fig. 2). When the

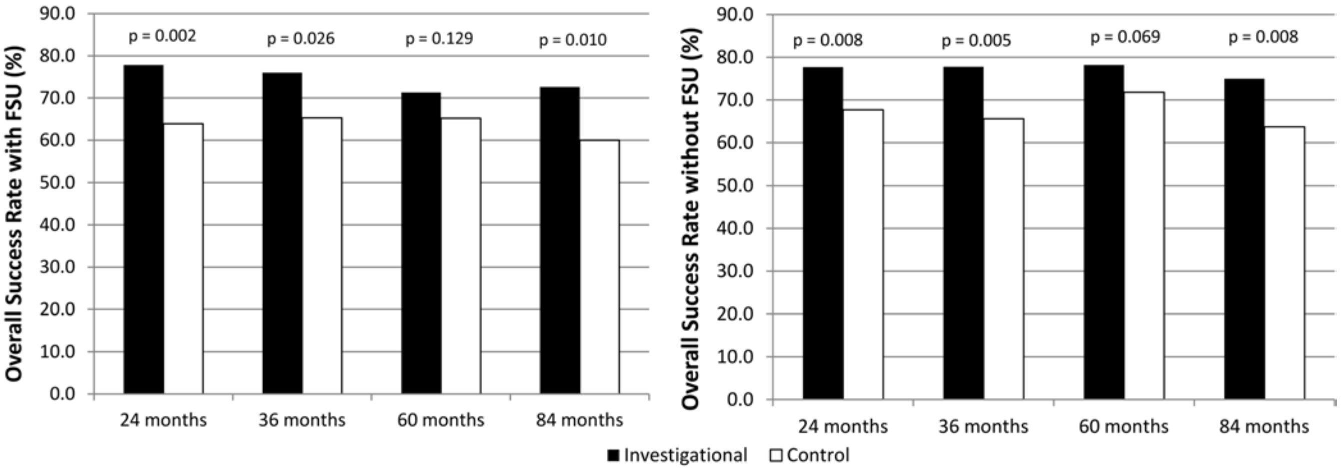

Fig. 2. Overall success with **(left)** and without **(right)** disc height.

FSU measure was excluded, the overall success rates at 60 months were 78.2% and 71.8% for the investigational and control groups, respectively, a 6.4% difference that was also not statistically significant (p = 0.069). However, at 84 months the overall success rates with and without the FSU measure were both significantly higher in the investigational group. Overall success rates with the FSU measure were 72.6% and 60.0% for the investigational and control groups, respectively (p = 0.010), and were 75.0% and 63.7% without the FSU measure for the investigational and control groups, respectively (p = 0.008).

NDI Scores

The NDI questionnaire measures the level of pain and disability associated with various activities. The NDI scores improved significantly in both groups from the preoperative scores by 1.5 months, and these improvements were maintained through 7 years (p < 0.001). For the investigational group, average NDI scores improved 37.6 points at 84 months from a mean preoperative score of 55.7. For the control group, average NDI scores improved 32.6 points at 84 months from a mean preoperative score of 56.4. These improvements were similar to those at 60 months, which were 38.2 points and 34.7 points for the investigational and control groups, respectively. At both the 60-month and 84-month periods, there were significant between-group differences in favor of arthroplasty (p = 0.014 and 0.002, respectively) (Table 2).

The NDI success criterion is based on the preoperative NDI score. A 15-point or greater NDI score improvement after surgery was required to be considered a successful outcome. The 2 groups demonstrated similar rates of NDI success at 60 and 84 months, which were 85.4% and 83.4%, respectively, for the investigational group and 84.8% and 80.1%, respectively, for the control group. There were no significant between-group differences in NDI success rates at 60 months (p = 0.293) or at 84 months (p = 0.109).

Arm Pain

Arm pain scores improved significantly in all groups from the preoperative scores by 1.5 months, and these improvements were maintained through 7 years (p < 0.001). For the investigational group, average arm pain scores improved 46.4 points at 84 months, from a mean preoperative score of 59.1. The control group showed comparable improvement in average arm pain scores improving 47.4 points from a preoperative score of 62.4. There were no significant between-group differences in arm pain scores at 60 months (p = 0.092) or at 84 months (p = 0.174) (Table 2).

Neck Pain

Neck pain scores improved significantly in both groups from the preoperative scores by 1.5 months, and these improvements were maintained through 7 years (p < 0.001). For the investigational group, average neck pain scores improved 55.1 points at 84 months, from a mean preoperative score of 68.2. For the control group, average neck pain scores improved 49.9 points at 84 months, from a preoperative score of 69.3. Neck pain was significantly lower (p = 0.033) at 60 months in the investigational group (12.7) than in the control group (16.9), and was also significantly lower (p = 0.004) at 84 months in the investigational group (13.1) than in the control group (19.4) (Table 2).

SF-36 PCS

The SF-36 measures specific health concepts related to physical functioning, social functioning, and health perceptions. The SF-36 PCS scores improved significantly in both groups from preoperative scores by 6 months (p < 0.001), which was the first postoperative period for SF-36 evaluation, and these improvements were maintained through 7 years. There were no significant between-group differences in SF-36 PCS at 60 months (45.8 points and 44.7 points for the investigational and control groups, respectively) (p = 0.098). At 84 months, the SF-36 PCS score for the investigational group was 45.1 points compared with 43.2 points in the control group (p = 0.017) (Table 2).

Neurological Success

Neurological status of the patients was determined by measuring 3 objective clinical findings: motor func-

TABLE 2: Comparisons of functional, pain, and quality of life outcomes

	Investigational Group		Control Group		
Variable	No. of Patients	Mean ± SD	No. of Patients	Mean ± SD	p Value*
NDI					
preop	276	55.7 ± 14.8	264	56.4 ± 15.9	
24 mos	253	20.0 ± 21.4	220	22.4 ± 21.5	0.140
36 mos	197	18.9 ± 21.5	160	24.0 ± 20.4	0.008
60 mos	219	17.5 ± 20.4	188	21.7 ± 20.7	0.014
84 mos	211	18.1 ± 20.0	181	23.8 ± 21.6	0.002
arm pain					
preop	276	59.1 ± 29.4	264	62.4 ± 28.5	
24 mos	253	13.9 ± 24.6	220	14.2 ± 24.3	0.521
36 mos	197	12.9 ± 24.4	161	15.6 ± 23.1	0.141
60 mos	218	10.6 ± 21.5	189	13.6 ± 23.5	0.092
84 mos	210	12.7 ± 24.1	181	15.0 ± 24.9	0.174
neck pain					
preop	276	68.2 ± 22.7	264	69.3 ± 21.5	
24 mos	253	15.6 ± 24.4	220	16.6 ± 1.62	0.368
36 mos	197	14.2 ± 23.0	161	19.6 ± 26.1	0.018
60 mos	217	12.7 ± 22.4	189	16.9 ± 24.4	0.033
84 mos	210	13.1 ± 23.3	181	19.4 ± 24.8	0.004
SF-36 PCS					
preop	275	31.9 ± 7.0	263	32.0 ± 7.5	
24 mos	248	44.6 ± 12.2	218	44.4 ± 12.0	0.277
36 mos	197	45.6 ± 11.9	160	43.7 ± 11.5	0.032
60 mos	217	45.8 ± 11.7	187	44.7 ± 11.9	0.098
84 mos	209	45.1 ± 12.0	179	43.2 ± 12.1	0.017

* The p values are for comparisons between the treatment groups from the ANCOVA with preoperative score as the covariate.

tion, sensory function, and deep tendon reflexes. Neurological success for each of these 3 objective findings was based on postoperative maintenance or improvement in condition compared with preoperative status. Overall neurological status success was determined by maintenance or improvement in all 3 clinical findings.

In the investigational group, overall neurological success (maintenance or improvement) rates were high, exceeding 88% at all follow-up intervals (Fig. 3). The overall success rates of neurological status in the investigational group were 92.2%, and 88.2%, respectively, at 60 and 84 months, compared with 85.7% and 79.7% in the control group (p = 0.017 and 0.011, respectively. The neurological success rate may have been influenced by the difference in surgical technique between the study groups. The arthroplasty surgical technique required an extensive posterior and posterolateral decompression across the disc space. In the arthroplasty group, the entire posterior annulus and posterior longitudinal ligament were resected. Uncovertebral joints were partially removed, enlarging the neuroforamina. This extensive dissection and decompression was not required in the fusion control group. Indirect enlargement of the neuroforamina through intradiscal distraction was permitted in the control group. Assuming missing-equals-failure for patients lost to follow-up, the differences between the treatment groups in neurological success remains in favor of the investigational arthroplasty group (Table 3).

Work Status

Before surgery, 65.9% of the investigational group was working compared with 62.6% of the control group. At 84 months, the percentage of working patients in the investigational group was 73.9% and in the control group, 73.1%, reflecting no difference between the groups. Statistical tests were not done at each follow-up interval to determine if differences in working rates were statistically significant; instead, time to return to work was compared between the 2 treatment groups using the Kaplan-Meier approach. The difference in days after surgery for return to work between the 2 groups (p = 0.022, Wilcoxon test) favored an earlier return to work in the investigational group. A Cox proportional hazard model, adjusting for preoperative work status, was also used to evaluate differences in days for return to work and again demonstrated an earlier return to work in the disc arthroplasty group (p = 0.033).

Radiographic Outcomes

The radiographic outcomes were based on measure-

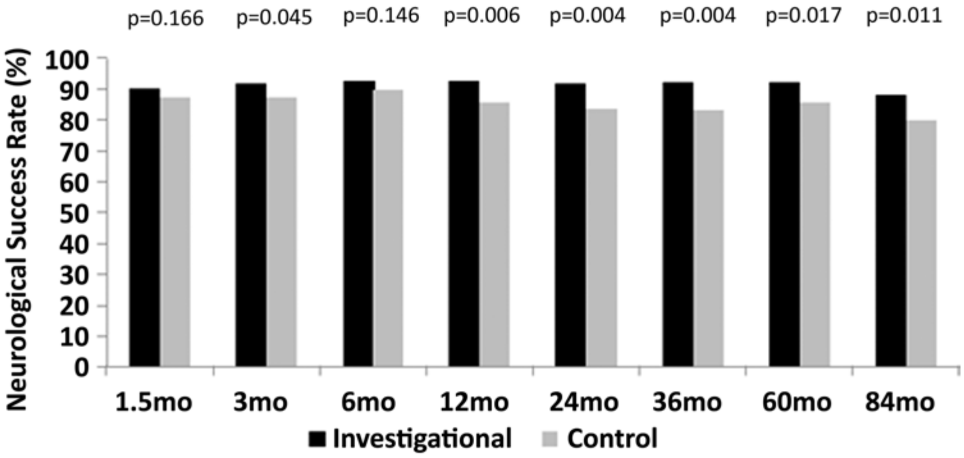

Fig. 3. Neurological success rates in the investigational group and control group. The p values are one-sided from normal approximation with standard error derived from the Farrington-Manning method.

ments recorded by 2 independent radiologists. In the control group, sagittal angular motion was restricted after surgery. The fusion success rate was 97.8% (131 of 133) at 60 months and 96.9% (127 of 131) at 84 months.

In the investigational group, the Prestige implant effectively maintained sagittal angular motion averaging 6.67° at 60 months and 6.75° at 84 months after surgery (Figs. 4 and 5). Preoperatively, sagittal angulation at the target disc space averaged 7.55°. Sagittal angular motion greater than 4° and less than or equal to 20° was seen in 70.5% (146 of 207) of patients at 60 months and in 68.8% (141 of 205) of patients at 84 months (Figs. 6 and 7). In the investigational patients, bridging bone was observed in 13 (6.2%) of 209 patients with complete radiographic follow-up at 60 months and in 20 (10.0%) of 201 patients at 84 months, compared with 2 (0.8%) of 250 patients at 24 months.

Functional spinal unit failure, a surrogate measure of subsidence, was defined as a decrease of more than 2 mm in FSU height from 1.5 months after surgery. This calculation required that the entire vertebral body above and below the index surgical level was able to be visualized, which was not possible in all of the patients with complete sets of radiographs. In those patients with adequate visualization of the vertebral bodies, FSU failure was observed in 12 (7.3%) of 165 patients at 60 months and 7 (4.2%) of 166 patients at 84 months. As a comparison, the corresponding FSU failure rates in the control group were 6 (4.5%) of 134 patients at 60 months and 4 (3.1%) of 127 patients at 84 months. There was no statistically significant difference in those rates between the 2 treatment groups at 60 months ($p = 0.844$) or 84 months ($p = 0.683$).

At the 84-month follow-up examination, 1 patient (of 204 [0.5%]) showed radiographic evidence of disc implant migration. No disc migrations were observed at any previous follow-up interval. At 84 months, 5 patients (of 204 [2.4%]) showed evidence of a broken or fractured Prestige screw. In the control group, there was no evidence of graft migration and no evidence of broken or fractured implants or screws.

Adjacent-Segment Angular Motion

Adjacent-level disc degeneration and ossification were not specific data points captured in this study; however, the independent radiologists reviewed adjacent-segment angular motion patterns. At 6 weeks postoperatively, motion at the superior adjacent segment was 10.6° and 9.7° for the investigational and control groups respectively ($p = 0.041$). There were no other significant between-group differences in motion at adjacent levels at any other time point ($p > 0.097$). In the investigational group, at the adjacent disc spaces, sagittal angular motion was effectively maintained above the implant, averaging 10.7° at 60 months (208 patients) and 11.4° at 84 months after surgery (206 patients). In the control group, similar motion patterns were seen at the superior disc space averaging 10.7° at 60 months (179 patients) and remained un-

TABLE 3: Comparison of observed neurological success rates*

Period	Neurological Success Determination	Neurological Success (%)	
		Investigational	Control
24 mos	observed	91.6	83.6
	imputing LTFU as failures	83.3	69.4
60 mos	observed	92.2	85.7
	imputing LTFU as failures	73.2	61.1
84 mos	observed	88.2	79.7
	imputing LTFU as failures	67.4	54.7

* Rates were calculated by imputing patients who were lost to follow-up as failures. LTFU = lost to follow-up.

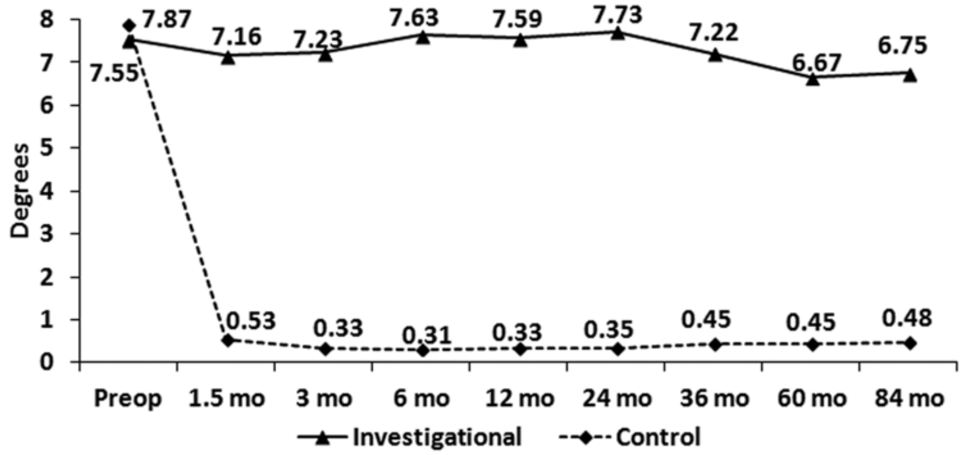

Fig. 4. Mean angular motion at the index surgical level in the investigational group and control group.

changed at 10.7° at 84 months after surgery (170 patients). At the disc space level below the index surgery, in the investigational group, sagittal angular motion averaged 8.0° at 60 months (126 patients) and 7.7° at 84 months after surgery (134 patients). In the control group, sagittal angular motion averaged 8.4° at 60 months (111 patients) and 8.5° at 84 months (113 patients) after surgery.

Adverse Events

Adverse events reported by both groups are included in Table 4. There were 259 (97.7%) of 276 investigational group patients and 232 (94.5%) of 265 control group patients who reported at least 1 adverse event ($p = 0.958$) through completion of the study. Excluding nonunion, there were only 2 categories of adverse event in which the occurrence rates differed between the treatment groups. Investigational patients reported fewer spinal events than control patients (20.9% vs 38.9%, $p < 0.001$), whereas the control patients reported fewer urogenital events than the investigational patients (20.1% vs 12.2%, $p = 0.024$).

Dysphagia and Dysphonia

Complaints of dysphagia and dysphonia were identified and recorded as adverse events in both treatment groups. At the 84-month follow-up, the cumulative rate at 84 months for the control group was a 10.5% compared with a cumulative rate of 11.5% for the investigational group. There was no statistically significant difference between the groups.

Secondary Surgical Procedures

Secondary surgical procedures performed after the index operation occurred in both the investigational and the control treatment groups (Table 5). At 84 months of follow-up in the investigational disc arthroplasty group, 11 patients had secondary surgeries (cumulative rate 4.8%) performed at the initial treatment level; 29 patients had second surgeries (cumulative rate 13.7%) performed in the fusion control group at the index level ($p < 0.001$).

Revision Surgery

There were no (0%) revision surgeries, defined as any procedure that adjusted or modified the original implant configuration, in the investigational group compared with 5 revision surgeries in 5 control patients (2.1%), which resulted in a significant between-group difference ($p = 0.019$).

Supplemental Fixation

Procedures that provided additional stabilization at the index surgical site were considered supplemental fixation procedures. In the IDE trial protocol, use of an external bone growth stimulator was also considered as supplemental fixation. In the control group 5 patients (2.3%) underwent supplemental posterior spinal fixation surgeries compared with no investigational patients ($p = 0.017$), and 7 patients (3.0%) had application of an external bone graft stimulator to treat suspected symptomatic nonunion arising from the index fusion procedures, whereas no investigational patients underwent application of a bone growth stimulator ($p = 0.005$). The use of bone growth stimulators was not included in the comparison of surgical procedures in this paper, as bone growth stimulators were only used in the control group.

Implant Removal

Eight nonelective implant removals occurred in both treatment groups ($p = 0.808$). All patients underwent removal of the disc implant and interbody fusion because of persistent radicular pain. No disc arthroplasty implants were electively removed; in the control group, 13 patients (7.0%) underwent elective removal.

Reoperation

Reoperations occurred in both treatment groups at similar rates. The investigational group had a reoperation rate of 1.5% (4 patients) compared with 3.0% (4 patients) in the control group.

Second Surgery at Adjacent Levels

Secondary surgeries at adjacent cervical levels occurred as stand-alone procedures and also involved revision surgery at the initial index surgical level. Secondary surgery that involved only an adjacent level occurred in

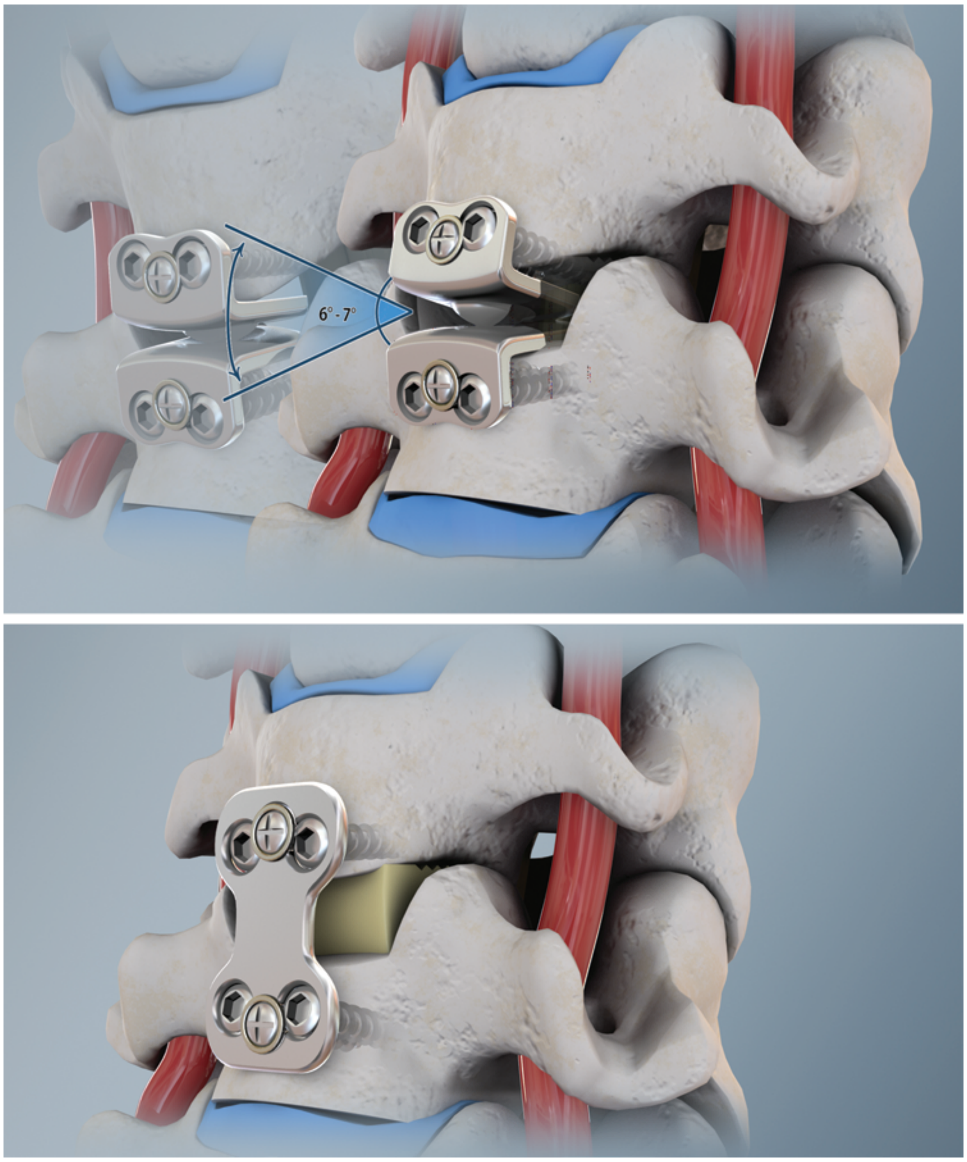

Fig. 5. **Upper:** Range of motion in flexion and extension of the cervical spine with the Prestige ST implant in place. The arthroplasty maintained a mean of 6° to 7° at 84 months. **Lower:** An anterior cervical fusion with allograft and plate fixation with no segmental motion.

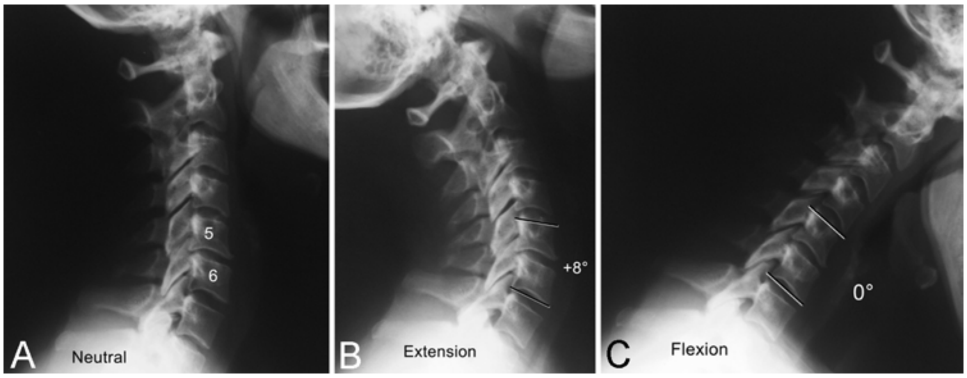

Fig. 6. **A:** Preoperative lateral radiograph showing disc space narrowing and radial osteophyte formation at the C5–6 level. **B:** Lateral extension radiograph shows 8° of lordosis across the C5–6 interspace. **C:** Lateral flexion radiograph showing the C5–6 interspace only flexes to 0°.

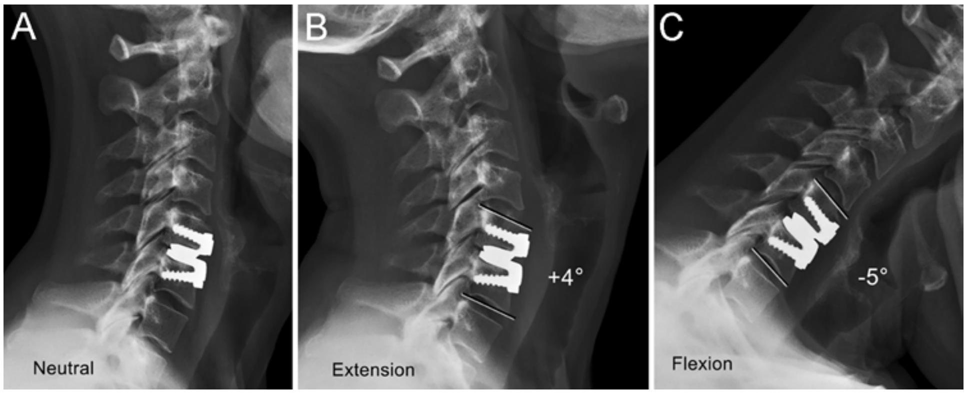

Fig. 7. **A:** Postoperative lateral radiograph obtained at 84 months, showing the disc prosthesis in place at the C5–6 level. **B:** Lateral extension radiograph showing 4° of lordosis at the C5–6 interspace. **C:** Lateral flexion radiograph showing an increase to 5° of kyphosis at the interspace.

8 patients (3.9%) in the investigational group and 10 patients (5.4%) in the control group (p = 0.451). The cumulative rates are calculated from the log-rank test.

Through 84 months, 11 investigational patients (4.6%) and 24 control patients (11.9%) underwent second surgeries that involved adjacent levels (p = 0.008) (Fig. 8).

Discussion

A large-scale, prospective, randomized, multicenter study in which the Prestige disc was compared with anterior cervical discectomy and fusion was initiated in 2002.[15] Investigators reported the interim data from this

TABLE 4: Summary and comparison of all adverse events based on life-table method

	24-Mo Follow-Up Window (911 days)		Cumulative Through Study Completion		
	Investigational (n = 276)	Control (n = 265)	Investigational (n = 276)	Control (n = 265)	
Adverse Event Category	No. of Patients (Cumulative Rate %)*	No. of Patients (Cumulative Rate %)*	No. of Patients (Cumulative Rate %)*	No. of Patients (Cumulative Rate %)*	p Value†
patients w/ any adverse events	235 (86.4)	219 (87.5)	259 (97.7)	232 (94.5)	0.958
anatomic technical difficulty	1 (0.4)	0 (0.0)	1 (0.4)	0 (0.0)	0.327
cancer	5 (1.9)	2 (0.8)	12 (5.2)	5 (2.4)	0.148
cardiovascular	18 (6.8)	13 (5.5)	39 (17.8)	31 (15.5)	0.601
carpal tunnel syndrome	18 (6.8)	7 (2.9)	24 (10.6)	12 (6.0)	0.076
death	0 (0.0)	3 (1.3)	2 (0.9)	5 (2.2)	0.197
dysphagia/dysphonia	24 (8.7)	22 (8.4)	29 (11.5)	26 (10.5)	0.868
gastrointestinal	30 (11.2)	30 (12.5)	56 (24.7)	51 (24.9)	0.915
implant displacement/loosening	2 (0.8)	5 (2.1)	6 (3.1)	7 (3.1)	0.623
infection	32 (11.9)	24 (10.0)	62 (27.0)	41 (20.5)	0.091
neck or arm pain	145 (53.8)	121 (48.8)	178 (70.2)	148 (64.8)	0.094
neurological	68 (25.2)	60 (24.6)	99 (42.5)	87 (41.5)	0.79
nonunion	0 (0.0)	9 (3.6)	0 (0.0)	9 (3.6)	0.002
nonunion (outcome pending)	0 (0.0)	21 (8.9)	0 (0.0)	22 (10.0)	<0.001
other	81 (30.2)	85 (35.0)	137 (57.2)	127 (57.1)	0.637
other pain	82 (31.1)	64 (26.3)	137 (58.7)	107 (50.1)	0.081
respiratory	10 (3.8)	8 (3.4)	22 (9.7)	19 (10.1)	0.897
spinal event	23 (8.6)	50 (20.6)	45 (20.9)	82 (38.9)	<0.001
subsidence	1 (0.4)	0 (0.0)	3 (2.3)	0 (0.0)	0.087
trauma	72 (27.2)	50 (20.8)	107 (44.9)	93 (44.9)	0.688
urogenital	20 (7.7)	8 (3.3)	44 (20.1)	23 (12.2)	0.024
intraop vascular injury	5 (1.8)	2 (0.8)	6 (2.2)	3 (1.3)	0.36

* Cumulative rates include adverse events from patients who withdrew from the study.
† The p values are from log-rank test for time-to-event analysis.

TABLE 5: Summary of secondary surgeries at the treated level based on the life-table method

	24-Mo Follow-Up Window (911 days)		Cumulative Through Study Completion		
	Investigational (n = 276)	Control (n = 265)	Investigational (n = 276)	Control (n = 265)	
Surgery*	No. of Patients (Cumulative Rate %)	No. of Patients (Cumulative Rate %)	No. of Patients (Cumulative Rate %)	No. of Patients (Cumulative Rate %)	p Value†
secondary surgery at treated level	9 (3.4)	19 (7.9)	11 (4.8)	29 (13.7)	<0.001
revision	0 (0.0)	4 (1.6)	0 (0.0)	5 (2.1)	0.019
removal	6 (2.3)	8 (3.3)	8 (3.6)	8 (3.3)	0.808
elective removal	0 (0.0)	4 (1.7)	0 (0.0)	13 (7.0)	<0.001
supplemental fixation	0 (0.0)	3 (1.3)	0 (0.0)	5 (2.3)	0.017
reoperation	4 (1.5)	2 (0.8)	4 (1.5)	4 (3.0)	0.894
supplemental fixation: BGS‡	0 (0.0)	6 (2.5)	0 (0.0)	7 (3.0)	0.006

* Some patients had more than 1 secondary procedure.
† The p value is for between-group comparisons through study completion from the log-rank test.
‡ BGS = external bone growth stimulator, which is not included in the total number of secondary surgeries at the treated level.

study as patients enrolled in the study began their 24-, 36-, and 60-month evaluations.[4] In this article, we report the final 60- and 84-month outcomes from this study.

Using the Prestige disc implant in patients with single-level cervical degenerative disc disease, sustained clinical and radiographic improvements in validated clinical outcome measurements were maintained at 84 months following surgery. A slightly earlier return to work was seen in the disc replacement group.[15,22] The Prestige disc maintained physiological segmental motion after implantation at the index surgical level; motion was also maintained at both adjacent-level disc spaces. Preservation of segmental motion may be related to a reduction in degenerative radiographic findings in adjacent segments in patients treated with arthroplasty.[9,10,17,24] In addition, there was only 1 implant migration through 84 months and low rates of bridging bone at the implant site.

Similar outcomes were reported with the Bryan disc in an RCT at 12- and 24-month follow-up.[1,8] The postoperative data from that study showed improvement in all clinical outcome measures by 12 months; at 24 months after surgery, the disc replacement group had a statistically greater improvement in the primary outcome variables, including NDI score and overall success, than the fusion group. These clinical and radiographic improvements were maintained out to 4 years.[21] Outcomes at 2 and 5 years have been reported for the ProDisc-C cervical disc replacement.[16,27] In an RCT evaluating the surgical treatment of single-level cervical disc disease, the investigators reported that in the ProDisc-C treatment group there was a statistically and clinically significant improvement at 2 and 5 years compared with baseline. At 5 years, ProDisc-C patients had statistically significantly less neck pain intensity and frequency compared with the fusion control group.

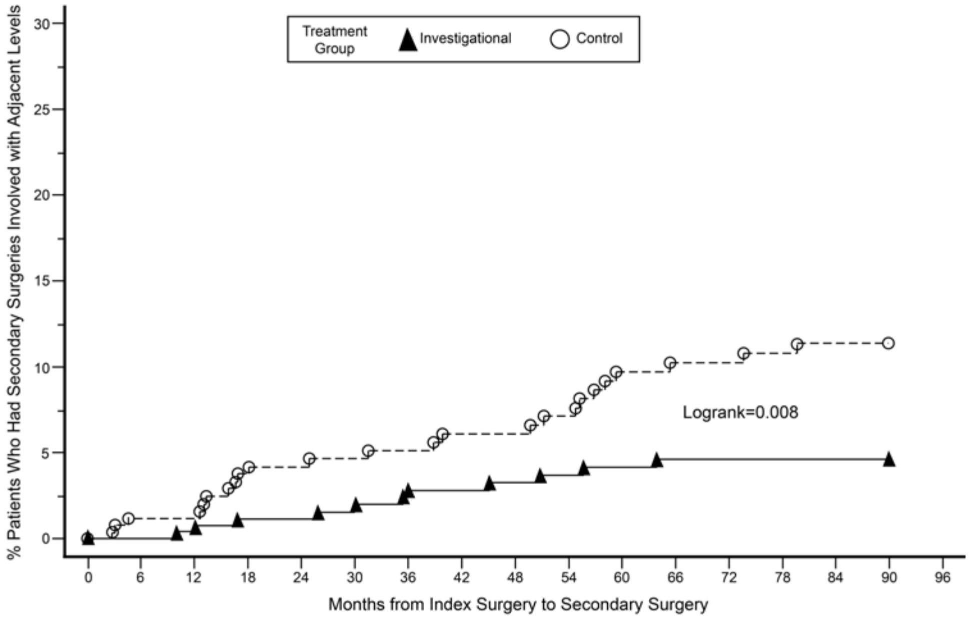

Fig. 8. Second surgeries that involved adjacent levels were those at adjacent levels only or involved both adjacent and index levels.

In a different RCT of a metal-on-metal cervical disc replacement (Kineflex|C) in patients with 24 months of follow-up, the investigators reported similar outcomes to the Prestige disc. The mean NDI and visual analog pain scores improved significantly by 6 weeks after surgery and remained significantly improved throughout the 24-month follow-up period.[5] The overall success rate was significantly greater in the disc replacement group when compared with the fusion group.

Dysphagia is a common occurrence after anterior cervical spinal procedures.[2] Surgical level, number of levels, instrumentation, and operative time have been associated with its occurrence. In addition, a correlation between intraoperative pharynx/esophagus retraction and postoperative swallowing disturbances has been established.[14] There were no differences in the incidence of dysphagia or dysphonia between the 2 groups at any of the time frames studied. The incidence of postoperative dysphagia and the long-term resolution of the dysphagia were similar in a previously reported RCT.[11]

The Prestige disc is able to maintain sagittal angular motion throughout 7 years postoperatively at the index surgical site. In addition, the superior and inferior adjacent levels to the index surgery showed similar motion preservation for preoperative measurements. Motion preservation after cervical disc replacement is impacted by spontaneous fusion and the development of heterotopic ossification. Differences in occurrence rate of heterotopic ossification are, in part, related to the prosthesis type.[13,26] The Prestige disc has the lowest rate of heterotopic ossification formation reported among contemporary RCTs for cervical disc replacements.

Conclusions

Cervical disc arthroplasty has the potential for preserving motion at the operated level while providing biomechanical stability and global neck mobility and may result in a reduction in adjacent-segment degeneration. The Prestige disc maintains improved clinical outcomes and segmental motion after implantation at 7 years of follow-up.

Acknowledgments

We acknowledge the following investigators: Perry J. Argires, M.D.; Hyun Bae, M.D.; David Benglis, M.D.; Paul A. Broadstone, M.D.; Wade M. Ceola, M.D.; Johnny Delashaw, M.D.; Kevin Foley, M.D.; Jose Garcia-Corrada, M.D.; Douglas Geiger, M.D.; Matthew F. Gornet, M.D.; Joseph Grant, M.D.; Jeremy Greenlee, M.D.; Jason Highsmith, M.D.; Theodore Jacobs, M.D.; Jason R. Hubbard, M.D.; Jorge E. Isaza, M.D.; Ross Jenkins, M.D.; Kee D. Kim, M.D.; Thomas Kopitnik, M.D.; Jeffrey Lewis, M.D.; R. Dean Martz, M.D.; Bruce Mathern, M.D.; Hallett Mathews, M.D.; Matthew T. Mayr, M.D.; Lloyd Mobley, M.D.; Robert Narotzky, M.D.; Russell P. Nockels, M.D.; Charles C. Park, M.D.; Joel Pickett, M.D.; Ralph F. Reeder, M.D.; Joseph Riina, M.D.; James Robinson, M.D.; Timothy Ryken, M.D.; Patrick Ryan, M.D.; Paul Sawin, M.D.; David D. Schwartz, M.D.; Joseph Sramek, M.D.; Rebecca E. Stachniak, M.D.; Ann Stroink, M.D.; Steven E. Swanson, M.D.; Brian Wieder, M.D.; Charles Winters, M.D.; and Thomas A. Zdeblick, M.D.

Disclosure

Medtronic biostatisticians, Guorong Ma, Ph.D.; Feng Tang, Ph.D.; and Youjun Zhu, M.S., provided data analysis and statistical review of the manuscript. David Wootten, Ph.D., also an employee of Medtronic, assisted with manuscript review and editing. Dr. Burkus has received reportable payments for consulting fees and royalty payments from Medtronic, Inc. He has no proprietary interest, such as a patent, trademark, or copyright ownership or a licensing relationship, with regard to the product under investigation. Since the commencement of the clinical study, Dr. Burkus has not had an equity interest (such as ownership, stock, or stock options) in Medtronic, Inc. Since the commencement of the clinical study, he has not received compensation in which the value of compensation could be affected by study outcome, such as a royalty interest. Dr. Traynelis is a consultant to and receives royalties and institutional research support from Medtronic. He receives institutional research support from the NIH and institutional fellowship support from Globus. Dr. Haid receives royalties from Medtronic and Globus and is a consultant for NuVasive and Piedmont Healthcare. He serves on the Medical Board of Directors for Globus and has stock in NuVasive, Globus, Spinewave, and Vertical Health. Dr. Mummaneni is not a consultant for and he has not received payments from Medtronic, the manufacturer of the Prestige implant. He has received honoraria from Globus and DePuy Spine and has received a royalty from DePuy Spine related to thoracolumbar instrumentation but not related to any cervical implant. He receives royalities from Quality Medical Publishing and Thieme Publishing.

Author contributions to the study and manuscript preparation include the following. Conception and design: all authors. Acquisition of data: all authors. Analysis and interpretation of data: all authors. Drafting the article: Burkus. Critically revising the article: all authors. Reviewed submitted version of manuscript: all authors. Approved the final version of the manuscript on behalf of all authors: Burkus.

References

1. Anderson PA, Sasso RC, Riew KD: Comparison of adverse events between the Bryan artificial cervical disc and anterior cervical arthrodesis. **Spine (Phila Pa 1976) 33:**1305–1312, 2008
2. Bazaz R, Lee MJ, Yoo JU: Incidence of dysphagia after anterior cervical spine surgery: a prospective study. **Spine (Phila Pa 1976) 27:**2453–2458, 2002
3. Beaurain J, Bernard P, Dufour T, Fuentes JM, Hovorka I, Huppert J, et al: Intermediate clinical and radiological results of cervical TDR (Mobi-C) with up to 2 years of follow-up. **Eur Spine J 18:**841–850, 2009
4. Burkus JK, Haid RW, Traynelis VC, Mummaneni PV: Long-term clinical and radiographic outcomes of cervical disc replacement with the Prestige disc: results from a prospective randomized controlled clinical trial. Clinical article. **J Neurosurg Spine 13:**308–318, 2010
5. Coric D, Nunley PD, Guyer RD, Musante D, Carmody CN, Gordon CR, et al: Prospective, randomized, multicenter study of cervical arthroplasty: 269 patients from the Kineflex|C artificial disc investigational device exemption study with a minimum 2-year follow-up. Clinical article. **J Neurosurg Spine 15:**348–358, 2011
6. Cummins BH, Robertson JT, Gill SS: Surgical experience with an implanted artificial cervical joint. **J Neurosurg 88:**943–948, 1998
7. Farrington CP, Manning G: Test statistics and sample size formulae for comparative binomial trials with null hypothesis of non-zero risk difference or non-unity relative risk. **Stat Med 9:**1447–1454, 1990
8. Heller JG, Sasso RC, Papadopoulos SM, Anderson PA, Fessler RG, Hacker RJ, et al: Comparison of BRYAN cervical disc arthroplasty with anterior cervical decompression and fusion: clinical and radiographic results of a randomized, controlled, clinical trial. **Spine (Phila Pa 1976) 34:**101–107, 2009

9. Kim SW, Limson MA, Kim SB, Arbatin JJ, Chang KY, Park MS, et al: Comparison of radiographic changes after ACDF versus Bryan disc arthroplasty in single and bi-level cases. **Eur Spine J 18:**218–231, 2009
10. Kulkarni V, Rajshekhar V, Raghuram L: Accelerated spondylotic changes adjacent to the fused segment following central cervical corpectomy: magnetic resonance imaging study evidence. **J Neurosurg 100 (1 Suppl Spine):**2–6, 2004
11. McAfee PC, Cappuccino A, Cunningham BW, Devine JG, Phillips FM, Regan JJ, et al: Lower incidence of dysphagia with cervical arthroplasty compared with ACDF in a prospective randomized clinical trial. **J Spinal Disord Tech 23:**1–8, 2010
12. McAfee PC, Reah C, Gilder K, Eisermann L, Cunningham B: A meta-analysis of comparative outcomes following cervical arthroplasty or anterior cervical fusion: results from 4 prospective multicenter randomized clinical trials and up to 1226 patients. **Spine (Phila Pa 1976) 37:**943–952, 2012
13. Mehren C, Suchomel P, Grochulla F, Barsa P, Sourkova P, Hradil J, et al: Heterotopic ossification in total cervical artificial disc replacement. **Spine (Phila Pa 1976) 31:**2802–2806, 2006
14. Mendoza-Lattes S, Clifford K, Bartelt R, Stewart J, Clark CR, Boezaart AP: Dysphagia following anterior cervical arthrodesis is associated with continuous, strong retraction of the esophagus. **J Bone Joint Surg Am 90:**256–263, 2008
15. Mummaneni PV, Burkus JK, Haid RW, Traynelis VC, Zdeblick TA: Clinical and radiographic analysis of cervical disc arthroplasty compared with allograft fusion: a randomized controlled clinical trial. **J Neurosurg Spine 6:**198–209, 2007
16. Murrey D, Janssen M, Delamarter R, Goldstein J, Zigler J, Tay B, et al: Results of the prospective, randomized, controlled multicenter Food and Drug Administration investigational device exemption study of the ProDisc-C total disc replacement versus anterior discectomy and fusion for the treatment of 1-level symptomatic cervical disc disease. **Spine J 9:**275–286, 2009
17. Park DK, Lin EL, Phillips FM: Index and adjacent level kinematics after cervical disc replacement and anterior fusion: in vivo quantitative radiographic analysis. **Spine (Phila Pa 1976) 36:**721–730, 2011
18. Park JH, Rhim SC, Roh SW: Mid-term follow-up of clinical and radiologic outcomes in cervical total disk replacement (Mobi-C): incidence of heterotopic ossification and risk factors. **J Spinal Disord Tech 26:**141–145, 2013
19. Porchet F, Metcalf NH: Clinical outcomes with the Prestige II cervical disc: preliminary results from a prospective randomized clinical trial. **Neurosurg Focus 17(3):**E6, 2004
20. Robertson JT, Metcalf NH: Long-term outcome after implantation of the Prestige I disc in an end-stage indication: 4-year results from a pilot study. **Neurosurg Focus 17(3):**E10, 2004
21. Sasso RC, Anderson PA, Riew KD, Heller JG: Results of cervical arthroplasty compared with anterior discectomy and fusion: four-year clinical outcomes in a prospective, randomized controlled trial. **J Bone Joint Surg Am 93:**1684–1692, 2011
22. Steinmetz MP, Patel R, Traynelis V, Resnick DK, Anderson PA: Cervical disc arthroplasty compared with fusion in a workers' compensation population. **Neurosurgery 63:**741–747, 2008
23. Upadhyaya CD, Wu JC, Trost G, Haid RW, Traynelis VC, Tay B, et al: Analysis of the three United States Food and Drug Administration investigational device exemption cervical arthroplasty trials. Clinical article. **J Neurosurg Spine 16:**216–228, 2012
24. Wigfield C, Gill S, Nelson R, Langdon I, Metcalf N, Robertson J: Influence of an artificial cervical joint compared with fusion on adjacent-level motion in the treatment of degenerative cervical disc disease. **J Neurosurg 96 (1 Suppl):**17–21, 2002
25. Wigfield CC, Gill SS, Nelson RJ, Metcalf NH, Robertson JT: The new Frenchay artificial cervical joint: results from a two-year pilot study. **Spine (Phila Pa 1976) 27:**2446–2452, 2002
26. Yi S, Kim KN, Yang MS, Yang JW, Kim H, Ha Y, et al: Difference in occurrence of heterotopic ossification according to prosthesis type in the cervical artificial disc replacement. **Spine (Phila Pa 1976) 35:**1556–1561, 2010
27. Zigler JE, Delamarter R, Murrey D, Spivak J, Janssen M: Pro-Disc-C and anterior cervical discectomy and fusion as surgical treatment for single-level cervical symptomatic degenerative disc disease: five-year results of a Food and Drug Administration study. **Spine (Phila Pa 1976) 38:**203–209, 2013

Manuscript submitted November 5, 2013.

Accepted June 12, 2014.

Please include this information when citing this paper: published online July 18, 2014; DOI: 10.3171/2014.6.SPINE13996.

Address correspondence to: J. Kenneth Burkus, M.D., The Hughston Clinic, 6262 Veterans Parkway, Columbus, GA 31908. email: jkb66@knology.net.
