# Supplementary material for: Adjacent segment degeneration or disease after cervical total disc replacement: a meta-analysis of randomized controlled trials
Source: J Orthop Surg Res. 2018 Oct 3;13:244. doi: 10.1186/s13018-018-0940-9 (PMC6169069; doi:10.1186/s13018-018-0940-9)
Supplement: Supplementary file 2 — File S1. Original data of 11 included articles. (ZIP 12 mb) [file 13018_2018_940_MOESM2_ESM.zip › 11 included articles and original data referred in this article/11 included articles/29 Zhang, H Xú¿CHAú⌐.pdf]

International Orthopaedics (SICOT) (2014) 38:2533–2541
DOI 10.1007/s00264-014-2497-5

ORIGINAL PAPER

A prospective, randomised, controlled multicentre study comparing cervical disc replacement with anterior cervical decompression and fusion

Hao-Xuan Zhang · Yuan-Dong Shao · Yu Chen · Yong Hou · Lei Cheng · Meng Si · Lin Nie

Received: 22 July 2014 / Accepted: 4 August 2014 / Published online: 11 September 2014
© SICOT aisbl 2014

Abstract

Purpose Total cervical artificial disc replacement (TDR) simulates normal disc structure, thus avoiding the drawbacks of anterior cervical decompression and fusion (ACDF). This prospective, randomized, controlled and multicentre study aimed to evaluate clinical and radiographic outcomes by comparing cervical disc replacement using Mobi-C disc prostheses with ACDF.

Methods This prospective, randomized, controlled and multicentre study consisted of 111 patients undergoing single-level Mobi-C disc prosthesis replacement (TDR group, n=55) or ACDF (n=56) from February 2008 to November 2009 at 11 medical centres across China. Patients were assessed before surgery, at seven days postoperation and one, three, six, 12, 24, 36 and 48 months postoperation. Clinical and neurological outcome was determined by measuring the Japanese Orthopaedic Association (JOA) scores, visual analogue scale (VAS) and Neck Disability Index (NDI). Static and dynamic radiographs were obtained of the cervical curvature, the functional spinal unit (FSU) angle and range of motion (ROM) of the cervical spine, FSU angle and treated and adjacent segments.

Results A total of 111 patients were included and randomly assigned to either Mobi-C disc prosthesis replacement or ACDF. JOA, VAS and NDI showed statistically significant improvements 48 months after surgery (P<0.05). ROM, FSU angle, treated segment and adjacent segments in the Mobi-C group were not significantly different before and after replacement (p>0.05). ROM in the ACDF group was significantly reduced at one month and remained so throughout the follow-up. By 48-months, more ACDF patients required secondary surgery (four of 56 patients).

Conclusions Although ACDF may increase the risk of additional surgery, clinical outcomes indicated that both Mobi-C artificial cervical disc replacement and ACDF were reliable. Radiographic data showed that ROM of the cervical spine, FSU angle and treated and adjacent segments were relatively better reconstructed and maintained in the Mobi-C group compared with those in the ACDF group.

Keywords Artificial cervical disc replacement · Mobi-C disc · Cervical degenerative disc disease

Abbreviations

ACDF	Anterior cervical decompression and fusion
JOA	Japanese Orthopaedic Association Scores
VAS	Visual analogue scale/score
NDI	Neck Disability Index
ROM	Range of motion
FSU	Functional spinal unit
TDR	Total cervical artificial disc replacement
CT	Computed tomography
MRI	Magnetic resonance imaging
HO	Heterotopic ossification

H.-X. Zhang · Y. Hou · L. Cheng · M. Si · L. Nie (✉)
Department of Orthopedics, Shandong University Qilu Hospital, No.107, Wen Hua Xi Road, Jinan, Shandong 250012, People's Republic of China
e-mail: hoho05@126.com

Y.-D. Shao
Department of Orthopedic Surgery, Binzhou People' s Hospital, Binzhou, Shandong, China

Y. Chen
School of Public Health, Shanghai Jiaotong University, Shanghai, China

Introduction

Anterior cervical discectomy and fusion (ACDF) is one of the most widely used treatments for cervical degenerative disc disease. Although numerous studies have shown that ACDF can achieve good therapeutic effect [1–3], increasing evidence suggests that ACDF may also accelerate adjacent segment degeneration and even lead to a secondary surgery [4–6]. Recently, total cervical disc replacement (TDR) has been developed to promote the formation of normal disc structure and rebuild or reserve the cervical motor function and biomechanical environment, thus reducing adjacent-segment degeneration and avoiding fusion-related complications. Currently, TDR is widely applied in the field of spine surgery, and its short-term efficacy has been recognised [7–9]. However, only a few studies have assessed its long-term efficacy in patients with symptomatic cervical spondylosis. In this study, a total of 111 cervical spondylosis patients who were treated by either single-level TDR using Mobi-C prosthesis or ACDF at 11 medical centres in China were assessed. We comprehensively determined the long-term (minimum of 48-month follow-up) clinical efficacy of TDR and ACDF and explored the role Mobi-C prostheses in rebuilding or reserving cervical curvature, functional spinal unit (FSU) angle and range of motion (ROM) of the cervical spine and FSU of the treated and adjacent segments.

Materials and methods

Ethics statement

This study was approved by Medical Ethical Committee of Qilu Hospital, Shandong University, China. Written informed consent was obtained from each participant before enrollment, and their identity was kept confidential.

Inclusion and exclusion criteria

Patients with single-level disease were included in the study. Patients 18–68 years of age and were included if they had a diagnosis of degenerative cervical spondylosis of one segmental level that was supported by clinical symptoms and imaging data and with no significant improvement after conservative treatment for at least three months before surgery. Patients were excluded if they had multisegmental-level cervical diseases, severe facet-joint degeneration, osteoporosis, cervical instability, spinal-canal stenosis, ossification of the posterior longitudinal ligament, tumor, infection or metal allergies.

Clinical data

From February 2008 to November 2009, 111 patients with single-level symptomatic cervical spondylosis were included from 11 separate medical institutions across China. Fifty-five patients were treated with Mobi-C disc replacement and 56 patients with ACDF using plate/cage. All patients received at least three months of prior strict conservative treatment with unsatisfactory efficacy. Neurogenic disease was ruled out by a neurologist. Before surgery, all patients underwent static and dynamic radiographs, cervical-spine computed tomography (CT) and magnetic resonance imaging (MRI). All patients were evaluated preoperatively, at seven days and at one, three, six, 12, 24, 36 and 48 months postoperatively. Clinical data were collected for a minimum of 48 months.

Clinical evaluation method

Japanese Orthopaedic Association (JOA) [10], visual analogue scale (VAS)[11] and Neck Disability Index (NDI) [12] were used to determining clinical outcomes and patient improvement.

Radiographic evaluation method

Cervical curvature was determined by measuring Cobb's angle between the inferior end plate of C2 and C7 vertebral bodies in the lateral cervical spine X-ray. ROM of the cervical spine was defined as the difference in Cobb angle in radiographs at full flexion and extension. FSU angle was used to determine the mobility of the treated segments and was assessed using the lines at the superior end plate of the superior vertebral body and those at the inferior end plate of the inferior body on cervical spine radiographs with the patient in a neutral position. FSU ROM was defined as the difference in FSU angle between full flexion and extension in lateral radiographs. The treated segment angle was measured by drawing lines at the inferior end plate of the superior vertebral body and at the superior end plate of the inferior body. A postoperative radiograph was taken for the shell of the Mobi-C prosthesis. ROM of the treated segment was defined as the difference in treated segment angle between full flexion and extension in lateral radiographs. ROM of the adjacent segment was calculated similarly. All radiologic evaluations were performed using SpineView® software (Surgiview, France) [13].

Statistical analysis

All data are expressed as mean ± standard deviation (SD), and statistical analysis was determined using a single-factor analysis of variance (ANOVA) with Bonferroni's post hoc tests for multiple comparisons of baseline within the treatment groups

at each follow-up timepoint . For between-treatment group comparisons, paired *t* tests were performed. All statistical analyses were performed with SPSS software (version 16.0, IL, Chicago, IL, USA); differences of $p<0.05$ were considered significant.

Results

Patient population

Overall demographics were similar between the TDR and ACDF groups. The TDR group consisted of 25 men and 30 women, and the ACDF group consisted of 26 men and 30 women. The average age was 44.8 years in the TDR group and 46.7 years (18–68) in the ACDF group. Body mass index (BMI) was 25.3 kg/m^2 in the TDR group and 26.5 kg/m^2 in the ACDF group. There were ten C3/4, 17 C4/5, 16 C5/6 and 12 C6/7 replacements in the TDR group, and ten C3/4, 18 C4/5, 16 C5/6 and 12 C6/7 replacements in the ACDF group. The average follow-up was 50.5 months in the TDR group and 52.1 months in the ACDF group. Estimated blood loss in the ACDF group was statistically higher than that in the TDR group. There were no blood transfusions in either group. Overall patient demographics were similar between groups.

Operative treatment

All 111 patients underwent either anterior discectomy and implantation of the Mobi-C disc prosthesis (55 patients) or fusion (56 patients) with autogenous iliac or clavicle bone graft. There was no difference between groups in operative time (Mobi-C, 84.5 minutes; ACDF, 92.2 minutes). Since Estimated blood loss from autogenous iliac and clavicle bone grafts were statistically higher for ACDF compared with Mobi-C patients were 69.1 ml and 40.4 ml, respectively.There were no blood transfusions in either group.

Postoperative general condition and complications

All patients in the Mobi-C group were transferred to ambulatory 48–72 hours of surgery without using a collar or other neck encumbering braces. However, patients in the ACDF group were ambulatory by 48 to 72 hours after surgery with neck-encumbering braces. A total of 28 (15 Mobi-C group and 13 ACDF) patients had pharyngeal discomfort or hoarseness ranging in severity, which may be due to the surgical approach, which may involve excessive stretch, stimulation of the esophagus, trachea, larynx or recurrent laryngeal nerve injury. All patients recovered in two weeks without special treatment.

In the Mobi-C group, the incidence of heterotopic ossification (HO) after replacement generally arose within the first year (18 of 55 patients) but it did not appear to increase after 12 months. Two cases at the 12-month follow-up and one case at 24 months showed forward movement of the prosthesis by about 2, 3 and 3 mm, respectively, which may be due to overactivity; however, none of them show any adverse symptoms. No patients expressed exacerbated symptoms, recompression of the spinal cord or nerve root, obvious cervical prosthesis subsidence or excursion and spontaneous fusion of the treated segment. In the ACDF group, pseudarthrosis rates in treated segments were 10.7 % (six of 56) at the 6-month follow-up and 1.8 % (one of 56) at a 48-month follow-up.There was a total of four adjacent-segment reoperations (7.1 %) in the ACDF group. Re-operation data is shown in Table 1. Adjacent-segment re-operations comprised one Mobi-C disc replacement and one ACDF and two posterior cervical open-door laminoplasties. Wound infections of iliac the bone-graft donor site were seen in two patients (3.6 %) and were all treated with a short course (three to five days) of antibiotics.

Clinical outcomes

JOA scores

In the TDR group, the average JOA score was 10.86 before treatment and increased to 12.80, 13.86 and 14.58, respectively, after seven days, one month and three months postoperatively. Pre-operative JOA score in the TDR group were significantly different from those seven days and 12, 24, 36 and 48 months postoperatively (Fig. 1), which was not significantly different at each time point after three months postoperatively ($p>0.05$). In the ACDF group, the average JOA score before surgery was 10.84 and was increased to 12.54, 13.64 and 14.44, respectively, after seven days and one and three months postoperatively, which was not significantly different at each time point after three months postoperatively ($p>0.05$). The JOA score was not significantly different between groups ($p>0.05$).

VAS scores

In the TDR group, the average VAS score before surgery was 6.72 and decreased to 4.20 and 2.04 at seven days and three months, respectively, postoperation. After three months, VAS score was not significantly changed (except at 48 months postoperation). In the ACDF group, the average VAS score before surgery was 6.64 and decreased to 4.44 and 2.24 at seven days and three months, respectively, postoperation. After three months, it was not significantly changed ($p>0.05$) (Fig. 2). Postoperative scores in both groups were significantly improved in comparison with preoperative scores.

Table 1 Reoperation data

	Sex	Implant Level	Reimplant Level	Interval Between Primary And Secondary Treatment	Reoperations
Case 1	Male	C4-5	C3-4	17 months	Mobi-C Disc Replacement
Case 2	Male	C6-7	C5-6	39 months	Posterior Open-door Laminoplasties
Case 3	Female	C3-4	C4-5	46 months	ACDF
Case 4	Male	C4-5	C3-4/C5-6	47 months	Posterior Open-door Laminoplasties

ACDF anterior cervical decompression and fusion

NDI scores

In the TDR group, the average NDI score before surgery was 37.44, which was decreased to 32.76, 19.00, 19.04, 19.56 and 19.60 at seven days, 12-months, 24-months, 36-months and 48-months postoperation, respectively. The mean postoperative NDI scores at each time point were significantly decreased in comparison to the pre-operative score. There were no significant improvements in mean NDI scores from six months to 48-months postoperation. In the ACDF group, the average NDI score before surgery was 37.76, which was decreased to 33.54, 19.22, 19.26, 19.86 and 20.10 at seven days, 12-months, 24-months, 36-months and 48-months postoperation, respectively. The mean postoperative NDI scores at each time point were significantly decreased in comparison to the pre-operative score. There were no significant improvements in the mean NDI scores from six months to 48-months postoperation. The NDI score was not significantly different between TDR and ACDF groups (Fig. 3).

Radiographic outcomes

Cervical curvature and ROM

In both ACDF and TDR groups, cervical curvature (Fig. 4a) and ROM (Fig. 4b) at seven days and one month postoperation were significantly lower than before operation but significantly greater than at the three months postoperation. From the 12-month to the final follow-up, cervical curvature was not significantly changed in either group ($p > 0.05$). From three

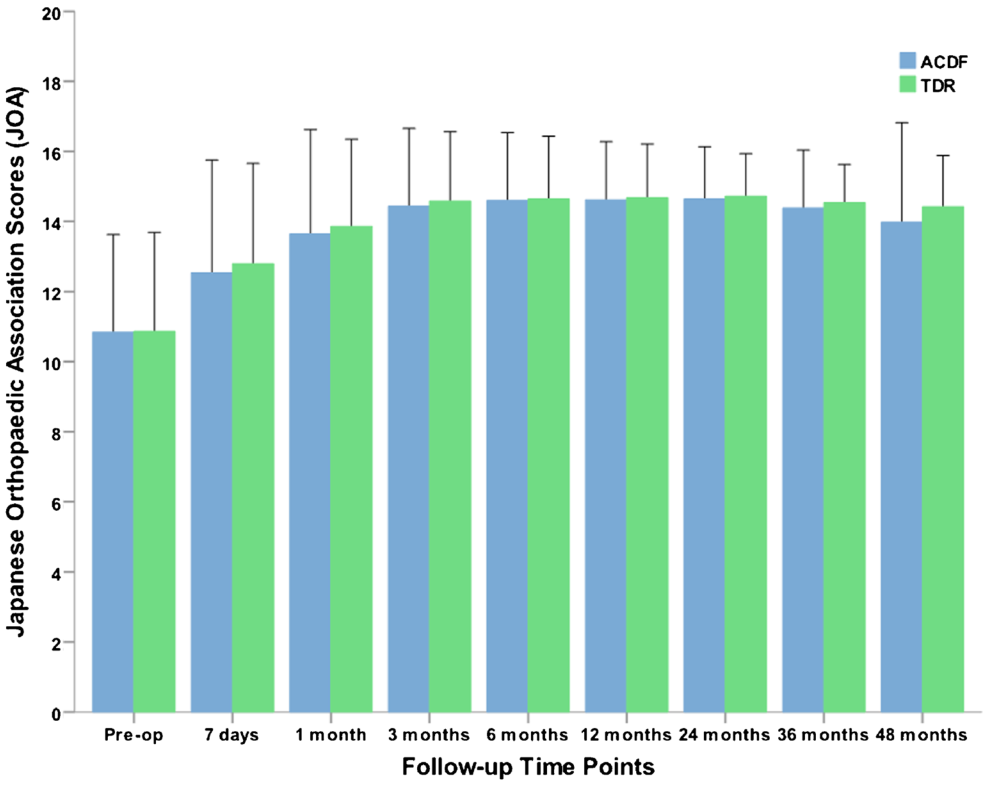

Fig. 1 Japanese Orthopaedic Association (JOA) scores for the Mobi-C total cervical artificial disc replacement (TDR) (55 patients) and anterior cervical decompression and fusion (ACDF) (56 patients) groups over time. Mean JOA score before and after surgery (seven days and one, three, six, 12, 24, 36 and 48 months) of 111 patients. Mean (±2 standard deviations) for each time point

Fig. 2 Visual analogue scale (VAS) scores for the Mobi-C total cervical artificial disc replacement (TDR) group (55 patients) and the anterior cervical decompression and fusion (ACDF) group (56 patients) over time. Mean before and after surgery (seven days and one, three, six, 12, 24, 36 and 48 months) of 111 patients. Mean (±2 standard deviations) for each time point

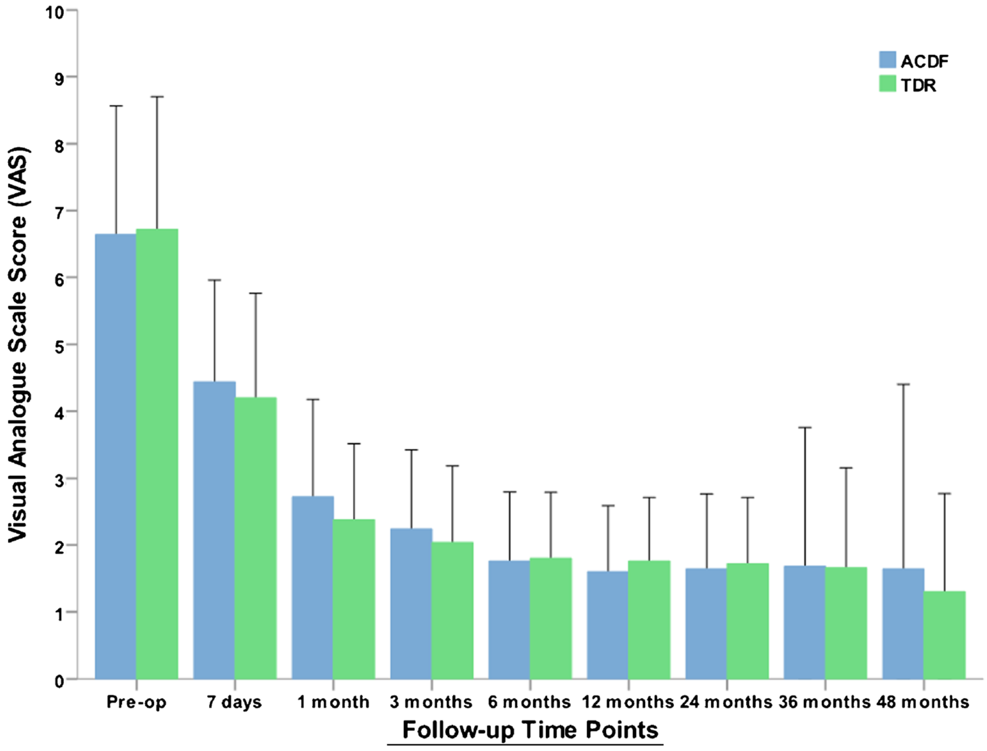

months postoperation to the end of the follow-up period, cervical spine ROM was not significantly changed in either group (p>0.05). Cervical curvature was not significantly different between groups (p>0.05). However, tcervical spine ROM in the TDR group was significantly greater than in the ACDF group from one to 48 months postoperation.

Fig. 3 Neck Disability Index (NDI) scores for the Mobi-C total cervical artificial disc replacement (TDR) group (55 patients) and the anterior cervical decompression and fusion (ACDF) group (56 patients) over time. Mean NDI score before and after surgery (seven days and one, three, six, 12, 24, 36 and 48 months) of 111 patients. Mean (±2 standard deviations) for each time point

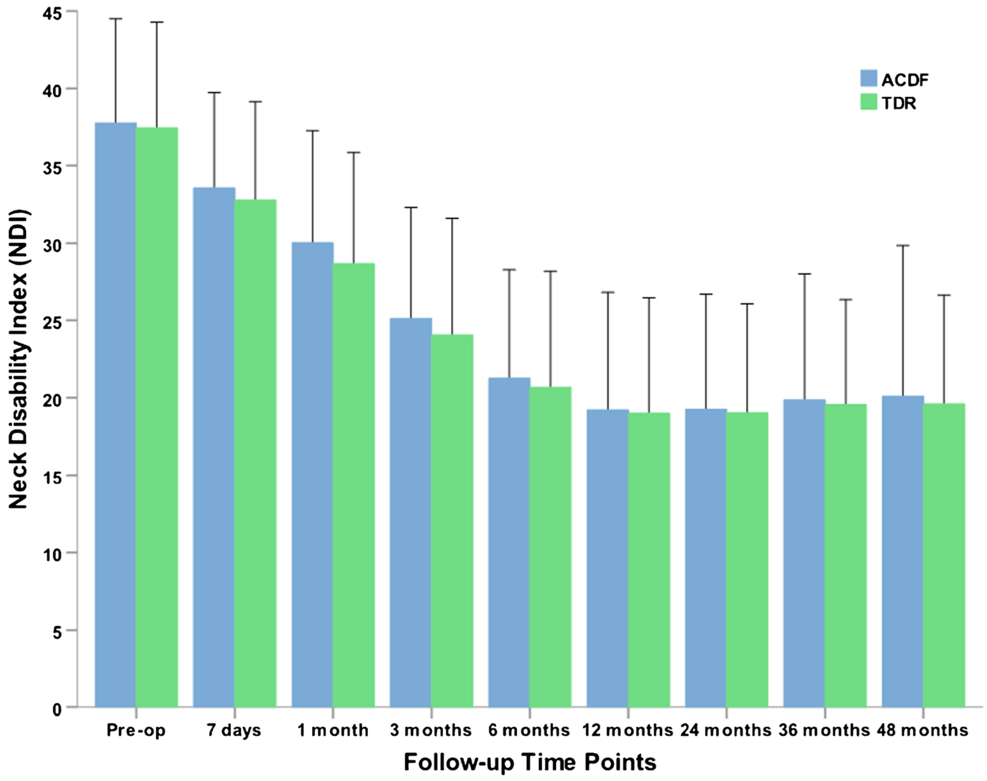

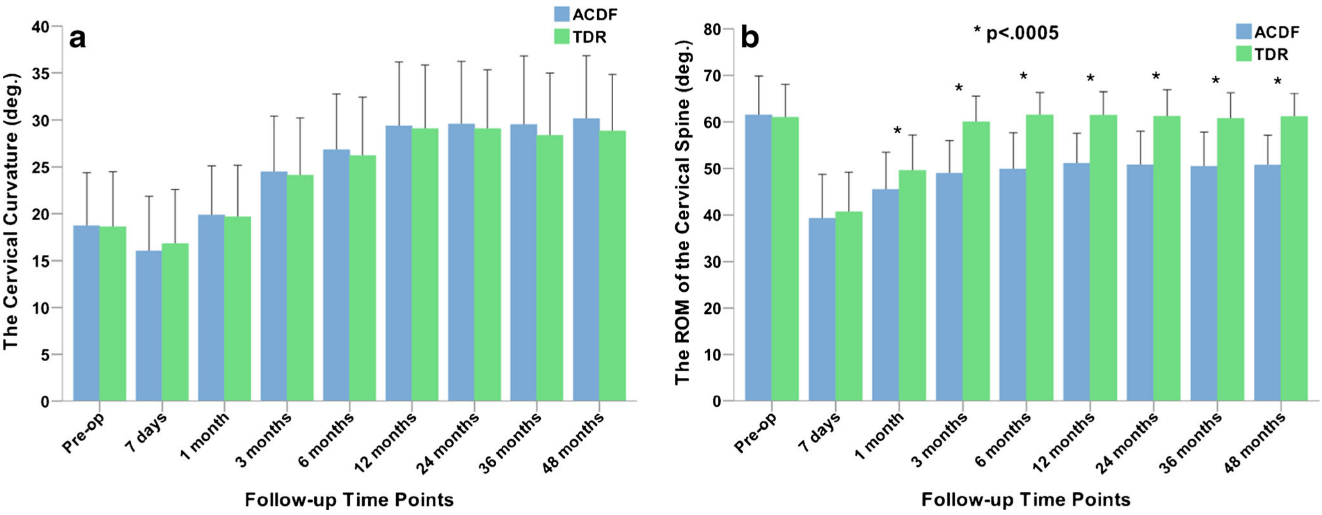

Fig. 4 Cervical curvature and range of motion (ROM) of the cervical spine for the Mobi-C total cervical artificial disc replacement (TDR) (55 patients) and anterior cervical decompression and fusion (ACDF) (56 patients) groups over time. Cervical curvature(**a**) and cervical-spine ROM (**b**) before and after surgery (seven days and one, three, six, 12, 24, 36 and 48 months). Mean (±2 standard deviation) for each time point

FSU angle and ROM

FSU angle (Fig. 5a) decreased significantly after surgery in both groups. However, it was restored to the preoperative level by six months postoperatively in the TDR group. FSU ROM (Fig. 5b) decreased after surgery in both groups. However, it was restored to preoperative levels three months postoperation in the TDR group, and FSU angle did not change significantly from three to 48 months postoperation ($p>0.05$). In the ACDF group, FSU angle did not change significantly from seven days postoperation to the end of follow-up ($p>0.05$). FSU angle and ROM in the TDR group was significantly greater than in the ACDF group from seven days to 48 months postoperation ($p<0.05$).

ROM of treated and adjacent segments

ROM of treated (Fig.6a) and adjacent (Fig.6b, c) segments decreased significantly after surgery in both groups. However, returned to preoperative levels by three months postoperation in the TDR group and significantly lower than that in the ACDF group from six to 48 months postoperation.

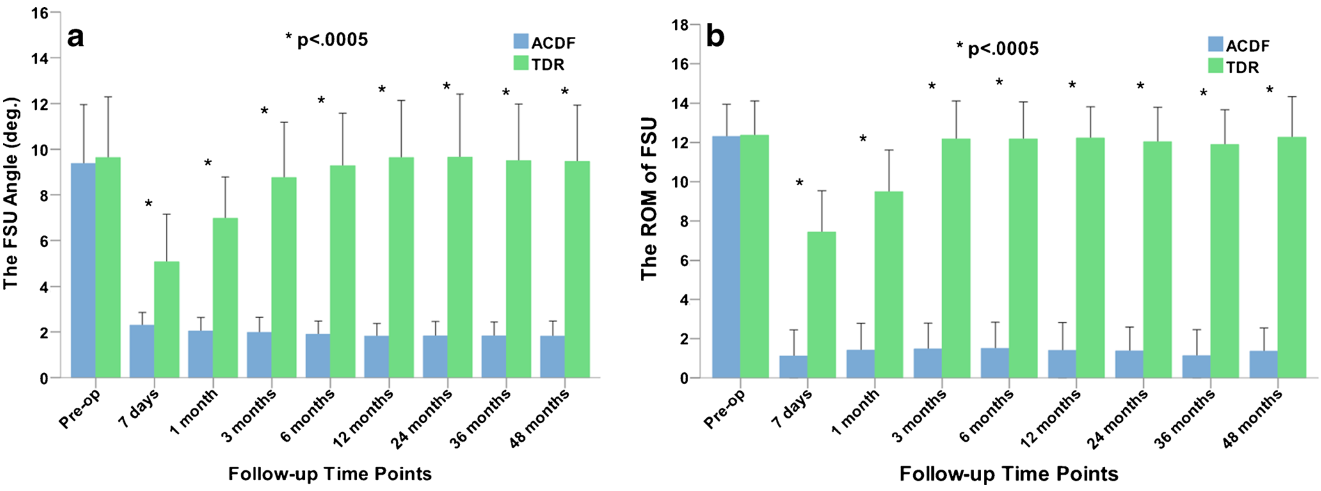

Fig. 5 Functional spinal unit (FSU) angle and range of motion (ROM) for the Mobi-C total cervical artificial disc replacement (TDR) (55 patients) and the anterior cervical decompression and fusion (ACDF) (56 patients) groups over time. FSU angle (**a**) and ROM (**b**) before and after surgery (seven days and one, three, six, 12, 24, 36 and 48 months). Mean (±2 standard deviations) for each time point

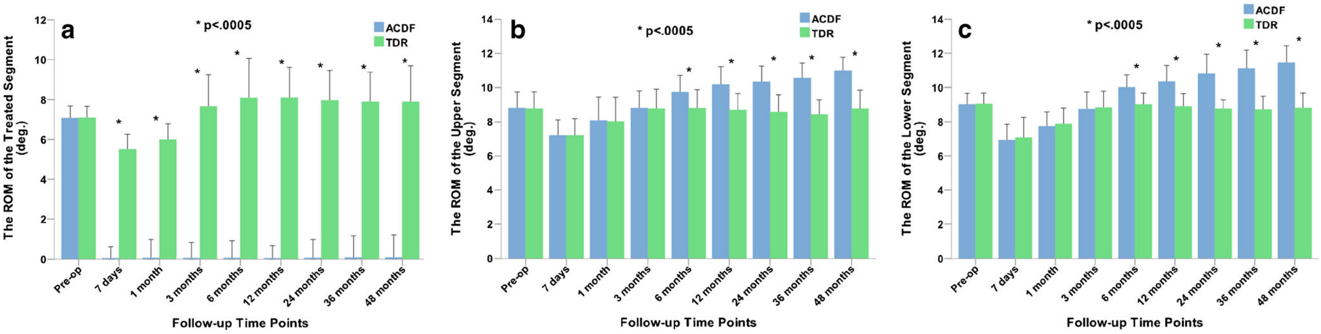

Fig. 6 Range of motion (ROM) of treated and adjacent segments for the Mobi-C total cervical artificial disc replacement (TDR) (55 patients) and anterior cervical decompression and fusion (ACDF) (56 patients) groups over time. ROM of treated (**a**), upper (**b**) and lower (**c**) segments before and after surgery (seven days and one, three, six, 12, 24, 36 and 48 months). Mean (±2 standard deviations) for each time point

Discussion

ACDF has been widely used and proven to clinically provide stability for cervical degenerative disc disease, especially cervical radiculopathy and spondylotic myelopathy. However, ACDF may change rotational movement of the cervical spine axis and increase load of adjacent segments, thus accelerating the degeneration of adjacent segments [14, 15]. Meanwhile, graft subsidence and loosening, bone nonunion and chronic pain at the graft donor site are also common problems associated with ACDF. Wada et al. [16] found that about 25 % of patients experienced pseudarthrosis, and vertebral ROM decreased from 39.4 to 19.2 (49 %) after ACDF. Veeravagu et al. [17] reviewed 28,777 cases undergoing ACDF and found that 9.13 % in the single-level and 10.7 % for multilevel ACDF groups required a second operation within two years. The number of fusion levels is significantly correlated with the rate of re-operations. Silber et al. [18] followed up on 134 patients undergoing ACDF and concluded that 11.2 % of them used medication for donor-site pain and 15.7 % experienced abnormal sensations at the site. Furthermore, impaired ambulation and recreational, work-, daily living, sexual and household activities occurred in 12.7 %, 11.9 %, 9.7 %, 8.2 %, 7.5 % and 6.7 % of patients, respectively.

The emergence and development of artificial cervical disc replacement provides an alternative surgical treatment for anterior cervical disease; at present, Mobi-C disc is the most widely used protheses. After complete removal of intervertebral disc tissue and the rear osteophyte, either anterior fusion or Mobi-C disc replacement allows the nerve root canal to obtain sufficient decompression, which ensures the therapeutic effect. Compared with ACDF, cervical artificial disc replacement can maintain normal cervical motor function of the treated and adjacent segments after decompression, thus avoiding degeneration of adjacent segments after surgery. Colle et al. [19] found that normal biomechanics could be restored after TDR, whereas rigid fixation could cause significant changes. Davis et al. [20] reviewed 225 cases undergoing TDR with Mobi-C disc prostheses and 105 cases undergoing ACDF and found that the TDR group maintained ROM at the treated segments and expressed greater improvement than the ACDF group.

In our study, both Mobi-C disc replacement and ACDF indicated good clinical outcomes. At 48 months postoperation, JOA, VAS and NDI scores were significantly improved, with no significant difference between groups from seven days to 48 months postoperation. Early clinical improvement is achieved by complete surgical decompression, removal of pathologically herniated discs and recovery of pathological spinal cord and nerve roots. The difference between groups was that the Mobi-C prostheses rebuilt or reserved cervical spine intervertebral height, ROM and FSU of treated and adjacent segments instantly, therefore avoiding acceleration of adjacent segment degeneration. Preoperative ROM was significantly different from postoperative ROM, which might be related to psychological stress, neck discomfort, postoperative neck pain and other factors. Radiological data showed that parameters related to kinetics, such as ROM, FSU and treated and adjacent segments were relatively better maintained in the TDR than in the ACDF group. In the TDR group, ROM was recovered to pre-operative level at the final follow-up.

There appears to be fewer patients undergoing re-operations of adjacent segments in the TDR group compared with in the ACDF group. We speculate that this might be due to the higher variations in postoperative measurements in the ACDF group. Although the clinical effect of cervical artificial disc replacement is encouraging, there are potential complications, such as HO prosthesis loosening, subsidence and displacement and spontaneous fusion. Jin et al. [21] reported a 62.1 % of type 1 HO, 13.7 % of type 2 and 4.2 % of type 3 associated with TDR according to the McAfee classification system. However, the clinical outcome was not significantly correlated with the occurrence of HO. In our study, 11 cases of type 1 HO, five of type 2 and two of type 3 occurred in the TDR group. However, occurrence of HO did not affect ROM

of the treated segment or cause spinal cord or nerve root recompression. Occurrence of HO in TDR may be due to the following:

(1) muscle injuries during operative procedure and residual bone debris [22–24]
(2) male gender and older age [25]
(3) uncommon use of nonsteroidal anti-inflammatory drugs (NSAIDS) during the peri-operative period [26, 27]
(4) different types of prosthesis
(5) excessive correction of FSU height and increase in ROM of the treated segment [28]
(6) posterior longitudinal ligament retention
(7) appropriate prosthesis size and learning curve [29].

In our study, three prostheses displayed 2–3 mm of forward displacement, but no patient reported obvious discomfort. Moderate functional exercise and appropriate implant size and position is advised to maintain prosthesis stability and avoid loosening, subsidence and displacement. Spontaneous fusion after TDR was not found in our study. Future studies will be designed to focus on the long-term effect of TDR and the mechanisms of lateral bending and axial rotation associated with TDR.

Conclusions

In conclusion, after 48 months of follow-up, data reported in this prospective, randomised, controlled and multicentre study indicate that clinical status improved in both TDR and ACDF groups. Radiographic parameters, such as cervical curvature and activity, were relatively better maintained in the TDR group compared with the ACDF group. Patients treated with TDR demonstrated statistically superior radiographic outcomes compared with those treated with ACDF. The maintenance of motion and less radiographic change might have resulted in the fewer adjacent-segment reoperations in our study.

Acknowledgments We are grateful to Jing-Kun Li and Shuai-Shuai Wang of the Department of Orthopedics, Shandong University Qilu Hospital, Jinan, Shandong, China, for assistance with data collection.

References

1. Cauthen JC, Kinard RE, Vogler JB et al (1998) Outcome analysis of noninstrumented anterior cervical discectomy and interbody fusion in 348 patients. Spine 23(2):188–192
2. Dickerman RD, Reynolds AS, Morgan B (2008) Polyetheretherketone (PEEK) cage filled with bone morphogenic protein and demineralised bone matrix in anterior cervical discectomy and fusion. Int Orthop 32(5):717–717
3. Kim SW, Limson MA, Kim SB et al (2009) Comparison of radiographic changes after ACDF versus Bryan disc arthroplasty in single and bi-level cases. Eur Spine J 18(2):218–231
4. Hacker RJ, Cauthen JC, Gilbert TJ et al (2000) A prospective randomized multicenter clinical evaluation of an anterior cervical fusion cage. Spine 25(20):2646–2655
5. Liu T, Yang HL, Xu YZ et al (2011) ACDF with the PCB cage-plate system versus laminoplasty for multilevel cervical spondylotic myelopathy. J Spinal Disord Tech 24(4):213–220
6. Davis RJ, Hoffman GA, Bae HW et al (2013) Cervical disc arthroplasty results in fewer secondary surgeries through 48 months compared to ACDF: results for a prospective randomized IDE study for Two-level Use. Spine J 13(9):S164–S165
7. Walraevens J, Demaerel P, Suetens P et al (2010) Longitudinal prospective long-term radiographic follow-up after treatment of single-level cervical disk disease with the Bryan cervical disc. Neurosurgery 67(3):679–687
8. Coric D, Nunley PD, Guyer RD et al (2011) Prospective, randomized, multicenter study of cervical arthroplasty: 269 patients from the Kineflex| C artificial disc investigational device exemption study with a minimum 2-year follow-up: clinical article. J Neurosurg: Spine 15(4):348–358
9. Zhao H, Cheng L, Hou Y et al (2014) Multi-level cervical disc arthroplasty (CDA) versus single-level CDA for the treatment of cervical disc diseases: a meta-analysis. European Spine Journal 1-12
10. Fukui M, Chiba K, Kawakami M et al (2007) Japanese orthopaedic association cervical myelopathy evaluation questionnaire (JOACMEQ): part 2. Endorsement of the alternative item. J Orthop Sci 12(3):241–248
11. McCormack HM, del Horne DJ, Sheather S (1988) Clinical applications of visual analogue scales: a critical review. Psychol Med 18(04): 1007–1019
12. Vernon H, Mior S (1991) The neck disability index: a study of reliability and validity. J Manip Physiol Ther 14(7):409–415
13. Guerin P, Obeid I, Bourghli A et al (2012) Heterotopic ossification after cervical disc replacement: Clinical significance and radiographic analysis. A prospective study. Acta Orthop Belg 78(1):80
14. Chang U K, Kim D H, Lee M C et al (2007) Changes in adjacent-level disc pressure and facet joint force after cervical arthroplasty compared with cervical discectomy and fusion
15. Gao Y, Liu M, Li T et al (2013) A meta-analysis comparing the results of cervical disc arthroplasty with anterior cervical discectomy and fusion (ACDF) for the treatment of symptomatic cervical disc disease. J Bone Joint Surg 95(6):555–561
16. Wada E, Suzuki S, Kanazawa A et al (2001) Subtotal corpectomy versus laminoplasty for multilevel cervical spondylotic myelopathy: a long-term follow-up study over 10 years. Spine 26(13):1443–1447
17. Veeravagu A, Cole T, Jiang B et al (2013) Revision rates and complication incidence in single-and multilevel anterior cervical discectomy and fusion procedures: an administrative database study. The spine journal: official journal of the North American Spine Society
18. Silber JS, Anderson DG, Daffner SD et al (2003) Donor site morbidity after anterior iliac crest bone harvest for single-level anterior cervical discectomy and fusion. Spine 28(2):134–139
19. Colle KO, Butler JB, Reyes PM et al (2013) Biomechanical evaluation of a metal-on-metal cervical intervertebral disc prosthesis. Spine J 13(11):1640–1649
20. Davis RJ, Kim KD, Hisey MS et al (2013) Cervical total disc replacement with the Mobi-C cervical artificial disc compared with anterior discectomy and fusion for treatment of 2-level symptomatic degenerative disc disease: a prospective, randomized, controlled multicenter clinical trial: clinical article. J Neurosurg: Spine 19(5): 532–545

21. Jin YJ, Park SB, Kim MJ et al (2013) An analysis of heterotopic ossification in cervical disc arthroplasty: a novel morphologic classification of an ossified mass. Spine J 13(4):408–420
22. Chalmers J, Gray DH, Rush J (1975) Observations on the induction of bone in soft tissues[J]. J Bone Joint Surg Br 57(1):36–45
23. Kaplan FS, Glaser DL, Hebela N et al (2004) Heterotopic ossification. J Am Acad Orthop Surg 12(2):116–125
24. Wenger M, Hoonacker P, Zachee B et al (2009) Bryan cervical disc prostheses: preservation of function over time. J Clin Neurosci 16(2): 220–225
25. Leung C, Casey AT, Goffin J et al (2005) Clinical significance of heterotopic ossification in cervical disc replacement: a prospective multicenter clinical trial. Neurosurgery 57(4):759–763
26. Heller J G, Park A E, Tortolani P J (2003) T-scan assessment of anterior paravertebral bone formation after total cervical disc replacement: temporal relationships and the effects of NSAID[C]//19th Annual Meeting of the Cervical Spine Research Society, Barcelona, Spain 18-19
27. Mehren C, Suchomel P, Grochulla F et al (2006) Heterotopic ossification in total cervical artificial disc replacement. Spine 31(24):2802–2806
28. Goffin J, Van Loon J, Van Calenbergh F (2006) 3: 57124. Cervical arthroplasty with the Bryan disc: 4-year results. Spine J 6(5):62S–63S
29. Su K K, Hwa H D (2013) Do Postoperative Biomechanical Changes Induce Heterotopic Ossification After Cervical Arthroplasty? A 5-year Follow-Up Study. Journal of spinal disorders & techniques
