# Supplementary material for: Adjacent segment degeneration or disease after cervical total disc replacement: a meta-analysis of randomized controlled trials
Source: J Orthop Surg Res. 2018 Oct 3;13:244. doi: 10.1186/s13018-018-0940-9 (PMC6169069; doi:10.1186/s13018-018-0940-9)
Supplement: Supplementary file 2 — File S1. Original data of 11 included articles. (ZIP 12 mb) [file 13018_2018_940_MOESM2_ESM.zip › 11 included articles and original data referred in this article/11 included articles/30 Davis, R J(US).pdf]

JNS SPINE

CLINICAL ARTICLE

J Neurosurg Spine 22:15–25, 2015

Two-level total disc replacement with Mobi-C cervical artificial disc versus anterior discectomy and fusion: a prospective, randomized, controlled multicenter clinical trial with 4-year follow-up results

Reginald J. Davis, MD,[1] Pierce Dalton Nunley, MD,[2] Kee D. Kim, MD,[3] Michael S. Hisey, MD,[4] Robert J. Jackson, MD,[5] Hyun W. Bae, MD,[6] Gregory A. Hoffman, MD,[7] Steven E. Gaede, MD,[8] Guy O. Danielson III, MD,[9] Charles Gordon, MD,[9] and Marcus B. Stone, PhD[2]

[1]Greater Baltimore Neurosurgical Associates, Baltimore, Maryland; [2]Spine Institute of Louisiana, Shreveport, Louisiana; [3]Department of Neurological Surgery, University of California, Davis, Sacramento, California; [4]Texas Back Institute, Plano, Texas; [5]Orange County Neurosurgical Associates, Laguna Hills, California; [6]Cedars-Sinai Spine Center, Los Angeles, California; [7]Orthopaedics Northeast, Fort Wayne, Indiana; [8]Oklahoma Brain and Spine Institute, Tulsa, Oklahoma; and [9]Precision Spine Care, Tyler, Texas

OBJECT The purpose of this study was to evaluate the safety and effectiveness of 2-level total disc replacement (TDR) using a Mobi-C cervical artificial disc at 48 months' follow-up.

METHODS A prospective randomized, US FDA investigational device exemption pivotal trial of the Mobi-C cervical artificial disc was conducted at 24 centers in the US. Three hundred thirty patients with degenerative disc disease were randomized and treated with cervical total disc replacement (225 patients) or the control treatment, anterior cervical discectomy and fusion (ACDF) (105 patients). Patients were followed up at regular intervals for 4 years after surgery.

RESULTS At 48 months, both groups demonstrated improvement in clinical outcome measures and a comparable safety profile. Data were available for 202 TDR patients and 89 ACDF patients in calculation of the primary endpoint. TDR patients had statistically significantly greater improvement than ACDF patients for the following outcome measures compared with baseline: Neck Disability Index scores, 12-Item Short Form Health Survey Physical Component Summary scores, patient satisfaction, and overall success. ACDF patients experienced higher subsequent surgery rates and displayed a higher rate of adjacent-segment degeneration as seen on radiographs. Overall, TDR patients maintained segmental range of motion through 48 months with no device failure.

CONCLUSIONS Four-year results from this study continue to support TDR as a safe, effective, and statistically superior alternative to ACDF for the treatment of degenerative disc disease at 2 contiguous cervical levels.

Clinical trial registration no.: NCT00389597 (clinicaltrials.gov)

http://thejns.org/doi/abs/10.3171/2014.7.SPINE13953

KEY WORDS cervical arthroplasty; anterior cervical discectomy and fusion; Mobi-C; artificial disc; degenerative disc disease; multilevel

ABBREVIATIONS ACDF = anterior cervical discectomy and fusion; AE = adverse event; CEC = clinical events committee; HO = heterotopic ossification; IDE = investigational device exemption; MCS = Mental Component Summary; NDI = Neck Disability Index; NSAID = nonsteroidal antiinflammatory drug; PCS = Physical Component Summary; ROM = range of motion; SEM = standard error of the mean; SF-12 = 12-Item Short Form Health Survey; TDR = total disc replacement; VAS = visual analog scale.

SUBMITTED October 25, 2013. **ACCEPTED** July 21, 2014.

INCLUDE WHEN CITING Published online November 7, 2014; DOI: 10.3171/2014.7.SPINE13953.

DISCLOSURE LDR contributed to the design and conduct of the study and also provided assistance with analysis of data and manuscript review. The authors also report receiving writing or editorial assistance for this paper from LDR. Dr. Davis has served as a consultant for LDR, has received clinical or research support for this study from LDR, and has received support for statistical analysis and for study/writing or editorial assistance on this paper from LDR. Dr. Bae is a patent holder for LDR Spine, has invested in a private fund that holds equity in LDR Spine among other medical companies, has received research support from LDR Spine, has served as a consultant for LDR Spine, and receives royalties for products from NuVasive, Stryker, and Zimmer Spine. Dr. Hisey serves as a consultant for LDR Spine, Zimmer Spine, DePuy/Synthes Spine, and Baxano Surgical and is a patent holder for LDR Spine and Zimmer Spine. Dr. Kim is a patent holder for LDR, and is a consultant for LDR, Globus, and Stryker. Dr. Nunley has direct stock ownership in Amedica, Paradigm Spine, and Spineology; has received support of non–study-related clinical or research effort from the Cervical Spine Research Society, K2M, Biomet Spine, Axiomed, Medtronic, Nutech, NuVasive, Spinal Motion, and Vertiflex; is a patent holder for K2M and LDR Spine (specifically for the ROI-A anterior lumbar interbody fusion cage); is a consultant for Nutech, Amedica, K2M, and LDR; and has received royalties, performed speaking and teaching arrangements, and served on the scientific advisory board of Osprey Biomedical and K2M. Dr. Hoffman has direct stock ownership in, and serves as a consultant to, LDR and Nanovis. Dr. Gaede received clinical or research support for this study from LDR. Dr. Danielson and Dr. Gordon have received clinical or research support for this study from LDR. Dr. Jackson is a consultant for LDR Spine and has received clinical or research support for this study from LDR. Dr. Stone did not receive research support from LDR for this paper but has received support for other related research.

©AANS, 2015

Anterior cervical discectomy and fusion (ACDF) remains a standard surgical treatment for symptomatic radiculopathy and myelopathy caused by degenerative disc disease.[1,2,4,10,29] However, ACDF eliminates the natural motion of treated segments and has been shown to induce hypermobility and heightened intradiscal pressures at adjacent levels.[6–8,19] Changes in stress and motion profiles are hypothesized to be a primary cause for adjacent-segment degeneration observed in ACDF patients.[13,17,25] By preserving treated segment natural motion as well as overall cervical spine biomechanics, total disc replacement (TDR) may avoid exacerbating adjacent segment degeneration and related symptoms while providing mechanical stability following neural decompression.[14,22] As the number of treated segments increases from one level to two contiguous levels, the available clinical data comparing TDR to ACDF diminish greatly. This paper presents the 4-year follow-up results of a prospective, randomized, controlled FDA investigational device exemption (IDE) trial. This study included the largest known cohort of randomized patients treated with TDR compared with ACDF at 2 contiguous levels under a prospectively controlled clinical study protocol.

The Mobi-C cervical artificial disc (LDR Medical; Troyes, France) is a semiconstrained, mobile bearing, bone-sparing TDR (Fig. 1). Previous 2-year follow-up results[5] have shown TDR to be safe and effective for use in indicated patients with specific clinical advantages over ACDF. Furthermore, 2-level TDR has been shown to produce statistically better outcomes for both pain and function within a 2-year window.[5] Extensive outside the United States (OUS) experience with TDR further supports the observation of enhanced clinical outcomes.[15] Here, we test the hypothesis that the clinical advantages of 2-level TDR demonstrated at 2 years will be maintained through 4 years.

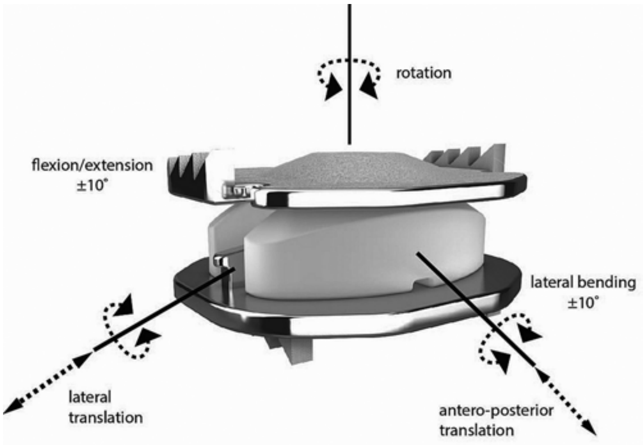

FIG. 1. Mobi-C cervical artificial disc with 2 cobalt-chromium-molybdenum alloy endplates and an ultra-high-molecular-weight polyethylene mobile insert facilitating 5 independent degrees of freedom. Reproduced with permission from Davis et al: *J Neurosurg Spine, 19:*532–545, 2013.

Methods

This trial was approved by the Western Institutional Review Board (for 8 of the 24 participating sites) and local institutional review boards (for the remaining 16 sites). It was registered with the ClinicalTrials.gov database (http://clinicaltrials.gov), and its registration number is NCT00389597.

Subjects and Study Design

A full description of the study design and surgical technique was previously reported.[5] Patients underwent surgery between April 2006 to March 2008 at 24 clinical sites in the US as part of the FDA IDE randomized, controlled clinical trial. Enrollment required a diagnosis of degenerative disc disease with radiculopathy or myeloradiculopathy at 2 contiguous levels from C-3 to C-7 that was unresponsive to nonoperative treatment for at least 6 weeks or demonstrated progressive symptoms calling for immediate surgery (Table 1). Exclusion criteria included any prior cervical spine surgery (Table 2). After giving informed consent, patients were randomized in a 2:1 ratio (TDR patients to ACDF patients), resulting in 225 patients receiving treatment with a Mobi-C cervical artificial disc and 105 patients receiving corticocancellous allograft and an anterior cervical plate using the standard ACDF technique. The total randomized population of 330 patients is the primary analysis population. An additional 9 patients were treated with the TDR device as training cases. The safety population of 339 patients is comprised of these 9 training patients and the 330 randomized patients. It was not possible to blind the surgeons to the treatment; however, patients were blinded until after surgery.

Postoperative care was left to the discretion of the treating surgeon. All patients were prescribed a rehabilitation program aimed at returning the patient to normal activity as soon as possible. Patients were evaluated preoperatively and at 6 weeks, 3 months, 6 months, 12 months, 18 months, 24 months, 36 months, and 48 months postoperatively. Both groups were asked to refrain from taking nonsteroidal antiinflammatory drugs (NSAIDs) from a week before surgery to 3 months after surgery. An exception was made for TDR patients diagnosed with heterotopic ossification (HO) after surgery.

Clinical Outcomes

Outcomes measures used to evaluate pain, function, patient satisfaction, and overall clinical success included the Neck Disability Index (NDI), visual analog scales (VASs) for neck and arm pain, the SF-12 (12-Item Short Form Health Survey) Mental Component Summary (MCS) and SF-12 Physical Component Summary (PCS), subsequent surgical intervention, complications, neurological function, return to work, radiographic success, patient satisfaction, range of motion (ROM), HO, and adjacent-segment degeneration. All radiographic evaluations were conducted through Medical Metrics, Inc. (MMI) utilizing independent radiologists and validated software.[24] Adjacent-segment degeneration was determined by the Kellgren-Lawrence scale of disc degeneration.[3,16] Adverse events (AEs) were considered to be any clinically adverse sign, symptom, syndrome, or illness that occurred or worsened during or after the initial surgery, regardless of cause. All AEs were evaluated and classified by the clinical events committee (CEC), which was composed of 2 orthopedic surgeons and 1 neurosurgeon.

TABLE 1. Study inclusion criteria

Inclusion Criteria
Age 18–69 yrs
Symptomatic cervical degenerative disc disease in two contiguous levels btwn C-3 & C-7 w/<br>Neck &/or arm pain &/or<br>Decreased muscle strength &/or<br>Abnormal sensation &/or abnormal reflexes
Deficit confirmed by imaging (CT, MRI, or plain radiographs)
NDI score of ≥30
Unresponsive to nonoperative, conservative treatment for at least 6 wks or presence of progressive symptoms or signs of nerve root/spinal cord compression despite continued nonoperative treatment
No prior surgery at the operative level & no prior cervical fusion procedure at any level
Physically & mentally able & willing to comply w/ the protocol
Signed informed consent
Willingness to discontinue all use of NSAIDs from 1 wk before surgery until 3 mos after surgery

Neurological function was determined by the investigator through tests of reflex, motor, and sensory function. Neurological success was defined as the absence of significant neurological deterioration. These evaluations included sensory assessments with pinprick and light touch, motor assessments of muscle strength, and reflex assessments of the treated levels.

Patient satisfaction was determined by a questionnaire, which asked patients if they were very satisfied, somewhat satisfied, somewhat dissatisfied, or very dissatisfied with their treatment. Patients were also asked if they would definitely, probably, probably not, or definitely not recommend the same treatment method to a friend with the same symptoms and indications.

Subsequent surgical intervention was considered to be any secondary surgery at an index-level segment and was classified as a removal, revision, supplemental fixation, or reoperation. Adjacent-level subsequent surgeries that did not involve a treated level did not indicate a study failure and were documented for further analysis. Radiographic success for the ACDF group was defined as fusion of both treated levels—less than 2° of angular motion in flexion/extension and evidence of bridging bone across the disc space and radiolucent lines at no more than 50% of the graft vertebral interfaces. Radiographic success for the TDR group was defined at least 2° angular motion in flexion/extension or no evidence of bridging trabecular bone across the disc space. ROM was determined from lateral flexion/extension and anteroposterior right/left lateral bending radiographs at each treated level.

TABLE 2. Study exclusion criteria

Exclusion Criteria
More than 2 vertebral levels requiring treatment/immobile level btwn C-1 & C-7 from any cause
Any prior spine surgery at operative level of any prior cervical fusion at any level
Disc height <3 mm
t-score less than −1.5 (osteoporosis evaluation)
Paget’s disease, osteomalacia, or any other metabolic bone disease other than osteoporosis
Active systemic infection of surgical site or history of or anticipated treatment for systemic infection including HIV/hepatitis C
Active malignancy: a history of any invasive malignancy (except nonmelanoma skin cancer), unless treated w/ curative intent & there had been no clinical signs or symptoms of the malignancy >5 yrs
Marked cervical instability on resting lateral or flexion/extension radiographs
Known allergy to cobalt, chromium, molybdenum, or polyethylene
Segmental angulation >11° at treatment or adjacent levels
Rheumatoid arthritis, lupus, or other autoimmune disease
Any diseases or conditions that would preclude accurate clinical evaluation
Daily, high-dose oral &/or inhaled steroids or a history of chronic use of high-dose steroids
BMI >40
Use of any other investigational drug or medical device w/in 30 days prior to surgery
Pending personal litigation relating to spinal injury (workers’ compensation not included)
Smoking >1 pack of cigarettes per day
Reported to have mental illness or belonged to a vulnerable population

BMI = body mass index.

Overall success rates for both treatments were determined from a composite endpoint with multiple conditions including: 1) ≥ 30-point improvement for patients with baseline NDI ≥ 60 or 50% improvement for patients with baseline NDI < 60; 2) no subsequent surgical intervention at either treated level; 3) AEs assessed by the CEC as major complications; 4) maintenance or improvement in neurological function; and 5) radiographic success. Patients not meeting one or more of these 5 outcome metrics were deemed treatment failures for study purposes. A more detailed description of the overall success components has been previously defined.[5]

Statistical Methods

The study included a noninferiority hypothesis of the overall success rate of the investigational procedure versus that of the control procedure. A power analysis was performed in determine sample size. Assuming a 2:1 randomization rate, a total sample size of 294 randomized patients (196 TDR and 98 ACDF) was required. Noninferiority was assessed using an exact 95% one-sided confidence bound. A post hoc test was preplanned to test for superiority in the event of noninferiority. Superiority was assessed using a 97.5% one-sided confidence bound in the event a 10% noninferiority margin could be excluded.

Two-sided t-tests were used to determine statistical significance for all continuous outcome measures between groups at each time point. Fisher's exact tests were used to determine success or incident rates. Wilcoxon signed-rank tests were used to compare the change from baseline within treatment groups. A p value less than 0.05 was considered significant. Mean values are presented with standard deviations unless otherwise indicated.

Results

Patient Accountability and Baseline Demographics

Three hundred forty-seven patients were enrolled and randomly assigned to a group within the study. Of the randomized patients, 225 TDR patients and 105 ACDF patients underwent surgery, totaling 330 randomized and treated patients. Nine additional patients also underwent a TDR procedure as training cases and are not included in the randomized population. All results presented include comparison of the randomized populations only. Baseline demographic characteristics were evenly matched across each group with no statistically or clinically significant differences (Table 3). The 48-month follow-up rate was 89.0% for the TDR group and 81.2% for the ACDF group.

Neck Disability Index

Groups had similar mean NDI scores at baseline and both showed significant improvement at every time point through 48 months ($p < 0.0001$). The TDR group showed significantly greater improvement in NDI score in comparison with the ACDF group at every postoperative time point (Fig. 2). At 48 months, the mean improvement in NDI score was 36.5 ± 21.3 for the TDR group and 28.5 ± 18.3 for the ACDF group ($p = 0.0048$). The TDR NDI success rate was significantly higher than the ACDF NDI success rate at every time point ($p < 0.05$) including 48 months at which the NDI success rates were 79.3% (TDR) and 53.4% (ACDF) ($p < 0.0001$).

Neck and Arm Pain VAS Scores

The TDR and the ACDF group had similar VAS neck and arm pain scores at baseline and both demonstrated significant improvement from baseline in both measures through the 48-month follow-up period ($p < 0.0001$). The TDR group had lower VAS neck pain scores than the ACDF group at every time point (Fig. 3 upper), and statistically significant differences were noted at 3 months and 6 months in favor of the TDR group. The mean improvement in VAS neck pain score from baseline at 48 months was 53 ± 30 for the TDR group and 48 ± 29 for the ACDF group. Mean VAS arm pain scores (Fig. 3 lower) were derived from the most symptomatic arm at baseline, carried through 48 months. The mean improvement in VAS arm pain score from baseline was similar between groups with 56 ± 31 for TDR patients and 53 ± 31 for ACDF patients.

SF-12 MCS and PCS Scores

The two treatment groups showed similar and significant improvement in SF-12 MCS scores from baseline at all time points ($p < 0.0001$). At 48 months, the TDR group had a mean SF-12 MCS score improvement of 11 ± 12 versus an improvement of 10 ± 12 for the ACDF group (Fig. 4 upper). Both groups also showed significant improvement through 48 months in SF-12 PCS scores ($p < 0.0001$). However, with statistically similar scores at baseline, the TDR group showed significantly greater improvement in SF-12 PCS scores than the ACDF group at all postoperative time points (Fig. 4 lower). The mean improvement from baseline SF-12 PCS scores was 13 ± 12 for the TDR group and 10 ± 12 for the ACDF group at 48 months ($p < 0.05$).

Neurological Success

Groups had similar scores at baseline, and at the 48-month follow-up period, 6.2% of TDR patients experienced neurological deterioration compared with 7.6% in the ACDF group. This difference was not statistically significant.

Return to Work

Time to return to work was calculated for working patients (191 in the TDR group and 86 in the ACDF group) and is defined as the time from their surgery date to the date they started working. Although the differences were not statistically significant, on average TDR patients returned to work 21 days earlier than ACDF patients, with an average return to work time of 46 ± 101 days for TDR patients and 67 ± 113 days for ACDF patients.

Patient Satisfaction

At 48 months, the percentage of patients very satisfied or somewhat satisfied with their treatment was higher in the TDR group (96.4%) than in the ACDF group (89.0%, $p = 0.03329$). Additionally, 95.9% of TDR patients and 86.3% of ACDF patients would definitely or probably recommend their treatment to a friend ($p = 0.01112$).

TABLE 3. Patient demographic characteristics at baseline

Variable	Patient Group*		p Value†
	TDR (n = 225)	ACDF (n = 105)	
Mean age in yrs (SD)	45.3 (8.1)	46.2 (8.0)	0.3725
Sex, no. (%)			0.2375
Male	113 (50.2%)	45 (42.9%)	
Female	112 (49.8%)	60 (57.1%)	
Ethnicity, no. (%)			>0.9999
Hispanic or Latino	14 (6.2%)	7 (6.7%)	
Not Hispanic or Latino	211 (93.8%)	98 (93.3%)	
Race,‡ no. (%)			>0.9999
American Indian	3 (1.3%)	1 (1.0%)	
Caucasian	212 (94.2%)	99 (94.3%)	
Asian	4 (1.8%)	0 (0.0%)	
Black or African American	5 (2.2%)	4 (3.8%)	
Native Hawaiian/other Pacific Islander	0 (0.0%)	0	
Other	1 (0.4%)	1 (1.0%)	
Mean BMI (SD)	27.6 (4.5)	28.1 (4.2)	0.3586
Work status,§ no. (%)			>0.9999
Able to work	141 (62.7%)	64 (61.0%)	
Not able to work	50 (22.2%)	22 (21.0%)	
NA	34 (15.1%)	19 (18.1%)	
Workers' compensation,§ no. (%)			0.1586
Receiving	11 (4.9%)	7 (6.7%)	
Not receiving	214 (95.1%)	98 (93.3%)	
Driving status,§ no. (%)			
Able to drive	210 (93.3%)	102 (97.1%)	0.4026
Not able to drive	12 (5.3%)	3 (2.9%)	
NA	3 (1.3%)	0	
Smoking status,§ no. (%)			
<1 pack per day	225 (100%)	105 (100%)	>0.9999
>1 pack per day	0 (0%)	0 (0%)	

NA = data not available.

* Values represent numbers of patients (%) unless otherwise indicated.

† The unpaired t-test was used to compare age and BMI across treatment groups, whereas the Fisher exact test was used to compare frequencies between treatment groups for all other variables.

‡ Patients with multiple races are included with the non-Caucasian group. Fisher exact p value calculation is based on Caucasian versus non-Caucasian.

§ Fisher exact p value is based on "able to" versus "not able to."

Major Complications

The CEC deemed AEs a major complication in 4.0% of the TDR group and 7.6% of the ACDF group at 48 months. This difference was not statistically significant.

Subsequent Surgical Intervention

The most common reason for subsequent surgery in the ACDF group was symptomatic pseudarthrosis, with 8.6% of ACDF patients receiving subsequent surgical intervention due to pseudarthrosis. The reasons for removal of artificial discs in the TDR group included stenosis, device migration, poor endplate fixation, and persistent neck and/or shoulder pain. At 48 months, the cumulative percentage of patients who underwent subsequent surgeries at the index level remained significantly lower ($p < 0.0001$) for the TDR group—4.0% (9 of 225 patients, with 10 surgeries)—than for the ACDF group—15.2% (16 of 105 patients, 18 surgeries).

Radiographic Outcomes

On average, TDR patients maintained their baseline ROM in flexion/extension (Fig. 5 upper) and lateral bending (Fig. 5 lower) at both segments. For TDR patients, the mean ROM at the superior level was 10.0° ± 6.0° in flexion/extension (baseline 9.1° ± 4.9°) and 5.5° ± 3.6° in lateral bending (baseline 5.8° ± 3.4°) at 48 months. At the inferior level, the mean ROM was 8.2° ± 5.3° in flexion/extension (baseline 7.4° ± 4.3°) and 5.1° ± 3.4° in right/left lateral bending (baseline 4.9° ± 3.3°) at 48 months.

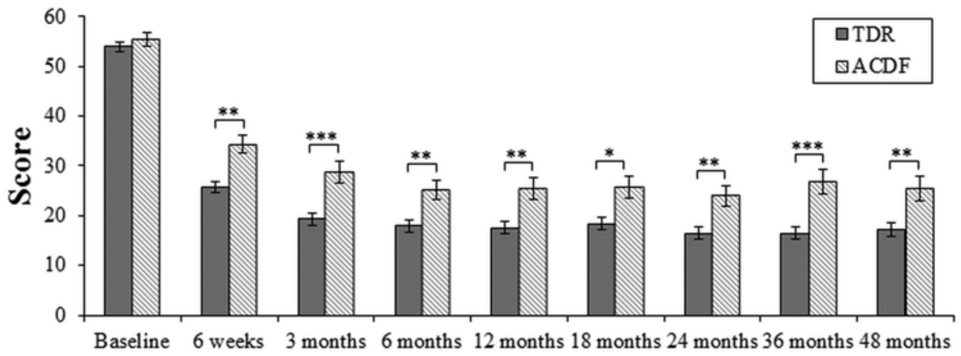

FIG. 2. Mean NDI scores at baseline and follow-up time points through 48 months. Mean NDI improvement was significantly greater in the TDR group than in the ACDF group at all postoperative time points. *Asterisks* denote statistically significant differences between treatment groups with respect to NDI improvement from baseline as determined by an unpaired, 2-sided t-test (*p < 0.05, **p < 0.01, ***p < 0.0001). *Error bars* represent standard error of the mean (SEM).

At 48 months, the results in 14 TDR patients and 11 ACDF patients failed to meet the criteria for radiographic success. Clinically relevant HO (Grades III and IV) was observed in 25.6% of 187 TDR patients with available radiographs at the 48-month time point, with Grade IV HO being evident in 10.2% of cases in at least 1 level (Fig. 6 left). TDR patients experienced clinically relevant HO in 15.4% of superior levels and 17.7% of inferior levels, totaling 16.6% (62/374) of treated levels.

In the ACDF cohort, fusion status was not achieved at 48 months in 14.8% of the 81 patients with available radiographs. ACDF patients experienced failed fusion in 13.5% of inferior levels and 2.5% of superior levels, totaling 7.8% of treated levels. This does not include patients who had corrective surgery for failed fusion at earlier time points (Fig. 6 right).

Adjacent-segment degeneration was defined as an increase of 1 or more points of the Kellgren-Lawrence grad-

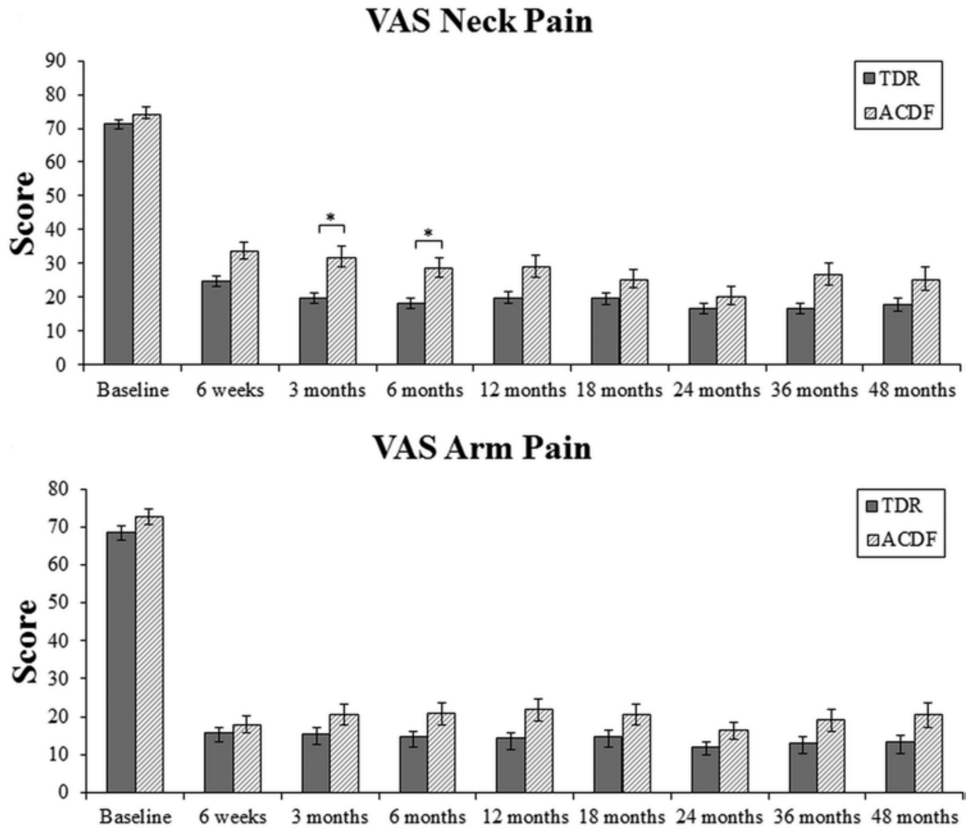

FIG. 3. Mean VAS neck pain scores **(upper)** and arm pain scores **(lower)** at baseline and follow-up time points through 48 months. *Asterisks* denote statistically significant differences between treatment groups with respect to VAS neck pain improvement from baseline as determined by an unpaired, 2-sided t-test (*p < 0.05). *Error bars* represent SEM.

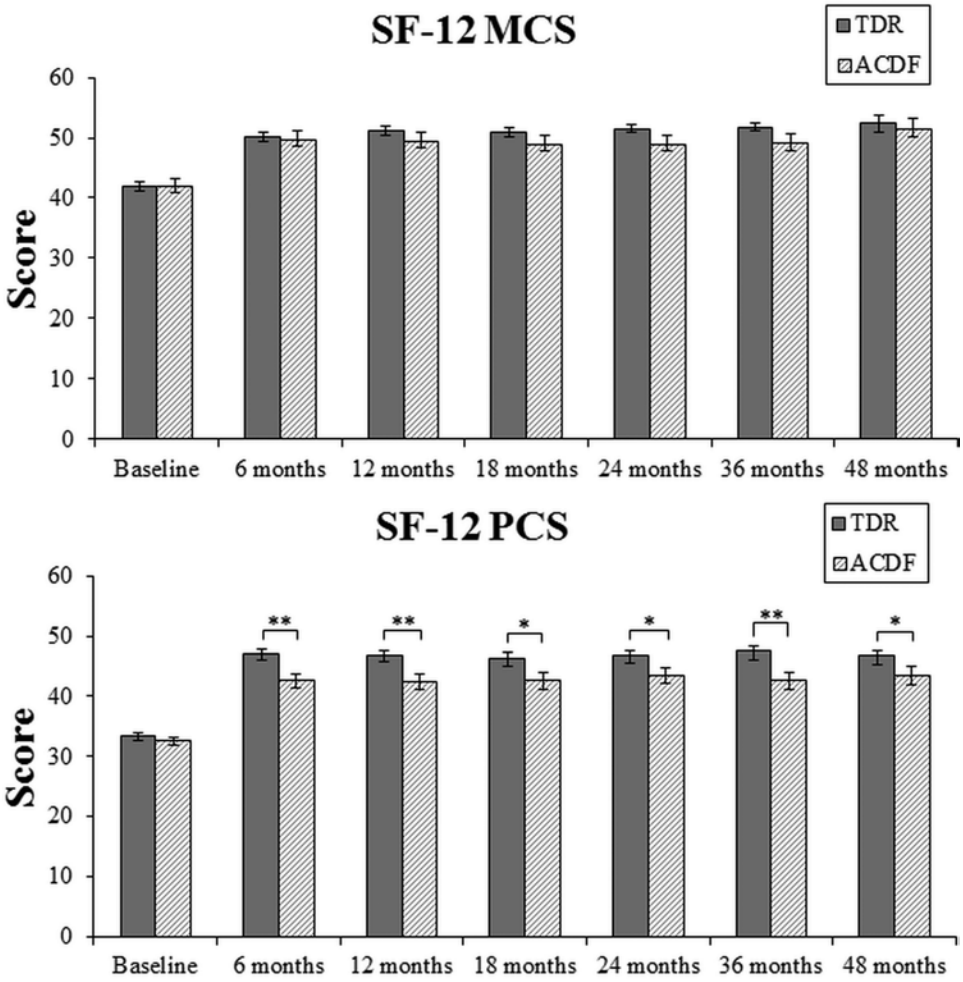

FIG. 4. Mean SF-12 MCS scores **(upper)** and PCS scores **(lower)** at baseline through 48 months. On average, the TDR group showed significantly greater improvement in SF-12 PCS scores. *Asterisks* denote statistical significantly differences in SF-12 PCS score improvement from baseline between treatments as determined by an unpaired, 2-sided t-test (*p < 0.05, **p < 0.01). *Error bars* represent SEM.

ing scale at either segment when compared with baseline values. Rates of adjacent-segment degeneration did not differ significantly between treatment groups for any grade of degeneration or combination of grades at baseline. The superior levels at 48 months indicated degeneration in 64.7% of the ACDF patients and 27.6% of the TDR patients, a significant difference (p < 0.0001). Results for the inferior levels were similar at 56.2% for the ACDF group and 16.4% for the TDR group (p < 0.0001). When reviewing cases in which patients experienced degeneration at either level, TDR patients were found to have significantly less adjacent-segment degeneration than the ACDF group (41.5% for TDR vs 85.9% for ACDF, p < 0.0001).

No cases of migration or subsidence have been reported since the 24-month results. The one case of posterior migration was reported previously and occurred prior to 24 months.

Overall Success

The results of the overall success criteria—applied to both treatment groups—incorporate the success rates of each of the 5 individual components of this composite endpoint. The results of this composite endpoint (Fig. 7) show at the 48-month follow-up period, 66.0% of the TDR group and 36.0% of the ACDF group achieved overall success based on the predefined criteria (p < 0.0001). While TDR showed better outcomes for all 5 components, only the NDI success and secondary surgery components were statistically significant in favor of TDR. NDI was the leading cause of failure in the ACDF group, with the NDI improvement criteria not met in 46.6% of cases in the ACDF group and 20.7% in the TDR group.

Discussion

Here, we investigated the safety and efficacy of 2-level TDR using 2 Mobi-C cervical artificial discs and compared the results to the standard surgical treatment, ACDF. Results were presented through 48 months as part of an ongoing randomized, controlled, clinical trial. This trial was an FDA, IDE trail and included the first Level 1 evidence[21] of 2-level TDR through 48 months. Treatment with

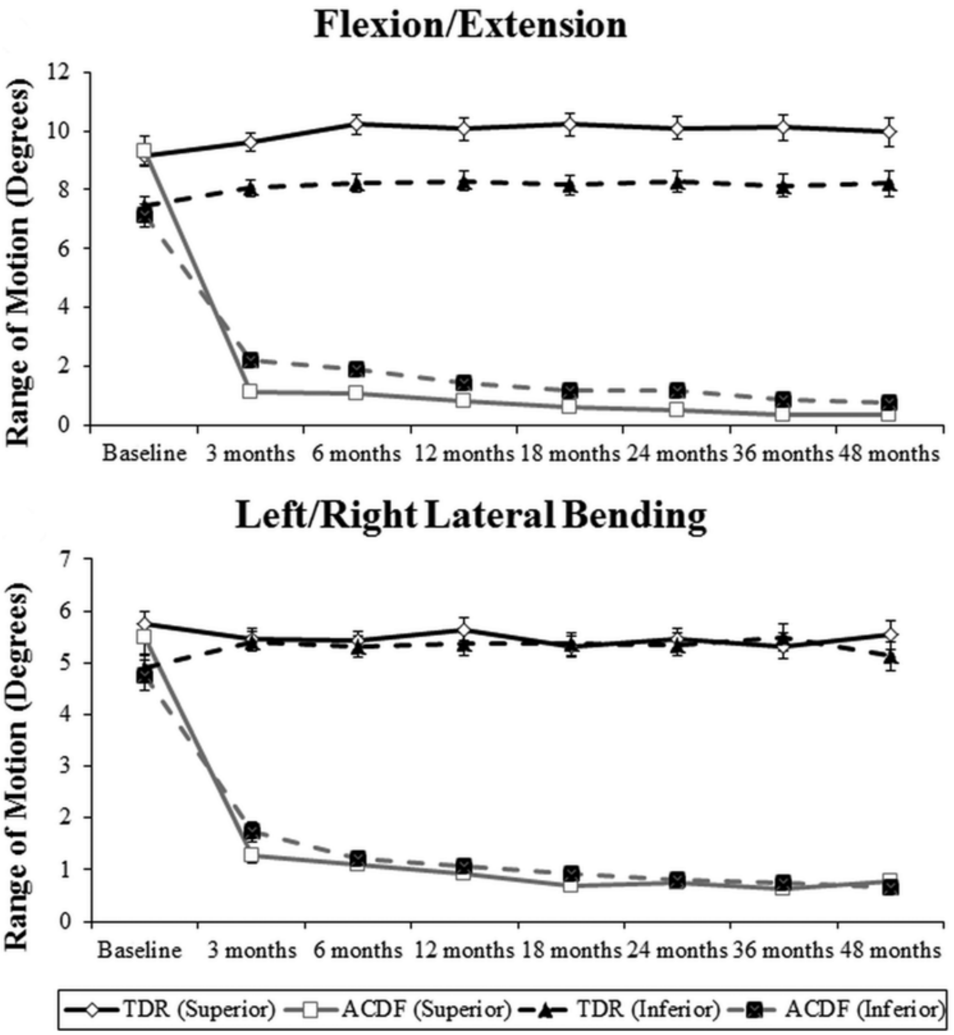

FIG. 5. ROM in both flexion/extension **(upper)** and left/right lateral bending **(lower)** through 48 months. As a whole, the TDR group maintained baseline ROM in both flexion/extension and left/right lateral bending while the ACDF group had a dramatic reduction in both measures. *Error bars* represent SEM.

TDR is statistically superior when compared with ACDF, as predefined by the study statistical plan, with regard to overall success rate. At 48 months both ACDF and TDR patients experienced significant improvement from baseline. TDR demonstrated statistically better results at 48 months for NDI scores, SF-12 PCS scores, patient satisfaction, subsequent surgery rates, adjacent-segment degeneration, and overall success.

The 48-month results of this 2-level study confirm the hypothesis that the previously reported results from the same study at 24 months will continue through 48 months. Statistically significant differences were maintained between 24 and 48 months for NDI scores, SF-12 PCS scores, patient satisfaction, subsequent surgery rates, adjacent-segment degeneration, and overall success. In addition, the 2-level TDR results concur with those of single-level TDR IDE study results.[11,20] Garrido et al.[11] reported similar or better outcomes for their single-level TDR group with respect to NDI, neck pain, arm pain, and secondary surgery rates in a 48-month study of the BRYAN Cervical Disc (Medtronic Sofamor Danek).

The overall success of TDR, a composite outcome consisting of 5 components, was statistically superior ($p < 0.0001$) to ACDF at 48 months with 66.0% success versus 36.0%. NDI success and subsequent surgeries are the main components creating the statistical differences in overall success between TDR and ACDF. The significant differences in NDI success was maintained from 24 months to 48 months ($p < 0.0001$). Previously reported at 24 months, 3.1% of TDR patients required subsequent surgeries compared with 11.4% of ACDF patients. This difference is widened at 48 months with 4.0% of TDR patients and 15.2% of ACDF patients requiring at least 1 subsequent surgery.

Heterotopic ossification is a primary concern for TDR. In the current study, we observed clinically relevant HO (Grades III and IV) in 16.6% of segments and 25.6% of patients treated with TDR, which is similar to or less than the rates in other reports.[23,26,28] HO was present in 15.5% of patients and 10.7% of segments as Grade III and 10.1% of patients and 5.9% of segments as Grade IV. A European study investigating HO rates in Prodisc-C (Synthes Spine

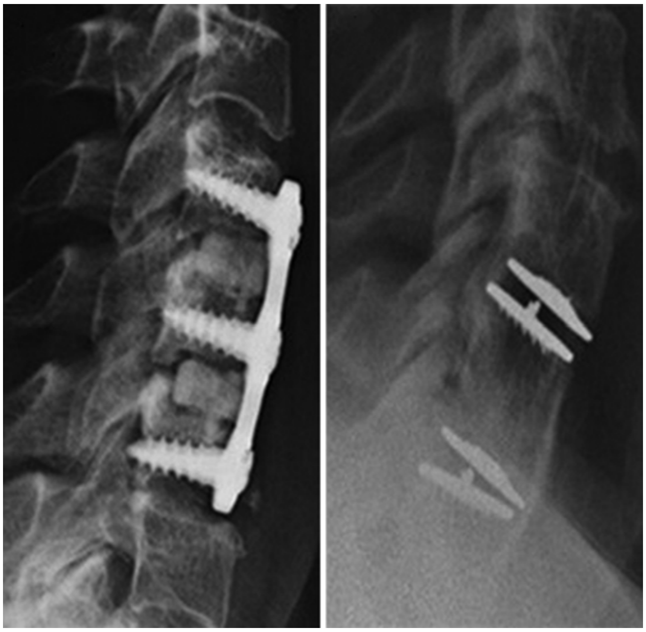

FIG. 6. Neutral-position lateral radiographs obtained in a TDR patient with clinically relevant HO **(left)** and an ACDF patient with pseudarthrosis **(right)**.

Company, L.P.) patients reported clinically relevant HO was observed in a combined 63% of treated levels (Grade III = 45%, Grade IV = 18%) at 4 years.[26] Another study of 21 patients treated with a BRYAN Cervical Disc reported an overall 42.9% incidence of Grade III (14.3%) and IV (28.6%) HO at 8 year follow-up.[23] While the 5-year results of ProDisc-C in the US showed Grade IV HO present at the index level in 6 of the 103 patients, Grade III HO was not reported, leaving the overall picture for clinically relevant HO for this study unclear.[28] One major difference that should be noted between the current study and the others mentioned is the use of NSAIDs. The use of NSAIDs has been shown to decrease HO formation. Other device trials did not restrict the use of NSAIDS postsurgery. However, in this study patients were asked to refrain from using NSAIDs before and after surgery. Regardless, comparisons among TDR devices are difficult given the limited amount of data on HO rates for TDR at 4 or more years. In any case, HO is still a concern for the TDR procedure and further long-term investigations are needed to clarify HO rates and the effects of HO on clinical outcomes.

Adjacent-segment degeneration is another major concern for patients undergoing surgery for degenerative disc disease. Although ACDF is associated with higher rates of adjacent-segment degeneration, the underlying mechanisms are ill defined. We report an adjacent-segment degeneration rate of 85.9% for ACDF patients and 41.5% for TDR patients at 48 months postsurgery. While TDR may not entirely prevent adjacent-segment degeneration, the advancement of the radiographic degeneration is slowed with TDR. It is still widely debated whether the preservation of the adjacent-segment with TDR stems from the preserved biomechanics at the index and adjacent levels. The underlying mechanism defining the relationship between decreased radiographic degeneration in patients treated with TDR remains uncorrelated, and further long-term follow-up should continue to correlate these results.

With 2-level TDR only recently being approved in the US for degenerative disc disease, published data for direct comparison is limited. However, long-term data on single-level TDR with long-term have shown it to be at least as effective as a single-level ACDF in relieving the neurological pain and motor impairment caused by degenerative disc disease.[12,20] There are follow-up data out to 5 years postsurgery for TDR devices, showing safe and effective outcomes.[28] One recent study indicates that single-level TDR is a superior treatment to ACDF.[9] Also, a growing amount of evidence has shown that ACDF efficacy decreases as more levels become involved and that ACDF at multiple levels may result in higher stresses and hypermobility at adjacent segments.[18,27] These stressors could be a primary factor for an increase in the rate of adjacent-segment degeneration observed in patients treated with ACDF. Because 2-level TDR preserves cervical mobility at treated levels, it potentially avoids inducing the excess motion and stresses at adjacent levels seen in ACDF patients.[5]

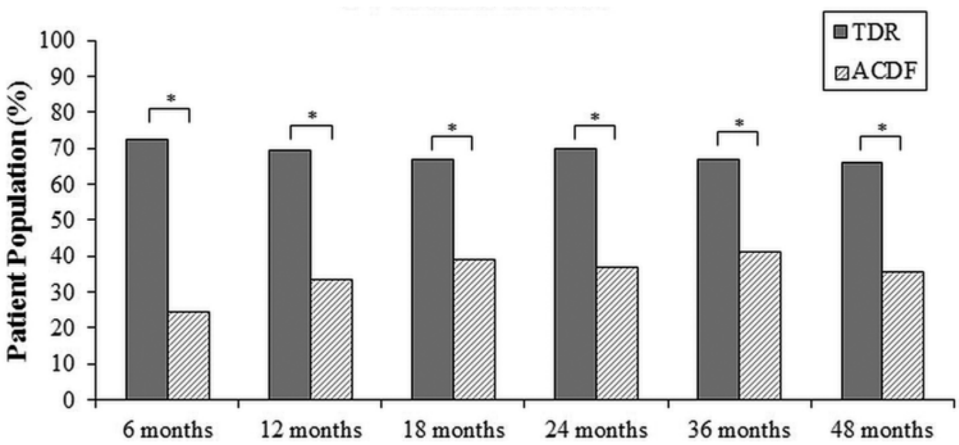

FIG. 7. Overall success for the TDR and ACDF groups through 48 months. The TDR group had significantly higher success rates than the ACDF group at every time point. *Asterisks* denote statistical significantly differences in overall success rate between treatments as determined by a Fisher exact test (*p < 0.0001).

All of the authors participated as investigators in the Mobi-C IDE clinical study. To ensure that any potential conflicts of interest have not affected study outcomes, an analysis was completed at each time point to compare the overall success rates of patients between sites with and without financial interest (as defined by FDA regulation 21CFR54). Additionally, this analysis was reported to the FDA as part of the premarket approval package through the 24-month primary endpoint. At 6, 12, 18, 24, 36, and 48 months, this analysis confirmed that financial interest was not indicative of a better (or worse) outcome for either treatment group ($p = 0.3132$ at 48 months).

Conclusions

The data from this IDE study through 48 months signify a number of clinically relevant benefits for TDR over ACDF. Patients experienced improved clinical outcomes with TDR—including improvement in pain and function outcomes and superiority in overall primary endpoint success. Additionally, incidences of adjacent-segment degeneration and subsequent surgeries were reduced with TDR. We expect that future studies and also longer-term follow-up of this patient cohort may continue to establish 2-level cervical TDR as a superior surgical option for symptomatic degenerative disc disease.

Acknowledgments

We would like to thank the other principal investigators for their contributions to the study: Daniel Peterson, MD, Austin Brain and Spine, Austin, Texas; Ralph Rashbaum, MD, Texas Back Institute, Plano, Texas; Arnold Schwartz, MD, Orthopedic Spine Care of Long Island, Huntington Station, New York; Ali Araghi, MD, The Core Institute, Phoenix, Arizona; David Tahernia, MD, Desert Orthopedic Center, Rancho Mirage, California; Hazem Eltahawy, MD, University Neurologic Systems, Wayne State University, Detroit, Michigan; Reginald Tall, MD, Southeastern Clinical Research, Orlando, Florida; Douglas Wong, MD, Panorama Orthopedics & Spine Center, Golden, Colorado; Gerald Schell, MD, St. Mary's of Saginaw, Field Neurosciences Institute, Saginaw, Michigan; Michael Ramsey, MD, West Texas Spine, Odessa, Texas; B. Christoph Meyer, MD, Houston Orthopedic Hospital, Bellaire, Texas; Robert McLain, MD, Cleveland Clinic, Cleveland, Ohio; Jon Park, MD, Stanford Hospital and Clinics, Stanford, California; Ed Simmons, MD, Simmons Orthopaedics and Spine Associates, Buffalo, New York; Mark Stern, MD, California Institute of Neurological Surgery, Escondido, California; and Phillip S. Yuan, MD, Memorial Orthopaedic Surgical Group, Long Beach, California. The principal author would also like to thank Kelly Frank, MS, of LDR Spine, Austin, Texas, for her assistance with data analysis and manuscript review.

References

1. Brodke DS, Zdeblick TA: Modified Smith-Robinson procedure for anterior cervical discectomy and fusion. **Spine (Phila Pa 1976) 17 (10 Suppl):**S427–S430, 1992
2. Brown JA, Havel P, Ebraheim N, Greenblatt SH, Jackson WT: Cervical stabilization by plate and bone fusion. **Spine (Phila Pa 1976) 13:**236–240, 1988
3. Croft P: An introduction to the Atlas of Standard Radiographs of Arthritis. **Rheumatology (Oxford) 44 Suppl 4:**iv42, 2005
4. Cummins BH, Robertson JT, Gill SS: Surgical experience with an implanted artificial cervical joint. **J Neurosurg 88:**943–948, 1998
5. Davis RJ, Kim KD, Hisey MS, Hoffman GA, Bae HW, Gaede SE, et al: Cervical total disc replacement with the Mobi-C cervical artificial disc compared with anterior discectomy and fusion for treatment of 2-level symptomatic degenerative disc disease: a prospective, randomized, controlled multicenter clinical trial. Clinical article. **J Neurosurg Spine 19:**532–545, 2013
6. DiAngelo DJ, Roberston JT, Metcalf NH, McVay BJ, Davis RC: Biomechanical testing of an artificial cervical joint and an anterior cervical plate. **J Spinal Disord Tech 16:**314–323, 2003
7. Dmitriev AE, Cunningham BW, Hu N, Sell G, Vigna F, McAfee PC: Adjacent level intradiscal pressure and segmental kinematics following a cervical total disc arthroplasty: an in vitro human cadaveric model. **Spine (Phila Pa 1976) 30:**1165–1172, 2005
8. Elsawaf A, Mastronardi L, Roperto R, Bozzao A, Caroli M, Ferrante L: Effect of cervical dynamics on adjacent segment degeneration after anterior cervical fusion with cages. **Neurosurg Rev 32:**215–224, 2009
9. Food and Drug Administration: **Summary of Safety and Effectiveness Data (SSED): SECURE®-C Cervical Artificial Disc.** (http://www.accessdata.fda.gov/cdrh_docs/pdf10/p100003b.pdf) [Accessed July 28, 2014]
10. Fraser JF, Härtl R: Anterior approaches to fusion of the cervical spine: a metaanalysis of fusion rates. **J Neurosurg Spine 6:**298–303, 2007
11. Garrido BJ, Taha TA, Sasso RC: Clinical outcomes of Bryan cervical disc arthroplasty a prospective, randomized, controlled, single site trial with 48-month follow-up. **J Spinal Disord Tech 23:**367–371, 2010
12. Heller JG, Sasso RC, Papadopoulos SM, Anderson PA, Fessler RG, Hacker RJ, et al: Comparison of BRYAN cervical disc arthroplasty with anterior cervical decompression and fusion: clinical and radiographic results of a randomized, controlled, clinical trial. **Spine (Phila Pa 1976) 34:**101–107, 2009
13. Hilibrand AS, Carlson GD, Palumbo MA, Jones PK, Bohlman HH: Radiculopathy and myelopathy at segments adjacent to the site of a previous anterior cervical arthrodesis. **J Bone Joint Surg Am 81:**519–528, 1999
14. Hsu WK: The rationale for cervical disc arthroplasty. **Tech Orthop 25:**84–87, 2010
15. Huppert J, Beaurain J, Steib JP, Bernard P, Dufour T, Hovorka I, et al: Comparison between single- and multi-level patients: clinical and radiological outcomes 2 years after cervical disc replacement. **Eur Spine J 20:**1417–1426, 2011
16. Kellgren JH, Lawrence JS: Radiological assessment of osteoarthrosis. **Ann Rheum Dis 16:**494–502, 1957
17. Kulkarni V, Rajshekhar V, Raghuram L: Accelerated spondylotic changes adjacent to the fused segment following central cervical corpectomy: magnetic resonance imaging study evidence. **J Neurosurg 100 (1 Suppl Spine):**2–6, 2004
18. Lopez-Espina CG, Amirouche F, Havalad V: Multilevel cervical fusion and its effect on disc degeneration and osteophyte formation. **Spine (Phila Pa 1976) 31:**972–978, 2006
19. Matsunaga S, Kabayama S, Yamamoto T, Yone K, Sakou T, Nakanishi K: Strain on intervertebral discs after anterior cervical decompression and fusion. **Spine (Phila Pa 1976) 24:**670–675, 1999
20. Murrey D, Janssen M, Delamarter R, Goldstein J, Zigler J, Tay B, et al: Results of the prospective, randomized, controlled multicenter Food and Drug Administration investigational device exemption study of the ProDisc-C total disc replacement versus anterior discectomy and fusion for the treatment of 1-level symptomatic cervical disc disease. **Spine J 9:**275–286, 2009
21. Phillips B, Ball C, Sackett D, Badenoch D, Straus S, Haynes B, et al: Oxford Centre for Evidence-Based Medicine – levels of evidence (March 2009). **Centre for Evidence Based**

Medicine. (http://www.cebm.net/oxford-centre-evidence-based-medicine-levels-evidence-march-2009/) [Accessed July 22, 2014]
22. Pracyk JB, Traynelis VC: Treatment of the painful motion segment: cervical arthroplasty. **Spine (Phila Pa 1976) 30 (16 Suppl):**S23–S32, 2005
23. Quan GMY, Vital JM, Hansen S, Pointillart V: Eight-year clinical and radiological follow-up of the Bryan cervical disc arthroplasty. **Spine (Phila Pa 1976) 36:**639–646, 2011
24. Reitman CA, Hipp JA, Nguyen L, Esses SI: Changes in segmental intervertebral motion adjacent to cervical arthrodesis: a prospective study. **Spine (Phila Pa 1976) 29:**E221–E226, 2004
25. Robertson JT, Papadopoulos SM, Traynelis VC: Assessment of adjacent-segment disease in patients treated with cervical fusion or arthroplasty: a prospective 2-year study. **J Neurosurg Spine 3:**417–423, 2005
26. Suchomel P, Jurák L, Beneš V III, Brabec R, Bradác O, Elgawhary S: Clinical results and development of heterotopic ossification in total cervical disc replacement during a 4-year follow-up. **Eur Spine J 19:**307–315, 2010
27. Wigfield C, Gill S, Nelson R, Langdon I, Metcalf N, Robertson J: Influence of an artificial cervical joint compared with fusion on adjacent-level motion in the treatment of degenerative cervical disc disease. **J Neurosurg 96 (1 Suppl):**17–21, 2002
28. Zigler JE, Delamarter R, Murrey D, Spivak J, Janssen M: ProDisc-C and anterior cervical discectomy and fusion as surgical treatment for single-level cervical symptomatic degenerative disc disease: five-year results of a Food and Drug Administration study. **Spine (Phila Pa 1976) 38:**203–209, 2013
29. Zindrick M, Harris MB, Humphreys SC, O'Leary PT, Schneiderman G, Watters WC III, et al: Cervical disc arthroplasty. **J Am Acad Orthop Surg 18:**631–637, 2010

Author Contributions

Acquisition of data: Davis, Nunley, Kim, Hisey, Jackson, Bae, Hoffman, Gaede, Danielson, Gordon. Analysis and interpretation of data: Davis. Drafting the article: Davis. Critically revising the article: all authors. Reviewed submitted version of manuscript: all authors. Approved the final version of the manuscript on behalf of all authors: Davis.

Supplemental Information

Previous Presentation

Some results from this paper have been presented at the North American Spine Society Meeting in 2011, 2012, and 2013; International Society for Advancement of Spine Surgery Meeting in 2010 and 2013; Cervical Spine Research Society Meeting in 2011, 2012, and 2013; Eurospine in 2011; and AANS/CNS Joint Spine Section Meeting in 2011.

Correspondence

Reginald J. Davis, Department of Neurosurgery, Greater Baltimore Neurosurgical Associates, 6535 N. Charles St., Ste. 600, Baltimore, MD 21204. email: rjdavismd@aol.com.
