# Supplementary material for: Adjacent segment degeneration or disease after cervical total disc replacement: a meta-analysis of randomized controlled trials
Source: J Orthop Surg Res. 2018 Oct 3;13:244. doi: 10.1186/s13018-018-0940-9 (PMC6169069; doi:10.1186/s13018-018-0940-9)
Supplement: Supplementary file 2 — File S1. Original data of 11 included articles. (ZIP 12 mb) [file 13018_2018_940_MOESM2_ESM.zip › 11 included articles and original data referred in this article/11 included articles/31 Phillips, F M(US).pdf]

Spine

SPINE Volume 40, Number 10, pp 674-683
©2015, Wolters Kluwer Health, Inc. All rights reserved.

RANDOMIZED TRIAL

Long-term Outcomes of the US FDA IDE Prospective, Randomized Controlled Clinical Trial Comparing PCM Cervical Disc Arthroplasty With Anterior Cervical Discectomy and Fusion

Frank M. Phillips, MD,* Fred H. Geisler, MD, PhD,† Kye M. Gilder, PhD,‡ Christopher Reah, PhD,§ Kelli M. Howell, MS,§ and Paul C. McAfee, MD, MBA¶

Study Design. Prospective, multicenter, randomized clinical trial.

Objective. To evaluate the long-term safety and effectiveness of the PCM Cervical Disc compared with anterior cervical discectomy and fusion (ACDF) in treatment of patients with symptomatic single-level degenerative spondylosis between C3–C4 and C7–T1 with or without prior cervical fusion.

Summary of Background Data. The 2-year results of the PCM Cervical Disc trial have been reported previously. The current study reports the long-term results of the same trial.

Methods. Patients with single-level cervical spondylosis and radiculopathy with or without myelopathy unresponsive to nonoperative treatment were enrolled. The per protocol patient sample at 5 years included 293 patients (163 PCM, 130 ACDF). Adverse events and secondary surgical procedures are reported on the cohorts through current follow-up, which include 110 patients (68 PCM, 42 ACDF) at 7 years.

Results. At 5 years postoperative, all patient-reported outcomes—neck and arm pain visual analogue scale score, neck disability index, and general health (36-Item Short Form Health Survey physical and mental component scores: physical component summary, mental component summary)—were significantly improved from baselines in both groups, and mean scores were significantly better in the PCM group for neck disability index ($P = 0.001$), neck pain ($P = 0.002$), general health ($P_{\text{physical component summary}} = 0.014$; $P_{\text{mental component summary}} = 0.004$), and patient satisfaction ($P = 0.005$). PCM patients trended toward fewer 2- to 7-year device-related serious adverse events (1/214, 0.5% PCM; 2/190, 1.1% ACDF) and secondary surgical procedures (7/211, 3.3% PCM; 14/290, 7.6% ACDF). Adjacent-level degeneration was radiographically more frequent after ACDF (33.1% PCM, 50.9% ACDF; $P = 0.006$) and was the primary indication for the increase in late-term secondary surgical procedures after ACDF.

Conclusion. The long-term results show good clinical outcomes after ACDF and PCM arthroplasty. PCM patients showed greater improvement in neck disability index and neck pain scores with a lower rate of radiographical adjacent-level degeneration and a trend toward fewer secondary surgical procedures. These data support PCM arthroplasty to be a viable and sustainable alternative to ACDF.

Key words: cervical total disc replacement, PCM, ACDF, arthroplasty, randomized clinical trial, long-term outcomes.

Level of Evidence: 1

Spine 2015;40:674–683

From the *Midwest Orthopaedics at Rush University Medical Center, Chicago, IL; †Chicago Back Institute, Chicago, IL; ‡Kye Gilder Consulting, LLC, San Diego, CA; §NuVasive, Inc., San Diego, CA; and ¶University of Maryland St. Joseph Medical Center, Baltimore, MD.

Acknowledgment date: July 20, 2014. Revision date: December 26, 2014. Acceptance date: January 28, 2015.

The device(s)/drug(s) is/are FDA approved or approved by corresponding national agency for this indication.

Nuvasive funds were received to support this work.

Relevant financial activities outside the submitted work: board membership, consultancy, employment, payment for manuscript preparation, expert testimony, grants, payment for lecture, patents, royalties, stocks.

Address correspondence and reprint requests to Frank M. Phillips, MD, Midwest Orthopaedics at Rush University Medical Center, 1611 W. Harrison St, Suite 300, Chicago, IL 60612; E-mail: fphillips@rushortho.com

DOI: 10.1097/BRS.0000000000000869

Cervical total disc replacement has been studied as a motion-preserving alternative to anterior cervical discectomy and fusion (ACDF). Several high-level clinical trials have been performed and their 2-year results presented with notable consistency.[1–5] Longer-term outcomes are now becoming more widely available.[6–13]

The 2-year results of a US Food and Drug Administration investigational device exemption (IDE) prospective, randomized clinical trial on a nonconstrained disc replacement device, the PCM Cervical Disc (NuVasive Inc., San Diego, CA), *versus* ACDF have been reported.[5] The results of the same trial with complete follow-up out to 5 years and ongoing follow-up up to 7 years are reported herein.

Copyright © 2015 Wolters Kluwer Health, Inc. Unauthorized reproduction of this article is prohibited.

MATERIALS AND METHODS

Study Design

A prospective, multicenter, randomized, institutional review board–approved clinical trial was conducted under a Food and Drug Administration–approved IDE to compare the safety and effectiveness of the PCM Cervical Disc with ACDF with allograft and plate in treating patients with a degenerated cervical disc at 1 level from C3–C4 to C7–T1. The details of the PCM device, surgical techniques of both treatment groups, and the trial design were described in the publication of the 2-year results.[5] A total of 416 patients were randomized (224 PCM, 192 ACDF) at 24 investigational sites within the United States. Of the randomized patients, 218 PCM and 185 ACDF patients underwent surgical treatment between January 2005 and December 2007, including 49 (29 PCM, 20 ACDF), approximately 12%, with prior adjacent or nonadjacent single-level fusions. Patients were evaluated preoperatively, intraoperatively, immediately postoperatively, and then at 1.5, 3, 6, and 12 months, and annually thereafter. Of the treated patients, 163 PCM and 130 ACDF patients were considered per protocol at 5 years postoperative. Observable data were available through 7 years postoperative on 68 PCM and 42 ACDF patients, among whom safety measures such as adverse events and rates of secondary surgery are reported. A flow diagram of patient accountability is presented in Figure 1.

Fisher exact test was used to compare categorical variables and success rates between the PCM and ACDF groups. To compare continuous variables, either the *t* test or the Mann-Whitney-Wilcoxon test was used. To assess statistically significant improvement within each treatment group from preoperative to the latest visit, a paired *t* test was used. Reported *P* values are 2-sided and unadjusted for multiplicity. Statistical analyses were performed using SAS software (Version 9.3 for Windows, Copyright© 2002–2010, SAS Institute Inc., Cary, NC) and R software (Version 3.01.1, Copyright© 2013, The R Foundation for Statistical Computing, Vienna, Austria).

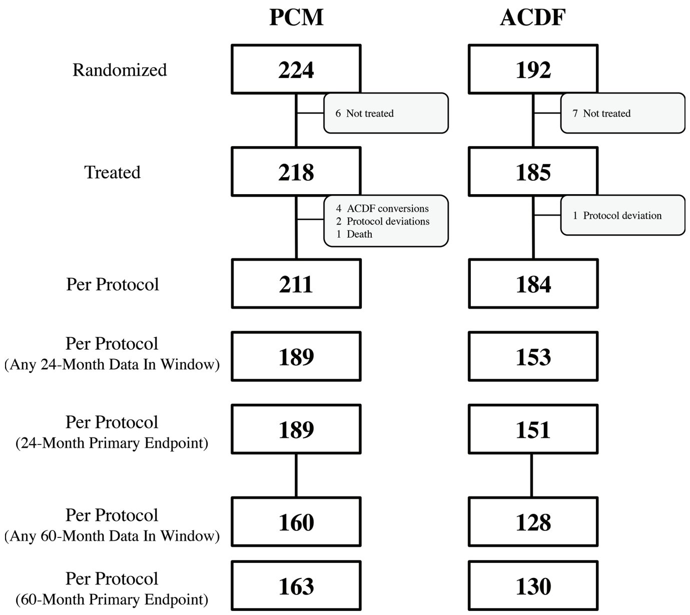

Figure 1. Patients included in the long-term analyses are those who are in the per protocol population, which is any 5-year data in window or evaluable for the primary overall success endpoint. PCM patients denoted as "ACDF conversions" were intraoperatively switched to undergo the ACDF procedure because of surgical reasons. ACDF indicates anterior cervical discectomy and fusion.

Clinical and Radiographical Outcome Assessments

Evaluations included patient-reported self-assessments, physical and neurological examination, and quantitative and qualitative radiographical analysis. Validated self-assessment outcomes measures included neck disability index (NDI) (a measure of pain-related dysfunction),[14,15] 36-Item Short Form Health Survey (SF-36) mental and physical general health surveys (mental component summary and physical component summary),[16] and neck and arm pain scores on a 0- to 100-mm visual analogue scale (VAS). Success on NDI, neck and worst arm pain, and SF-36 scores was defined as a minimum 20%, 20-mm, and 15% improvement over the preoperative score, respectively. Neurological examinations evaluated muscle strength, sensory deficit, and reflex functions. Neurological success was defined as the postoperative maintenance or improvement in each of the individual neurological evaluations compared with the preoperative status. Additional clinical assessments included dysphagia (0- to 100-mm VAS) and patient satisfaction (0- to 100-mm VAS and questionnaire). All adverse event occurrences were recorded prospectively, categorized, and evaluated for causality. Treatment group trends were also assessed.

Plain radiographs were obtained and consisted of neutral lateral and anteroposterior, flexion/extension, and lateral bending films. Preoperative computed tomographic scans and magnetic resonance images were also collected. All radiographical analyses were performed by an independent radiographical review service (Medical Metrics Inc., Houston, TX). Fusion was defined as evidence of continuous bridging bone between the adjacent endplates of the involved motion segment, radiolucent lines at 50% of the graft-vertebra interfaces or less, and 2° or less segmental rotation on lateral flexion/extension radiographs. Disc heights were measured at each time point, and normal disc height defined as 80% or more that of the disc superiorly adjacent to the index operative level. Success in maintenance of disc height over time was defined as measuring 80% or more of the postoperative height. Heterotopic ossification was classified as grade 0 (none) through grade IV (ankylosed).[17] Signs of adjacent-level degeneration were independently graded on the basis of observation of disc height loss (compared with adjacent normal discs), presence and size of osteophytes, and endplate sclerosis (thickening and density compared with adjacent normal discs). Each observation was scored and a total score summed to within a range of 0 to 9.[18] Total score was further categorized on a 4-point scale of degeneration: 0 (none: total score of 0), 1 (mild: total score of 1–3), 2 (moderate: total score of 4–6), and 3 (severe: total score of 7–9). Rates of success on this measure were reported and required no worsening of the score compared with preoperative baseline.

Copyright © 2015 Wolters Kluwer Health, Inc. Unauthorized reproduction of this article is prohibited.

RESULTS

Baseline patient demographics and treatment characteristics have been previously reported, showing no differences between groups.[5] At 5 years, the overall follow-up rate, based on the modified intent-to-treat population, was 74.8% (163/218) in the PCM group and 70.3% (130/185) in the ACDF group. Observable data were available through patients having reached 7 years postoperatively on 31.2% (68/218) PCM and 22.7% (42/185) ACDF patients, among whom safety measures such as adverse events and rates of secondary surgery are reported.

Neck Disability Index

Mean NDI improved significantly over preoperative scores at all postoperative time points in both treatment groups ($P < 0.001$). The mean improvement in NDI was greater in the PCM group at all postoperative time points than in the ACDF group (Figure 2), the difference increasing over time. Mean NDI score at 5 years was significantly lower in the PCM group (20.4 *vs*. 28.5; $P = 0.001$). At 5 years, NDI success, defined as 20% improvement or more compared with preoperative score, was achieved by 85.0% in the PCM group compared with 74.2% in the ACDF group ($P = 0.026$) (Table 1 and Figure 3).

Neck and Worst Arm Pain

Mean VAS pain scores improved significantly over preoperative scores for all postoperative time points ($P < 0.001$) in both treatment groups (Figure 4A,B). The mean neck pain VAS score was lower in the PCM group at all postoperative time points than that in the ACDF group, significantly so at 5 years ($P = 0.002$). Mean VAS scores for worst arm pain were not statistically different between groups at any time point ($P > 0.05$) (Figure 4B). At 5 years, success on VAS pain scores, defined as a clinically significant change of 20 mm or more compared with preoperative score, was not different between groups for either neck pain ($P = 0.502$) or worst arm pain ($P = 0.070$) (Table 1).

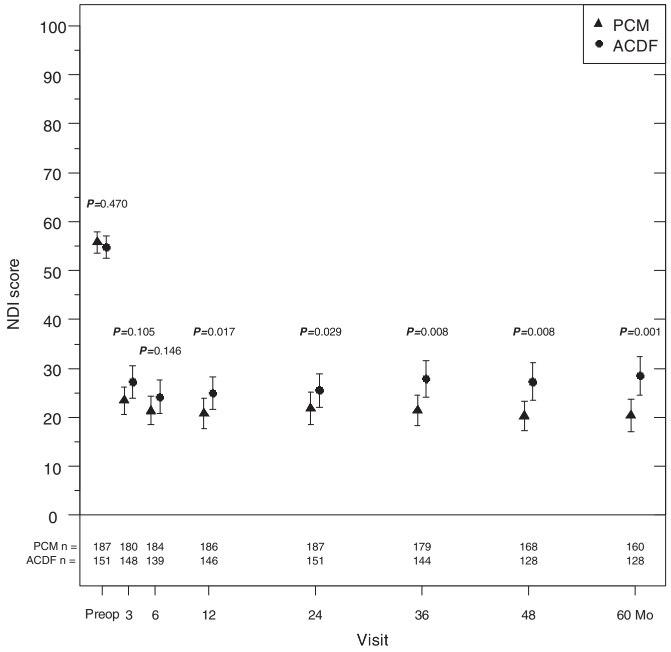

Figure 2. Comparison of mean NDI scores between the PCM and ACDF groups. Only per protocol patients who have both a preoperative and a 5-year NDI score are included, with sample sizes denoted in the bottom panel. Error bars represent 95% confidence intervals for the mean, and unadjusted *P* values are based on Mann-Whitney-Wilcoxon test (2-sided). NDI indicates Neck Disability Index; ACDF, anterior cervical discectomy and fusion.

Short Form-36 Health Survey

At all postoperative time points for both the mean SF-36 physical component summary and mental component summary scores, both treatment groups improved significantly over preoperative scores ($P < 0.001$) (Figure 5A,B). At 5 years, 73.7% of the PCM group reported clinically significant improvement of 15% or more in physical component summary scores compared with 56.7% of the ACDF group ($P = 0.004$), whereas 46.2% of the PCM group reported clinically significant improvement in mental component summary scores compared with 54.3% of the ACDF group ($P = 0.189$) (Table 1).

Neurological Success

At 5 years, the neurological success rate was 92.4% (146/158) in the PCM group compared with 87.5% (112/128) in the ACDF group ($P = 0.229$) (Table 1).

Dysphagia

Mean VAS scores for swallowing difficulty at 5 years were 8.8/100 mm in the PCM group and 16.9/100 mm in the ACDF group ($P = 0.001$) (Table 1).

Patient Satisfaction

At 5 years, the mean patient satisfaction VAS scores were 86.9/100 mm and 78.3/100 mm for the PCM and ACDF groups, respectively ($P = 0.005$) (Table 1). When asked whether they were satisfied with their 5-year outcomes, 88.8% of the PCM patients responded either "very" or "moderately" satisfied, compared with 78.7% of those in the ACDF group. Similarly, 94.4% and 85.0% would "definitely" or "probably" recommend the treatment in the PCM and ACDF groups, respectively.

Serious Adverse Events

Serious adverse events within the first 2 years postoperative were previously reported[5] and showed no statistical difference in incidence between the PCM and ACDF groups. New serious adverse events between 2 years postoperative and current follow-up (up to 7 yr) occurred in 45/214 (21.0%) as-treated PCM patients and 33/190 (17.4%) as-treated ACDF patients (denominators include crossover between treatment groups). The majority of these events were systemic or medical in nature, with 1 (0.5%) new device-related serious adverse event in the PCM group and 2 (1.1%) in the ACDF group.

Copyright © 2015 Wolters Kluwer Health, Inc. Unauthorized reproduction of this article is prohibited.

TABLE 1. Summary of Clinical Effectiveness Endpoints at 5 Years

Component	PCM (N = 163)	ACDF (N = 130)	*P**
Clinical endpoints			
Neck Disability Index (≥20% improvement)	85.0% (136/160)	74.2% (95/128)	0.026
Neck Disability Index (≥15-point improvement)	80.0% (128/160)	69.5% (89/128)	0.054
Neck Pain VAS (≥20 mm improvement)	71.9% (115/160)	75.8% (97/128)	0.502
Worst arm pain VAS (≥20 mm improvement)	80.6% (129/160)	71.1% (91/128)	0.070
SF-36 PCS (≥15% improvement)	73.7% (115/156)	56.7% (72/127)	0.004
SF-36 MCS (≥15% improvement)	46.2% (72/156)	54.3% (69/127)	0.189
Neurological success (maintained or improved)	92.4% (146/158)	87.5% (112/128)	0.229
Dysphagia swallowing VAS, mean (SD) mm	8.8 (15.7)	16.9 (24.2)	0.001
Myelopathy (Nurick; maintained or improved)	99.4% (156/157)	96.9% (124/128)	0.177
Patient satisfaction VAS, mean (SD) mm	86.9 (21.6)	78.3 (29.6)	0.005
Radiographical endpoints			
Range of motion, mean (SD), degrees	5.2 (3.8)	0.5 (0.5)	NA
Fusion rate†	6.0% (9/150)	94.4% (119/126)	NA
Normal disc height (≥80% of superior level)	94.9% (149/157)	82.9% (102/123)	0.001
Disc height maintenance (≥80% of postoperative)	87.4% (132/151)	80.3% (94/117)	0.129
Incidence of adjacent-level degeneration[18]			
Superior	33.1% (48/145)	50.9% (54/106)	0.006
Inferior	49.2% (58/118)	51.7% (46/89)	0.779
Incidence of heterotopic ossification[17]			
Grade III	6.7% (10/149)	NA	NA
Grade IV	6.0% (9/149)		

Patients included are those who are in the per protocol (any 5-yr data in window or evaluable for the primary overall success endpoint) population.

**Fisher exact test (2-sided) for categorical variables and Mann-Whitney-Wilcoxon test (2-sided) for continuous variables.*

†Fusion defined as evidence of continuous bridging bone between the adjacent endplates of the involved motion segment, radiolucent lines at ≤50% of the graft-vertebra interfaces, and ≤2° of segmental rotation on lateral flexion/extension radiographs.

ACDF indicates anterior cervical discectomy and fusion; VAS, visual analogue scale; SF-36, 36-Item Short Form Health Survey; PCS, physical component summary; MCS, mental component summary; NA, not applicable.

Subsequent Secondary Surgical Interventions

As with other IDE clinical trials of cervical arthroplasty, subsequent secondary surgical intervention (SSSI) was defined as any revision, removal, reoperation, or supplemental fixation. Through 5 years, 8.1% (17/211) patients in the PCM group and 12.0% (22/184) patients in the ACDF group required an SSSI ($P = 0.237$). At 7 years, these rates increase slightly to 8.5% (18/211) PCM and 13.0% (24/184) ACDF ($P = 0.190$) (Table 2). The new events occurring between 2 and 7 years included 6 removals of the PCM device for pain and/or device migration, 3 of those after a trauma, and 1 ACDF for adjacent segment disease. Four of the PCM removals were converted to single-level ACDF, 1 to a 2-level ACDF, and 1 to another arthroplasty. In the ACDF group, 13 of the 14 late-term secondary surgical procedures were for adjacent-level degeneration and 1 was performed for a pseudarthrosis. Patients were treated with either revision and extension of fusion or cervical arthroplasty.

Figure 6 illustrates the Kaplan-Meier curves comparing the time to first occurrence of an SSSI between groups, showing the incidence of SSSI at each time point out to 7 years. The curves are not statistically different ($P = 0.123$) but begin separating at about 3 years where, as follow-up continues, there is a greater probability of additional surgery in ACDF patients than in PCM patients.

Radiographical Findings

At 5 years, the mean flexion/extension range of motion in the PCM group was 5.2° (SD: 3.8°; range: 0°–16.1°) compared with 0.5° (SD: 0.5°; range: 0°–4.1°) for the ACDF group (Table 1 and Figure 7). The radiographical success rate in the PCM group was 94.0% (141/150), with 6% (9/149)

Copyright © 2015 Wolters Kluwer Health, Inc. Unauthorized reproduction of this article is prohibited.

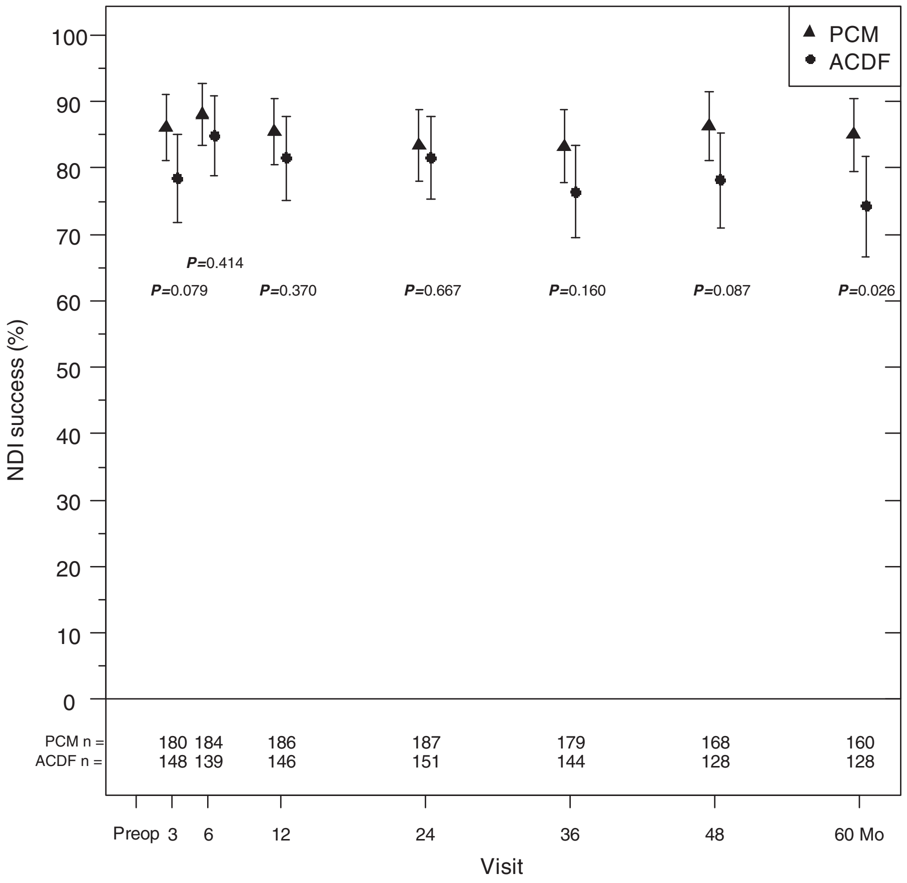

Figure 3. Comparison of NDI success between the PCM and ACDF groups. NDI success defined as a 20% or more improvement compared with the preoperative score. Only per protocol patients who have both a preoperative and a 5-year NDI score are included, with sample sizes denoted in the bottom panel. Error bars represent 95% confidence intervals for proportions, and unadjusted *P* values are based on Fisher exact test (2-sided). NDI indicates Neck Disability Index; ACDF, anterior cervical discectomy and fusion.

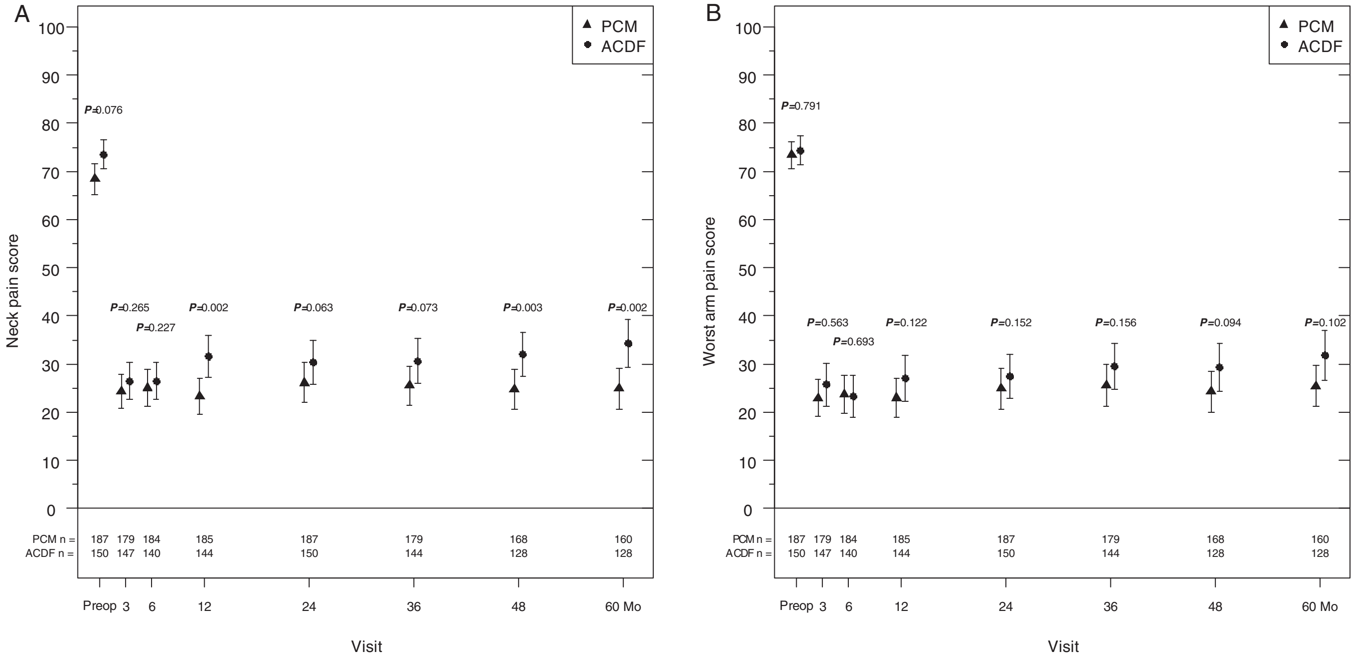

Figure 4. Comparison of mean neck pain visual analogue scale (VAS) score (**A**) and mean worst arm pain VAS score (**B**) between PCM and ACDF groups. Only per protocol patients who have both a preoperative and a 5-year neck pain or worst arm VAS score are included, with sample sizes denoted in the bottom panel. Error bars represent 95% confidence intervals for the mean, and unadjusted *P* values are based on Mann-Whitney-Wilcoxon test (2-sided). ACDF indicates anterior cervical discectomy and fusion.

Copyright © 2015 Wolters Kluwer Health, Inc. Unauthorized reproduction of this article is prohibited.

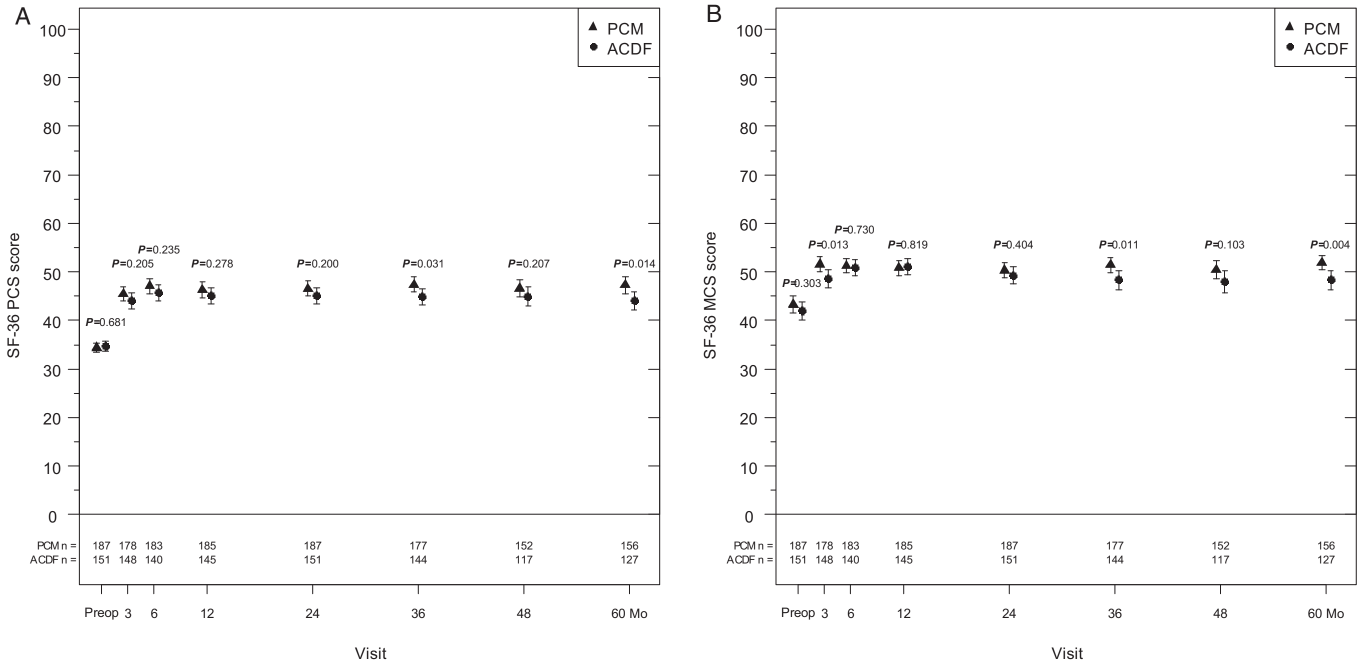

Figure 5. Comparison of mean SF-36 physical component summary (PCS) (**A**) and mean SF-36 mental component summary (MCS) (**B**) scores between PCM and ACDF groups. Only per protocol patients who have both a preoperative and a 5-year PCS and MCS are included, with sample sizes denoted in the bottom panel. Error bars represent 95% confidence intervals for the mean, and unadjusted *P* values are based on *t* test (2-sided). SF-36 indicates 36-Item Short Form Health Survey; ACDF, anterior cervical discectomy and fusion.

demonstrating grade IV heterotopic ossification with bony ankylosis. The fusion rate in the ACDF group was 94.4% (119/126).

Disc height at the operative level was within 80% of that of preoperative adjacent levels in 94.9% of the PCM group at 5 years, compared with 82.9% in the ACDF group (P = 0.001) (Table 1).

Degeneration at the superiorly adjacent disc level was identified per radiographical protocol in 33.1% (48/145) of PCM patients and 50.9% (54/106) of the ACDF patients

TABLE 2. Summary of Subsequent Secondary Surgical Interventions Through 7 Years

	≤2 yr		2–5 yr		>5 yr	
	PCM	ACDF	PCM	ACDF	PCM	ACDF
**Total**	**11**	**10**	**6**	**12**	**1**	**2**
Removal (reason below)	8	10	6	12		2
Pain	3	1	2			
Device migration, subsidence	4		4			
Adjacent segment disease		6		11		2
Nonunion		3		1		
Unknown	1					
Reoperation (additional decompression)	3					
Supplemental fixation (adjacent ACDF)					1	

ACDF indicates anterior cervical discectomy and fusion.

Copyright © 2015 Wolters Kluwer Health, Inc. Unauthorized reproduction of this article is prohibited.

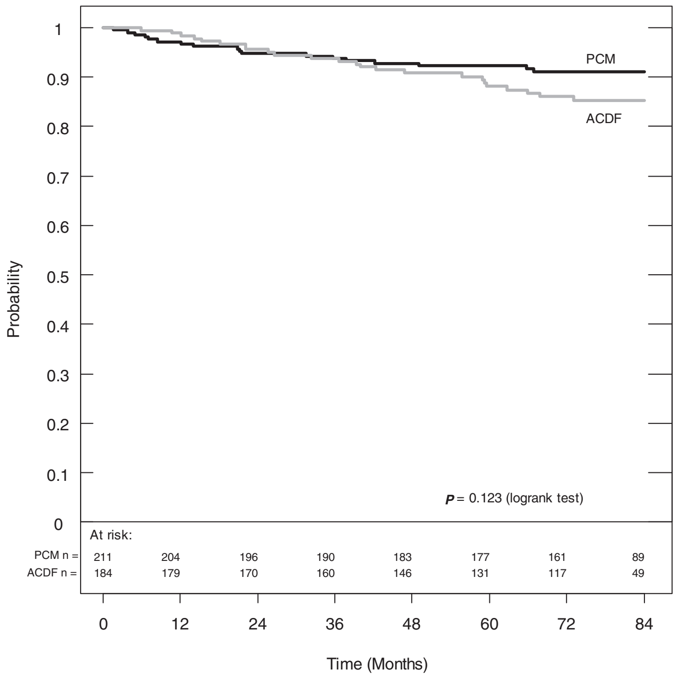

Figure 6. Kaplan-Meier curves comparing the time to first occurrence of a subsequent secondary surgical intervention between groups. ACDF indicates anterior cervical discectomy and fusion.

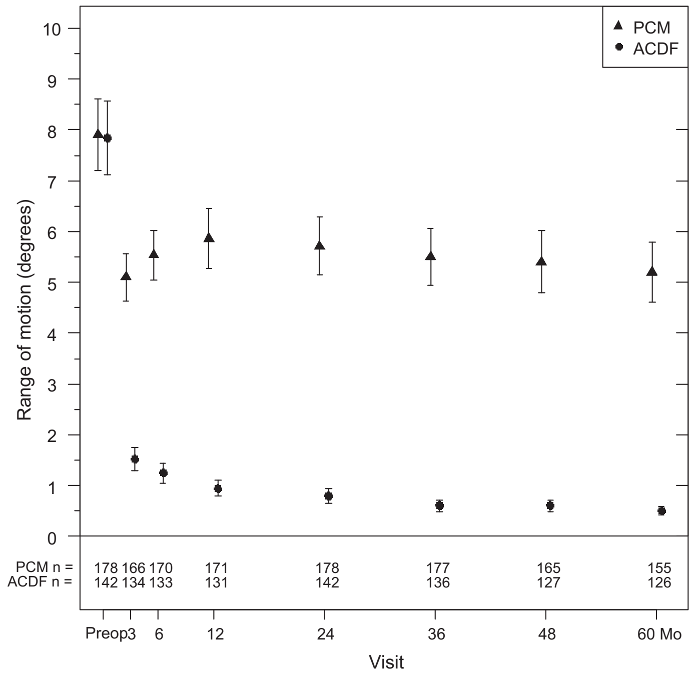

Figure 7. Comparison of mean flexion/extension range of motion (ROM) between the PCM and ACDF groups. Only per protocol patients who have both a preoperative and a 5-year ROM are included, with sample sizes denoted in the bottom panel. Error bars represent 95% confidence intervals for the mean. ACDF indicates anterior cervical discectomy and fusion.

(P = 0.006) (Table 1). Degeneration at the inferiorly adjacent level was radiographically identified about equally in both groups: 49.2% (58/118) in PCM and 51.7% (46/89) in ACDF (P = 0.779).

Case Example

Figure 8A–F shows the radiographical chronology of a 50-year-old male who presented with significant neck and arm pain (preoperative VAS scores of 77/100 and 91/100, respectively) because of C6–C7 spondylosis and underwent cervical decompression and arthroplasty using the PCM Cervical Disc. Preoperative (Figure 8A), immediate postoperative (Figure 8B), and 2-year (Figure 8C) lateral neutral radiographs showing good postoperative restoration and maintenance of disc height and alignment. Lateral neutral (Figure 8D), flexion (Figure 8E), and extension (Figure 8F) radiographs at 4-year follow-up, showing continued maintenance of correction, as well as continued motion, despite mild (grade 1) heterotopic ossification posteriorly. The patient returned to all activities within 6 weeks, and at 6 years after surgery, he reports no pain-related disability and complete satisfaction with his treatment.

DISCUSSION

The results of the current study demonstrated excellent long-term clinical outcomes in both the PCM and ACDF groups in the treatment of symptomatic single-level cervical spondylosis. Clinical outcomes with the use of the PCM Cervical Disc were superior to instrumented anterior cervical fusion, with significantly better mean NDI, VAS, and SF-36 scores; higher NDI success rate; lower rate of prolonged dysphagia; and greater patient satisfaction scores at 5 years of follow-up. Cervical motion in PCM patients was maintained at 5 years. In addition, patients undergoing arthroplasty with the PCM Cervical Disc exhibited statistically lower rates of radiographical adjacent-level degeneration and incurred fewer secondary surgical procedures than ACDF controls out to 7 years, representing the longest reported follow-up in the US cervical arthroplasty IDE trials to date.

Results from several prospective, randomized clinical trials of various cervical total disc replacement device designs have been reported, all finding at least equivalent clinical outcome measures, rates of secondary surgical interventions, and overall success compared with ACDF at 2 years of follow-up.[1–5] In a meta-analysis of 2-year outcomes studies, pooled results from 4 cervical disc replacement IDE trials concluded that cervical arthroplasty was superior to ACDF on rate of neurological success, secondary surgical interventions, and overall success, whereas at least equivalent on NDI success.[19] More recently, 5-year outcome data have started to be reported for cervical arthroplasty.[6–13] The current study supports the durability of results for the PCM arthroplasty device out to at least 5 years, with support for 7-year survivability.

Across multiple trials, including the current study, clinically and statistically significant improvements in patient-reported outcomes are shown after both fusion and cervical arthroplasty procedures, suggesting that both are clinically effective treatment options for symptomatic cervical spondylosis. Interestingly, however, some clinical measures seem to be superior after cervical arthroplasty. Notably, higher mean NDI scores and greater percentage of NDI success rates (>15-point or

Copyright © 2015 Wolters Kluwer Health, Inc. Unauthorized reproduction of this article is prohibited.

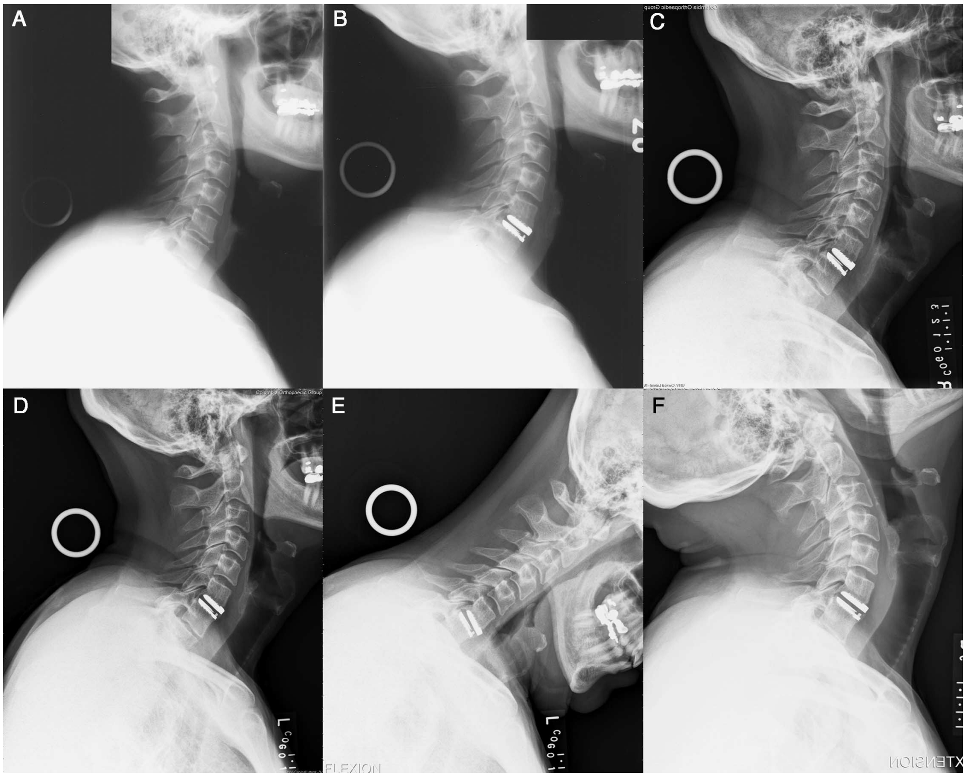

Figure 8. Radiographical results of a representative PCM patient. Lateral neutral radiographs at preoperative (**A**), immediate postoperative (**B**), and 2-year follow-up visits (**C**). Lateral neutral (**D**), flexion (**E**), and extension (**F**) radiographs at 4-year follow-up.

>20% improvement from baseline) are consistently reported for arthroplasty over fusion across all US IDE trials,[1–13,19] although not always statistically different. Although patient euphoric bias (having received a motion-preserving option in a randomized trial *vs*. fusion) has been suggested to be a factor in 2-year results, it is unlikely that it continues to significantly affect results 5 or more years after the randomization process as was observed in the current trial.[20] In the current study, the differences between groups also increased over time, making euphoric bias less likely, and perhaps explained by survivorship analysis, which showed that fusion patients more often presented with late-term onset of new symptoms, requiring secondary surgery at the adjacent level. These findings suggest that cervical arthroplasty may have a protective effect against accelerated adjacent-level degeneration, which may translate to patient-reported assessment of pain-related function.

Neck pain likely follows a similar pattern with benefit of arthroplasty over fusion. Again, these results are consistent across arthroplasty trials, in which improvement in neck pain and maintenance of that improvement are better after arthroplasty, even while improvement in arm pain is similar between groups. These results suggest that both procedures provide effective neural decompression, but motion preservation may provide better relief of neck pain, possibly associated with the more physiologic regional kinematics[21] or decreased adjacent segment degeneration.

Another interesting finding in the current study was a higher rate of dysphagia in the fusion group even at 5 years postoperative. Prior studies have similarly reported lower rates of dysphagia after cervical arthroplasty than those after ACDF.[7,13] Suggested mechanisms include the absence of any anterior profile of the PCM device that sits entirely intradiscal compared with an anterior cervical plate. Others have suggested that applying an anterior plate requires greater esophageal retraction.[22]

In the current trial, both disc height and range of motion at the index level were maintained up to 5 years after PCM. Although heterotopic ossification has been raised as a concern for the long-term mobility of cervical disc replacements, only 6% of PCM patients were classified as grade IV or ankylosed at 5 years postoperative, or alternatively, 94% of PCM patients maintained at least 2° of flexion-extension motion, with an average of 5.2°. Loss of disc height was seen early in the postoperative follow-up period in the ACDF group, continuing to decrease through 12 months but then without significant decrease at later time points where it is presumed that the levels fused in the subsided position. Loss of lordosis due to subsidence can cause axial pain and stresses at adjacent levels.[23] Indeed, a higher incidence of radiographical degeneration of the superiorly adjacent segment was observed after ACDF than that after PCM. This trend was seen at 2 years and continued to a significant degree out to 5 years. It should be noted, however, that not all radiographically identified degeneration is necessarily symptomatic or requiring surgical intervention. In the current study, the incidence of secondary surgery was lower for PCM patients than that for ACDF patients, even out to 7 years, as the survivability curves of each treatment diverge in favor of arthroplasty starting at about 3 years postoperative and continue to separate as an increasingly greater number of ACDF patients require additional surgical procedures, mainly for adjacent-level symptoms. These results are similar to those reported in other long-term reports

Copyright © 2015 Wolters Kluwer Health, Inc. Unauthorized reproduction of this article is prohibited.

of cervical arthroplasty. The rates of reported combined late-term secondary surgical procedures (at index and adjacent levels) are in the range of 2.9% at 5 years for the ProDisc-C device,[7] 6.9% at 5 years for the Prestige disc,[6] and 7.9% at 4 years for the Bryan disc[12] *versus* 14.5%, 15.8%, and 8.6% of respective ACDF controls. Although cost metrics were not directly studied in this trial, given that repeat surgery is the primary driver of cost after an index surgery, these results lend support to arthroplasty as a cost-effective treatment option, assuming similar costs at intervention and fewer costs and better maintenance of treatment in the long term.

The follow-up rates in the current trial (75% PCM, 70% ACDF) compare favorably with what has been reported in these other long-term cervical arthroplasty studies: 74.8% Bryan disc *versus* 62.4% of controls,[12] 72.7% ProDisc-C *versus* 63.5% of controls,[7] and 52% Prestige disc *versus* 48% of controls,[6] lending strength to these results.

Overall, this prospective, multicenter, randomized Food and Drug Administration–approved IDE clinical trial found that cervical disc arthroplasty with the PCM Cervical Disc provides sustainably effective treatment of symptomatic single-level cervical spondylosis. Compared with instrumented anterior cervical fusion, equivalent or better clinical outcomes were achieved while preserving cervical motion. In addition, PCM patients had improved function, lower rate of prolonged dysphagia, and greater patient satisfaction at 5 years postoperative, with a lower incidence of adjacent-level degeneration and secondary surgical procedures out to 7 years postoperative. These results confirm prior reports of clinical success after cervical arthroplasty and support the role of the PCM Cervical Disc as an effective alternative to ACDF.

➢ Key Points

- ❑ Both PCM arthroplasty and ACDF resulted in excellent long-term clinical results in the treatment of single-level degenerative spondylosis with or without prior fusions.
- ❑ At 5 years, the PCM group showed greater improvements in neck pain, NDI, and general health, and higher patient satisfaction than the ACDF cohort. Rates of adverse events and secondary surgical procedures trended lower in the PCM cohort out to current follow-up of 7 years.
- ❑ Radiographical metrics showed stability of PCM treatment over time, with continued motion (flexion/extension range of motion at the index level averaged 5.2° [SD: 3.8°; range: 0°–16.1°] at 5 yr), and no significant increase in heterotopic ossification or loss of disc height since 2-year results.
- ❑ Cervical arthroplasty is thought to protect adjacent levels; at 5 years, signs of adjacent-level degeneration were identified in statistically fewer levels adjacent to PCM than to ACDF ($P = 0.006$).

Acknowledgments

The authors thank the other participating investigators for their participation and contributions to the trial: Todd J. Albert, MD; Howard An, MD; Donald Atkins, MD; Sam Bakshian, MD; Clyde T. Carpenter, MD; James B. Chadduck, MD; Ying Chen, MD; Bradford L. Currier, MD; Dave Dewitt, MD; Amir H. Fayazzi, MD; Allan H. Fergus, MD; Bruce E. Fredrickson, MD; Gary Ghiselli, MD; Karl Greene, MD; Harry Herkowitz, MD; Thomas R. Highland, MD; Alan S. Hillibrand, MD; Patrick D. Ireland, MD; John I. Iskandar, MD; Sanjay Jatana, MD; Saad Khairi, MD; Timothy Kuklo, MD; Daniel Laich, DO; Todd Lanman, MD; Carl Lauryssen, MD; Michael Leahy, MD; Isador Leiberman, MD; John Miles, MD; Jean-Pierre Mobasser, MD; James Mok, MD; Jeffrey Parker, MD; Eric Potts, MD; Mark Rahm, MD; John Regan, MD; Patrick Rhoten, MD; Michael Rosner, MD; Richard Schlenk, MD; Steven R. Schopick, MD; Ashwini Sharan, MD; Michael Steinmetz, MD; Daryl Sybert, MD; Richard A. Tallarico, MD; Robert Tibbs, MD; Justin Tortalani, MD; Eeric Truumees, MD; Peter F. Ullrich, MD; Franco Vigna, MD; David A. Wong, MD; and Hansen A. Yuan, MD.

References

1. Coric D, Nunley PD, Guyer RD, et al. Prospective, randomized, multicenter study of cervical arthroplasty: 269 patients from the Kineflex|C artificial disc investigational device exemption study with a minimum 2-year follow-up: clinical article. *J Neurosurg Spine* 2011;15:348–58.
2. Heller JG, Sasso RC, Papadopoulos SM, et al. Comparison of BRYAN cervical disc arthroplasty with anterior cervical decompression and fusion: clinical and radiographic results of a randomized, controlled, clinical trial. *Spine* 2009;34:101–7.
3. Mummaneni PV, Burkus JK, Haid RW, et al. Clinical and radiographic analysis of cervical disc arthroplasty compared with allograft fusion: a randomized controlled clinical trial. *J Neurosurg Spine* 2007;6:198–209.
4. Murrey D, Janssen M, Delamarter R, et al. Results of the prospective, randomized, controlled multicenter Food and Drug Administration investigational device exemption study of the ProDisc-C total disc replacement versus anterior discectomy and fusion for the treatment of 1-level symptomatic cervical disc disease. *Spine J* 2009;9:275–86.
5. Phillips FM, Lee JY, Geisler FH, et al. A prospective, randomized, controlled clinical investigation comparing PCM cervical disc arthroplasty with anterior cervical discectomy and fusion. 2-year results from the US FDA IDE clinical trial. *Spine* 2013;38:E907–18.
6. Burkus JK, Haid RW, Traynelis VC, et al. Long-term clinical and radiographic outcomes of cervical disc replacement with the Prestige disc: results from a prospective randomized controlled clinical trial. *J Neurosurg Spine* 2010;13:308–18.
7. Delamarter RB, Zigler J. Five-year reoperation rates, cervical total disc replacement versus fusion, results of a prospective randomized clinical trial. *Spine* 2013;38:711–7.
8. Delamarter RB, Murrey D, Janssen ME, et al. Results at 24 months from the prospective, randomized, multicenter Investigational Device Exemption trial of ProDisc-C versus anterior cervical discectomy and fusion with 4-year follow-up and continued access patients. *SAS J* 2010;4:122–8.
9. Mummaneni PV, Amin BY, Wu JC, et al. Cervical artificial disc replacement versus fusion in the cervical spine: a systematic review comparing long-term follow-up results from two FDA trials. *Evid Based Spine Care J* 2012;3:59–66.
10. Quan GM, Vital JM, Hansen S, et al. Eight-year clinical and radiological follow-up of the Bryan cervical disc arthroplasty. *Spine* 2011;36:639–46.

Copyright © 2015 Wolters Kluwer Health, Inc. Unauthorized reproduction of this article is prohibited.

11. Ryu WH, Kowalczyk I, Duggal N. Long-term kinematic analysis of cervical spine after single-level implantation of Bryan cervical disc prosthesis. *Spine J* 2013;13:628–34.
12. Sasso RC, Anderson PA, Riew KD, et al. Results of cervical arthroplasty compared with anterior discectomy and fusion: four-year clinical outcomes in a prospective, randomized controlled trial. *J Bone Joint Surg Am* 2011;93:1684–92.
13. Zigler JE, Delamarter R, Murrey D, et al. ProDisc-C and anterior cervical discectomy and fusion as surgical treatment for single-level cervical symptomatic degenerative disc disease: five-year results of a Food and Drug Administration study. *Spine* 2013;38:203–9.
14. Vernon H, Mior S. The Neck Disability Index: a study of reliability and validity. *J Manipulative Physiol Ther* 1991;14:409–15.
15. Westaway MD, Stratford PW, Binkley JM. The patient-specific functional scale: validation of its use in persons with neck dysfunction. *J Orthop Sports Phys Ther* 1998;27:331–8.
16. McHorney CA, Ware JE Jr, Lu JF, et al. The MOS 36-item Short-Form Health Survey (SF-36): III. Tests of data quality, scaling assumptions, and reliability across diverse patient groups. *Med Care* 1994;32:40–66.
17. McAfee PC, Cunningham BW, Devine J, et al. Classification of heterotopic ossification (HO) in artificial disk replacement. *J Spinal Disord Tech* 2003;16:384–9.
18. Walraevens J, Demaerel P, Suetens P, et al. Longitudinal prospective long-term radiographic follow-up after treatment of single-level cervical disk disease with the Bryan Cervical Disc. *Neurosurgery* 2010;67:679–87.
19. McAfee PC, Reah C, Gilder K, et al. A meta-analysis of comparative outcomes following cervical arthroplasty or anterior cervical fusion: results from 4 prospective multicenter randomized clinical trials and up to 1226 patients. *Spine* 2012;37:943–52.
20. Singh K, Phillips FM, Park DK, et al. Factors affecting reoperations after anterior cervical discectomy and fusion within and outside of a Federal Drug Administration investigational device exemption cervical disc replacement trial. *Spine J* 2012;12:372–8.
21. Cunningham BW, Hu N, Zorn CM, et al. Biomechanical comparison of single- and two-level cervical arthroplasty versus arthrodesis: effect on adjacent-level spinal kinematics. *Spine J* 2010;10:341–9.
22. Tortolani PJ, Cunningham BW, Vigna F, et al. A comparison of retraction pressure during anterior cervical plate surgery and cervical disc replacement: a cadaveric study. *J Spinal Disord Tech* 2006;19:312–7.
23. Katsuura A, Hukuda S, Saruhashi Y, et al. Kyphotic malalignment after anterior cervical fusion is one of the factors promoting the degenerative process in adjacent intervertebral levels. *Eur Spine J* 2001;10:320–4.

Copyright © 2015 Wolters Kluwer Health, Inc. Unauthorized reproduction of this article is prohibited.
