# Supplementary material for: Adjacent segment degeneration or disease after cervical total disc replacement: a meta-analysis of randomized controlled trials
Source: J Orthop Surg Res. 2018 Oct 3;13:244. doi: 10.1186/s13018-018-0940-9 (PMC6169069; doi:10.1186/s13018-018-0940-9)
Supplement: Supplementary file 3 — Table S2. The Cochrane Collaboration’s tool for assessing quality and risk of bias. (PDF 266 kb) [file 13018_2018_940_MOESM3_ESM.pdf]

S2 Table. The Cochrane Collaboration's tool for assessing quality and risk of bias

**Domain**	**Description**	**Review authors' judgement**
**Sequence generation.**	Describe the method used to generate the allocation sequence in sufficient detail to allow an assessment of whether it should produce comparable groups.	Was the allocation sequence adequately generated?
**Allocation concealment.**	Describe the method used to conceal the allocation sequence in sufficient detail to determine whether intervention allocations could have been foreseen in advance of, or during, enrolment.	Was allocation adequately concealed?
**Blinding of participants, personnel and outcome assessors** *Assessments should be made for each main outcome (or class of outcomes).*	Describe all measures used, if any, to blind study participants and personnel from knowledge of which intervention a participant received. Provide any information relating to whether the intended blinding was effective.	Was knowledge of the allocated intervention adequately prevented during the study?
**Incomplete outcome data** *Assessments should be made for each main outcome (or class of outcomes).*	Describe the completeness of outcome data for each main outcome, including attrition and exclusions from the analysis. State whether attrition and exclusions were reported, the numbers in each intervention group (compared with total randomized participants), reasons for attrition/exclusions where reported, and any re-inclusions in analyses performed by the review authors.	Were incomplete outcome data adequately addressed?
**Selective outcome reporting.**	State how the possibility of selective outcome reporting was examined by the review authors, and what was found.	Are reports of the study free of suggestion of selective outcome reporting?
**Other sources of bias.**	State any important concerns about bias not addressed in the other domains in the tool.<br>If particular questions/entries were pre-specified in the review's protocol, responses should be provided for each question/entry.	Was the study apparently free of other problems that could put it at a high risk of bias?
